# Computational study of effective matrix metalloproteinase 9 (MMP9) targeting natural inhibitors

**DOI:** 10.18632/aging.203581

**Published:** 2021-10-04

**Authors:** Naimeng Liu, Xinhui Wang, Hao Wu, Xiaye Lv, Haoqun Xie, Zhen Guo, Jing Wang, Gaojing Dou, Chenxi Zhang, Mindan Sun

**Affiliations:** 1 Department of Nephrology, The First Hospital of Jilin University, Changchun, China; 2 Department of Breast Surgery, The First Hospital of Jilin University, Changchun, China; 3 Department of Oncology, The First Hospital of Jilin University, Changchun, China; 4 Clinical College, Jilin University, Changchun, China; 5 Department of Hematology, The First Clinical Medical School of Lanzhou University, Lanzhou, Gansu, China

**Keywords:** MMP9, drug development, virtual screening, CCRCC, targeted therapy

## Abstract

Object: The present study screened ideal lead natural compounds that could target and inhibit matrix metalloproteinase 9 (MMP9) protein from the ZINC database to develop drugs for clear cell renal cell carcinoma (CCRCC)-targeted treatment.

Methods: Discovery Studio 4.5 was used to compare and screen the ligands with the reference drug, solasodine, to identify ideal candidate compounds that could inhibit MMP9. The LibDock module was used to analyze compounds that could strongly bind to MMP9, and the top 20 compounds determined by the LibDock score were selected for further research. ADME and TOPKAT modules were used to choose the safe compounds from these 20 compounds. The selected compounds were analyzed using the CDOCKER module for molecular docking and feature mapping for pharmacophore prediction. The stability of these compound–MMP9 complexes was analyzed by molecular dynamic simulation. Cell counting kit-8, colony-forming, and scratch assays were used to analyze the anti-CCRCC effects of these ligands.

Results: Strong binding to MMP9 was exhibited by 6,762 ligands. Among the top 20 compounds, sappanol and sventenin exhibited nearly undefined blood–brain barrier level and lower aqueous solubility, carcinogenicity, and hepatotoxicity than the positive control drug, solasodine. Additionally, these compounds exhibited lower potential energies with MMP9, and the ligand–MMP9 complexes were stable in the natural environment. Furthermore, sappanol inhibited CCRCC cell migration and proliferation.

Conclusion: Sappanol and sventenin are safe and reliable compounds to target and inhibit MMP9. Sappanol can CCRCC cell migration and proliferation. These two compounds may give new thought to the targeted therapy for patients with CCRCC.

## INTRODUCTION

Clear cell renal cell carcinoma (CCRCC) accounts for nearly 75% of kidney cancer, being recognized as the most common subtype of renal cell carcinoma (RCC) [1–3]. According to the global cancer burden, RCC accounted for 1.8% of cancer deaths and 2.2% of new cancer cases worldwide [4]. Although CCRCC occurs in patients aged more than 40 years, it is usually diagnosed by approximately 60 years of age [5]. Surgery is usually the treatment of choice for CCRCC. However, approximately 30% of patients with advanced RCC exhibit postoperative tumor recurrence and metastasis [6]. Therefore, a comprehensive therapy to improve the quality of life and prolong survival is required. Moreover, new targeted drugs to cure CCRCC must be identified.

The enzyme family, matrix metalloproteinases (MMPs), is defined by the Zn^2+^ ion in the catalytic center [7]. The main function of MMPs is the degradation and regulation of extracellular matrix (ECM) proteins [8]. They also liberate bio-active proteins, including cytokines, chemokines, and growth factors [9]. Therefore, MMPs can promote tumor invasion and metastasis. MMPs comprise more than 20 proteases, which are the products of different genes exhibiting slightly different functions [10]. MMP9, one of the human MMPs, belongs to the gelatinase subtype of MMPs, participating in multiple biological processes, including proteolytic ECM degradation, cell–ECM or cell–cell interactions, and extracellular proteins and cell surface cleavage, owing to the extracellular proteolytic cleavage activities [11–17]. Additionally, MMP9 can degrade type IV collagen and destroy the basement membrane, which is related to tumor invasion and metastases [18, 19]. Several studies have exhibited the crucial role of MMP9 in angiogenesis, leading to chronic kidney disease (CKD) [20]. Additionally, some researchers have reported MMP9 overexpression in CCRCC, which might be related to the excessive activation of the MARK/ERK signaling pathway [21]. The MMP9 overexpression in patients with CCRCC is related to poor prognosis, suggesting the use of MMP9 as an ideal target for CCRCC treatment [11, 22]. Therefore, the use of novel MMP9 inhibitors could be an effective therapeutic method for CCRCC.

A natural drug, solasodine, has been reported to inhibit MMP9 and induce cell apoptosis, particularly in human lung cancer [23–26]. However, the pharmacokinetics, safety, and effectiveness of this drug in clinical practice remain unclear. MMP9 targeted drugs have not been used in the clinical setting [27]. Therefore, targeted MMP9 drugs must be screened for treating patients with CCRCC.

The interest in molecular biology is increasing. Purohit et al. identified a SARS-CoV-2 inhibitor through computational approach [28]. Additionally, numerous natural ligands could be used and applied as lead compounds in a clinical setting. These natural ligands have advantages such as decreased toxicity and mild side effects. Thus, the present study attempted to use computational tools to identify natural ligands that can target MMP9 and facilitate the treatment of patients with CCRCC.

## METHODS

### Ligand database and discovery studio 4.5 software

Discovery Studio 4.5 is a user-friendly tool for protein simulation, optimization, and drug design and was used to simulate small molecule and macromolecule systems. The software integrates the storage and management of experimental data with professional-level modeling and simulation tools, providing a platform for cooperation and information sharing among research teams. It also visualizes the data and converts experimental data into a three-dimensional molecular model. This software was used to study protein function and for drug research. A natural product database containing 17931 ligands was downloaded from the ZINC database. The Irwin and Shoichet laboratories, which is in the department of pharmaceutical chemistry at the University of California, San Francisco (UCSF), providing the ZINC database as a free commercial compound database [29].

### Structure-based virtual screening by LibDock

**Figure 1:**
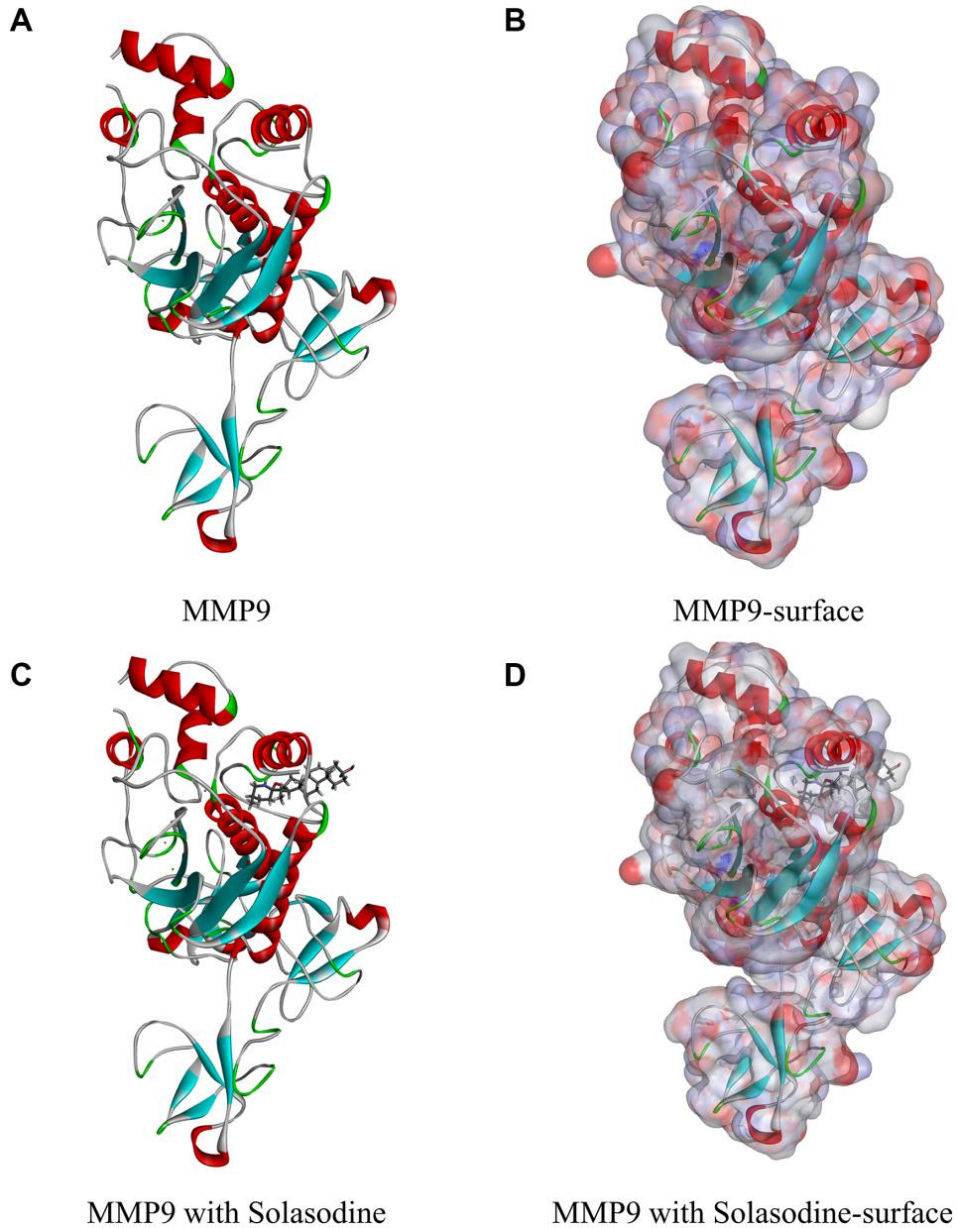
**Molecular structure of MMP9.** (**A**) Initial molecular structure. (**B**) Binding area surface. Blue and red indicate positive and negative charges, respectively. (**C**) Molecular structure of the MMP9–solasodine complex. (**D**) Molecular structure of the MMP9–solasodine complex with surface. Blue and red indicate positive and negative charges, respectively.

**Figure 2:**
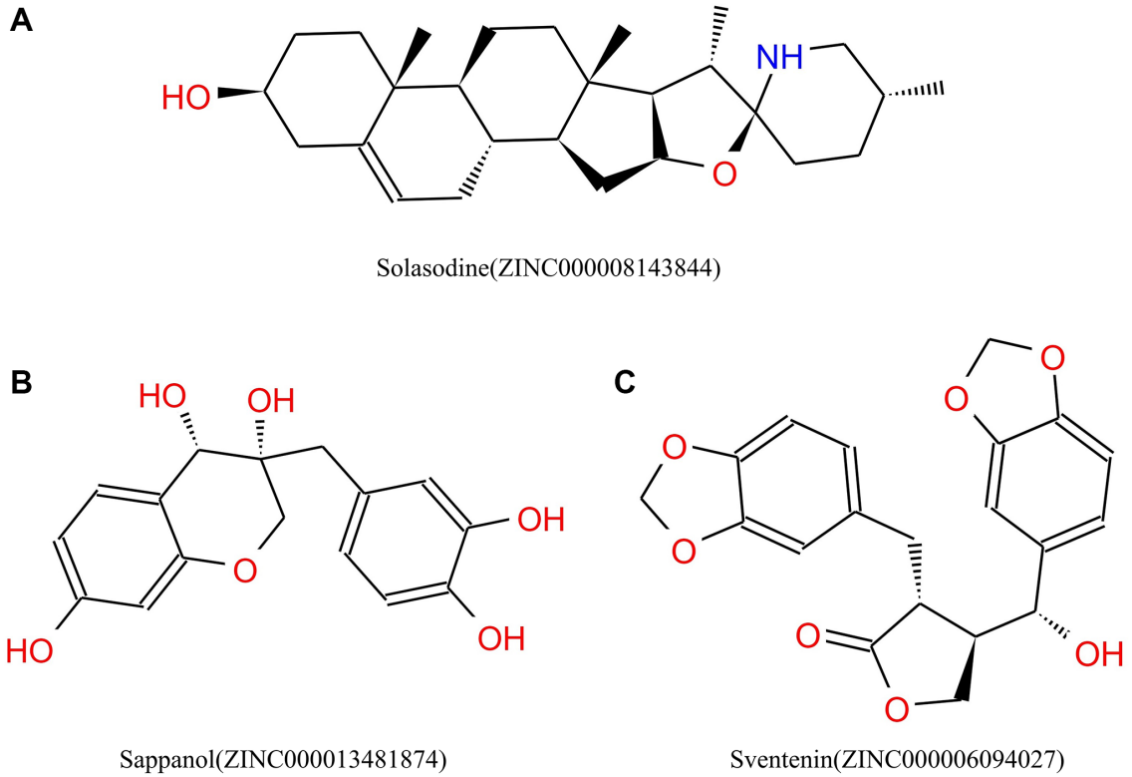
Chemical structures of (**A**) Solasodine (**B**) Sappanol (**C**) Sventenin.

The preliminary screening of the ideal candidate compounds of MMP9 inhibitors was conducted by using the LibDock module of Discovery Studio. The crystal structures of MMP9 (Protein Data Bank identifier: 1L6J) and its inhibitor solasodine (Protein Data Bank identifier: ZINC000008143844) were downloaded from the ZINC database as well as the RCSB protein data bank (Figure 1). The chemical structure of MMP9 is illustrated in Figure 2. Then, MMP9 was imported to the LibDock; the crystal water and other heteroatoms were removed, and protonation, hydrogen energy minimization, and ionization were introduced into the system. The binding site from PDB site records, which were the MMP9 S1’ inhibitor-binding pocket, were chosen [30]. Then, solasodine and the 17931 ligands were input to the LibDock to obtain the LibDock score of the ligands. The LibDock scores of these compounds were ranked and listed [31].

### Prediction of absorption, distribution, metabolism, excretion (ADME), and toxicity

The TOPKAT and ADME modules were applied to calculate ADME and the toxicity of the compounds. The pharmacologic properties and safety were also considered for selecting natural ligands capable of inhibiting MMP9 [32].

### Molecule docking by CDOCKER and ligand pharmacophore prediction

Molecular docking was conducted using the CDOCKER module, on the basis of CHARMM force field, which predicts the results of high-precision docking. Water molecule was removed and hydrogen atom was added onto MMP9 protein, in case the conformation of the receptor–ligand complex was affected by the fixed water molecules [33].

Feature mapping was used to generate pharmacophore models with predictive activity on the basis of a series of compounds with well-defined activity values for specific biological targets. It can analyze hydrophobic, hydrogen bond (HB) acceptor, HB-donor, ring aromatic, and positive ion, a total of five types of attribute elements. The pharmacophore of the two compounds and solasodine was compared.

### Molecular dynamics simulation

The molecule docking program was run for selecting the best binding conformations of the ligand–MMP9 complexes among different poses. Then, they were imported to perform the molecular dynamics simulation, which is one of the most commonly used methods in molecular simulation. Sodium chloride was subsequently added into the system to simulate the physiologic environment. Then, the system was relaxed by minimizing energy in the CHARMM force field. The trajectory protocol of the CDOCKER structural characteristics and potential energy were analyzed using the software.

### Cell lines and reagents

The 786-O cells, a human CCRCC cell line, was cultured in RPMI1640 plus 10% fetal bovine serum (Gibco, Thermo Fisher Scientific, Waltham, Massachusetts, USA) at 37°C under 5% carbon dioxide condition. Solasodine and sappanol were obtained from Wuhan ChemFaces Biochemical Co., Ltd. (Wuhan, China). To obtain the stock solution, the 786-O cells were dissolved in dimethyl sulfoxide. The cell culture medium was configured with different concentrations of 786-O by mixing the acquired stock solution with the appropriate culture medium.

### Cell counting Kit-8 (CCK-8) assay

The human renal clear cell adenocarcinoma cell, 786-O, was assessed using CCK-8 (Dojindo Laboratories, Kumamoto, Japan). The cells were seeded in a 96-well plate for overnight culture until reaching a density of 1.0 × 10^5^ cells/well. Different doses of solasodine and sappanol were added to the cells after washing the culture medium and cultured for 24 h. Then, nine wells were prepared for solasodine and sappanol doses (concentration gradients of 0, 0.4, 0.8, 1.6, 3.2, 6.4, 12.8, 25.6, and 51.2 μmol/L). Cells were then cultured for 1 h after adding CCK-8 at a concentration of 10 μL/well. The OD value of each well was measured at 450 nm wavelength on a microplate reader (Multiskan, Thermo, USA).

### Colony forming assay

The 786-O cells were inoculated in a 6-well cell culture plate (with a surface area of each well as 9.6 cm^2^) until reaching a density of 50 cells/cm^2^. After 24 h, the cultural medium was configured with solasodine and sappanol concentrations of 1.0 and 3.0 mmol/L, respectively. After 10 days, colonies were counted and identified as per a previous study [34]. Additionally, colonies were fixed in 4% paraformaldehyde ad then 30-min dyed using 5% crystal violet.

### *In Vitro* scratch assay

The 786-O cell line was cultured in a 24-well Permanox plate. Consistent cell-free areas were created using 1-mL pipette tips across each well. The loose cells were gently washed out by Dulbecco’s modified Eagle medium. Subsequently, the cells were exposed to various doses of solasodine and sappanol. After culturing for 24 h, different solasodine and sappanol doses were employed for cell treatment at 0, 12, and 24 h. The wound and scratch widths were measured by capturing images for scraped areas through phase contrast microscopy.

### Statistical analysis

Data analysis was conducted using SPSS 18.0 (SPSS Inc., Chicago, Illinois, USA). The quantitative data were analyzed by independent sample *t*-tests. A *P* value less than 0.05 was considered statistically significant.

### RESULTS

### Natural products database virtual screening against MMP9

The natural product database downloaded from the ZINC website comprises 17931 ligands. The MMP9 chemical structure (1L6J) was chosen as the receptor, and the binding site from PDB site records, which were the MMP9 S1’ inhibitor-binding pocket, was chosen [30]. Additionally, the reference drug, solasodine, binds with MMP9 through this binding site. A total of 6,762 ligands were proved to bind firmly with MMP9 (Supplementary Table 1), with Table 1 listing the top 20 ligands. Solasodine (ZINC000008143844) was chosen as the positive control drug.

**Table 1:** LibDock scores of the Top 20 compounds.

**Number**	**Compounds**	**LIBDOCK score**
1	ZINC000001565353	175.657
2	ZINC000014558326	167.389
3	ZINC000001587152	165.616
4	ZINC000091297329	163.192
5	ZINC000018258326	160.061
6	ZINC000033834009	159.355
7	ZINC000004098610	159.116
8	ZINC000006094027	158.445
9	ZINC000004098719	157.044
10	ZINC000005854502	156.570
11	ZINC000004098466	156.333
12	ZINC000013481874	155.842
13	ZINC000004098742	155.799
14	ZINC000014824027	155.782
15	ZINC000021981288	155.756
16	ZINC000003874585	155.264
17	ZINC000001680659	155.003
18	ZINC000015115057	154.595
19	ZINC000000340372	154.464
20	ZINC000012360009	154.338

### Toxicity prediction and ADME

The pharmacological properties of the top 20 compounds and the positive control drug, solasodine, were analyzed by the ADME module of Discovery Studio (Table 2). All compounds, especially ZINC000013481874 and ZINC000001565353, were soluble in water. However, the aqueous-solubility level of solasodine was low. Of the 20 compounds 6 compounds exhibited undefined blood–brain barrier (BBB) level, whereas three compounds exhibited a low BBB level. On the contrary, the BBB level of solasodine was high. According to the cytochrome P450 2D6 (CYP2D6) inhibition, the vast majority of the 20 compounds and solasodine, except ZINC000015115057 and ZINC000000340372, were predicted to be CYP2D6 inhibitors. Of the 20 compounds, 11 compounds and solasodine were hepatotoxic; however, the others were not. Most of the compounds and solasodine exhibited superior absorption levels. The exceptions included ZINC000001565353 and ZINC000033834009 exhibiting a very poor level, ZINC000004098610 exhibiting a poor level, and ZINC000004098466 exhibiting a moderate level. The prediction of plasma protein binding levels exhibited that seven compounds could be strong absorbents, and the other 13 compounds, which exhibited weak absorption, were similar to solasodine.

**Table 2:** Compound properties of adsorption, distribution, metabolism, and excretion.

**Number**	**Compounds**	**Solubility Level**	**BBB Level**	**CYP2D6**	**Hepatotoxicity**	**Absorption Level**	**PPB Level**
1	ZINC000015115057	2	1	0	0	0	0
2	ZINC000005854502	3	2	1	0	0	1
3	ZINC000004098466	2	4	1	1	1	0
4	ZINC000014558326	2	2	1	1	0	0
5	ZINC000033834009	3	4	1	0	3	1
6	ZINC000000340372	2	1	0	1	0	0
7	ZINC000021981288	2	3	1	1	0	0
8	ZINC000003874585	3	2	1	1	0	0
9	ZINC000013481874	4	4	1	0	0	1
10	ZINC000012360009	2	1	1	1	0	0
11	ZINC000006094027	3	3	1	0	0	0
12	ZINC000001680659	2	1	1	1	0	0
13	ZINC000004098719	2	1	1	0	0	0
14	ZINC000001587152	3	3	1	1	0	1
15	ZINC000004098742	2	1	1	0	0	0
16	ZINC000001565353	4	4	1	1	3	1
17	ZINC000091297329	3	4	1	1	0	0
18	ZINC000004098610	3	4	1	0	2	1
19	ZINC000018258326	2	2	1	0	0	0
20	ZINC000014824027	3	2	1	1	0	1
21	Solasodine	1	1	1	1	0	0

Abbreviations: BBB: blood-brain barrier; CYP2D6: cytochrome P-450 2D6; PPB: plasma protein binding. Aqueous-solubility level: 0, extremely low; 1, very low, but possible; 2, low; 3, good. BBB level: 0, very high penetrant; 1, high; 2, medium; 3, low; 4, undefined. CYP2D6 level: 0, noninhibitor; 1, inhibitor. Hepatotoxicity: 0, nontoxic; 1, toxic. Human-intestinal absorption level: 0, good; 1, moderate; 2, poor; 3, very poor. PPB: 0, absorbent weak; 1, absorbent strong.

Safety must be considered during drug screening. The TOPKAT module of the Discovery Studio that can quickly calculate and predict the toxicity and environmental effects of these compounds, including Ames mutagenicity (Ames test), developmental toxicity potential (DTP), and rodent carcinogenicity, was used to analyze the safety of these 20 compounds and solasodine (Table 3). Of the 20 compounds, 15 compounds and solasodine were predicted to be non-mutagenic. Additionally, 11 ligands in female mouse, 9 compounds in male mouse, 6 compounds in female rat, and 12 compounds in male rat were noncarcinogenic. Solasodine was demonstrated to be a carcinogen in female mouse. Only five compounds were predicted to be nontoxic in DTP, whereas others and solasodine were demonstrated to be toxic. Thus, ZINC000013481874 and ZINC000006094027 were considered to be the two safe lead candidate compounds for further studies (Figure 2).

**Table 3:** Compound toxicities.

**Number**	**Compounds**	**Mouse NTP**		**Rat NTP**		**Ames**	**DTP**
		**Female**	**Male**	**Female**	**Male**		
1	ZINC000015115057	0.917	1	0.017	0.001	1	0.999
2	ZINC000005854502	1	1	1	1	0	1
3	ZINC000004098466	0.09	1	1	1	1	1
4	ZINC000014558326	0	1	1	1	0	0.14
5	ZINC000033834009	0.964	1	0	0.97	0.091	1
6	ZINC000000340372	0	0.999	1	0.001	0	1
7	ZINC000021981288	0.002	0	0	0.09	0	1
8	ZINC000003874585	0	0	1	0.004	0	0.001
9	ZINC000013481874	0	0	1	0.005	0	0.999
10	ZINC000012360009	0.292	0	1	0	0.997	0.02
11	ZINC000006094027	0.026	0	0	0	0.004	1
12	ZINC000001680659	0.292	0	1	0	0.997	0.02
13	ZINC000004098719	0	0.025	1	1	0	1
14	ZINC000001587152	1	0.881	0.971	0.035	0	0.999
15	ZINC000004098742	0	0.002	1	1	0	0.828
16	ZINC000001565353	0.963	1	0.087	1	0.81	1
17	ZINC000091297329	0.314	0	0.065	0.016	0.001	0.151
18	ZINC000004098610	0.985	1	1	0.999	0.001	1
19	ZINC000018258326	0.868	1	1	0	0	0.888
20	ZINC000014824027	1	0.862	0.877	0.025	0	0.999
21	Solasodine	1	0	0	0	0.038	1

Abbreviations: NTP: U.S. National Toxicology Program; DTP: developmental toxicity potential. NTP <0.3 (noncarcinogen); >0.8 (carcinogen). Ames <0.3 (nonmutagen); >0.8 (mutagen). DTP <0.3 (nontoxic); >0.8 (toxic).

### Ligand binding and pharmacophore analyses

CDOCKER, which is based on the CHARMM force field, was employed to study the mechanisms of binding of these ligands with MMP9. ZINC000013481874 is also called sappanol, whereas ZINC000006094027 is also called sventenin. Sappanol, sventenin, and solasodine were imported and bonded with MMP9 in the CDOCKER module; the potential energies are listed in Table 4. The CDOCKER energies of sappanol (−50.817 kcal/mol) and sventenin (−51.7422 kcal/mol) were lower than that of solasodine (−23.1805 kcal/mol), which proved that ZINC000013481874 and ZINC000006094027 could bind more firmly with MMP9 than solasodine.

**Table 4:** CDOCKER potential energy of different compounds with MMP9.

**Compounds**	**-CDOCKER Potential Energy (kcal/mol)**
ZINC000006094027	51.7422
ZINC000013481874	50.817
ZINC000008143844	23.1805

**Figure 3:**
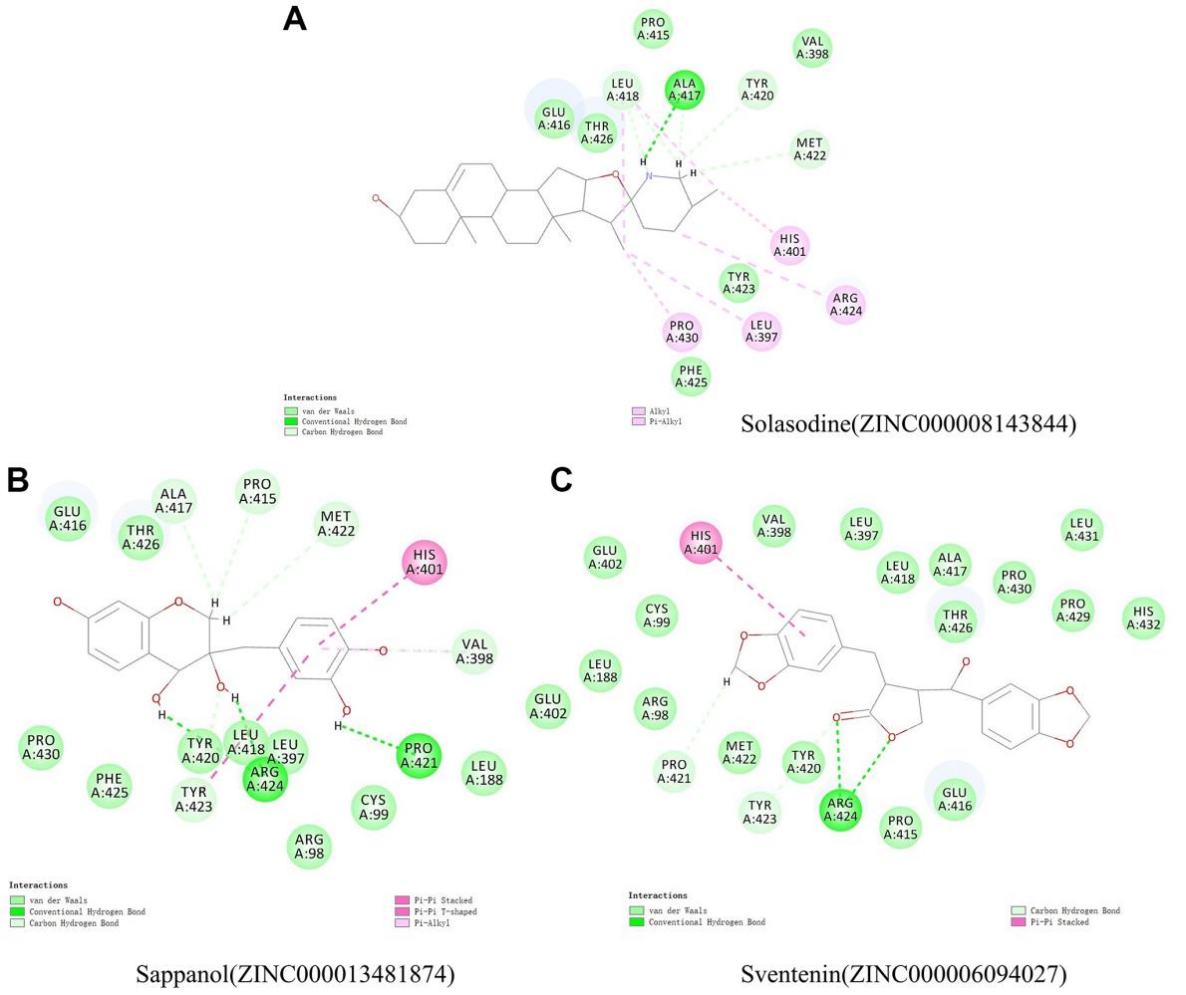
Schematic illustration for intermolecular interaction of the predicted binding modes between MMP9 and (**A**) Solasodine, (**B**) Sappanol, and (**C**) Sventenin.

**Figure 4:**
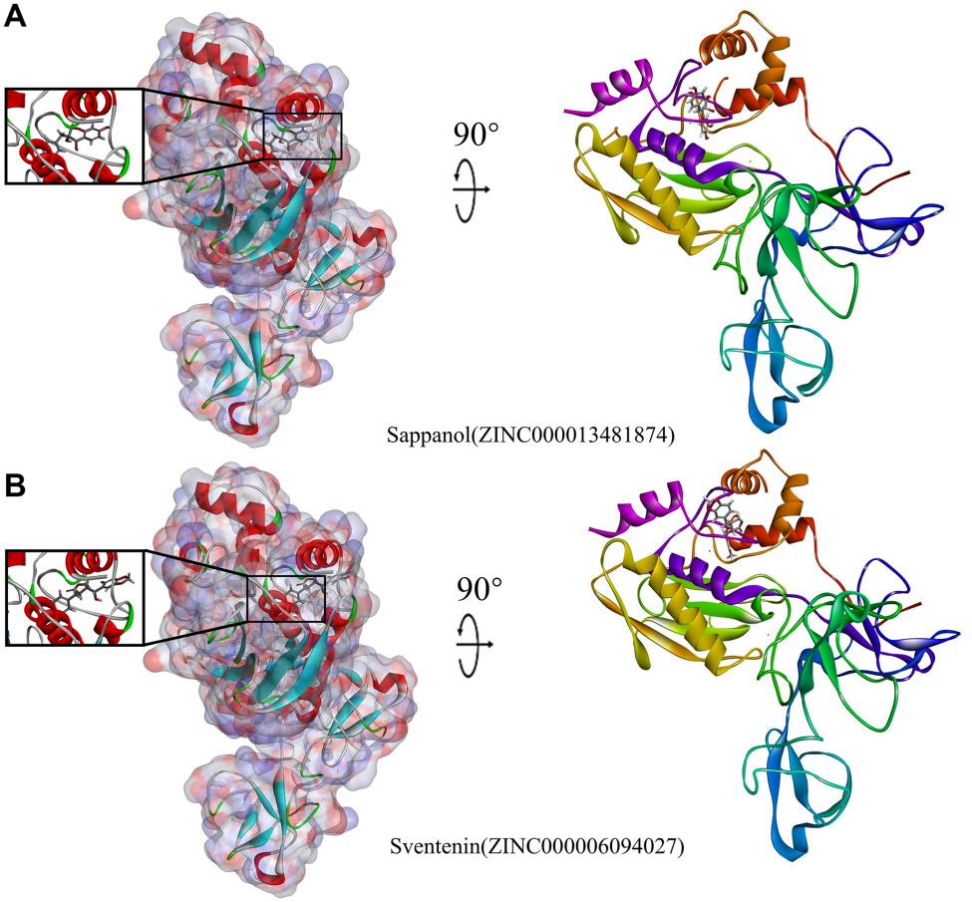
**Schematic illustration for MMP9-ligand interactions, showing the surface of the binding areas.** Blue and red indicate positive and negative charges, respectively; ligands are shown as sticks, with structures surrounding the ligand-receptor junction displayed as thinner sticks. (**A**) Sappanol-MMP9 complex. (**B**) Sventenin-MMP9 complex.

The structural computation study also exhibited the HBs and π-related interactions between these ligands and MMP9 (Figures 3 and 4). We found that sappanol formed 8 pairs of HBs with MMP9, whereas ZINC000006094027 formed 4 pairs of hydrogen bonds with MMP9, including ARG424:HN and TYR423: HA of the ligand with O1 of the MMP9, ARG424:HH11 of the ligand with O3 of the MMP9, and PRO421:O of the ligand with H43 of the MMP9 (Table 5). Additionally, sventenin demonstrated π-related interactions with HIS401 of the MMP9. It also demonstrated π-related interactions with HIS401, TYR423, and VAL398 of MMP9 (Table 6). Additionally, solasodine formed 6 pairs of π-related interactions and 6 pairs of hydrogen bonds with MMP9.

**Table 5:** Hydrogen bond Interaction parameters of different compounds with MMP9.

**Receptor**	**Compound**	**Donor Atom**	**Receptor Atom**	**Distances (Å)**
MMP9	ZINC000013481874	ZINC000013481874:H29	ARG424:O	2.16
		ZINC000013481874:H35	PRO421:O	1.85
		ZINC000013481874:H38	ARG424:O	2.52
		VAL398:HA	ZINC000013481874:O17	2.3
		TYR423:HA	ZINC000013481874:O11	2.86
		ZINC000013481874:H27	PRO415:O	2.85
		ZINC000013481874:H27	ALA417:O	2.49
		ZINC000013481874:H28	MET422:O	3.07
	ZINC000006094027	ARG424:HN	ZINC000006094027:O1	2.36
		ARG424:HH11	ZINC000006094027:O3	2.59
		TYR423:HA	ZINC000006094027:O1	3.07
		ZINC000006094027:H43	PRO421:O	2.95
	Solasodine	Molecule:H73	ALA417:O	1.82
		LEU418:HA	Molecule:N30	2.7
		Molecule:H71	ALA417:O	3.05
		Molecule:H71	LEU418:O	2.99
		Molecule:H71	TYR420:O	2.78
		Molecule:H72	MET422:O	2.97

**Table 6:** π-Related interaction parameters of different compounds with MMP9.

**Receptor**	**Compound**	**Donor Atom**	**Receptor Atom**	**Distances (Å)**
MMP9	ZINC000013481874	HIS401	ZINC000013481874	4.05
		TYR423	ZINC000013481874	5.76
		ZINC000013481874	VAL398	5.45
	ZINC000006094027	HIS401	ZINC000006094027	4.92
	Solasodine	Molecule:C1	LEU397	5.35
		Molecule:C1	LEU418	3.97
		Molecule:C1	PRO430	4.23
		Molecule:C26	ARG424	4.94
		Molecule:C28	LEU418	5.01
		HIS401	Molecule:C28	5.08

**Figure 5:**
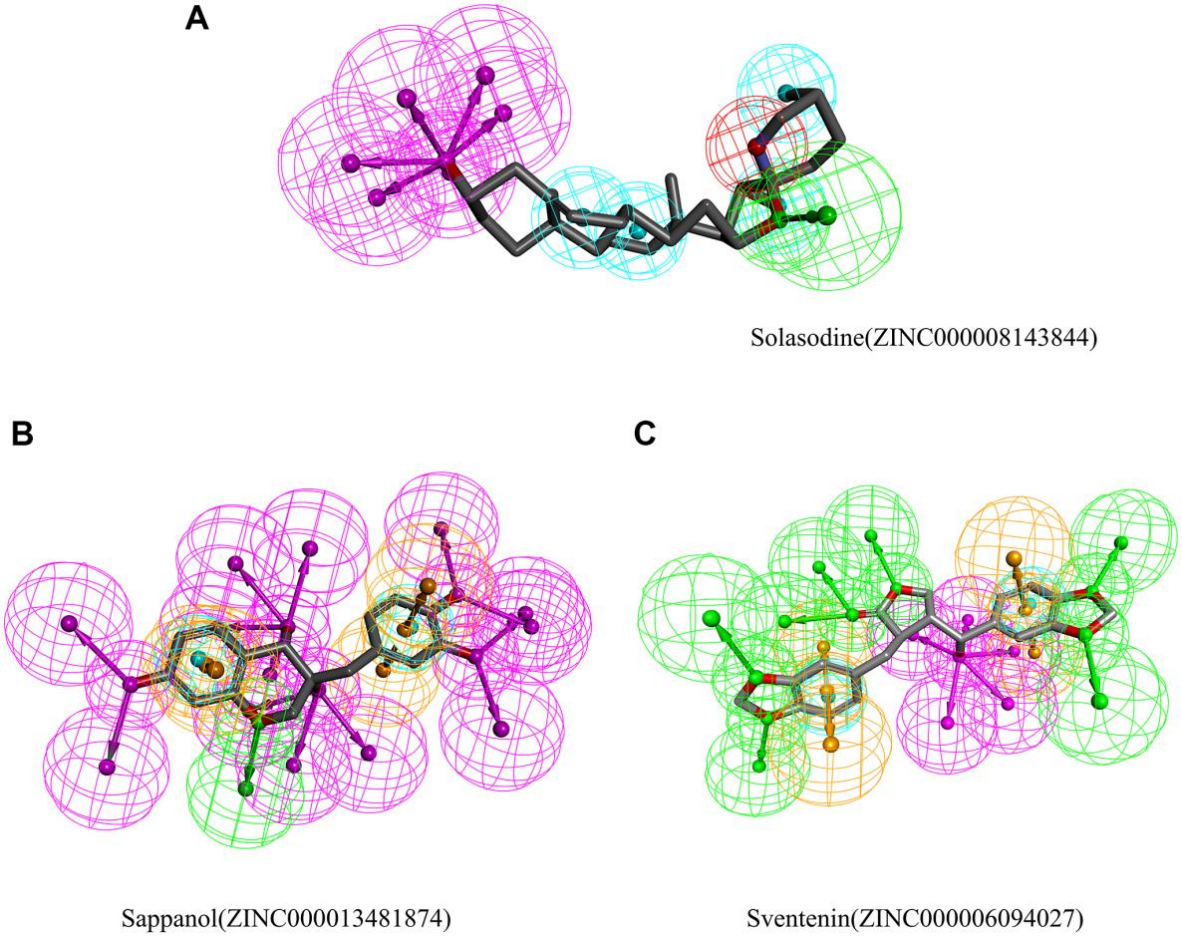
Results of the pharmacophore for (**A**) Solasodine (**B**) Sappanol (**C**) Sventenin.

Then, the pharmacophores of these two candidate ligands and solasodine were calculated. The results exhibited 33 features in sappanol including 14 HB acceptors, 13 HB-donors, 2 hydrophobics, and four ring aromatics (Figure 5). Additionally, 23 features in sventenin, including 12 HB-acceptors, 5 HB-donors, 2 hydrophobics, and four ring aromatics were observed. Only 18 features, including 7 HB-acceptor, 6 HB-donors, 4 hydrophobics, and one positive ion, were observed in solasodine.

### Molecular dynamics simulation

**Figure 6:**
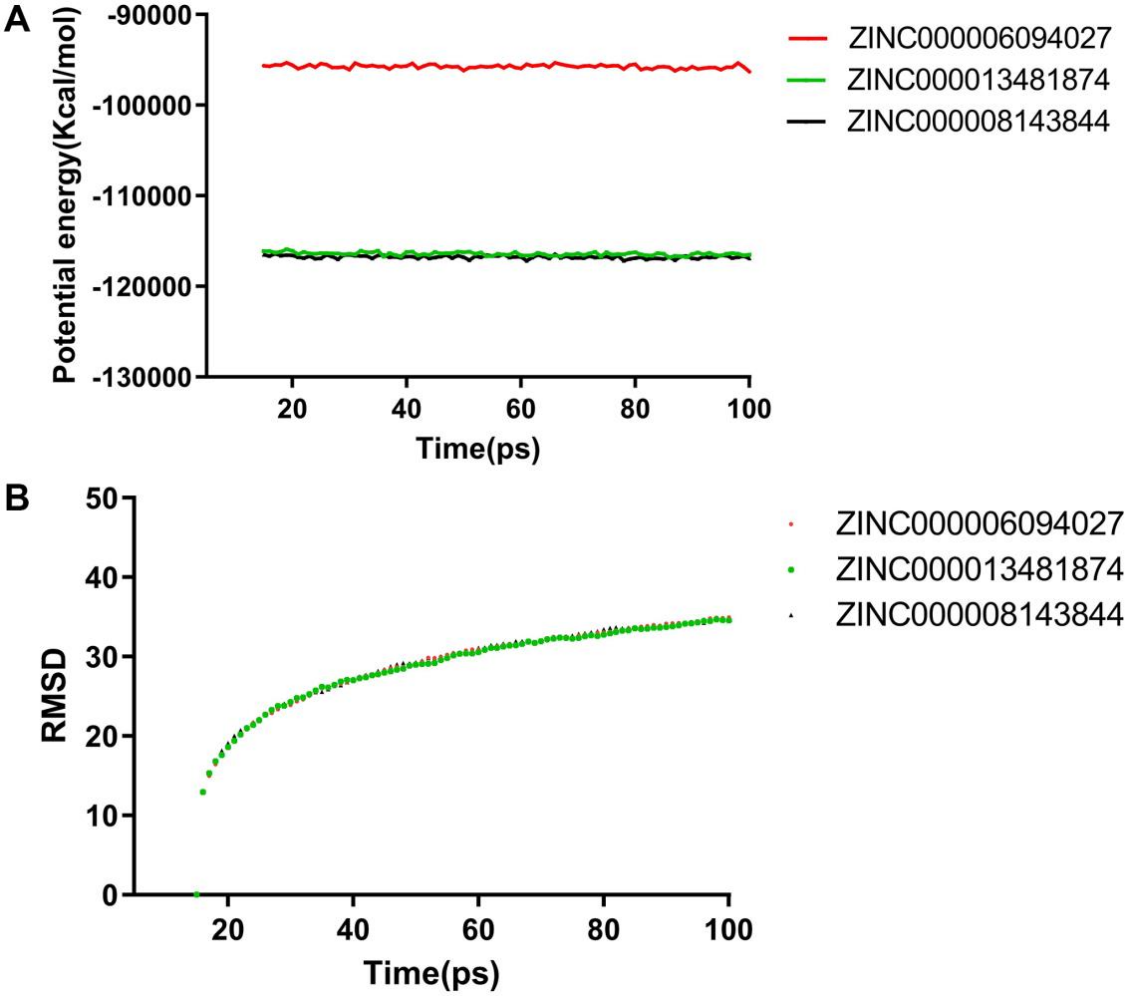
**Molecular dynamics simulation results for sappanol and sventenin.** (**A**) Potential energy and average backbone root-mean-square deviation. (**B**) Root-mean-square deviation (RMSD).

In addition to safety, stability should be another vital thing and be fully considered during drug screening. The molecular dynamics simulation module of the software analyzed the stability of these compound–MMP9 complexes in the natural environment. The result of the CDOCKER was used to run the molecular docking experiment and to obtain the CDOCKER potential energy and RMSD curves of these compound–MMP9 complexes (Figure 6). Their CDOCKER potential energy gradually became stabilized with time going by. The π-related interactions and hydrogen bonds between the compounds and the MMP9 are beneficial for the stability of these complexes. Therefore, solasodine, sappanol and sventenin could bind with MMP9, and the ligand–MMP9 complexes could be stable under natural circumstances.

### Sappanol reduced 786-O cell proliferation

**Figure 7:**
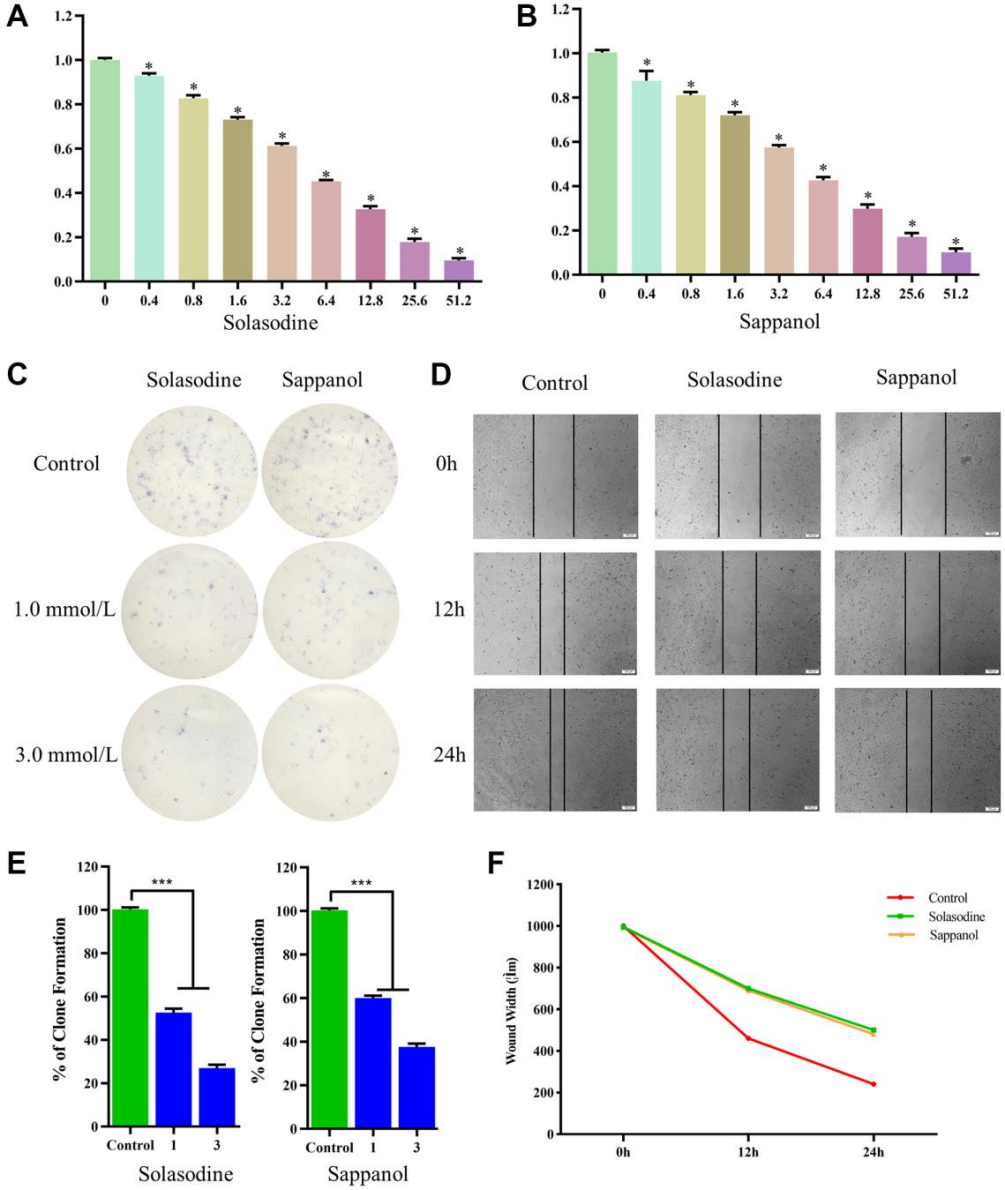
The 786-O cell viability following (**A**) solasodine and (**B**) sappanol treatments. (**C**) Clonogenicity in Petri dishes with various doses of solasodine and sappanol. (**D**) Scratch assay in control, solasodine, and sappanol groups. (**E**) The number of clones formed in the 786-O cell lines. (**F**) Wound width in control, solasodine, and sappanol groups.

The CCK-8 assay was used to calculate and compare survival of cells after sappanol and solasodine treatments. The viability of 786-O cells declined significantly after drug concentration augmentation (Figure 7A and 7B). Additionally, the descent rates of solasodine and sappanol were roughly similar, illustrating that sappanol had similar inhibitory effects on 786-O cells.

The colony-forming assay results demonstrated low clonogenicities in petri dish treated with solasodine and sappanol, in comparison with the control (Figure 7C). The clonogenicities of 786-O cells in the 1.0 mmol/L solasodine and 3.0 mmol/L doses were similar to those in the sappanol doses. The percentages of clone formation following drug treatments were significantly reduced, in comparison with the control (Figure 7E).

### Sappanol inhibited 786-O cell migration

The *in vitro* scratch assay was conducted to evaluate 786-O cell migration. The widths of the cell-free areas were measured 12 and 24 h after the scratch. The widths of the scratched area in solasodine and sappanol groups were significantly smaller than those in controls (*P* < 0.05) (Figure 7D and 7F).

## DISCUSSION

CCRCC is insensitive to chemotherapy and radiation [35]. The life quality and survival of CCRCC patients are dependent on the genomic landscape of the tumor [36]. Targeted therapy has gained interest, with several scientists researching about developing targeted drugs [37, 38]. Additionally, some studies have reported that the protein and mRNA levels of MMP9 in CCRCC were higher than those in normal tissues. Moreover, a high MMP9 level was correlated with poor prognosis in CCRCC patients [21]. On the one hand, MMP9 facilitates tumor migration and angiogenesis by promoting ECM degradation [8, 39, 40]. On the other hand, MMP9 can activate the mitogen-activated protein kinase (MARK)/ERK and TGF-β/SMAD signaling pathways to further promote tumor metastasis [21]. Therefore, MMP9 targeted drugs must be identified for treating patients with CCRCC.

The present study mainly applied Discovery Studio 4.5 in performing computational experiments and screen candidate ligands. Initially, 17931 ligands were downloaded as the ligand database from the ZINC website, and the LibDock module of this software was used to preliminarily screen the ligands that can combine with MMP9. The results exhibited that 6,762 ligands could firmly combine with the MMP9 crystal structural. The top 20 ligands were chosen on the basis of the LibDock score for further research.

The ADME and TOPKAT results exhibited that two natural ligands, ZINC000013481874 and ZINC000006094027, were safer than solasodine. For example, both ZINC000013481874 and ZINC000006094027 exhibited low aqueous-solubility levels and nearly undefined BBB levels. Additionally, they were both predicted to be non-hepatotoxic, whereas solasodine exhibited hepatotoxicity. The TOPKAT result exhibited that ZINC000006094027 had lower carcinogenicity than solasodine in female mice. ZINC000013481874 was predicted to be similar to solasodine in TOPKAT aspect. Thus, ZINC000013481874 and ZINC000006094027 were chosen to be candidate nontoxic compounds with better aqueous-solubility levels, lower BBB levels, better intestinal absorption levels, and lesser carcinogenicity than solasodine. Except these two ligands, the other 18 ligands exhibited some disadvantages. However, they still may be useful in developing other drugs.

The CDOCKER module was employed to verify that sappanol and sventenin could bind with MMP9. Additionally, the CDOCKER potential energy of these two candidate ligands and solasodine was analyzed. The results suggested that sappanol and sventenin had lower potential energy than solasodine. We also compared the hydrogen bonds, π-related interactions, and pharmacophore of these two compounds and solasodine. The results indicated that sappanol and sventenin exhibited a higher binding force with MMP9 than solasodine.

Molecular dynamics simulation was employed to verify the stability of the compound–MMP9 complexes by running the RMSD and calculating potential energy. Calculations exhibited that the trajectories of both sappanol and sventenin reached their equilibrium and stabilized with time, indicating that the three ligand–MMP9 complexes could become stable in a short time period under natural circumstances. Therefore, sappanol and sventenin could be regarded as the ideal natural ligands for MMP9 inhibitor drug development and may be used for the treatment of patients with CCRCC.

Because a suitable sventenin medicine could not be obtained, we purchased sappanol for further studies. CCK-8, colony-forming, and *in vitro* scratch assays were used to evaluate the anti-CCRCC effects of sappanol by comparing them with the reference drug, solasodine. In CCK-8 and colony-forming assays, sappanol and solasodine reduced the survival of cells and the clonogenicities of the 786-O cell line compared with those of controls. Additionally, the augmentation of drug concentrations significantly reduced the proliferation of 786-O cells. The widths of the scratched area in controls decreased sharply, whereas those in solasodine and sappanol groups were higher than those in controls. The effects of sappanol and solasodine increased with an increase in the dose. Sappanol exhibited similar effects on solasodine, which proved that sappanol is an ideal lead ligand that can reduce proliferation and inhibit migration of CCRCC cells.

Targeted therapies have been widely used for treating patients with cancer. However, no suitable drug is available to treat patients with CCRCC. The present study used computational tools to identify ideal candidate natural ligands, which was the first step in drug designation to treat patients with CCRCC.

## CONCLUSION

The LibDock result proved that 6,762 ligands could bind firmly with MMP9. The ADME and TOPKAT results illustrated that sappanol and sventenin were safer candidate ligands than the reference drug, solasodine. The CDOCKER results and feature mapping results exhibited that these two compounds had lower binding potential energies than solasodine, indicating that they can bind firmly with MMP9. The molecular dynamics simulation proved that the ligand–MMP9 complexes could exist stably in the natural environment. CCK-8, colony-forming, and *in vitro* scratch assays proved that sappanol could reduce proliferation and inhibit CCRCC cell migration. Thus, sappanol could be the perfect ligand for treating patients with CCRCC.

## Supplementary Materials

Supplementary Table 1
