# Supplementary material for: Computational study of effective matrix metalloproteinase 9 (MMP9) targeting natural inhibitors
Source: Aging (Albany NY). 2021 Oct 4;13(19):22867–82. doi: 10.18632/aging.203581 (PMC8544340; doi:10.18632/aging.203581)
Supplement: Supplementary Table 1 [file aging-13-203581-s001.docx]

**Supplementary Table 1. Compounds capable of binding with MMP9, and their LibDock scores.**

| **Number** | **Compounds** | **Libdock score** | **Number** | **Compounds** | **Libdock score** |
| --- | --- | --- | --- | --- | --- |
| 1 | ZINC000004706573 | 75.8512 | 3364 | ZINC000005514849 | 80.8492 |
| 2 | ZINC000004742844 | 90.5589 | 3365 | ZINC000005574966 | 108.124 |
| 3 | ZINC000004027288 | 93.1124 | 3366 | ZINC000005647278 | 82.7794 |
| 4 | ZINC000000388776 | 117.039 | 3367 | ZINC000001680757 | 108.736 |
| 5 | ZINC000004532782 | 94.741 | 3368 | ZINC000001692446 | 55.7824 |
| 6 | ZINC000005963547 | 142.887 | 3369 | ZINC000001699438 | 81.4693 |
| 7 | ZINC000014726530 | 124.33 | 3370 | ZINC000001701826 | 80.9382 |
| 8 | ZINC000012402601 | 126.809 | 3371 | ZINC000001722340 | 56.0701 |
| 9 | ZINC000012358697 | 73.3252 | 3372 | ZINC000001732441 | 96.5103 |
| 10 | ZINC000012493443 | 122.247 | 3373 | ZINC000001757816 | 50.1909 |
| 11 | ZINC000012358753 | 125.216 | 3374 | ZINC000001841229 | 72.4213 |
| 12 | ZINC000012358842 | 83.4739 | 3375 | ZINC000001848437 | 94.3428 |
| 13 | ZINC000012958660 | 57.2249 | 3376 | ZINC000001849842 | 88.5422 |
| 14 | ZINC000000164902 | 84.033 | 3377 | ZINC000001849951 | 88.7622 |
| 15 | ZINC000004097087 | 150.348 | 3378 | ZINC000004995653 | 74.9523 |
| 16 | ZINC000000388770 | 72.2271 | 3379 | ZINC000005053120 | 97.9035 |
| 17 | ZINC000000393698 | 104.425 | 3380 | ZINC000005115783 | 111.645 |
| 18 | ZINC000000391936 | 98.3865 | 3381 | ZINC000005131770 | 76.8995 |
| 19 | ZINC000000391197 | 95.8325 | 3382 | ZINC000005132207 | 117.013 |
| 20 | ZINC000000391197 | 89.6976 | 3383 | ZINC000005132732 | 77.5652 |
| 21 | ZINC000000388747 | 80.7653 | 3384 | ZINC000005132840 | 62.7098 |
| 22 | ZINC000100776199 | 61.3297 | 3385 | ZINC000005134370 | 46.4393 |
| 23 | ZINC000005159263 | 95.9816 | 3386 | ZINC000005138397 | 71.3273 |
| 24 | ZINC000049538670 | 89.1928 | 3387 | ZINC000005158937 | 135.813 |
| 25 | ZINC000004096677 | 114.295 | 3388 | ZINC000005159440 | 64.847 |
| 26 | ZINC000100372229 | 64.9908 | 3389 | ZINC000005159529 | 92.4252 |
| 27 | ZINC000049538669 | 93.1313 | 3390 | ZINC000005191517 | 94.4809 |
| 28 | ZINC000013829483 | 130.956 | 3391 | ZINC000005191791 | 98.8608 |
| 29 | ZINC000015115057 | 154.595 | 3392 | ZINC000005195091 | 123.827 |
| 30 | ZINC000031500056 | 113.526 | 3393 | ZINC000001683675 | 83.4167 |
| 31 | ZINC000031500056 | 119.092 | 3394 | ZINC000001690407 | 63.9575 |
| 32 | ZINC000004261992 | 74.3248 | 3395 | ZINC000001693578 | 71.2056 |
| 33 | ZINC000004261993 | 72.1747 | 3396 | ZINC000001699551 | 65.162 |
| 34 | ZINC000032154765 | 54.0389 | 3397 | ZINC000001718840 | 91.899 |
| 35 | ZINC000032211383 | 71.8619 | 3398 | ZINC000001765432 | 85.4408 |
| 36 | ZINC000059724990 | 79.4272 | 3399 | ZINC000001842389 | 100.786 |
| 37 | ZINC000005954694 | 81.0964 | 3400 | ZINC000001845884 | 62.1904 |
| 38 | ZINC000004271722 | 77.5126 | 3401 | ZINC000001849637 | 92.7954 |
| 39 | ZINC000059868199 | 69.4694 | 3402 | ZINC000001849853 | 98.4489 |
| 40 | ZINC000031500048 | 117.809 | 3403 | ZINC000001850009 | 65.7331 |
| 41 | ZINC000031500048 | 124.989 | 3404 | ZINC000004474604 | 111.517 |
| 42 | ZINC000002101359 | 102.706 | 3405 | ZINC000004501355 | 50.7054 |
| 43 | ZINC000032150628 | 61.9465 | 3406 | ZINC000004501357 | 53.9211 |
| 44 | ZINC000060274334 | 39.1523 | 3407 | ZINC000004521350 | 90.6447 |
| 45 | ZINC000032211379 | 75.3659 | 3408 | ZINC000004521597 | 89.9326 |
| 46 | ZINC000059727266 | 86.882 | 3409 | ZINC000004521745 | 79.6037 |
| 47 | ZINC000059558639 | 67.486 | 3410 | ZINC000004538403 | 88.415 |
| 48 | ZINC000001849758 | 112.543 | 3411 | ZINC000004753654 | 73.7705 |
| 49 | ZINC000001634346 | 112.684 | 3412 | ZINC000004791946 | 99.4805 |
| 50 | ZINC000001531391 | 127.007 | 3413 | ZINC000004802621 | 57.9105 |
| 51 | ZINC000001737786 | 87.4976 | 3414 | ZINC000004802645 | 50.9632 |
| 52 | ZINC000001699558 | 66.4897 | 3415 | ZINC000004802659 | 52.715 |
| 53 | ZINC000001699969 | 79.935 | 3416 | ZINC000004803379 | 110.072 |
| 54 | ZINC000001704768 | 97.6918 | 3417 | ZINC000004826818 | 77.1577 |
| 55 | ZINC000000336205 | 89.0973 | 3418 | ZINC000004995650 | 75.2126 |
| 56 | ZINC000000336939 | 74.9443 | 3419 | ZINC000001680828 | 85.6023 |
| 57 | ZINC000000898356 | 140.859 | 3420 | ZINC000001690027 | 74.2798 |
| 58 | ZINC000000899804 | 98.783 | 3421 | ZINC000001693339 | 63.7509 |
| 59 | ZINC000000899909 | 137.182 | 3422 | ZINC000001699439 | 72.811 |
| 60 | ZINC000001572827 | 66.5618 | 3423 | ZINC000001718570 | 89.3231 |
| 61 | ZINC000001692348 | 101.373 | 3424 | ZINC000001733761 | 55.055 |
| 62 | ZINC000001850660 | 84.1062 | 3425 | ZINC000001849636 | 92.9166 |
| 63 | ZINC000001850774 | 120.7 | 3426 | ZINC000001849844 | 87.9455 |
| 64 | ZINC000001850913 | 94.3872 | 3427 | ZINC000001849954 | 90.7952 |
| 65 | ZINC000002011323 | 61.8381 | 3428 | ZINC000004098388 | 86.603 |
| 66 | ZINC000002037477 | 81.0693 | 3429 | ZINC000004098632 | 134.42 |
| 67 | ZINC000002083321 | 83.7539 | 3430 | ZINC000004098719 | 157.043 |
| 68 | ZINC000002545304 | 85.7641 | 3431 | ZINC000004098911 | 66.1041 |
| 69 | ZINC000000895845 | 77.0068 | 3432 | ZINC000004100760 | 108.019 |
| 70 | ZINC000000895846 | 82.068 | 3433 | ZINC000004141385 | 85.3382 |
| 71 | ZINC000000899886 | 124.641 | 3434 | ZINC000004234841 | 93.5504 |
| 72 | ZINC000000900156 | 97.5386 | 3435 | ZINC000004234861 | 93.6255 |
| 73 | ZINC000001228645 | 110.483 | 3436 | ZINC000004262098 | 70.6195 |
| 74 | ZINC000001850433 | 61.8115 | 3437 | ZINC000004262495 | 53.4458 |
| 75 | ZINC000001850773 | 121.457 | 3438 | ZINC000004262497 | 56.4477 |
| 76 | ZINC000001850914 | 93.255 | 3439 | ZINC000004271680 | 72.2927 |
| 77 | ZINC000002003738 | 90.9628 | 3440 | ZINC000004283567 | 97.4998 |
| 78 | ZINC000002018831 | 106.989 | 3441 | ZINC000004284428 | 90.3716 |
| 79 | ZINC000002565013 | 113.849 | 3442 | ZINC000004284430 | 86.9646 |
| 80 | ZINC000002566914 | 103.156 | 3443 | ZINC000001683870 | 80.119 |
| 81 | ZINC000001693678 | 96.2991 | 3444 | ZINC000001687335 | 92.7388 |
| 82 | ZINC000001708730 | 103.3 | 3445 | ZINC000001691362 | 49.6666 |
| 83 | ZINC000001850799 | 86.5033 | 3446 | ZINC000001693581 | 79.3444 |
| 84 | ZINC000001532247 | 80.8969 | 3447 | ZINC000001696686 | 55.4523 |
| 85 | ZINC000013513935 | 116.307 | 3448 | ZINC000001699889 | 84.8352 |
| 86 | ZINC000013513935 | 117.662 | 3449 | ZINC000001705987 | 84.847 |
| 87 | ZINC000005510222 | 112.66 | 3450 | ZINC000001747831 | 86.7954 |
| 88 | ZINC000012495246 | 105.158 | 3451 | ZINC000001842391 | 96.9835 |
| 89 | ZINC000012496482 | 114.991 | 3452 | ZINC000001845895 | 57.2265 |
| 90 | ZINC000013373002 | 97.6813 | 3453 | ZINC000001849647 | 110.079 |
| 91 | ZINC000013460181 | 101.423 | 3454 | ZINC000001849896 | 106.114 |
| 92 | ZINC000014410370 | 134.146 | 3455 | ZINC000003861378 | 54.3717 |
| 93 | ZINC000014449640 | 94.4947 | 3456 | ZINC000003861605 | 58.7685 |
| 94 | ZINC000012495252 | 101.194 | 3457 | ZINC000003872070 | 126.167 |
| 95 | ZINC000013301807 | 128.531 | 3458 | ZINC000003872070 | 126.494 |
| 96 | ZINC000013302910 | 114.901 | 3459 | ZINC000003874616 | 89.6538 |
| 97 | ZINC000013359969 | 132.11 | 3460 | ZINC000003880802 | 57.6782 |
| 98 | ZINC000013359969 | 129.653 | 3461 | ZINC000003947504 | 153.539 |
| 99 | ZINC000013373003 | 91.9568 | 3462 | ZINC000003979039 | 116.213 |
| 100 | ZINC000013377494 | 110.635 | 3463 | ZINC000003984017 | 100.944 |
| 101 | ZINC000014452127 | 107.83 | 3464 | ZINC000004023242 | 102.163 |
| 102 | ZINC000014452584 | 107.717 | 3465 | ZINC000004023310 | 69.2587 |
| 103 | ZINC000014593989 | 110.097 | 3466 | ZINC000004024376 | 75.5086 |
| 104 | ZINC000014758886 | 118.741 | 3467 | ZINC000004095723 | 70.0255 |
| 105 | ZINC000014758887 | 93.4613 | 3468 | ZINC000004095906 | 76.3416 |
| 106 | ZINC000014813431 | 105.574 | 3469 | ZINC000001596545 | 73.4107 |
| 107 | ZINC000015207084 | 104.606 | 3470 | ZINC000001597265 | 96.9066 |
| 108 | ZINC000016343182 | 120.718 | 3471 | ZINC000001597267 | 90.2633 |
| 109 | ZINC000016343185 | 118.54 | 3472 | ZINC000001597281 | 87.8391 |
| 110 | ZINC000016343188 | 125.598 | 3473 | ZINC000001597294 | 87.8836 |
| 111 | ZINC000016343191 | 120.435 | 3474 | ZINC000001626971 | 79.1509 |
| 112 | ZINC000016343194 | 122.582 | 3475 | ZINC000001634150 | 84.2501 |
| 113 | ZINC000016343197 | 120.812 | 3476 | ZINC000001641013 | 72.5646 |
| 114 | ZINC000100777313 | 131.683 | 3477 | ZINC000001653218 | 101.208 |
| 115 | ZINC000004983273 | 77.1395 | 3478 | ZINC000001668230 | 70.3223 |
| 116 | ZINC000005008005 | 46.4845 | 3479 | ZINC000001669780 | 118.469 |
| 117 | ZINC000002384572 | 85.0833 | 3480 | ZINC000001675443 | 95.4139 |
| 118 | ZINC000004972689 | 61.7392 | 3481 | ZINC000002581983 | 74.1043 |
| 119 | ZINC000005020010 | 89.6224 | 3482 | ZINC000002584477 | 114.817 |
| 120 | ZINC000002384631 | 117.2 | 3483 | ZINC000002585420 | 87.5168 |
| 121 | ZINC000002384643 | 102.195 | 3484 | ZINC000002713312 | 100.809 |
| 122 | ZINC000002384645 | 74.4508 | 3485 | ZINC000003581248 | 75.2421 |
| 123 | ZINC000002384655 | 117.391 | 3486 | ZINC000003599047 | 85.1977 |
| 124 | ZINC000085881267 | 133.11 | 3487 | ZINC000003610022 | 117.962 |
| 125 | ZINC000001849640 | 83.4624 | 3488 | ZINC000103200603 | 74.8456 |
| 126 | ZINC000001532813 | 83.9784 | 3489 | ZINC000003651034 | 57.4569 |
| 127 | ZINC000001845978 | 104.172 | 3490 | ZINC000003860322 | 91.0347 |
| 128 | ZINC000001677787 | 100.394 | 3491 | ZINC000003860607 | 65.044 |
| 129 | ZINC000031500058 | 119.318 | 3492 | ZINC000003860659 | 79.6302 |
| 130 | ZINC000031500058 | 123.594 | 3493 | ZINC000003860818 | 60.544 |
| 131 | ZINC000031500052 | 118.744 | 3494 | ZINC000003860936 | 53.3672 |
| 132 | ZINC000031500052 | 119.07 | 3495 | ZINC000003861087 | 114.716 |
| 133 | ZINC000000295922 | 106.008 | 3496 | ZINC000003861336 | 61.5777 |
| 134 | ZINC000059436273 | 38.5519 | 3497 | ZINC000001600828 | 96.5479 |
| 135 | ZINC000059588412 | 102.141 | 3498 | ZINC000001608733 | 119.986 |
| 136 | ZINC000059725378 | 79.3975 | 3499 | ZINC000001648294 | 90.5532 |
| 137 | ZINC000004262304 | 61.8437 | 3500 | ZINC000001657745 | 83.1548 |
| 138 | ZINC000004262387 | 60.1328 | 3501 | ZINC000001665020 | 92.5755 |
| 139 | ZINC000000338304 | 112.645 | 3502 | ZINC000001668232 | 86.3508 |
| 140 | ZINC000053147419 | 77.426 | 3503 | ZINC000001673347 | 95.4495 |
| 141 | ZINC000053147420 | 93.5364 | 3504 | ZINC000001675771 | 55.8532 |
| 142 | ZINC000053147422 | 122.926 | 3505 | ZINC000002516010 | 80.1226 |
| 143 | ZINC000000000641 | 115.511 | 3506 | ZINC000002516016 | 83.9476 |
| 144 | ZINC000000001691 | 85.195 | 3507 | ZINC000002517078 | 79.7903 |
| 145 | ZINC000000001890 | 89.685 | 3508 | ZINC000002534689 | 103.749 |
| 146 | ZINC000000005608 | 111.941 | 3509 | ZINC000002534721 | 94.0588 |
| 147 | ZINC000000347886 | 106.581 | 3510 | ZINC000002555297 | 98.563 |
| 148 | ZINC000000490791 | 128.785 | 3511 | ZINC000002556747 | 109.26 |
| 149 | ZINC000002039624 | 114.163 | 3512 | ZINC000002557807 | 87.073 |
| 150 | ZINC000003874857 | 97.4534 | 3513 | ZINC000002557911 | 99.5898 |
| 151 | ZINC000003875469 | 129.601 | 3514 | ZINC000002558980 | 98.251 |
| 152 | ZINC000003881408 | 131.694 | 3515 | ZINC000002559095 | 107.438 |
| 153 | ZINC000003881421 | 120.751 | 3516 | ZINC000002560605 | 54.9111 |
| 154 | ZINC000003999653 | 112.716 | 3517 | ZINC000002561201 | 83.0651 |
| 155 | ZINC000004081771 | 125.213 | 3518 | ZINC000002563377 | 61.8093 |
| 156 | ZINC000004428527 | 121.505 | 3519 | ZINC000002563647 | 133.758 |
| 157 | ZINC000005763835 | 104.836 | 3520 | ZINC000002563976 | 54.2214 |
| 158 | ZINC000018113502 | 81.2773 | 3521 | ZINC000002564116 | 87.9152 |
| 159 | ZINC000008214966 | 121.05 | 3522 | ZINC000002566085 | 92.1721 |
| 160 | ZINC000004083557 | 93.2611 | 3523 | ZINC000002567766 | 108.002 |
| 161 | ZINC000000005878 | 76.7251 | 3524 | ZINC000002568012 | 62.7211 |
| 162 | ZINC000002011298 | 83.332 | 3525 | ZINC000002569363 | 75.4919 |
| 163 | ZINC000008214610 | 36.9423 | 3526 | ZINC000002575098 | 82.8289 |
| 164 | ZINC000008437654 | 37.5155 | 3527 | ZINC000002575201 | 87.872 |
| 165 | ZINC000002036797 | 98.164 | 3528 | ZINC000002577416 | 94.0401 |
| 166 | ZINC000002036970 | 105.792 | 3529 | ZINC000001599253 | 99.7174 |
| 167 | ZINC000002026704 | 81.2904 | 3530 | ZINC000001599379 | 72.0844 |
| 168 | ZINC000002041074 | 48.0707 | 3531 | ZINC000001612318 | 127.279 |
| 169 | ZINC000008215746 | 38.7454 | 3532 | ZINC000001626972 | 79.7974 |
| 170 | ZINC000002040988 | 127.884 | 3533 | ZINC000001641350 | 85.7303 |
| 171 | ZINC000002040979 | 108.647 | 3534 | ZINC000001648223 | 76.6606 |
| 172 | ZINC000000391144 | 67.1965 | 3535 | ZINC000001655483 | 72.5022 |
| 173 | ZINC000000391935 | 97.0471 | 3536 | ZINC000001664926 | 91.1244 |
| 174 | ZINC000000391109 | 66.3905 | 3537 | ZINC000001668231 | 94.7283 |
| 175 | ZINC000002041366 | 90.2692 | 3538 | ZINC000001671375 | 99.6339 |
| 176 | ZINC000002018445 | 88.1931 | 3539 | ZINC000001675676 | 87.1719 |
| 177 | ZINC000002029389 | 121.184 | 3540 | ZINC000002040666 | 72.8077 |
| 178 | ZINC000002031661 | 38.5634 | 3541 | ZINC000002041055 | 81.9077 |
| 179 | ZINC000002034597 | 63.7241 | 3542 | ZINC000002041071 | 56.3243 |
| 180 | ZINC000002040946 | 102.931 | 3543 | ZINC000002041154 | 105.913 |
| 181 | ZINC000002018446 | 88.6628 | 3544 | ZINC000002146674 | 73.4073 |
| 182 | ZINC000002019498 | 75.0154 | 3545 | ZINC000002164143 | 82.1619 |
| 183 | ZINC000002029393 | 113.036 | 3546 | ZINC000002169176 | 68.5795 |
| 184 | ZINC000002031664 | 83.5526 | 3547 | ZINC000002169365 | 85.8576 |
| 185 | ZINC000002034635 | 61.4841 | 3548 | ZINC000002169368 | 83.161 |
| 186 | ZINC000002035950 | 55.1122 | 3549 | ZINC000002173157 | 93.4502 |
| 187 | ZINC000002030901 | 94.6041 | 3550 | ZINC000002242704 | 66.079 |
| 188 | ZINC000005652375 | 67.965 | 3551 | ZINC000002381590 | 111.725 |
| 189 | ZINC000002041008 | 86.5351 | 3552 | ZINC000002382836 | 106.909 |
| 190 | ZINC000002041265 | 86.5327 | 3553 | ZINC000002383464 | 56.9746 |
| 191 | ZINC000002018675 | 79.3323 | 3554 | ZINC000002384609 | 89.0645 |
| 192 | ZINC000008294957 | 76.2484 | 3555 | ZINC000002389359 | 102.34 |
| 193 | ZINC000002041073 | 69.8311 | 3556 | ZINC000002391144 | 119.233 |
| 194 | ZINC000002037639 | 94.7857 | 3557 | ZINC000002504420 | 57.9543 |
| 195 | ZINC000002038278 | 88.5179 | 3558 | ZINC000002509971 | 74.7413 |
| 196 | ZINC000002039831 | 121.96 | 3559 | ZINC000002510149 | 76.0575 |
| 197 | ZINC000002001177 | 87.4407 | 3560 | ZINC000002510282 | 85.3195 |
| 198 | ZINC000002015944 | 87.7723 | 3561 | ZINC000002510287 | 73.6656 |
| 199 | ZINC000000002054 | 77.6656 | 3562 | ZINC000002510293 | 89.0433 |
| 200 | ZINC000053165481 | 91.5965 | 3563 | ZINC000001600505 | 98.7718 |
| 201 | ZINC000001532777 | 83.7895 | 3564 | ZINC000001605436 | 108.345 |
| 202 | ZINC000085836541 | 109.227 | 3565 | ZINC000001608901 | 53.8561 |
| 203 | ZINC000015113184 | 105.973 | 3566 | ZINC000001627234 | 80.787 |
| 204 | ZINC000014811261 | 114.156 | 3567 | ZINC000001631205 | 86.8313 |
| 205 | ZINC000056874629 | 94.6052 | 3568 | ZINC000001648298 | 98.9828 |
| 206 | ZINC000062233813 | 102.004 | 3569 | ZINC000001665640 | 117.906 |
| 207 | ZINC000044431032 | 93.6041 | 3570 | ZINC000001668234 | 77.2019 |
| 208 | ZINC000056874533 | 124.089 | 3571 | ZINC000001673420 | 63.785 |
| 209 | ZINC000001850884 | 50.0411 | 3572 | ZINC000001677092 | 105.72 |
| 210 | ZINC000001850911 | 101.6 | 3573 | ZINC000002018324 | 92.3635 |
| 211 | ZINC000001850974 | 80.8555 | 3574 | ZINC000002019414 | 76.4115 |
| 212 | ZINC000001851038 | 107.208 | 3575 | ZINC000002019416 | 82.3625 |
| 213 | ZINC000002545403 | 103.71 | 3576 | ZINC000002019685 | 78.923 |
| 214 | ZINC000000404384 | 77.3346 | 3577 | ZINC000002019768 | 77.7881 |
| 215 | ZINC000000405277 | 95.3528 | 3578 | ZINC000002020149 | 74.1292 |
| 216 | ZINC000000409387 | 64.5996 | 3579 | ZINC000002031351 | 90.4483 |
| 217 | ZINC000002569743 | 106.657 | 3580 | ZINC000002034318 | 75.0707 |
| 218 | ZINC000001849749 | 95.5589 | 3581 | ZINC000002034380 | 72.4914 |
| 219 | ZINC000002570182 | 114.184 | 3582 | ZINC000002034789 | 55.3818 |
| 220 | ZINC000005082283 | 97.1527 | 3583 | ZINC000002034874 | 81.7915 |
| 221 | ZINC000002046170 | 94.6669 | 3584 | ZINC000002036101 | 78.56 |
| 222 | ZINC000070455029 | 101.363 | 3585 | ZINC000002036798 | 102.564 |
| 223 | ZINC000065742963 | 112.143 | 3586 | ZINC000002036815 | 105.703 |
| 224 | ZINC000001577061 | 84.5522 | 3587 | ZINC000002036910 | 99.2768 |
| 225 | ZINC000001843030 | 95.2496 | 3588 | ZINC000002037726 | 63.2235 |
| 226 | ZINC000003651514 | 91.4407 | 3589 | ZINC000002037827 | 67.3872 |
| 227 | ZINC000022061023 | 100.168 | 3590 | ZINC000002037829 | 64.0535 |
| 228 | ZINC000000899824 | 115.1 | 3591 | ZINC000002037835 | 80.9241 |
| 229 | ZINC000000899824 | 119.249 | 3592 | ZINC000002037837 | 81.9886 |
| 230 | ZINC000065748339 | 110.367 | 3593 | ZINC000002037928 | 82.352 |
| 231 | ZINC000065748340 | 98.122 | 3594 | ZINC000002038824 | 110.075 |
| 232 | ZINC000065748340 | 110.367 | 3595 | ZINC000001069171 | 66.3787 |
| 233 | ZINC000000895969 | 40.6063 | 3596 | ZINC000001081105 | 71.8186 |
| 234 | ZINC000001843029 | 94.0207 | 3597 | ZINC000001081110 | 66.7833 |
| 235 | ZINC000003651513 | 104.146 | 3598 | ZINC000001081496 | 74.7752 |
| 236 | ZINC000003651516 | 103.281 | 3599 | ZINC000001090557 | 71.1348 |
| 237 | ZINC000003860369 | 112.683 | 3600 | ZINC000100010228 | 74.6883 |
| 238 | ZINC000005116994 | 84.551 | 3601 | ZINC000100023851 | 64.1754 |
| 239 | ZINC000008214588 | 107.085 | 3602 | ZINC000001561522 | 86.6928 |
| 240 | ZINC000022060952 | 109.056 | 3603 | ZINC000001566476 | 84.214 |
| 241 | ZINC000065749696 | 107.227 | 3604 | ZINC000001577445 | 79.2288 |
| 242 | ZINC000065749697 | 106.514 | 3605 | ZINC000001583785 | 95.872 |
| 243 | ZINC000029566455 | 109.913 | 3606 | ZINC000001586759 | 101.254 |
| 244 | ZINC000029566459 | 114.573 | 3607 | ZINC000001504642 | 91.9375 |
| 245 | ZINC000004025169 | 86.5377 | 3608 | ZINC000001529972 | 114.862 |
| 246 | ZINC000000388658 | 136.364 | 3609 | ZINC000001530331 | 80.8473 |
| 247 | ZINC000004811698 | 100.859 | 3610 | ZINC000100013228 | 73.8288 |
| 248 | ZINC000000056547 | 121.289 | 3611 | ZINC000001574399 | 56.9387 |
| 249 | ZINC000000074709 | 82.5778 | 3612 | ZINC000001576866 | 98.8153 |
| 250 | ZINC000033831958 | 141.894 | 3613 | ZINC000100048384 | 78.4062 |
| 251 | ZINC000000898659 | 105.153 | 3614 | ZINC000100056016 | 101.939 |
| 252 | ZINC000001614326 | 106.601 | 3615 | ZINC000001235108 | 96.4455 |
| 253 | ZINC000014860239 | 143.743 | 3616 | ZINC000001261423 | 74.6395 |
| 254 | ZINC000017197884 | 133.651 | 3617 | ZINC000001435981 | 67.4242 |
| 255 | ZINC000005999015 | 89.7481 | 3618 | ZINC000001531865 | 114.838 |
| 256 | ZINC000002566202 | 75.2927 | 3619 | ZINC000001537498 | 130.051 |
| 257 | ZINC000002567934 | 91.3844 | 3620 | ZINC000100011250 | 63.9628 |
| 258 | ZINC000002575198 | 106.89 | 3621 | ZINC000100024297 | 92.4132 |
| 259 | ZINC000004467398 | 91.8897 | 3622 | ZINC000001561524 | 80.341 |
| 260 | ZINC000004501380 | 54.391 | 3623 | ZINC000001576855 | 86.3606 |
| 261 | ZINC000004521669 | 69.1361 | 3624 | ZINC000100048378 | 78.8801 |
| 262 | ZINC000004528591 | 85.3092 | 3625 | ZINC000001577725 | 77.9867 |
| 263 | ZINC000001586382 | 81.7901 | 3626 | ZINC000001581503 | 117.008 |
| 264 | ZINC000001726893 | 66.6059 | 3627 | ZINC000001586760 | 80.071 |
| 265 | ZINC000001730614 | 80.4546 | 3628 | ZINC000001529305 | 91.9694 |
| 266 | ZINC000000967522 | 78.0592 | 3629 | ZINC000001532549 | 91.0827 |
| 267 | ZINC000001733699 | 54.5742 | 3630 | ZINC000100013729 | 57.0829 |
| 268 | ZINC000095619239 | 81.2057 | 3631 | ZINC000001561741 | 69.131 |
| 269 | ZINC000095618245 | 84.156 | 3632 | ZINC000001569924 | 101.678 |
| 270 | ZINC000097125343 | 54.9888 | 3633 | ZINC000001583154 | 67.484 |
| 271 | ZINC000071254667 | 92.2607 | 3634 | ZINC000001586314 | 73.8381 |
| 272 | ZINC000095618221 | 87.4384 | 3635 | ZINC000001587152 | 165.616 |
| 273 | ZINC000095617504 | 96.9194 | 3636 | ZINC000100075817 | 98.0883 |
| 274 | ZINC000095617959 | 87.7874 | 3637 | ZINC000000402722 | 118.073 |
| 275 | ZINC000095619240 | 85.9702 | 3638 | ZINC000000402960 | 99.118 |
| 276 | ZINC000086022667 | 109.897 | 3639 | ZINC000000403022 | 94.251 |
| 277 | ZINC000095618246 | 83.6398 | 3640 | ZINC000000407231 | 135.397 |
| 278 | ZINC000095618222 | 89.6529 | 3641 | ZINC000000896380 | 90.2508 |
| 279 | ZINC000001728129 | 109.854 | 3642 | ZINC000000897611 | 132.43 |
| 280 | ZINC000006030643 | 91.7923 | 3643 | ZINC000000898788 | 136.436 |
| 281 | ZINC000006031286 | 106.378 | 3644 | ZINC000000899115 | 117.54 |
| 282 | ZINC000033949373 | 135.985 | 3645 | ZINC000000899884 | 138.536 |
| 283 | ZINC000038220510 | 92.1842 | 3646 | ZINC000000967534 | 62.0757 |
| 284 | ZINC000038611097 | 97.6164 | 3647 | ZINC000000968250 | 91.8849 |
| 285 | ZINC000039217111 | 77.7259 | 3648 | ZINC000002017189 | 71.0614 |
| 286 | ZINC000039374927 | 87.0621 | 3649 | ZINC000000404389 | 86.7203 |
| 287 | ZINC000039374929 | 89.6891 | 3650 | ZINC000000404782 | 96.0975 |
| 288 | ZINC000043762293 | 112.512 | 3651 | ZINC000000404784 | 94.9639 |
| 289 | ZINC000044709727 | 74.9299 | 3652 | ZINC000000406940 | 108.997 |
| 290 | ZINC000002039410 | 84.1292 | 3653 | ZINC000000407027 | 56.6366 |
| 291 | ZINC000000402705 | 132.372 | 3654 | ZINC000000407077 | 79.9144 |
| 292 | ZINC000006021147 | 71.7922 | 3655 | ZINC000000895055 | 89.4809 |
| 293 | ZINC000006036939 | 73.9038 | 3656 | ZINC000000895958 | 142.826 |
| 294 | ZINC000006050189 | 133.159 | 3657 | ZINC000000896832 | 54.1753 |
| 295 | ZINC000033951616 | 77.4735 | 3658 | ZINC000000900525 | 80.1385 |
| 296 | ZINC000038220509 | 89.7843 | 3659 | ZINC000000901095 | 125.08 |
| 297 | ZINC000039217112 | 73.6241 | 3660 | ZINC000000967594 | 82.2398 |
| 298 | ZINC000039374928 | 84.9143 | 3661 | ZINC000000403089 | 90.5461 |
| 299 | ZINC000039374930 | 87.9379 | 3662 | ZINC000000403119 | 103.88 |
| 300 | ZINC000100293345 | 74.8527 | 3663 | ZINC000000403155 | 129.612 |
| 301 | ZINC000100293345 | 72.3637 | 3664 | ZINC000000404073 | 64.6773 |
| 302 | ZINC000001849655 | 72.5677 | 3665 | ZINC000000404273 | 84.174 |
| 303 | ZINC000006030233 | 70.2168 | 3666 | ZINC000000404377 | 66.2762 |
| 304 | ZINC000006037016 | 73.9675 | 3667 | ZINC000000896402 | 80.9558 |
| 305 | ZINC000006050191 | 132.907 | 3668 | ZINC000000899119 | 126.923 |
| 306 | ZINC000006070261 | 102.157 | 3669 | ZINC000000967542 | 90.2944 |
| 307 | ZINC000033953643 | 105.673 | 3670 | ZINC000001003581 | 114.58 |
| 308 | ZINC000032163906 | 73.4471 | 3671 | ZINC000000003182 | 68.9115 |
| 309 | ZINC000032165379 | 64.9597 | 3672 | ZINC000000003184 | 65.2806 |
| 310 | ZINC000032165383 | 60.3384 | 3673 | ZINC000000021790 | 103.305 |
| 311 | ZINC000032166512 | 70.5344 | 3674 | ZINC000000081248 | 88.441 |
| 312 | ZINC000032166516 | 71.184 | 3675 | ZINC000000120326 | 92.396 |
| 313 | ZINC000032166629 | 92.9227 | 3676 | ZINC000000135453 | 92.6358 |
| 314 | ZINC000032166953 | 75.9466 | 3677 | ZINC000000154095 | 72.214 |
| 315 | ZINC000032167539 | 89.9133 | 3678 | ZINC000000156868 | 81.0384 |
| 316 | ZINC000032167539 | 86.9774 | 3679 | ZINC000000157414 | 88.2594 |
| 317 | ZINC000032175843 | 63.228 | 3680 | ZINC000000159554 | 73.1629 |
| 318 | ZINC000032180821 | 88.4191 | 3681 | ZINC000000173360 | 80.4779 |
| 319 | ZINC000033645169 | 99.102 | 3682 | ZINC000000314951 | 89.9622 |
| 320 | ZINC000033838366 | 95.9936 | 3683 | ZINC000000388080 | 100.714 |
| 321 | ZINC000033839397 | 62.854 | 3684 | ZINC000000388442 | 86.3843 |
| 322 | ZINC000033841553 | 96.8527 | 3685 | ZINC000000389503 | 88.131 |
| 323 | ZINC000033842666 | 55.8465 | 3686 | ZINC000000394557 | 122.222 |
| 324 | ZINC000033846442 | 97.7356 | 3687 | ZINC000000397605 | 106.562 |
| 325 | ZINC000033951138 | 85.3853 | 3688 | ZINC000000402721 | 110.001 |
| 326 | ZINC000034265091 | 120.057 | 3689 | ZINC000000001904 | 92.8179 |
| 327 | ZINC000034278894 | 105.305 | 3690 | ZINC000000001937 | 135.647 |
| 328 | ZINC000034449169 | 91.7065 | 3691 | ZINC000000001953 | 87.3485 |
| 329 | ZINC000034743443 | 73.7882 | 3692 | ZINC000000002050 | 92.1982 |
| 330 | ZINC000001850336 | 95.3702 | 3693 | ZINC000000002067 | 100.223 |
| 331 | ZINC000002034811 | 82.451 | 3694 | ZINC000000073700 | 96.9438 |
| 332 | ZINC000000057359 | 105.89 | 3695 | ZINC000000120283 | 101.846 |
| 333 | ZINC000006067733 | 128.376 | 3696 | ZINC000000135449 | 99.4884 |
| 334 | ZINC000006070277 | 101.098 | 3697 | ZINC000000153660 | 99.4215 |
| 335 | ZINC000032163567 | 71.198 | 3698 | ZINC000000156526 | 74.2192 |
| 336 | ZINC000032163583 | 69.7725 | 3699 | ZINC000000157402 | 65.3195 |
| 337 | ZINC000032164794 | 83.0377 | 3700 | ZINC000000158760 | 65.6101 |
| 338 | ZINC000032164797 | 85.6139 | 3701 | ZINC000000173167 | 83.3939 |
| 339 | ZINC000032165377 | 58.6436 | 3702 | ZINC000000388076 | 80.5417 |
| 340 | ZINC000032165381 | 64.8945 | 3703 | ZINC000000388413 | 82.025 |
| 341 | ZINC000032166509 | 70.1373 | 3704 | ZINC000000388765 | 79.2551 |
| 342 | ZINC000032166514 | 75.8497 | 3705 | ZINC000000391976 | 131.854 |
| 343 | ZINC000032166520 | 78.2568 | 3706 | ZINC000000394340 | 81.3535 |
| 344 | ZINC000032166944 | 76.7251 | 3707 | ZINC000000397393 | 95.9524 |
| 345 | ZINC000032167536 | 89.9133 | 3708 | ZINC000000402720 | 128.244 |
| 346 | ZINC000032167536 | 86.9774 | 3709 | ZINC000000052601 | 101.397 |
| 347 | ZINC000032170010 | 76.1497 | 3710 | ZINC000000056530 | 103.172 |
| 348 | ZINC000032175845 | 63.1046 | 3711 | ZINC000000057585 | 82.5274 |
| 349 | ZINC000032228394 | 72.6054 | 3712 | ZINC000000102690 | 55.5656 |
| 350 | ZINC000032228394 | 73.194 | 3713 | ZINC000000120337 | 95.2116 |
| 351 | ZINC000033838365 | 94.1902 | 3714 | ZINC000000155720 | 76.1665 |
| 352 | ZINC000033839268 | 90.9999 | 3715 | ZINC000000156991 | 78.6296 |
| 353 | ZINC000033839585 | 95.5161 | 3716 | ZINC000000157471 | 62.5201 |
| 354 | ZINC000033841554 | 94.9087 | 3717 | ZINC000000163858 | 110.563 |
| 355 | ZINC000033844381 | 73.9389 | 3718 | ZINC000000265473 | 110.304 |
| 356 | ZINC000033846443 | 96.8676 | 3719 | ZINC000000388180 | 94.3149 |
| 357 | ZINC000033950748 | 97.591 | 3720 | ZINC000000388704 | 62.9638 |
| 358 | ZINC000034278895 | 108.745 | 3721 | ZINC000000391110 | 79.135 |
| 359 | ZINC000034617733 | 88.4937 | 3722 | ZINC000000393780 | 120.906 |
| 360 | ZINC000035464254 | 103.842 | 3723 | ZINC000000394965 | 103.536 |
| 361 | ZINC000006067627 | 127.775 | 3724 | ZINC000000402695 | 111.26 |
| 362 | ZINC000006070419 | 99.5294 | 3725 | ZINC000100826429 | 93.2623 |
| 363 | ZINC000032840808 | 89.7827 | 3726 | ZINC000000120539 | 87.1426 |
| 364 | ZINC000015048174 | 116.191 | 3727 | ZINC000000156048 | 82.891 |
| 365 | ZINC000015119192 | 84.6186 | 3728 | ZINC000000157145 | 88.3649 |
| 366 | ZINC000015119986 | 77.1747 | 3729 | ZINC000000158146 | 73.3145 |
| 367 | ZINC000015120356 | 98.1666 | 3730 | ZINC000000164484 | 65.4738 |
| 368 | ZINC000015120455 | 106.38 | 3731 | ZINC000000338222 | 129.196 |
| 369 | ZINC000015120487 | 86.8385 | 3732 | ZINC000000338222 | 122.763 |
| 370 | ZINC000015120744 | 86.546 | 3733 | ZINC000000388293 | 96.9902 |
| 371 | ZINC000015120821 | 85.2263 | 3734 | ZINC000000391793 | 69.7298 |
| 372 | ZINC000015121942 | 134.26 | 3735 | ZINC000000395098 | 82.9408 |
| 373 | ZINC000015146068 | 98.1838 | 3736 | ZINC000006070309 | 80.1782 |
| 374 | ZINC000015160858 | 49.1936 | 3737 | ZINC000006071877 | 83.4976 |
| 375 | ZINC000015160860 | 70.6257 | 3738 | ZINC000006072072 | 107.542 |
| 376 | ZINC000015207465 | 91.0823 | 3739 | ZINC000006072327 | 99.5006 |
| 377 | ZINC000015209579 | 101.1 | 3740 | ZINC000006092198 | 146.446 |
| 378 | ZINC000015221890 | 103.438 | 3741 | ZINC000006092239 | 109.636 |
| 379 | ZINC000015221896 | 102.175 | 3742 | ZINC000006093219 | 118.362 |
| 380 | ZINC000015253419 | 83.4305 | 3743 | ZINC000006093399 | 135.064 |
| 381 | ZINC000015261745 | 112.309 | 3744 | ZINC000006117762 | 95.5544 |
| 382 | ZINC000017025158 | 104.885 | 3745 | ZINC000006117762 | 91.971 |
| 383 | ZINC000017411343 | 72.173 | 3746 | ZINC000006458014 | 144.462 |
| 384 | ZINC000028128258 | 116.613 | 3747 | ZINC000006483721 | 144.974 |
| 385 | ZINC000028539472 | 95.685 | 3748 | ZINC000006576154 | 98.082 |
| 386 | ZINC000031361507 | 58.1159 | 3749 | ZINC000001850468 | 113.433 |
| 387 | ZINC000001995224 | 69.7558 | 3750 | ZINC000001850498 | 100.653 |
| 388 | ZINC000000306694 | 92.7275 | 3751 | ZINC000001850595 | 59.0188 |
| 389 | ZINC000006041766 | 121.626 | 3752 | ZINC000001850708 | 97.2914 |
| 390 | ZINC000033605294 | 78.2696 | 3753 | ZINC000001850844 | 69.1949 |
| 391 | ZINC000033605294 | 76.2857 | 3754 | ZINC000001850844 | 74.5352 |
| 392 | ZINC000015119191 | 84.8962 | 3755 | ZINC000001850962 | 72.7491 |
| 393 | ZINC000015119193 | 104.902 | 3756 | ZINC000001867147 | 62.8919 |
| 394 | ZINC000015119985 | 76.7343 | 3757 | ZINC000001995292 | 87.8496 |
| 395 | ZINC000015120290 | 97.2619 | 3758 | ZINC000002003668 | 77.0583 |
| 396 | ZINC000015120357 | 99.5 | 3759 | ZINC000006018747 | 108.925 |
| 397 | ZINC000015120486 | 86.7291 | 3760 | ZINC000006019680 | 107.691 |
| 398 | ZINC000015120743 | 90.9629 | 3761 | ZINC000006030493 | 84.5318 |
| 399 | ZINC000015120820 | 85.2675 | 3762 | ZINC000006032054 | 78.4037 |
| 400 | ZINC000015121707 | 75.6468 | 3763 | ZINC000006037250 | 85.034 |
| 401 | ZINC000015121943 | 135.932 | 3764 | ZINC000006037454 | 88.6157 |
| 402 | ZINC000015160859 | 69.5567 | 3765 | ZINC000006037677 | 102.356 |
| 403 | ZINC000015204986 | 65.0409 | 3766 | ZINC000006068806 | 99.5833 |
| 404 | ZINC000015209575 | 93.7553 | 3767 | ZINC000001850478 | 95.678 |
| 405 | ZINC000015221887 | 100.792 | 3768 | ZINC000001850507 | 80.1243 |
| 406 | ZINC000015221893 | 101.559 | 3769 | ZINC000001850603 | 101.855 |
| 407 | ZINC000015261744 | 109.184 | 3770 | ZINC000001850663 | 78.6103 |
| 408 | ZINC000015270318 | 73.7552 | 3771 | ZINC000001850874 | 88.1952 |
| 409 | ZINC000026675136 | 74.7529 | 3772 | ZINC000001850874 | 88.0195 |
| 410 | ZINC000032155223 | 80.6679 | 3773 | ZINC000001851016 | 126.093 |
| 411 | ZINC000001841361 | 84.9799 | 3774 | ZINC000001874360 | 110.582 |
| 412 | ZINC000001849665 | 101.005 | 3775 | ZINC000002000964 | 93.3122 |
| 413 | ZINC000006031302 | 105.791 | 3776 | ZINC000002010383 | 79.765 |
| 414 | ZINC000006031990 | 75.7236 | 3777 | ZINC000002015850 | 57.0555 |
| 415 | ZINC000006037353 | 64.323 | 3778 | ZINC000002015915 | 69.5558 |
| 416 | ZINC000006037912 | 105.407 | 3779 | ZINC000005821934 | 80.6207 |
| 417 | ZINC000006068778 | 129.213 | 3780 | ZINC000005822249 | 80.9301 |
| 418 | ZINC000006070276 | 102.949 | 3781 | ZINC000005839582 | 74.7872 |
| 419 | ZINC000014658084 | 118.334 | 3782 | ZINC000005844826 | 83.3159 |
| 420 | ZINC000014680174 | 93.6001 | 3783 | ZINC000005863151 | 112.451 |
| 421 | ZINC000014684767 | 87.3367 | 3784 | ZINC000005863166 | 113.286 |
| 422 | ZINC000014690788 | 71.4448 | 3785 | ZINC000005863713 | 76.5275 |
| 423 | ZINC000014708247 | 90.5103 | 3786 | ZINC000005924776 | 87.2306 |
| 424 | ZINC000014714039 | 97.8525 | 3787 | ZINC000005933622 | 106.978 |
| 425 | ZINC000014719300 | 95.6886 | 3788 | ZINC000005934751 | 83.5693 |
| 426 | ZINC000014719903 | 115.863 | 3789 | ZINC000005934860 | 98.5167 |
| 427 | ZINC000014723446 | 82.1784 | 3790 | ZINC000005956847 | 75.7127 |
| 428 | ZINC000014765839 | 83.1328 | 3791 | ZINC000005964796 | 87.0969 |
| 429 | ZINC000014766024 | 110.119 | 3792 | ZINC000005997103 | 103.339 |
| 430 | ZINC000014772164 | 101.499 | 3793 | ZINC000005998287 | 66.483 |
| 431 | ZINC000014811592 | 134.097 | 3794 | ZINC000005998787 | 118.446 |
| 432 | ZINC000014818163 | 98.2231 | 3795 | ZINC000005999091 | 108.626 |
| 433 | ZINC000014823314 | 86.138 | 3796 | ZINC000005999205 | 129.19 |
| 434 | ZINC000014855779 | 107.555 | 3797 | ZINC000001850470 | 101.458 |
| 435 | ZINC000014859929 | 96.6927 | 3798 | ZINC000001850602 | 100.97 |
| 436 | ZINC000014859990 | 106.837 | 3799 | ZINC000001850751 | 93.9112 |
| 437 | ZINC000002003741 | 83.2409 | 3800 | ZINC000001850858 | 95.1823 |
| 438 | ZINC000002039412 | 86.2903 | 3801 | ZINC000001850988 | 94.9749 |
| 439 | ZINC000000488469 | 101.829 | 3802 | ZINC000001997105 | 79.7428 |
| 440 | ZINC000006070245 | 119.002 | 3803 | ZINC000002011524 | 75.531 |
| 441 | ZINC000032192158 | 84.7209 | 3804 | ZINC000005650711 | 80.8269 |
| 442 | ZINC000014652103 | 92.5166 | 3805 | ZINC000005722488 | 65.2734 |
| 443 | ZINC000014657476 | 96.2067 | 3806 | ZINC000005732688 | 96.7211 |
| 444 | ZINC000014680230 | 77.5128 | 3807 | ZINC000005732688 | 97.7918 |
| 445 | ZINC000014684772 | 89.8975 | 3808 | ZINC000005733059 | 105.799 |
| 446 | ZINC000014708250 | 88.2991 | 3809 | ZINC000005751120 | 94.7205 |
| 447 | ZINC000014714041 | 95.2798 | 3810 | ZINC000005751122 | 88.7689 |
| 448 | ZINC000014719306 | 103.241 | 3811 | ZINC000005759852 | 77.5817 |
| 449 | ZINC000014719905 | 114.831 | 3812 | ZINC000005761863 | 69.2703 |
| 450 | ZINC000014756811 | 138.011 | 3813 | ZINC000005761902 | 90.9926 |
| 451 | ZINC000014756811 | 138.622 | 3814 | ZINC000005767277 | 105.651 |
| 452 | ZINC000014766023 | 98.9015 | 3815 | ZINC000005771137 | 75.7574 |
| 453 | ZINC000014770819 | 83.5425 | 3816 | ZINC000005783274 | 64.1687 |
| 454 | ZINC000014772029 | 102.218 | 3817 | ZINC000005784545 | 113.772 |
| 455 | ZINC000014776358 | 98.0017 | 3818 | ZINC000005819403 | 80.626 |
| 456 | ZINC000014811594 | 141.104 | 3819 | ZINC000005819419 | 82.1416 |
| 457 | ZINC000014818165 | 101.825 | 3820 | ZINC000005820027 | 105.639 |
| 458 | ZINC000014823312 | 83.2371 | 3821 | ZINC000005820434 | 92.728 |
| 459 | ZINC000014859830 | 115.882 | 3822 | ZINC000005820498 | 98.2805 |
| 460 | ZINC000014859988 | 108.04 | 3823 | ZINC000005820598 | 95.8215 |
| 461 | ZINC000014860575 | 77.2253 | 3824 | ZINC000001850497 | 120.728 |
| 462 | ZINC000014960424 | 78.4509 | 3825 | ZINC000001850593 | 60.5006 |
| 463 | ZINC000005856362 | 56.2768 | 3826 | ZINC000001850626 | 66.9205 |
| 464 | ZINC000031500674 | 62.268 | 3827 | ZINC000001850674 | 74.5553 |
| 465 | ZINC000032166563 | 62.9758 | 3828 | ZINC000001850768 | 99.2382 |
| 466 | ZINC000032175839 | 61.547 | 3829 | ZINC000001850813 | 100.968 |
| 467 | ZINC000014584225 | 88.4579 | 3830 | ZINC000001850875 | 62.3053 |
| 468 | ZINC000014584229 | 90.323 | 3831 | ZINC000001851018 | 119.361 |
| 469 | ZINC000014585854 | 86.5063 | 3832 | ZINC000002000965 | 102.138 |
| 470 | ZINC000014588414 | 86.5651 | 3833 | ZINC000002010484 | 84.7909 |
| 471 | ZINC000014588463 | 88.1063 | 3834 | ZINC000005202740 | 85.3002 |
| 472 | ZINC000014588465 | 91.0794 | 3835 | ZINC000005226073 | 89.6401 |
| 473 | ZINC000014588470 | 87.0219 | 3836 | ZINC000005227198 | 54.9897 |
| 474 | ZINC000014588532 | 95.7455 | 3837 | ZINC000005298949 | 82.2834 |
| 475 | ZINC000014588554 | 98.3185 | 3838 | ZINC000005339827 | 90.3324 |
| 476 | ZINC000014588556 | 99.8282 | 3839 | ZINC000005356395 | 92.6533 |
| 477 | ZINC000014588733 | 87.5486 | 3840 | ZINC000005358286 | 93.2623 |
| 478 | ZINC000014588735 | 87.9745 | 3841 | ZINC000005392428 | 112.052 |
| 479 | ZINC000014588889 | 90.7332 | 3842 | ZINC000005439913 | 83.5682 |
| 480 | ZINC000014588937 | 90.5672 | 3843 | ZINC000005447649 | 93.6303 |
| 481 | ZINC000014588939 | 90.5151 | 3844 | ZINC000005455583 | 88.4559 |
| 482 | ZINC000014591035 | 69.1347 | 3845 | ZINC000005457565 | 75.3863 |
| 483 | ZINC000014591971 | 72.8039 | 3846 | ZINC000005512459 | 108.48 |
| 484 | ZINC000014592277 | 104.252 | 3847 | ZINC000005512600 | 107.234 |
| 485 | ZINC000014593064 | 78.4181 | 3848 | ZINC000005514846 | 83.0054 |
| 486 | ZINC000014613369 | 85.8418 | 3849 | ZINC000005524327 | 94.2592 |
| 487 | ZINC000014613371 | 87.6287 | 3850 | ZINC000005574965 | 110.542 |
| 488 | ZINC000014614538 | 75.5235 | 3851 | ZINC000005647271 | 83.9659 |
| 489 | ZINC000014616382 | 79.3309 | 3852 | ZINC000001687374 | 93.8728 |
| 490 | ZINC000014616800 | 95.3877 | 3853 | ZINC000001691984 | 67.4675 |
| 491 | ZINC000014616802 | 96.869 | 3854 | ZINC000001693643 | 99.2991 |
| 492 | ZINC000014616833 | 77.7052 | 3855 | ZINC000001697401 | 69.9707 |
| 493 | ZINC000014616838 | 86.8413 | 3856 | ZINC000001699998 | 65.2903 |
| 494 | ZINC000014616840 | 99.7002 | 3857 | ZINC000001710003 | 69.7754 |
| 495 | ZINC000014616854 | 79.9221 | 3858 | ZINC000100349759 | 90.2012 |
| 496 | ZINC000014619019 | 93.6099 | 3859 | ZINC000001747839 | 87.7654 |
| 497 | ZINC000001693693 | 90.4467 | 3860 | ZINC000001825278 | 79.8768 |
| 498 | ZINC000005854353 | 153.429 | 3861 | ZINC000001849897 | 105.335 |
| 499 | ZINC000005854633 | 95.7761 | 3862 | ZINC000005103852 | 104.26 |
| 500 | ZINC000005998227 | 124.164 | 3863 | ZINC000005115722 | 144.09 |
| 501 | ZINC000031284479 | 79.8098 | 3864 | ZINC000005115722 | 145.957 |
| 502 | ZINC000031500668 | 62.7358 | 3865 | ZINC000005132733 | 82.4504 |
| 503 | ZINC000031538516 | 106.22 | 3866 | ZINC000005157592 | 95.3467 |
| 504 | ZINC000032166556 | 74.7868 | 3867 | ZINC000005159516 | 91.5899 |
| 505 | ZINC000032166566 | 62.2528 | 3868 | ZINC000005167572 | 95.5889 |
| 506 | ZINC000032167694 | 60.1907 | 3869 | ZINC000005191516 | 87.1966 |
| 507 | ZINC000014585856 | 87.03 | 3870 | ZINC000005191778 | 83.2501 |
| 508 | ZINC000014588412 | 91.1079 | 3871 | ZINC000005191814 | 81.1367 |
| 509 | ZINC000014588464 | 94.8763 | 3872 | ZINC000005195179 | 122.9 |
| 510 | ZINC000014588466 | 93.0416 | 3873 | ZINC000005196560 | 55.7131 |
| 511 | ZINC000014588471 | 99.8063 | 3874 | ZINC000001680431 | 84.5366 |
| 512 | ZINC000014588553 | 98.0483 | 3875 | ZINC000001687400 | 107.417 |
| 513 | ZINC000014588555 | 96.9854 | 3876 | ZINC000001692439 | 51.7783 |
| 514 | ZINC000014588732 | 88.7649 | 3877 | ZINC000001693901 | 122.594 |
| 515 | ZINC000014588734 | 87.2636 | 3878 | ZINC000001701739 | 66.2393 |
| 516 | ZINC000014588887 | 95.9625 | 3879 | ZINC000001714287 | 125.51 |
| 517 | ZINC000014588936 | 86.8285 | 3880 | ZINC000001720584 | 72.0403 |
| 518 | ZINC000014588938 | 87.0513 | 3881 | ZINC000001729181 | 104.133 |
| 519 | ZINC000014588986 | 87.499 | 3882 | ZINC000001747873 | 119.426 |
| 520 | ZINC000014589671 | 74.5484 | 3883 | ZINC000001849914 | 67.2846 |
| 521 | ZINC000014590816 | 106.785 | 3884 | ZINC000004501354 | 53.1189 |
| 522 | ZINC000014591034 | 69.8392 | 3885 | ZINC000004501356 | 54.5913 |
| 523 | ZINC000014592276 | 104.412 | 3886 | ZINC000004501358 | 54.1523 |
| 524 | ZINC000014593063 | 82.8273 | 3887 | ZINC000004521351 | 89.734 |
| 525 | ZINC000014613368 | 85.2618 | 3888 | ZINC000004521598 | 92.9919 |
| 526 | ZINC000014613370 | 90.5288 | 3889 | ZINC000004529333 | 96.9878 |
| 527 | ZINC000014614408 | 87.0961 | 3890 | ZINC000004538042 | 93.6689 |
| 528 | ZINC000014614797 | 76.8523 | 3891 | ZINC000004791944 | 100.741 |
| 529 | ZINC000014616363 | 82.0349 | 3892 | ZINC000004802601 | 74.4593 |
| 530 | ZINC000014616799 | 95.3174 | 3893 | ZINC000004802634 | 58.8123 |
| 531 | ZINC000014616801 | 96.6769 | 3894 | ZINC000004802650 | 50.1518 |
| 532 | ZINC000014616834 | 80.3055 | 3895 | ZINC000004802667 | 51.8767 |
| 533 | ZINC000014616839 | 85.9494 | 3896 | ZINC000004826812 | 72.7634 |
| 534 | ZINC000014616841 | 101.268 | 3897 | ZINC000004826825 | 76.4519 |
| 535 | ZINC000005812872 | 122.9 | 3898 | ZINC000100257509 | 106.571 |
| 536 | ZINC000005922016 | 108.001 | 3899 | ZINC000001680396 | 74.5078 |
| 537 | ZINC000030731547 | 84.7608 | 3900 | ZINC000001687392 | 103.143 |
| 538 | ZINC000031500678 | 61.6398 | 3901 | ZINC000001693657 | 97.9507 |
| 539 | ZINC000032150846 | 70.5055 | 3902 | ZINC000001697411 | 113.189 |
| 540 | ZINC000032166554 | 72.5952 | 3903 | ZINC000001700946 | 102.563 |
| 541 | ZINC000032166565 | 63.9321 | 3904 | ZINC000001710004 | 71.7809 |
| 542 | ZINC000032167692 | 60.4213 | 3905 | ZINC000001840969 | 74.3662 |
| 543 | ZINC000014444362 | 114.724 | 3906 | ZINC000001843071 | 96.1105 |
| 544 | ZINC000014444367 | 120.38 | 3907 | ZINC000001846541 | 90.7579 |
| 545 | ZINC000014448159 | 87.9637 | 3908 | ZINC000001849913 | 62.5682 |
| 546 | ZINC000014448216 | 89.9495 | 3909 | ZINC000004098284 | 99.7024 |
| 547 | ZINC000014448219 | 91.687 | 3910 | ZINC000004098478 | 95.0784 |
| 548 | ZINC000014448222 | 90.0903 | 3911 | ZINC000004098690 | 115.354 |
| 549 | ZINC000014451129 | 71.0901 | 3912 | ZINC000004098732 | 122.209 |
| 550 | ZINC000014451129 | 73.0487 | 3913 | ZINC000004098732 | 112.69 |
| 551 | ZINC000014455080 | 134.272 | 3914 | ZINC000004098742 | 155.799 |
| 552 | ZINC000014487916 | 89.5557 | 3915 | ZINC000004098928 | 149.422 |
| 553 | ZINC000014487920 | 90.8378 | 3916 | ZINC000004128067 | 115.727 |
| 554 | ZINC000014489891 | 90.6683 | 3917 | ZINC000004164657 | 126.825 |
| 555 | ZINC000014489895 | 87.4306 | 3918 | ZINC000004234860 | 95.9571 |
| 556 | ZINC000014489906 | 97.5848 | 3919 | ZINC000004262494 | 55.8545 |
| 557 | ZINC000014490101 | 71.9402 | 3920 | ZINC000004262496 | 55.4703 |
| 558 | ZINC000014491184 | 102.522 | 3921 | ZINC000004271681 | 69.535 |
| 559 | ZINC000014491215 | 102.57 | 3922 | ZINC000004283873 | 89.3197 |
| 560 | ZINC000014491514 | 106.362 | 3923 | ZINC000004284429 | 92.1908 |
| 561 | ZINC000014495038 | 85.8682 | 3924 | ZINC000004284443 | 85.4202 |
| 562 | ZINC000014504375 | 141.178 | 3925 | ZINC000004303672 | 85.4089 |
| 563 | ZINC000014504596 | 106.42 | 3926 | ZINC000001680660 | 73.0355 |
| 564 | ZINC000014505294 | 90.6506 | 3927 | ZINC000001688763 | 67.9492 |
| 565 | ZINC000014559786 | 91.5334 | 3928 | ZINC000001692445 | 55.7286 |
| 566 | ZINC000001694566 | 82.572 | 3929 | ZINC000001699427 | 70.6464 |
| 567 | ZINC000031500671 | 64.93 | 3930 | ZINC000001701825 | 76.3081 |
| 568 | ZINC000031703222 | 86.9237 | 3931 | ZINC000001757674 | 109.113 |
| 569 | ZINC000032166561 | 68.0341 | 3932 | ZINC000001841214 | 95.7221 |
| 570 | ZINC000032175836 | 61.0049 | 3933 | ZINC000001847929 | 81.5999 |
| 571 | ZINC000014419848 | 95.4261 | 3934 | ZINC000003861377 | 54.4287 |
| 572 | ZINC000014444364 | 116.889 | 3935 | ZINC000003861659 | 61.6726 |
| 573 | ZINC000014448160 | 88.2003 | 3936 | ZINC000003861699 | 96.297 |
| 574 | ZINC000014448218 | 97.5546 | 3937 | ZINC000003872521 | 109.224 |
| 575 | ZINC000014448221 | 91.1301 | 3938 | ZINC000003874615 | 88.8762 |
| 576 | ZINC000014448224 | 92.0316 | 3939 | ZINC000003880801 | 58.2036 |
| 577 | ZINC000014451127 | 74.4188 | 3940 | ZINC000003881381 | 76.6795 |
| 578 | ZINC000014451127 | 74.6284 | 3941 | ZINC000003881710 | 86.0872 |
| 579 | ZINC000014455079 | 140.794 | 3942 | ZINC000003978494 | 127.885 |
| 580 | ZINC000014487914 | 85.0749 | 3943 | ZINC000003984116 | 131.38 |
| 581 | ZINC000014487918 | 94.5772 | 3944 | ZINC000004023243 | 105.257 |
| 582 | ZINC000014488919 | 78.633 | 3945 | ZINC000004023520 | 71.3769 |
| 583 | ZINC000014489893 | 89.0378 | 3946 | ZINC000004026533 | 136.617 |
| 584 | ZINC000014489897 | 87.576 | 3947 | ZINC000004095722 | 70.0603 |
| 585 | ZINC000014489908 | 95.2507 | 3948 | ZINC000004095850 | 100.784 |
| 586 | ZINC000014495014 | 83.4463 | 3949 | ZINC000004098245 | 94.6429 |
| 587 | ZINC000014504598 | 112 | 3950 | ZINC000004098281 | 96.7459 |
| 588 | ZINC000014505076 | 97.2743 | 3951 | ZINC000001614079 | 67.6862 |
| 589 | ZINC000000895896 | 79.9465 | 3952 | ZINC000001616682 | 95.8207 |
| 590 | ZINC000015255435 | 74.3635 | 3953 | ZINC000001624462 | 138.829 |
| 591 | ZINC000017129257 | 113.06 | 3954 | ZINC000001627247 | 100.826 |
| 592 | ZINC000017783685 | 95.0479 | 3955 | ZINC000001631214 | 70.587 |
| 593 | ZINC000022058494 | 82.7644 | 3956 | ZINC000001638458 | 59.0416 |
| 594 | ZINC000026671806 | 73.5484 | 3957 | ZINC000001648299 | 96.2074 |
| 595 | ZINC000013429392 | 90.0553 | 3958 | ZINC000001658900 | 109.58 |
| 596 | ZINC000013433517 | 91.4663 | 3959 | ZINC000001666748 | 75.91 |
| 597 | ZINC000013434010 | 91.4896 | 3960 | ZINC000001668235 | 85.3598 |
| 598 | ZINC000013437575 | 86.7967 | 3961 | ZINC000001677778 | 95.3703 |
| 599 | ZINC000013437585 | 97.9963 | 3962 | ZINC000100825046 | 101.506 |
| 600 | ZINC000013459852 | 68.7017 | 3963 | ZINC000002585906 | 121.229 |
| 601 | ZINC000013459858 | 68.7055 | 3964 | ZINC000003594418 | 94.9322 |
| 602 | ZINC000013460029 | 85.6008 | 3965 | ZINC000003599046 | 85.0226 |
| 603 | ZINC000013462028 | 74.926 | 3966 | ZINC000003647753 | 115.008 |
| 604 | ZINC000013462032 | 68.3369 | 3967 | ZINC000003860323 | 90.4182 |
| 605 | ZINC000013462805 | 71.3659 | 3968 | ZINC000003860606 | 61.598 |
| 606 | ZINC000013481244 | 90.8528 | 3969 | ZINC000003860658 | 81.5066 |
| 607 | ZINC000013484719 | 119.155 | 3970 | ZINC000003860808 | 44.1131 |
| 608 | ZINC000013485434 | 103.158 | 3971 | ZINC000003860819 | 61.1586 |
| 609 | ZINC000013521389 | 71.2648 | 3972 | ZINC000003860971 | 80.1027 |
| 610 | ZINC000005854502 | 156.57 | 3973 | ZINC000003861334 | 66.015 |
| 611 | ZINC000005963549 | 142.242 | 3974 | ZINC000003861345 | 65.2861 |
| 612 | ZINC000015217369 | 120.697 | 3975 | ZINC000001614643 | 107.757 |
| 613 | ZINC000018169811 | 105.276 | 3976 | ZINC000001632635 | 129.995 |
| 614 | ZINC000019884527 | 87.5408 | 3977 | ZINC000001640047 | 98.2563 |
| 615 | ZINC000024398176 | 79.1069 | 3978 | ZINC000001648322 | 115.38 |
| 616 | ZINC000013412949 | 104.15 | 3979 | ZINC000001651126 | 138.5 |
| 617 | ZINC000013429396 | 94.4259 | 3980 | ZINC000001651126 | 142.162 |
| 618 | ZINC000013433458 | 91.0903 | 3981 | ZINC000001661081 | 61.1921 |
| 619 | ZINC000013433788 | 101.14 | 3982 | ZINC000001668768 | 118.14 |
| 620 | ZINC000013437570 | 78.2152 | 3983 | ZINC000001675320 | 64.2797 |
| 621 | ZINC000013437581 | 94.6305 | 3984 | ZINC000001677784 | 100.907 |
| 622 | ZINC000013437588 | 97.8548 | 3985 | ZINC000100825049 | 100.274 |
| 623 | ZINC000013458468 | 82.322 | 3986 | ZINC000002515951 | 84.0299 |
| 624 | ZINC000013459856 | 73.8898 | 3987 | ZINC000002522787 | 87.1322 |
| 625 | ZINC000013460178 | 102.794 | 3988 | ZINC000002522787 | 81.0519 |
| 626 | ZINC000013461938 | 74.6207 | 3989 | ZINC000002529828 | 119.373 |
| 627 | ZINC000013462030 | 70.9783 | 3990 | ZINC000002539567 | 59.1942 |
| 628 | ZINC000013462034 | 68.8494 | 3991 | ZINC000002556384 | 131.351 |
| 629 | ZINC000013462240 | 115.739 | 3992 | ZINC000002557908 | 87.5428 |
| 630 | ZINC000013480032 | 70.1395 | 3993 | ZINC000002557912 | 102.649 |
| 631 | ZINC000013484901 | 93.5171 | 3994 | ZINC000002558136 | 119.565 |
| 632 | ZINC000013484901 | 96.1179 | 3995 | ZINC000002558139 | 46.2927 |
| 633 | ZINC000013508926 | 88.8267 | 3996 | ZINC000002561200 | 109.632 |
| 634 | ZINC000013520481 | 86.2758 | 3997 | ZINC000002563407 | 102.754 |
| 635 | ZINC000013601808 | 83.2403 | 3998 | ZINC000002564113 | 87.5821 |
| 636 | ZINC000014416824 | 109.963 | 3999 | ZINC000002567751 | 88.2065 |
| 637 | ZINC000000898481 | 83.7565 | 4000 | ZINC000002567866 | 78.8972 |
| 638 | ZINC000005998597 | 99.3218 | 4001 | ZINC000002570064 | 109.1 |
| 639 | ZINC000005998597 | 97.6894 | 4002 | ZINC000002572266 | 99.9444 |
| 640 | ZINC000006018743 | 148.332 | 4003 | ZINC000002573799 | 98.3923 |
| 641 | ZINC000017149354 | 75.5457 | 4004 | ZINC000002575200 | 61.157 |
| 642 | ZINC000018531805 | 82.2956 | 4005 | ZINC000002575440 | 74.7699 |
| 643 | ZINC000012496486 | 100.103 | 4006 | ZINC000002578956 | 78.744 |
| 644 | ZINC000013108258 | 113.365 | 4007 | ZINC000001614080 | 51.091 |
| 645 | ZINC000013306704 | 86.4136 | 4008 | ZINC000001620281 | 139.474 |
| 646 | ZINC000013334177 | 99.4831 | 4009 | ZINC000001648306 | 83.1563 |
| 647 | ZINC000013334179 | 96.7605 | 4010 | ZINC000001658901 | 139.245 |
| 648 | ZINC000013335068 | 101.969 | 4011 | ZINC000001666852 | 81.7623 |
| 649 | ZINC000013340552 | 134.778 | 4012 | ZINC000001677781 | 89.4013 |
| 650 | ZINC000013341229 | 114.767 | 4013 | ZINC000002039466 | 44.4091 |
| 651 | ZINC000013346758 | 104.156 | 4014 | ZINC000002040992 | 78.7035 |
| 652 | ZINC000013361115 | 103.262 | 4015 | ZINC000002041068 | 54.8007 |
| 653 | ZINC000013377373 | 112.02 | 4016 | ZINC000002041131 | 103.505 |
| 654 | ZINC000013377639 | 92.6762 | 4017 | ZINC000002041165 | 78.0521 |
| 655 | ZINC000013379088 | 109.419 | 4018 | ZINC000002046963 | 92.9149 |
| 656 | ZINC000013380360 | 112.73 | 4019 | ZINC000002165898 | 108.92 |
| 657 | ZINC000013383237 | 81.8864 | 4020 | ZINC000002169175 | 73.7899 |
| 658 | ZINC000013383500 | 71.8191 | 4021 | ZINC000002169190 | 103.494 |
| 659 | ZINC000013383774 | 98.4472 | 4022 | ZINC000002169346 | 80.0565 |
| 660 | ZINC000001578706 | 77.9101 | 4023 | ZINC000002169363 | 92.0666 |
| 661 | ZINC000001694564 | 85.1839 | 4024 | ZINC000002169366 | 83.5981 |
| 662 | ZINC000005783633 | 131.95 | 4025 | ZINC000002243723 | 73.5487 |
| 663 | ZINC000005854532 | 147.286 | 4026 | ZINC000002382739 | 61.4558 |
| 664 | ZINC000005998758 | 139.263 | 4027 | ZINC000002384606 | 80.3441 |
| 665 | ZINC000005998758 | 138.161 | 4028 | ZINC000002384629 | 101.594 |
| 666 | ZINC000017129255 | 115.639 | 4029 | ZINC000002384653 | 111.774 |
| 667 | ZINC000018125667 | 96.8176 | 4030 | ZINC000002387302 | 78.5909 |
| 668 | ZINC000020231212 | 112.186 | 4031 | ZINC000002389918 | 76.6063 |
| 669 | ZINC000012502019 | 88.2222 | 4032 | ZINC000002392256 | 134.581 |
| 670 | ZINC000013303595 | 115.16 | 4033 | ZINC000002504554 | 89.0501 |
| 671 | ZINC000013306523 | 110.374 | 4034 | ZINC000002506590 | 86.1226 |
| 672 | ZINC000013306700 | 85.3592 | 4035 | ZINC000002509929 | 64.5574 |
| 673 | ZINC000013334178 | 94.1696 | 4036 | ZINC000002509980 | 67.4608 |
| 674 | ZINC000013334180 | 93.1204 | 4037 | ZINC000002510107 | 91.0724 |
| 675 | ZINC000013340553 | 139.902 | 4038 | ZINC000002510214 | 95.9153 |
| 676 | ZINC000013340870 | 129.032 | 4039 | ZINC000002510285 | 85.9542 |
| 677 | ZINC000013377095 | 116.194 | 4040 | ZINC000002510290 | 72.4341 |
| 678 | ZINC000013377375 | 106.225 | 4041 | ZINC000001615347 | 73.7789 |
| 679 | ZINC000013378415 | 115.097 | 4042 | ZINC000001640814 | 66.6447 |
| 680 | ZINC000013381684 | 101.011 | 4043 | ZINC000001646630 | 78.0252 |
| 681 | ZINC000013382833 | 82.3526 | 4044 | ZINC000001653028 | 79.0172 |
| 682 | ZINC000013383238 | 78.4165 | 4045 | ZINC000001668229 | 71.5116 |
| 683 | ZINC000013383501 | 70.8541 | 4046 | ZINC000001675421 | 82.2507 |
| 684 | ZINC000005158963 | 140.751 | 4047 | ZINC000001677806 | 107.771 |
| 685 | ZINC000005158963 | 150.548 | 4048 | ZINC000002018181 | 86.9249 |
| 686 | ZINC000005211474 | 87.2951 | 4049 | ZINC000002019415 | 78.0373 |
| 687 | ZINC000005386772 | 128.962 | 4050 | ZINC000002019417 | 72.7858 |
| 688 | ZINC000005688565 | 77.2458 | 4051 | ZINC000002019686 | 75.834 |
| 689 | ZINC000015120346 | 93.9765 | 4052 | ZINC000002020134 | 70.9888 |
| 690 | ZINC000015120611 | 125.409 | 4053 | ZINC000002029851 | 107.084 |
| 691 | ZINC000015120797 | 102.852 | 4054 | ZINC000002031331 | 105.079 |
| 692 | ZINC000015146195 | 117.733 | 4055 | ZINC000002031470 | 83.174 |
| 693 | ZINC000015150645 | 107.297 | 4056 | ZINC000002031649 | 52.8271 |
| 694 | ZINC000006019709 | 91.6039 | 4057 | ZINC000002033904 | 81.8306 |
| 695 | ZINC000006030349 | 108.266 | 4058 | ZINC000002034319 | 73.9159 |
| 696 | ZINC000006030766 | 87.8526 | 4059 | ZINC000002034328 | 80.3443 |
| 697 | ZINC000006031585 | 95.3792 | 4060 | ZINC000002034503 | 54.6773 |
| 698 | ZINC000006031817 | 62.2801 | 4061 | ZINC000002034637 | 62.6807 |
| 699 | ZINC000006037008 | 91.6848 | 4062 | ZINC000002034834 | 86.0857 |
| 700 | ZINC000006037450 | 88.5678 | 4063 | ZINC000002035722 | 66.1589 |
| 701 | ZINC000006037655 | 73.809 | 4064 | ZINC000002036726 | 38.1253 |
| 702 | ZINC000006038030 | 99.6054 | 4065 | ZINC000002036801 | 113.186 |
| 703 | ZINC000006067069 | 92.8373 | 4066 | ZINC000002036909 | 100.356 |
| 704 | ZINC000006070772 | 100.973 | 4067 | ZINC000002037267 | 76.4873 |
| 705 | ZINC000006071115 | 111.097 | 4068 | ZINC000002037537 | 82.2813 |
| 706 | ZINC000006072098 | 89.1922 | 4069 | ZINC000002037668 | 55.1658 |
| 707 | ZINC000006117172 | 84.218 | 4070 | ZINC000002037803 | 77.3928 |
| 708 | ZINC000006361277 | 82.2889 | 4071 | ZINC000002037828 | 63.3593 |
| 709 | ZINC000006494870 | 84.5189 | 4072 | ZINC000002037834 | 81.1468 |
| 710 | ZINC000008234293 | 98.5754 | 4073 | ZINC000002037836 | 74.1849 |
| 711 | ZINC000012153418 | 83.9709 | 4074 | ZINC000001531083 | 61.6736 |
| 712 | ZINC000012153444 | 82.5247 | 4075 | ZINC000001531861 | 121.244 |
| 713 | ZINC000012153769 | 114.811 | 4076 | ZINC000100017211 | 88.2624 |
| 714 | ZINC000001607934 | 65.5849 | 4077 | ZINC000001562141 | 67.6918 |
| 715 | ZINC000001664401 | 87.8312 | 4078 | ZINC000100047338 | 54.6807 |
| 716 | ZINC000001697408 | 79.8714 | 4079 | ZINC000001583510 | 83.3251 |
| 717 | ZINC000005298828 | 53.0849 | 4080 | ZINC000001586316 | 82.7283 |
| 718 | ZINC000005509484 | 87.1313 | 4081 | ZINC000001587582 | 73.3952 |
| 719 | ZINC000005649494 | 84.6368 | 4082 | ZINC000100824169 | 76.6725 |
| 720 | ZINC000015120613 | 79.9621 | 4083 | ZINC000100830171 | 83.0868 |
| 721 | ZINC000015120795 | 98.2143 | 4084 | ZINC000001531096 | 95.8045 |
| 722 | ZINC000006020345 | 79.0149 | 4085 | ZINC000001565353 | 175.657 |
| 723 | ZINC000006030769 | 85.4252 | 4086 | ZINC000001565353 | 144.788 |
| 724 | ZINC000006031742 | 94.9211 | 4087 | ZINC000001570892 | 100.337 |
| 725 | ZINC000006031851 | 91.7614 | 4088 | ZINC000100047769 | 100.437 |
| 726 | ZINC000006037122 | 105.93 | 4089 | ZINC000001577443 | 81.7646 |
| 727 | ZINC000006037453 | 87.7445 | 4090 | ZINC000100065283 | 123.744 |
| 728 | ZINC000006066725 | 92.0829 | 4091 | ZINC000001586528 | 62.2525 |
| 729 | ZINC000006066936 | 98.2512 | 4092 | ZINC000001433173 | 69.3067 |
| 730 | ZINC000006069801 | 99.9422 | 4093 | ZINC000001564959 | 129.148 |
| 731 | ZINC000006071114 | 110.653 | 4094 | ZINC000100047763 | 70.8715 |
| 732 | ZINC000006071999 | 80.2008 | 4095 | ZINC000001577442 | 80.0209 |
| 733 | ZINC000006117171 | 82.1928 | 4096 | ZINC000001583724 | 77.9727 |
| 734 | ZINC000006488104 | 80.0785 | 4097 | ZINC000001586523 | 113.818 |
| 735 | ZINC000006495406 | 97.5554 | 4098 | ZINC000001533462 | 104.661 |
| 736 | ZINC000012153082 | 91.7737 | 4099 | ZINC000100023849 | 64.4637 |
| 737 | ZINC000012153416 | 96.895 | 4100 | ZINC000001565456 | 88.2936 |
| 738 | ZINC000012153443 | 81.4011 | 4101 | ZINC000001571092 | 86.5015 |
| 739 | ZINC000012153446 | 86.1108 | 4102 | ZINC000001577444 | 79.5958 |
| 740 | ZINC000000967584 | 76.0305 | 4103 | ZINC000001586734 | 60.8533 |
| 741 | ZINC000005195818 | 130.612 | 4104 | ZINC000000409238 | 96.6308 |
| 742 | ZINC000005195818 | 133.221 | 4105 | ZINC000000409287 | 66.2724 |
| 743 | ZINC000005298988 | 80.405 | 4106 | ZINC000000409332 | 76.7952 |
| 744 | ZINC000005510218 | 100.8 | 4107 | ZINC000000409359 | 76.4101 |
| 745 | ZINC000005732362 | 107.756 | 4108 | ZINC000000410089 | 91.9565 |
| 746 | ZINC000005732362 | 107.818 | 4109 | ZINC000000493111 | 95.0706 |
| 747 | ZINC000005758832 | 120.293 | 4110 | ZINC000000520945 | 124.273 |
| 748 | ZINC000015120480 | 132.857 | 4111 | ZINC000000896153 | 79.33 |
| 749 | ZINC000015120796 | 108.101 | 4112 | ZINC000000898098 | 134.222 |
| 750 | ZINC000100828525 | 53.2724 | 4113 | ZINC000000899902 | 142.062 |
| 751 | ZINC000100828528 | 60.4322 | 4114 | ZINC000000900727 | 117.886 |
| 752 | ZINC000100828565 | 58.003 | 4115 | ZINC000000901356 | 100.009 |
| 753 | ZINC000005132601 | 78.6828 | 4116 | ZINC000000967635 | 87.5273 |
| 754 | ZINC000005133244 | 75.714 | 4117 | ZINC000002017409 | 84.8882 |
| 755 | ZINC000005138489 | 139.375 | 4118 | ZINC000000409241 | 77.2478 |
| 756 | ZINC000005157245 | 63.1854 | 4119 | ZINC000000409294 | 81.4264 |
| 757 | ZINC000005159624 | 98.2277 | 4120 | ZINC000000409334 | 70.8728 |
| 758 | ZINC000005355851 | 97.9338 | 4121 | ZINC000000410079 | 111.548 |
| 759 | ZINC000005359455 | 75.9655 | 4122 | ZINC000000410301 | 75.2492 |
| 760 | ZINC000005441040 | 90.15 | 4123 | ZINC000000493113 | 96.5052 |
| 761 | ZINC000005510517 | 104.929 | 4124 | ZINC000000895934 | 79.5541 |
| 762 | ZINC000005751230 | 99.9484 | 4125 | ZINC000000896169 | 86.6567 |
| 763 | ZINC000005757442 | 84.8312 | 4126 | ZINC000000899562 | 121.94 |
| 764 | ZINC000005761140 | 87.9946 | 4127 | ZINC000000899845 | 77.9658 |
| 765 | ZINC000005761369 | 81.1445 | 4128 | ZINC000000967333 | 91.7051 |
| 766 | ZINC000005767327 | 83.7061 | 4129 | ZINC000000967720 | 95.7095 |
| 767 | ZINC000005783272 | 64.4397 | 4130 | ZINC000100823353 | 84.6921 |
| 768 | ZINC000005844372 | 74.4794 | 4131 | ZINC000000409239 | 99.7622 |
| 769 | ZINC000005923355 | 112.495 | 4132 | ZINC000000409293 | 70.758 |
| 770 | ZINC000001581357 | 98.9764 | 4133 | ZINC000000409333 | 71.0184 |
| 771 | ZINC000001694437 | 81.7168 | 4134 | ZINC000000410077 | 118.42 |
| 772 | ZINC000005455871 | 79.691 | 4135 | ZINC000000410090 | 88.3116 |
| 773 | ZINC000015119267 | 90.8015 | 4136 | ZINC000000493112 | 96.427 |
| 774 | ZINC000015120008 | 86.4339 | 4137 | ZINC000000896168 | 85.626 |
| 775 | ZINC000015120347 | 97.0526 | 4138 | ZINC000000900814 | 69.0672 |
| 776 | ZINC000015120794 | 102.865 | 4139 | ZINC000000967330 | 65.2212 |
| 777 | ZINC000100828523 | 60.4102 | 4140 | ZINC000000967715 | 89.0903 |
| 778 | ZINC000100828526 | 60.3426 | 4141 | ZINC000000404781 | 98.2912 |
| 779 | ZINC000005115341 | 91.2235 | 4142 | ZINC000000404783 | 92.39 |
| 780 | ZINC000005132594 | 83.6035 | 4143 | ZINC000000406939 | 107.776 |
| 781 | ZINC000005157059 | 63.3197 | 4144 | ZINC000000406974 | 94.3331 |
| 782 | ZINC000005157263 | 84.6488 | 4145 | ZINC000000407028 | 65.5716 |
| 783 | ZINC000005158581 | 117.268 | 4146 | ZINC000000407078 | 79.8172 |
| 784 | ZINC000005159654 | 99.2459 | 4147 | ZINC000000409219 | 81.5189 |
| 785 | ZINC000005196288 | 76.1402 | 4148 | ZINC000000896073 | 77.0324 |
| 786 | ZINC000005334404 | 92.4106 | 4149 | ZINC000000897131 | 68.4858 |
| 787 | ZINC000005355915 | 98.4802 | 4150 | ZINC000000900539 | 113.45 |
| 788 | ZINC000005441043 | 89.009 | 4151 | ZINC000000901192 | 55.6329 |
| 789 | ZINC000005510509 | 102.031 | 4152 | ZINC000000968074 | 140.461 |
| 790 | ZINC000005566501 | 68.1064 | 4153 | ZINC000100828279 | 83.5487 |
| 791 | ZINC000005699887 | 73.1498 | 4154 | ZINC000000409274 | 69.4944 |
| 792 | ZINC000005742757 | 81.9576 | 4155 | ZINC000000409299 | 68.781 |
| 793 | ZINC000005752287 | 77.6857 | 4156 | ZINC000000409355 | 67.8152 |
| 794 | ZINC000005761276 | 78.6136 | 4157 | ZINC000000410082 | 104.338 |
| 795 | ZINC000005761501 | 78.5727 | 4158 | ZINC000000493110 | 94.408 |
| 796 | ZINC000005767208 | 84.1134 | 4159 | ZINC000000896284 | 90.2061 |
| 797 | ZINC000005768312 | 86.4123 | 4160 | ZINC000000897490 | 39.3502 |
| 798 | ZINC000005820095 | 86.4111 | 4161 | ZINC000000899671 | 129.154 |
| 799 | ZINC000005831820 | 78.108 | 4162 | ZINC000000901447 | 72.9395 |
| 800 | ZINC000005844820 | 82.8941 | 4163 | ZINC000000967412 | 93.0351 |
| 801 | ZINC000001685644 | 72.7204 | 4164 | ZINC000000967734 | 97.0043 |
| 802 | ZINC000005440783 | 100.514 | 4165 | ZINC000000000048 | 80.9388 |
| 803 | ZINC000005735747 | 83.0251 | 4166 | ZINC000000000076 | 107.379 |
| 804 | ZINC000014779276 | 88.8599 | 4167 | ZINC000000000352 | 107.045 |
| 805 | ZINC000014811058 | 153.541 | 4168 | ZINC000000000868 | 82.7274 |
| 806 | ZINC000014819519 | 114.385 | 4169 | ZINC000000001219 | 124.596 |
| 807 | ZINC000014820405 | 135.105 | 4170 | ZINC000000073686 | 105.945 |
| 808 | ZINC000014886646 | 129.564 | 4171 | ZINC000000119985 | 143.624 |
| 809 | ZINC000100216788 | 67.1547 | 4172 | ZINC000000130187 | 64.2125 |
| 810 | ZINC000100774878 | 85.3501 | 4173 | ZINC000000153027 | 69.6551 |
| 811 | ZINC000100776371 | 81.7187 | 4174 | ZINC000000157401 | 66.7945 |
| 812 | ZINC000100778694 | 96.0587 | 4175 | ZINC000000158629 | 88.8816 |
| 813 | ZINC000100779713 | 82.5487 | 4176 | ZINC000000164504 | 82.74 |
| 814 | ZINC000100779781 | 92.1491 | 4177 | ZINC000000265504 | 112.1 |
| 815 | ZINC000100779786 | 85.1469 | 4178 | ZINC000000388064 | 73.2316 |
| 816 | ZINC000100781111 | 73.1547 | 4179 | ZINC000000388339 | 80.9147 |
| 817 | ZINC000100781732 | 84.8312 | 4180 | ZINC000000388764 | 77.817 |
| 818 | ZINC000100782219 | 90.647 | 4181 | ZINC000000391824 | 80.3025 |
| 819 | ZINC000100784267 | 90.1704 | 4182 | ZINC000000393900 | 94.1124 |
| 820 | ZINC000100828303 | 93.844 | 4183 | ZINC000000396101 | 102.908 |
| 821 | ZINC000100828308 | 81.1155 | 4184 | ZINC000000402719 | 117.876 |
| 822 | ZINC000100828313 | 87.7624 | 4185 | ZINC000100827440 | 90.1694 |
| 823 | ZINC000100828322 | 72.9229 | 4186 | ZINC000000051924 | 122.863 |
| 824 | ZINC000001578707 | 81.1171 | 4187 | ZINC000000056529 | 99.056 |
| 825 | ZINC000001667423 | 83.4469 | 4188 | ZINC000000057912 | 104.534 |
| 826 | ZINC000005298936 | 83.12 | 4189 | ZINC000000120332 | 105.9 |
| 827 | ZINC000005510216 | 116.86 | 4190 | ZINC000000150863 | 60.7595 |
| 828 | ZINC000014811582 | 134.618 | 4191 | ZINC000000154666 | 87.2584 |
| 829 | ZINC000014814973 | 125.771 | 4192 | ZINC000000156980 | 67.4928 |
| 830 | ZINC000014814973 | 124.462 | 4193 | ZINC000000157469 | 63.8822 |
| 831 | ZINC000014820400 | 138.445 | 4194 | ZINC000000161206 | 60.5466 |
| 832 | ZINC000014927070 | 96.6454 | 4195 | ZINC000000236100 | 112.129 |
| 833 | ZINC000100307836 | 81.1295 | 4196 | ZINC000000346083 | 125.499 |
| 834 | ZINC000004995652 | 78.0211 | 4197 | ZINC000000388119 | 58.5764 |
| 835 | ZINC000100776368 | 88.0406 | 4198 | ZINC000000388671 | 92.5535 |
| 836 | ZINC000100778695 | 81.1255 | 4199 | ZINC000000389865 | 89.0269 |
| 837 | ZINC000100779312 | 95.3571 | 4200 | ZINC000000394936 | 102.805 |
| 838 | ZINC000100779492 | 91.9547 | 4201 | ZINC000000402693 | 121.899 |
| 839 | ZINC000100779783 | 85.491 | 4202 | ZINC000012341544 | 70.374 |
| 840 | ZINC000100779788 | 90.1721 | 4203 | ZINC000012341951 | 111.574 |
| 841 | ZINC000100781105 | 73.302 | 4204 | ZINC000012353732 | 96.546 |
| 842 | ZINC000100782218 | 85.4938 | 4205 | ZINC000012358644 | 79.9185 |
| 843 | ZINC000100828299 | 84.5558 | 4206 | ZINC000012358664 | 47.675 |
| 844 | ZINC000100828304 | 94.5674 | 4207 | ZINC000012358780 | 73.1059 |
| 845 | ZINC000100828310 | 89.1386 | 4208 | ZINC000012358877 | 80.7413 |
| 846 | ZINC000100828320 | 71.6579 | 4209 | ZINC000012358983 | 81.045 |
| 847 | ZINC000005299005 | 83.769 | 4210 | ZINC000012362995 | 74.8068 |
| 848 | ZINC000005362481 | 65.0967 | 4211 | ZINC000012370861 | 67.4139 |
| 849 | ZINC000005511592 | 96.0975 | 4212 | ZINC000012375086 | 119.717 |
| 850 | ZINC000005665087 | 103.6 | 4213 | ZINC000012402005 | 108.494 |
| 851 | ZINC000005733763 | 75.2907 | 4214 | ZINC000012402448 | 69.9834 |
| 852 | ZINC000014806341 | 131.803 | 4215 | ZINC000012404985 | 65.5094 |
| 853 | ZINC000014811056 | 143.444 | 4216 | ZINC000012405252 | 86.166 |
| 854 | ZINC000014819517 | 132.727 | 4217 | ZINC000012410579 | 103.108 |
| 855 | ZINC000014819517 | 132.22 | 4218 | ZINC000012418177 | 88.6022 |
| 856 | ZINC000014886643 | 129.04 | 4219 | ZINC000005761927 | 87.1223 |
| 857 | ZINC000014918650 | 99.8988 | 4220 | ZINC000005758615 | 86.1446 |
| 858 | ZINC000000899429 | 147.436 | 4221 | ZINC000011568127 | 108.07 |
| 859 | ZINC000001850638 | 100.635 | 4222 | ZINC000011568128 | 106.511 |
| 860 | ZINC000003869858 | 120.683 | 4223 | ZINC000006037471 | 93.835 |
| 861 | ZINC000003869858 | 125.412 | 4224 | ZINC000004025995 | 91.1109 |
| 862 | ZINC000004476635 | 94.9366 | 4225 | ZINC000000388060 | 93.4668 |
| 863 | ZINC000004481767 | 94.9869 | 4226 | ZINC000000388079 | 118.355 |
| 864 | ZINC000100068817 | 92.6005 | 4227 | ZINC000012153042 | 116.307 |
| 865 | ZINC000004533096 | 59.3159 | 4228 | ZINC000012153559 | 84.0865 |
| 866 | ZINC000100128032 | 70.091 | 4229 | ZINC000012153719 | 126.579 |
| 867 | ZINC000005167610 | 104.7 | 4230 | ZINC000012153686 | 88.3214 |
| 868 | ZINC000005298826 | 52.4384 | 4231 | ZINC000005765036 | 110.196 |
| 869 | ZINC000005412472 | 121.015 | 4232 | ZINC000006069162 | 106.022 |
| 870 | ZINC000005647497 | 92.8303 | 4233 | ZINC000006093351 | 130.95 |
| 871 | ZINC000005735772 | 83.7306 | 4234 | ZINC000008602415 | 89.1428 |
| 872 | ZINC000014779724 | 99.7537 | 4235 | ZINC000002016091 | 92.6644 |
| 873 | ZINC000014811580 | 132.877 | 4236 | ZINC000000391913 | 75.9071 |
| 874 | ZINC000014814969 | 131.118 | 4237 | ZINC000015253562 | 82.9396 |
| 875 | ZINC000014814969 | 136.556 | 4238 | ZINC000002038280 | 93.7526 |
| 876 | ZINC000014819208 | 118.869 | 4239 | ZINC000002039833 | 98.2081 |
| 877 | ZINC000014819753 | 129.297 | 4240 | ZINC000002040153 | 89.5088 |
| 878 | ZINC000014820415 | 137.689 | 4241 | ZINC000021989120 | 97.4686 |
| 879 | ZINC000014822203 | 81.189 | 4242 | ZINC000026895132 | 73.8603 |
| 880 | ZINC000014825436 | 140.681 | 4243 | ZINC000000058124 | 94.0318 |
| 881 | ZINC000014826093 | 129.626 | 4244 | ZINC000003834173 | 97.9476 |
| 882 | ZINC000001559232 | 146.68 | 4245 | ZINC000003831578 | 119.021 |
| 883 | ZINC000001850637 | 101.938 | 4246 | ZINC000000895800 | 88.8987 |
| 884 | ZINC000001850662 | 82.5033 | 4247 | ZINC000015207470 | 105.178 |
| 885 | ZINC000001858795 | 115.245 | 4248 | ZINC000014719304 | 102.528 |
| 886 | ZINC000002560879 | 71.4904 | 4249 | ZINC000091297329 | 163.192 |
| 887 | ZINC000003644819 | 86.0518 | 4250 | ZINC000015112395 | 135.315 |
| 888 | ZINC000100067690 | 99.6017 | 4251 | ZINC000006031334 | 101.15 |
| 889 | ZINC000004533097 | 58.08 | 4252 | ZINC000013377374 | 108.019 |
| 890 | ZINC000014719978 | 111.562 | 4253 | ZINC000013377376 | 114.144 |
| 891 | ZINC000014721621 | 66.8805 | 4254 | ZINC000015207468 | 101.216 |
| 892 | ZINC000014722467 | 113.273 | 4255 | ZINC000015207473 | 96.1007 |
| 893 | ZINC000014724931 | 71.5594 | 4256 | ZINC000003869625 | 100.095 |
| 894 | ZINC000014727931 | 138.141 | 4257 | ZINC000001653215 | 77.2906 |
| 895 | ZINC000014758993 | 131.379 | 4258 | ZINC000001649427 | 105.911 |
| 896 | ZINC000014763050 | 111.593 | 4259 | ZINC000000404444 | 82.1612 |
| 897 | ZINC000014763050 | 115.915 | 4260 | ZINC000000405269 | 64.8503 |
| 898 | ZINC000014766825 | 103.093 | 4261 | ZINC000000406999 | 73.7507 |
| 899 | ZINC000005158016 | 64.1349 | 4262 | ZINC000000407074 | 60.4735 |
| 900 | ZINC000014719302 | 93.3231 | 4263 | ZINC000000409240 | 61.8003 |
| 901 | ZINC000014722312 | 121.501 | 4264 | ZINC000001638429 | 90.2359 |
| 902 | ZINC000014722471 | 112.941 | 4265 | ZINC000001641021 | 63.0133 |
| 903 | ZINC000014727630 | 138.914 | 4266 | ZINC000001641695 | 99.2657 |
| 904 | ZINC000014728045 | 139.529 | 4267 | ZINC000001648177 | 67.9462 |
| 905 | ZINC000014762985 | 115.493 | 4268 | ZINC000001648290 | 79.2585 |
| 906 | ZINC000004293351 | 125.223 | 4269 | ZINC000001653257 | 56.4122 |
| 907 | ZINC000014719976 | 114.732 | 4270 | ZINC000035241121 | 121.441 |
| 908 | ZINC000014722465 | 117.534 | 4271 | ZINC000000057291 | 121.076 |
| 909 | ZINC000014728401 | 128.888 | 4272 | ZINC000000057291 | 115.232 |
| 910 | ZINC000014762987 | 108.225 | 4273 | ZINC000000057740 | 91.4369 |
| 911 | ZINC000014763077 | 115.761 | 4274 | ZINC000001638430 | 86.5154 |
| 912 | ZINC000014719992 | 105.736 | 4275 | ZINC000001641024 | 77.7219 |
| 913 | ZINC000014722469 | 116.908 | 4276 | ZINC000001641696 | 86.4828 |
| 914 | ZINC000014726235 | 118.494 | 4277 | ZINC000001648227 | 80.3895 |
| 915 | ZINC000014728044 | 141.199 | 4278 | ZINC000000900254 | 102.989 |
| 916 | ZINC000014757021 | 113.264 | 4279 | ZINC000000901020 | 56.4366 |
| 917 | ZINC000014762498 | 87.044 | 4280 | ZINC000000001392 | 92.7945 |
| 918 | ZINC000014762971 | 115.518 | 4281 | ZINC000000058111 | 89.1327 |
| 919 | ZINC000014762971 | 118.639 | 4282 | ZINC000035645588 | 98.2362 |
| 920 | ZINC000004215039 | 91.4696 | 4283 | ZINC000013378210 | 140.162 |
| 921 | ZINC000004215527 | 112.536 | 4284 | ZINC000001081533 | 123.371 |
| 922 | ZINC000004252574 | 131.671 | 4285 | ZINC000003978794 | 116.61 |
| 923 | ZINC000014616871 | 86.7234 | 4286 | ZINC000030726283 | 87.4285 |
| 924 | ZINC000014642741 | 144.828 | 4287 | ZINC000003881797 | 101.458 |
| 925 | ZINC000014643684 | 136.933 | 4288 | ZINC000001531790 | 103.054 |
| 926 | ZINC000014652292 | 113.95 | 4289 | ZINC000001319796 | 123.811 |
| 927 | ZINC000014682854 | 110.108 | 4290 | ZINC000019535049 | 104.411 |
| 928 | ZINC000004775643 | 67.8758 | 4291 | ZINC000003979155 | 122.928 |
| 929 | ZINC000004896201 | 76.3537 | 4292 | ZINC000003197739 | 135.928 |
| 930 | ZINC000005029090 | 98.2308 | 4293 | ZINC000003197739 | 135.928 |
| 931 | ZINC000014642747 | 148.96 | 4294 | ZINC000000164388 | 86.2845 |
| 932 | ZINC000014682692 | 102.115 | 4295 | ZINC000000388661 | 132.507 |
| 933 | ZINC000014642744 | 147.301 | 4296 | ZINC000003881648 | 133.191 |
| 934 | ZINC000014642912 | 127.682 | 4297 | ZINC000004081043 | 105.39 |
| 935 | ZINC000014689152 | 125.253 | 4298 | ZINC000016952419 | 106.151 |
| 936 | ZINC000005157981 | 64.2906 | 4299 | ZINC000013513938 | 121.179 |
| 937 | ZINC000014642750 | 152.743 | 4300 | ZINC000013513938 | 116.275 |
| 938 | ZINC000014642994 | 131.253 | 4301 | ZINC000012496332 | 126.571 |
| 939 | ZINC000004095836 | 72.5116 | 4302 | ZINC000085489337 | 89.8014 |
| 940 | ZINC000004098746 | 135.396 | 4303 | ZINC000003847491 | 99.1257 |
| 941 | ZINC000014588996 | 88.3788 | 4304 | ZINC000001092752 | 74.4172 |
| 942 | ZINC000014589115 | 61.031 | 4305 | ZINC000000057645 | 136.424 |
| 943 | ZINC000014614428 | 81.8021 | 4306 | ZINC000000058081 | 119.723 |
| 944 | ZINC000003870120 | 76.5772 | 4307 | ZINC000000402228 | 109.312 |
| 945 | ZINC000004096018 | 88.7933 | 4308 | ZINC000001089845 | 116.889 |
| 946 | ZINC000004098319 | 131.111 | 4309 | ZINC000000389574 | 72.0729 |
| 947 | ZINC000014588993 | 87.8224 | 4310 | ZINC000022000224 | 69.8094 |
| 948 | ZINC000014589058 | 97.887 | 4311 | ZINC000021999701 | 93.3221 |
| 949 | ZINC000014589117 | 67.443 | 4312 | ZINC000006484607 | 127.588 |
| 950 | ZINC000014590536 | 73.0748 | 4313 | ZINC000006484607 | 139.539 |
| 951 | ZINC000014592828 | 61.4781 | 4314 | ZINC000022000199 | 61.8107 |
| 952 | ZINC000014610083 | 120.58 | 4315 | ZINC000003983883 | 120.87 |
| 953 | ZINC000014613037 | 99.3294 | 4316 | ZINC000003833823 | 112.073 |
| 954 | ZINC000014614332 | 99.6694 | 4317 | ZINC000000896722 | 73.7547 |
| 955 | ZINC000003845689 | 89.2079 | 4318 | ZINC000001849645 | 78.7065 |
| 956 | ZINC000003846059 | 84.7066 | 4319 | ZINC000001695462 | 105.007 |
| 957 | ZINC000003869370 | 99.8176 | 4320 | ZINC000001698513 | 47.1499 |
| 958 | ZINC000003869623 | 95.3392 | 4321 | ZINC000001693631 | 92.671 |
| 959 | ZINC000004095477 | 130.904 | 4322 | ZINC000001716732 | 87.7245 |
| 960 | ZINC000004095952 | 92.7968 | 4323 | ZINC000001718839 | 83.3919 |
| 961 | ZINC000004098273 | 116.374 | 4324 | ZINC000001719254 | 108.448 |
| 962 | ZINC000014589116 | 69.7344 | 4325 | ZINC000001720924 | 98.1029 |
| 963 | ZINC000014592827 | 62.5302 | 4326 | ZINC000003831617 | 98.2816 |
| 964 | ZINC000014610079 | 132.525 | 4327 | ZINC000029786458 | 64.3069 |
| 965 | ZINC000014614433 | 79.991 | 4328 | ZINC000029786460 | 65.5927 |
| 966 | ZINC000003875791 | 107.692 | 4329 | ZINC000029786461 | 64.7207 |
| 967 | ZINC000003978525 | 119.629 | 4330 | ZINC000000388088 | 60.8503 |
| 968 | ZINC000003980114 | 114.53 | 4331 | ZINC000001693894 | 105.049 |
| 969 | ZINC000004095812 | 110.543 | 4332 | ZINC000001694404 | 91.3565 |
| 970 | ZINC000004096806 | 91.6793 | 4333 | ZINC000001850534 | 90.8859 |
| 971 | ZINC000014588995 | 86.6312 | 4334 | ZINC000001850617 | 76.6678 |
| 972 | ZINC000014589059 | 98.8379 | 4335 | ZINC000029786463 | 61.7767 |
| 973 | ZINC000014589118 | 60.7014 | 4336 | ZINC000000388061 | 88.7293 |
| 974 | ZINC000014590677 | 75.299 | 4337 | ZINC000001718826 | 60.5722 |
| 975 | ZINC000003819459 | 137.693 | 4338 | ZINC000001719253 | 101.908 |
| 976 | ZINC000003819461 | 148.458 | 4339 | ZINC000001720167 | 105.059 |
| 977 | ZINC000003979037 | 111.02 | 4340 | ZINC000001712310 | 69.4519 |
| 978 | ZINC000004098221 | 111.835 | 4341 | ZINC000001649222 | 110.695 |
| 979 | ZINC000014495041 | 86.337 | 4342 | ZINC000013513592 | 108.247 |
| 980 | ZINC000014504307 | 111.027 | 4343 | ZINC000003925938 | 89.0717 |
| 981 | ZINC000014559784 | 98.0503 | 4344 | ZINC000003860469 | 92.0522 |
| 982 | ZINC000003947593 | 92.2153 | 4345 | ZINC000003833824 | 104.052 |
| 983 | ZINC000004095788 | 127.187 | 4346 | ZINC000003860151 | 110.422 |
| 984 | ZINC000004097621 | 86.0735 | 4347 | ZINC000001693386 | 66.4759 |
| 985 | ZINC000004098411 | 82.0479 | 4348 | ZINC000003860468 | 90.2212 |
| 986 | ZINC000014503461 | 147.167 | 4349 | ZINC000030726859 | 100.79 |
| 987 | ZINC000014504316 | 118.334 | 4350 | ZINC000000057689 | 127.907 |
| 988 | ZINC000014505068 | 146.835 | 4351 | ZINC000013512224 | 77.1217 |
| 989 | ZINC000014517001 | 91.3528 | 4352 | ZINC000004097027 | 82.9541 |
| 990 | ZINC000014587260 | 140.94 | 4353 | ZINC000004097377 | 88.9058 |
| 991 | ZINC000003846058 | 84.7127 | 4354 | ZINC000005640448 | 129.801 |
| 992 | ZINC000003846060 | 82.2581 | 4355 | ZINC000005641301 | 108.704 |
| 993 | ZINC000003869583 | 98.2898 | 4356 | ZINC000005641299 | 110.138 |
| 994 | ZINC000004095998 | 84.5629 | 4357 | ZINC000004096671 | 111.692 |
| 995 | ZINC000014491258 | 78.848 | 4358 | ZINC000013509425 | 122.788 |
| 996 | ZINC000014504310 | 95.9802 | 4359 | ZINC000013517387 | 135.947 |
| 997 | ZINC000014505065 | 144.502 | 4360 | ZINC000013660176 | 92.8607 |
| 998 | ZINC000014505663 | 86.8436 | 4361 | ZINC000014684428 | 56.6163 |
| 999 | ZINC000014587259 | 145.107 | 4362 | ZINC000000039091 | 144.053 |
| 1000 | ZINC000004096365 | 132.275 | 4363 | ZINC000000159590 | 87.8098 |
| 1001 | ZINC000014503833 | 137.465 | 4364 | ZINC000000164367 | 87.7889 |
| 1002 | ZINC000014504319 | 105.59 | 4365 | ZINC000002040185 | 71.2571 |
| 1003 | ZINC000003683317 | 90.442 | 4366 | ZINC000004175546 | 124.162 |
| 1004 | ZINC000002045861 | 62.4666 | 4367 | ZINC000002036135 | 89.8721 |
| 1005 | ZINC000003136353 | 126.059 | 4368 | ZINC000013827879 | 80.717 |
| 1006 | ZINC000013551392 | 83.5747 | 4369 | ZINC000014516984 | 78.5455 |
| 1007 | ZINC000014452355 | 102.849 | 4370 | ZINC000002039807 | 62.3367 |
| 1008 | ZINC000002516013 | 74.9638 | 4371 | ZINC000014455605 | 99.2471 |
| 1009 | ZINC000002518855 | 66.0198 | 4372 | ZINC000008737707 | 64.0852 |
| 1010 | ZINC000002534722 | 96.8816 | 4373 | ZINC000014616379 | 80.3369 |
| 1011 | ZINC000002561270 | 91.329 | 4374 | ZINC000014410367 | 132.453 |
| 1012 | ZINC000013547256 | 77.1347 | 4375 | ZINC000000001886 | 62.8183 |
| 1013 | ZINC000014455585 | 70.9015 | 4376 | ZINC000000057060 | 112.226 |
| 1014 | ZINC000001851053 | 102.579 | 4377 | ZINC000000001677 | 84.6265 |
| 1015 | ZINC000002009758 | 55.5469 | 4378 | ZINC000014438721 | 102.241 |
| 1016 | ZINC000002035755 | 79.0903 | 4379 | ZINC000014419577 | 97.1903 |
| 1017 | ZINC000002039275 | 97.4079 | 4380 | ZINC000002039876 | 85.5166 |
| 1018 | ZINC000002040464 | 88.2893 | 4381 | ZINC000014616890 | 90.4379 |
| 1019 | ZINC000003130515 | 78.9221 | 4382 | ZINC000014589062 | 103.365 |
| 1020 | ZINC000013543186 | 80.9751 | 4383 | ZINC000013520048 | 143.337 |
| 1021 | ZINC000013838494 | 120.563 | 4384 | ZINC000013520048 | 143.894 |
| 1022 | ZINC000002169060 | 87.5953 | 4385 | ZINC000014614333 | 83.5024 |
| 1023 | ZINC000002384652 | 109.921 | 4386 | ZINC000003824868 | 118.227 |
| 1024 | ZINC000002517155 | 73.3384 | 4387 | ZINC000013545798 | 122.948 |
| 1025 | ZINC000013551397 | 78.4868 | 4388 | ZINC000000164447 | 85.7731 |
| 1026 | ZINC000014455484 | 137.69 | 4389 | ZINC000000161294 | 75.6189 |
| 1027 | ZINC000001850808 | 112.507 | 4390 | ZINC000013781916 | 119.428 |
| 1028 | ZINC000001850991 | 108.369 | 4391 | ZINC000013829462 | 84.844 |
| 1029 | ZINC000002539248 | 88.878 | 4392 | ZINC000006069529 | 121.691 |
| 1030 | ZINC000002565773 | 133.013 | 4393 | ZINC000014684566 | 64.5734 |
| 1031 | ZINC000003473539 | 141.68 | 4394 | ZINC000006119222 | 65.4443 |
| 1032 | ZINC000013412803 | 78.2542 | 4395 | ZINC000002039894 | 70.5347 |
| 1033 | ZINC000013437574 | 81.4348 | 4396 | ZINC000000265517 | 94.7926 |
| 1034 | ZINC000013459854 | 69.0308 | 4397 | ZINC000003815418 | 128.612 |
| 1035 | ZINC000013481668 | 102.039 | 4398 | ZINC000004214983 | 102.339 |
| 1036 | ZINC000013509134 | 141.966 | 4399 | ZINC000004521773 | 89.0261 |
| 1037 | ZINC000013533861 | 98.7099 | 4400 | ZINC000004685854 | 85.1109 |
| 1038 | ZINC000001850993 | 111.501 | 4401 | ZINC000005116154 | 140.28 |
| 1039 | ZINC000002154253 | 104.232 | 4402 | ZINC000005116154 | 142.06 |
| 1040 | ZINC000002384576 | 114.664 | 4403 | ZINC000005140706 | 114.506 |
| 1041 | ZINC000002572528 | 135.176 | 4404 | ZINC000005764534 | 106.26 |
| 1042 | ZINC000002572528 | 134.758 | 4405 | ZINC000005923859 | 116.645 |
| 1043 | ZINC000003130514 | 75.6167 | 4406 | ZINC000018185774 | 142.178 |
| 1044 | ZINC000003641060 | 120.397 | 4407 | ZINC000018185774 | 142.286 |
| 1045 | ZINC000013514412 | 139.667 | 4408 | ZINC000021981235 | 134.107 |
| 1046 | ZINC000101705819 | 137.42 | 4409 | ZINC000095908875 | 105.194 |
| 1047 | ZINC000002004458 | 79.097 | 4410 | ZINC000002034481 | 88.048 |
| 1048 | ZINC000002035757 | 77.0377 | 4411 | ZINC000002509772 | 111.915 |
| 1049 | ZINC000002039864 | 94.7261 | 4412 | ZINC000003869580 | 105.145 |
| 1050 | ZINC000002040466 | 83.5724 | 4413 | ZINC000022061252 | 111.325 |
| 1051 | ZINC000002555390 | 84.1727 | 4414 | ZINC000000002137 | 94.3504 |
| 1052 | ZINC000002561261 | 115.45 | 4415 | ZINC000000006787 | 127.443 |
| 1053 | ZINC000002569310 | 79.8305 | 4416 | ZINC000000057731 | 101.443 |
| 1054 | ZINC000002585551 | 102.926 | 4417 | ZINC000000119983 | 130.784 |
| 1055 | ZINC000013456549 | 102.033 | 4418 | ZINC000000160790 | 74.3325 |
| 1056 | ZINC000100826366 | 89.5046 | 4419 | ZINC000000896098 | 77.4144 |
| 1057 | ZINC000002042998 | 87.215 | 4420 | ZINC000001081286 | 87.1152 |
| 1058 | ZINC000002383087 | 59.1379 | 4421 | ZINC000001481993 | 89.7993 |
| 1059 | ZINC000002508248 | 91.9465 | 4422 | ZINC000001591814 | 73.4958 |
| 1060 | ZINC000002518378 | 136.026 | 4423 | ZINC000001622034 | 104.097 |
| 1061 | ZINC000002534693 | 87.5242 | 4424 | ZINC000002018621 | 112.8 |
| 1062 | ZINC000003136351 | 132.566 | 4425 | ZINC000003794714 | 88.4329 |
| 1063 | ZINC000013412797 | 97.1381 | 4426 | ZINC000003831329 | 112.211 |
| 1064 | ZINC000013437571 | 80.3274 | 4427 | ZINC000003831331 | 107.471 |
| 1065 | ZINC000013481667 | 99.6056 | 4428 | ZINC000003995890 | 90.9104 |
| 1066 | ZINC000013484972 | 132.023 | 4429 | ZINC000013517187 | 90.7319 |
| 1067 | ZINC000013512028 | 96.6045 | 4430 | ZINC000013559434 | 140.125 |
| 1068 | ZINC000100780877 | 88.4813 | 4431 | ZINC000084397769 | 59.0582 |
| 1069 | ZINC000100823359 | 86.8062 | 4432 | ZINC000003814410 | 119.319 |
| 1070 | ZINC000000898959 | 133.658 | 4433 | ZINC000003861213 | 91.8655 |
| 1071 | ZINC000000898959 | 146.022 | 4434 | ZINC000003869576 | 96.0064 |
| 1072 | ZINC000001584046 | 102.145 | 4435 | ZINC000100034396 | 109.614 |
| 1073 | ZINC000001595734 | 110.243 | 4436 | ZINC000000066104 | 116.514 |
| 1074 | ZINC000001662779 | 139.335 | 4437 | ZINC000001847459 | 91.3082 |
| 1075 | ZINC000001761712 | 97.5373 | 4438 | ZINC000002041003 | 97.7877 |
| 1076 | ZINC000001850071 | 102.338 | 4439 | ZINC000002512178 | 105.995 |
| 1077 | ZINC000013322992 | 101.92 | 4440 | ZINC000002584392 | 99.1099 |
| 1078 | ZINC000013340360 | 111.069 | 4441 | ZINC000004099013 | 115.39 |
| 1079 | ZINC000013377483 | 116 | 4442 | ZINC000003860441 | 106.506 |
| 1080 | ZINC000013380322 | 77.0109 | 4443 | ZINC000091689892 | 116.608 |
| 1081 | ZINC000100053681 | 91.4889 | 4444 | ZINC000003874419 | 91.4258 |
| 1082 | ZINC000100776357 | 106.452 | 4445 | ZINC000003869640 | 113.89 |
| 1083 | ZINC000000899892 | 145.795 | 4446 | ZINC000100034211 | 97.7944 |
| 1084 | ZINC000001557676 | 118.86 | 4447 | ZINC000100043983 | 95.6412 |
| 1085 | ZINC000001594276 | 71.8042 | 4448 | ZINC000000968226 | 81.7664 |
| 1086 | ZINC000001694563 | 83.0853 | 4449 | ZINC000000121456 | 97.404 |
| 1087 | ZINC000001721694 | 139.646 | 4450 | ZINC000000895828 | 106.813 |
| 1088 | ZINC000013327493 | 130.297 | 4451 | ZINC000001640621 | 105.552 |
| 1089 | ZINC000013359966 | 126.857 | 4452 | ZINC000013517144 | 98.8621 |
| 1090 | ZINC000013359966 | 135.931 | 4453 | ZINC000018153302 | 78.4587 |
| 1091 | ZINC000013373993 | 116.276 | 4454 | ZINC000002040950 | 88.0218 |
| 1092 | ZINC000013373993 | 124.036 | 4455 | ZINC000100006441 | 99.4529 |
| 1093 | ZINC000013374378 | 93.5497 | 4456 | ZINC000002516902 | 119.936 |
| 1094 | ZINC000013382498 | 109.87 | 4457 | ZINC000002598081 | 99.4054 |
| 1095 | ZINC000100772443 | 84.4502 | 4458 | ZINC000003814360 | 124.907 |
| 1096 | ZINC000001069091 | 121.176 | 4459 | ZINC000003861538 | 87.8848 |
| 1097 | ZINC000001584048 | 102.623 | 4460 | ZINC000028631193 | 119.033 |
| 1098 | ZINC000001595958 | 106.193 | 4461 | ZINC000013831818 | 83.8847 |
| 1099 | ZINC000001632198 | 107.933 | 4462 | ZINC000000967513 | 82.7124 |
| 1100 | ZINC000001668225 | 104.214 | 4463 | ZINC000004658606 | 43.7571 |
| 1101 | ZINC000001718609 | 62.2298 | 4464 | ZINC000005116153 | 148.743 |
| 1102 | ZINC000001765597 | 79.4551 | 4465 | ZINC000005116153 | 152.57 |
| 1103 | ZINC000001850440 | 74.6013 | 4466 | ZINC000005140707 | 114.704 |
| 1104 | ZINC000013311063 | 102.656 | 4467 | ZINC000005239470 | 110.231 |
| 1105 | ZINC000013327491 | 136.517 | 4468 | ZINC000012494489 | 67.8627 |
| 1106 | ZINC000013374337 | 124.552 | 4469 | ZINC000005783565 | 91.0396 |
| 1107 | ZINC000013380324 | 87.9628 | 4470 | ZINC000084462581 | 92.8039 |
| 1108 | ZINC000013382497 | 112.94 | 4471 | ZINC000000057344 | 111.095 |
| 1109 | ZINC000085966361 | 131.106 | 4472 | ZINC000000391789 | 88.7077 |
| 1110 | ZINC000087528988 | 87.6639 | 4473 | ZINC000000901616 | 57.4382 |
| 1111 | ZINC000095618237 | 76.3333 | 4474 | ZINC000002379161 | 100.189 |
| 1112 | ZINC000095618241 | 98.3497 | 4475 | ZINC000002584391 | 91.7048 |
| 1113 | ZINC000095618243 | 98.1132 | 4476 | ZINC000003833800 | 90.2346 |
| 1114 | ZINC000095620542 | 88.3111 | 4477 | ZINC000003871017 | 126.556 |
| 1115 | ZINC000095620555 | 88.2048 | 4478 | ZINC000022061258 | 121.69 |
| 1116 | ZINC000001595732 | 112.175 | 4479 | ZINC000000073711 | 104.871 |
| 1117 | ZINC000001708726 | 89.4297 | 4480 | ZINC000000113418 | 116.924 |
| 1118 | ZINC000001747260 | 84.2923 | 4481 | ZINC000000896628 | 73.4022 |
| 1119 | ZINC000001849837 | 103.319 | 4482 | ZINC000001481970 | 54.9477 |
| 1120 | ZINC000013340359 | 110.567 | 4483 | ZINC000001530244 | 83.6954 |
| 1121 | ZINC000013340653 | 111.535 | 4484 | ZINC000001603364 | 81.6537 |
| 1122 | ZINC000013382499 | 110.059 | 4485 | ZINC000001622033 | 102.596 |
| 1123 | ZINC000085851429 | 113.434 | 4486 | ZINC000001641925 | 111.488 |
| 1124 | ZINC000085933459 | 136.722 | 4487 | ZINC000001697403 | 62.3592 |
| 1125 | ZINC000085947832 | 90.3276 | 4488 | ZINC000002018620 | 115.985 |
| 1126 | ZINC000086047315 | 87.3049 | 4489 | ZINC000003869608 | 131.782 |
| 1127 | ZINC000095617867 | 119.005 | 4490 | ZINC000003871891 | 103.83 |
| 1128 | ZINC000095617999 | 85.849 | 4491 | ZINC000004095859 | 111.253 |
| 1129 | ZINC000095618236 | 74.5045 | 4492 | ZINC000004098610 | 159.116 |
| 1130 | ZINC000095618242 | 98.5767 | 4493 | ZINC000012402948 | 103.481 |
| 1131 | ZINC000095618244 | 95.4045 | 4494 | ZINC000003995861 | 88.5616 |
| 1132 | ZINC000095620556 | 84.7131 | 4495 | ZINC000003871576 | 138.62 |
| 1133 | ZINC000000899085 | 90.7901 | 4496 | ZINC000003871576 | 137 |
| 1134 | ZINC000000899315 | 111.557 | 4497 | ZINC000002077807 | 103.567 |
| 1135 | ZINC000000899786 | 114.633 | 4498 | ZINC000000155911 | 102.172 |
| 1136 | ZINC000001584047 | 103.097 | 4499 | ZINC000000402766 | 98.3636 |
| 1137 | ZINC000001615142 | 133.307 | 4500 | ZINC000000898237 | 139.644 |
| 1138 | ZINC000001663391 | 130.351 | 4501 | ZINC000000001217 | 95.8187 |
| 1139 | ZINC000001850073 | 104.255 | 4502 | ZINC000031163472 | 87.9144 |
| 1140 | ZINC000012496358 | 128.232 | 4503 | ZINC000000032312 | 75.3683 |
| 1141 | ZINC000012496764 | 82.4125 | 4504 | ZINC000004293904 | 68.2185 |
| 1142 | ZINC000013125765 | 121.269 | 4505 | ZINC000004292977 | 89.8809 |
| 1143 | ZINC000013125765 | 117.636 | 4506 | ZINC000002003388 | 82.5144 |
| 1144 | ZINC000013306848 | 71.6444 | 4507 | ZINC000002014874 | 55.6561 |
| 1145 | ZINC000083260318 | 89.1866 | 4508 | ZINC000001531529 | 107.956 |
| 1146 | ZINC000001319752 | 128.038 | 4509 | ZINC000001531537 | 93.3829 |
| 1147 | ZINC000001557677 | 128.285 | 4510 | ZINC000001608718 | 105.019 |
| 1148 | ZINC000001595731 | 115.711 | 4511 | ZINC000000895930 | 78.2461 |
| 1149 | ZINC000001724067 | 92.1249 | 4512 | ZINC000000896000 | 90.1556 |
| 1150 | ZINC000012496248 | 94.3113 | 4513 | ZINC000000896626 | 81.2999 |
| 1151 | ZINC000013108877 | 151.756 | 4514 | ZINC000014586981 | 110.4 |
| 1152 | ZINC000013132552 | 118.212 | 4515 | ZINC000014650833 | 113.447 |
| 1153 | ZINC000083260317 | 88.0223 | 4516 | ZINC000015122022 | 136.105 |
| 1154 | ZINC000085491763 | 106.911 | 4517 | ZINC000014438705 | 91.9764 |
| 1155 | ZINC000000899905 | 139.969 | 4518 | ZINC000015849045 | 66.7382 |
| 1156 | ZINC000001587150 | 107.598 | 4519 | ZINC000016697731 | 94.2006 |
| 1157 | ZINC000001596396 | 60.498 | 4520 | ZINC000016997539 | 98.2065 |
| 1158 | ZINC000001596396 | 62.2342 | 4521 | ZINC000017877781 | 107.601 |
| 1159 | ZINC000001634347 | 147.372 | 4522 | ZINC000018258326 | 160.061 |
| 1160 | ZINC000001685818 | 95.329 | 4523 | ZINC000018847036 | 133.788 |
| 1161 | ZINC000001721693 | 135.857 | 4524 | ZINC000018847044 | 136.048 |
| 1162 | ZINC000001846561 | 52.9331 | 4525 | ZINC000018847044 | 134.204 |
| 1163 | ZINC000001850495 | 102.341 | 4526 | ZINC000019074717 | 86.8864 |
| 1164 | ZINC000012496774 | 100.883 | 4527 | ZINC000019418976 | 92.2702 |
| 1165 | ZINC000013130921 | 122.332 | 4528 | ZINC000019877642 | 65.4806 |
| 1166 | ZINC000013307187 | 120.198 | 4529 | ZINC000021984838 | 72.0005 |
| 1167 | ZINC000062233814 | 107.793 | 4530 | ZINC000021999678 | 86.0683 |
| 1168 | ZINC000064634147 | 100.353 | 4531 | ZINC000021999687 | 84.8714 |
| 1169 | ZINC000067903300 | 130.992 | 4532 | ZINC000013350968 | 85.809 |
| 1170 | ZINC000070595319 | 77.5649 | 4533 | ZINC000014414926 | 109.392 |
| 1171 | ZINC000001584045 | 100.847 | 4534 | ZINC000014614334 | 85.5502 |
| 1172 | ZINC000001595733 | 111.341 | 4535 | ZINC000015251791 | 113.758 |
| 1173 | ZINC000001650575 | 108.145 | 4536 | ZINC000015251791 | 123.163 |
| 1174 | ZINC000001747853 | 83.261 | 4537 | ZINC000014612455 | 92.5105 |
| 1175 | ZINC000001850046 | 85.5016 | 4538 | ZINC000014614335 | 100.918 |
| 1176 | ZINC000013305131 | 94.0665 | 4539 | ZINC000014593990 | 86.6756 |
| 1177 | ZINC000059586886 | 103.488 | 4540 | ZINC000015204734 | 96.3406 |
| 1178 | ZINC000070451064 | 102.028 | 4541 | ZINC000014018383 | 116.66 |
| 1179 | ZINC000075518672 | 69.6541 | 4542 | ZINC000014593122 | 91.2497 |
| 1180 | ZINC000002018562 | 76.25 | 4543 | ZINC000013341252 | 114.424 |
| 1181 | ZINC000002038734 | 83.0635 | 4544 | ZINC000014018380 | 116.198 |
| 1182 | ZINC000000895662 | 119.672 | 4545 | ZINC000014592258 | 84.8016 |
| 1183 | ZINC000006524916 | 113.316 | 4546 | ZINC000015119988 | 77.218 |
| 1184 | ZINC000008220462 | 89.6942 | 4547 | ZINC000013860547 | 116.981 |
| 1185 | ZINC000012153161 | 97.4753 | 4548 | ZINC000014590710 | 69.0277 |
| 1186 | ZINC000044387250 | 107.522 | 4549 | ZINC000015113209 | 99.8408 |
| 1187 | ZINC000002014846 | 69.467 | 4550 | ZINC000014588433 | 90.6235 |
| 1188 | ZINC000002039413 | 86.0936 | 4551 | ZINC000014588966 | 109.734 |
| 1189 | ZINC000000517458 | 135.305 | 4552 | ZINC000014589057 | 86.2114 |
| 1190 | ZINC000000517458 | 133.93 | 4553 | ZINC000014589099 | 81.7175 |
| 1191 | ZINC000006091898 | 108.265 | 4554 | ZINC000013306845 | 78.0455 |
| 1192 | ZINC000006092566 | 123.746 | 4555 | ZINC000013515185 | 140.732 |
| 1193 | ZINC000006500050 | 123.994 | 4556 | ZINC000014588434 | 93.1179 |
| 1194 | ZINC000006525252 | 131.17 | 4557 | ZINC000014588973 | 86.3562 |
| 1195 | ZINC000007998027 | 122.3 | 4558 | ZINC000014589080 | 95.7312 |
| 1196 | ZINC000008214562 | 124.385 | 4559 | ZINC000014589100 | 63.9907 |
| 1197 | ZINC000012153650 | 103.424 | 4560 | ZINC000013540500 | 101.134 |
| 1198 | ZINC000044387247 | 126.03 | 4561 | ZINC000014953674 | 102.706 |
| 1199 | ZINC000044404288 | 125.409 | 4562 | ZINC000013542495 | 79.4288 |
| 1200 | ZINC000002039411 | 86.5214 | 4563 | ZINC000013481200 | 107.071 |
| 1201 | ZINC000006094124 | 144.931 | 4564 | ZINC000014557894 | 137.722 |
| 1202 | ZINC000006525249 | 133.586 | 4565 | ZINC000014859994 | 106.49 |
| 1203 | ZINC000006525249 | 132.216 | 4566 | ZINC000013460359 | 83.8422 |
| 1204 | ZINC000007974994 | 120.359 | 4567 | ZINC000014559789 | 96.6087 |
| 1205 | ZINC000008612885 | 71.8892 | 4568 | ZINC000014823208 | 87.6081 |
| 1206 | ZINC000012153223 | 85.718 | 4569 | ZINC000014820584 | 125.501 |
| 1207 | ZINC000038458574 | 118.509 | 4570 | ZINC000014820588 | 118.434 |
| 1208 | ZINC000039118427 | 65.3064 | 4571 | ZINC000014823206 | 87.6311 |
| 1209 | ZINC000001849664 | 101.428 | 4572 | ZINC000013481657 | 96.0017 |
| 1210 | ZINC000001850337 | 93.8488 | 4573 | ZINC000014504899 | 117.49 |
| 1211 | ZINC000000265501 | 119.353 | 4574 | ZINC000014813714 | 143.528 |
| 1212 | ZINC000000895551 | 134.563 | 4575 | ZINC000014813714 | 143.528 |
| 1213 | ZINC000006092209 | 137.863 | 4576 | ZINC000014814061 | 74.1762 |
| 1214 | ZINC000006092209 | 138.47 | 4577 | ZINC000013437580 | 87.8327 |
| 1215 | ZINC000006119185 | 91.4371 | 4578 | ZINC000013437567 | 79.802 |
| 1216 | ZINC000006119185 | 94.6877 | 4579 | ZINC000013437584 | 92.4088 |
| 1217 | ZINC000008234296 | 105.449 | 4580 | ZINC000014763011 | 138.916 |
| 1218 | ZINC000011680913 | 142.452 | 4581 | ZINC000014766021 | 99.4535 |
| 1219 | ZINC000038635145 | 107.222 | 4582 | ZINC000014774634 | 142.876 |
| 1220 | ZINC000038645381 | 86.5334 | 4583 | ZINC000014760151 | 134.619 |
| 1221 | ZINC000040164531 | 128.854 | 4584 | ZINC000014760155 | 143.5 |
| 1222 | ZINC000000156831 | 146.191 | 4585 | ZINC000014762761 | 136.706 |
| 1223 | ZINC000230070585 | 119.334 | 4586 | ZINC000014728402 | 133.418 |
| 1224 | ZINC000059589136 | 85.3463 | 4587 | ZINC000014489904 | 90.8029 |
| 1225 | ZINC000056874285 | 92.314 | 4588 | ZINC000014722381 | 120.802 |
| 1226 | ZINC000071404509 | 91.8928 | 4589 | ZINC000014723426 | 66.8143 |
| 1227 | ZINC000056874751 | 86.2682 | 4590 | ZINC000014724330 | 60.2956 |
| 1228 | ZINC000056875062 | 107.335 | 4591 | ZINC000014724334 | 61.8083 |
| 1229 | ZINC000003833872 | 110.994 | 4592 | ZINC000013385871 | 78.1689 |
| 1230 | ZINC000003833952 | 110.436 | 4593 | ZINC000014488928 | 88.1558 |
| 1231 | ZINC000000895837 | 110.904 | 4594 | ZINC000014489899 | 95.7426 |
| 1232 | ZINC000000388251 | 70.954 | 4595 | ZINC000014713558 | 99.7438 |
| 1233 | ZINC000000388318 | 94.3362 | 4596 | ZINC000014651626 | 101.313 |
| 1234 | ZINC000005766995 | 72.8276 | 4597 | ZINC000014651635 | 135.584 |
| 1235 | ZINC000005764527 | 121.447 | 4598 | ZINC000014651769 | 109.554 |
| 1236 | ZINC000000038028 | 87.0614 | 4599 | ZINC000014652219 | 101.746 |
| 1237 | ZINC000004245632 | 133.666 | 4600 | ZINC000014652228 | 101.283 |
| 1238 | ZINC000000050188 | 105.002 | 4601 | ZINC000013376124 | 76.3199 |
| 1239 | ZINC000000001360 | 138.469 | 4602 | ZINC000014445238 | 118.229 |
| 1240 | ZINC000002043392 | 83.3472 | 4603 | ZINC000014651624 | 105.718 |
| 1241 | ZINC000002043392 | 88.9554 | 4604 | ZINC000014651629 | 101.6 |
| 1242 | ZINC000017993401 | 112.298 | 4605 | ZINC000014651766 | 110.41 |
| 1243 | ZINC000017993401 | 113.754 | 4606 | ZINC000014652210 | 80.9131 |
| 1244 | ZINC000000388699 | 65.3188 | 4607 | ZINC000014652225 | 86.6214 |
| 1245 | ZINC000001712794 | 88.164 | 4608 | ZINC000014652231 | 117.673 |
| 1246 | ZINC000002558134 | 115.213 | 4609 | ZINC000014438727 | 87.5121 |
| 1247 | ZINC000002560604 | 100.945 | 4610 | ZINC000014645298 | 95.292 |
| 1248 | ZINC000000895826 | 79.4182 | 4611 | ZINC000014438522 | 85.83 |
| 1249 | ZINC000005735816 | 81.6354 | 4612 | ZINC000014438703 | 88.2083 |
| 1250 | ZINC000005761214 | 97.7321 | 4613 | ZINC000014438701 | 78.6755 |
| 1251 | ZINC000000132915 | 93.8125 | 4614 | ZINC000014642687 | 120.794 |
| 1252 | ZINC000005224354 | 40.0888 | 4615 | ZINC000022115627 | 81.1691 |
| 1253 | ZINC000001529846 | 103.652 | 4616 | ZINC000025695480 | 52.7263 |
| 1254 | ZINC000002597049 | 93.5245 | 4617 | ZINC000025695484 | 53.0722 |
| 1255 | ZINC000000014036 | 127.357 | 4618 | ZINC000025720799 | 80.6771 |
| 1256 | ZINC000005829553 | 85.5266 | 4619 | ZINC000025725630 | 90.1567 |
| 1257 | ZINC000005732865 | 87.3721 | 4620 | ZINC000025757012 | 72.6196 |
| 1258 | ZINC000005732867 | 104.257 | 4621 | ZINC000025757016 | 60.851 |
| 1259 | ZINC000005766919 | 95.7347 | 4622 | ZINC000018037965 | 153.139 |
| 1260 | ZINC000017175432 | 115.936 | 4623 | ZINC000018847037 | 143.833 |
| 1261 | ZINC000001687322 | 110.177 | 4624 | ZINC000018847037 | 138.963 |
| 1262 | ZINC000001690306 | 100.426 | 4625 | ZINC000018847051 | 130.679 |
| 1263 | ZINC000001691303 | 84.9185 | 4626 | ZINC000019735106 | 68.6544 |
| 1264 | ZINC000001693211 | 85.8129 | 4627 | ZINC000019877638 | 64.4303 |
| 1265 | ZINC000001693593 | 91.5088 | 4628 | ZINC000021984837 | 73.3638 |
| 1266 | ZINC000005308209 | 71.6007 | 4629 | ZINC000021999673 | 83.6054 |
| 1267 | ZINC000002504349 | 91.1128 | 4630 | ZINC000021999682 | 82.9439 |
| 1268 | ZINC000005138833 | 76.1584 | 4631 | ZINC000006031048 | 83.0803 |
| 1269 | ZINC000004245654 | 98.7451 | 4632 | ZINC000006031296 | 61.6455 |
| 1270 | ZINC000001081109 | 83.1888 | 4633 | ZINC000013346770 | 99.6703 |
| 1271 | ZINC000004825506 | 75.6621 | 4634 | ZINC000014616889 | 102.202 |
| 1272 | ZINC000005225098 | 67.8006 | 4635 | ZINC000014614316 | 111.414 |
| 1273 | ZINC000001568212 | 122.571 | 4636 | ZINC000013322244 | 125.236 |
| 1274 | ZINC000003814431 | 119.579 | 4637 | ZINC000013570997 | 115.15 |
| 1275 | ZINC000000895256 | 38.3724 | 4638 | ZINC000014593123 | 91.3759 |
| 1276 | ZINC000001850694 | 66.8074 | 4639 | ZINC000015169356 | 80.864 |
| 1277 | ZINC000001850956 | 54.9586 | 4640 | ZINC000015204735 | 91.7588 |
| 1278 | ZINC000001851036 | 90.1588 | 4641 | ZINC000013328754 | 119.347 |
| 1279 | ZINC000003593622 | 45.3938 | 4642 | ZINC000014592814 | 72.9216 |
| 1280 | ZINC000002584370 | 86.104 | 4643 | ZINC000015116102 | 91.689 |
| 1281 | ZINC000001687357 | 116.959 | 4644 | ZINC000015119659 | 66.253 |
| 1282 | ZINC000001690417 | 65.2362 | 4645 | ZINC000015119987 | 76.5889 |
| 1283 | ZINC000001691363 | 61.9196 | 4646 | ZINC000013334938 | 129.158 |
| 1284 | ZINC000001693270 | 87.961 | 4647 | ZINC000013704159 | 102.326 |
| 1285 | ZINC000001693594 | 92.8322 | 4648 | ZINC000015113207 | 104.281 |
| 1286 | ZINC000002575506 | 72.2238 | 4649 | ZINC000013481840 | 124.126 |
| 1287 | ZINC000001559692 | 93.2142 | 4650 | ZINC000013542496 | 78.6263 |
| 1288 | ZINC000003814366 | 126.597 | 4651 | ZINC000014588443 | 91.228 |
| 1289 | ZINC000003815415 | 125.469 | 4652 | ZINC000014588974 | 87.4801 |
| 1290 | ZINC000002528295 | 76.1385 | 4653 | ZINC000014589097 | 56.9022 |
| 1291 | ZINC000002534775 | 110.599 | 4654 | ZINC000014589773 | 102.715 |
| 1292 | ZINC000000895183 | 79.589 | 4655 | ZINC000013533343 | 108.101 |
| 1293 | ZINC000002504418 | 76.6038 | 4656 | ZINC000014588402 | 86.2432 |
| 1294 | ZINC000002510291 | 70.5723 | 4657 | ZINC000014589056 | 87.086 |
| 1295 | ZINC000095620480 | 122.112 | 4658 | ZINC000014589098 | 72.468 |
| 1296 | ZINC000085599508 | 107.784 | 4659 | ZINC000014589803 | 108.046 |
| 1297 | ZINC000085564529 | 104.106 | 4660 | ZINC000013118172 | 98.0872 |
| 1298 | ZINC000082304985 | 117.427 | 4661 | ZINC000013481841 | 119.28 |
| 1299 | ZINC000082304985 | 119.952 | 4662 | ZINC000013542723 | 63.652 |
| 1300 | ZINC000085489252 | 80.8036 | 4663 | ZINC000014959367 | 126.466 |
| 1301 | ZINC000000394284 | 105.425 | 4664 | ZINC000013481851 | 97.5247 |
| 1302 | ZINC000005416353 | 103.847 | 4665 | ZINC000013508504 | 90.5477 |
| 1303 | ZINC000005766692 | 75.4007 | 4666 | ZINC000013540496 | 102.989 |
| 1304 | ZINC000005817054 | 130.969 | 4667 | ZINC000013460027 | 88.5988 |
| 1305 | ZINC000005999212 | 88.2848 | 4668 | ZINC000014859992 | 116.396 |
| 1306 | ZINC000005732368 | 124.802 | 4669 | ZINC000014860574 | 73.055 |
| 1307 | ZINC000005732368 | 129.93 | 4670 | ZINC000014887408 | 129.829 |
| 1308 | ZINC000005734356 | 121.71 | 4671 | ZINC000013108863 | 128.942 |
| 1309 | ZINC000004096634 | 123.477 | 4672 | ZINC000013306796 | 69.5533 |
| 1310 | ZINC000003869683 | 113.994 | 4673 | ZINC000013460734 | 100.009 |
| 1311 | ZINC000004096696 | 93.5196 | 4674 | ZINC000014557891 | 127.9 |
| 1312 | ZINC000004097019 | 53.562 | 4675 | ZINC000014557980 | 101.189 |
| 1313 | ZINC000004097767 | 90.1252 | 4676 | ZINC000014824027 | 155.782 |
| 1314 | ZINC000004097902 | 48.5547 | 4677 | ZINC000014507030 | 90.128 |
| 1315 | ZINC000004098123 | 101.566 | 4678 | ZINC000014820583 | 136.983 |
| 1316 | ZINC000004654635 | 116.537 | 4679 | ZINC000014820586 | 129.364 |
| 1317 | ZINC000004654636 | 112.6 | 4680 | ZINC000014821367 | 91.2939 |
| 1318 | ZINC000004654841 | 116.682 | 4681 | ZINC000013481340 | 128.704 |
| 1319 | ZINC000004654842 | 112.109 | 4682 | ZINC000014507033 | 90.6661 |
| 1320 | ZINC000004658604 | 46.7256 | 4683 | ZINC000014813434 | 95.7948 |
| 1321 | ZINC000004705638 | 117.455 | 4684 | ZINC000014815122 | 126.659 |
| 1322 | ZINC000004991433 | 107.159 | 4685 | ZINC000014815122 | 121.957 |
| 1323 | ZINC000004991439 | 105.906 | 4686 | ZINC000014503848 | 114.41 |
| 1324 | ZINC000005183072 | 72.879 | 4687 | ZINC000014764651 | 60.4715 |
| 1325 | ZINC000005224268 | 87.9418 | 4688 | ZINC000014768312 | 135.774 |
| 1326 | ZINC000005273745 | 99.7326 | 4689 | ZINC000014774720 | 79.1328 |
| 1327 | ZINC000005273746 | 96.392 | 4690 | ZINC000014491260 | 75.9082 |
| 1328 | ZINC000005273747 | 94.4551 | 4691 | ZINC000014760153 | 138.227 |
| 1329 | ZINC000005273947 | 107.764 | 4692 | ZINC000013412523 | 86.6409 |
| 1330 | ZINC000005298876 | 53.048 | 4693 | ZINC000013437578 | 88.5184 |
| 1331 | ZINC000005298879 | 52.9467 | 4694 | ZINC000014496278 | 101.741 |
| 1332 | ZINC000005360178 | 109.249 | 4695 | ZINC000014727929 | 137.149 |
| 1333 | ZINC000005845755 | 141.823 | 4696 | ZINC000014488930 | 85.5554 |
| 1334 | ZINC000005999029 | 126.86 | 4697 | ZINC000014489316 | 111.177 |
| 1335 | ZINC000006020524 | 102.058 | 4698 | ZINC000014489901 | 95.9787 |
| 1336 | ZINC000006032266 | 77.9004 | 4699 | ZINC000014724332 | 58.4129 |
| 1337 | ZINC000034800312 | 87.8796 | 4700 | ZINC000014724336 | 62.0685 |
| 1338 | ZINC000005425259 | 107.169 | 4701 | ZINC000014489903 | 94.565 |
| 1339 | ZINC000005425259 | 111.876 | 4702 | ZINC000013397394 | 94.3227 |
| 1340 | ZINC000005457567 | 115.881 | 4703 | ZINC000014449163 | 75.3735 |
| 1341 | ZINC000005669265 | 148.249 | 4704 | ZINC000014455588 | 103.929 |
| 1342 | ZINC000005688625 | 79.3898 | 4705 | ZINC000014455587 | 94.7174 |
| 1343 | ZINC000005732760 | 81.6219 | 4706 | ZINC000008622375 | 83.1111 |
| 1344 | ZINC000005736030 | 62.8045 | 4707 | ZINC000013379107 | 108.739 |
| 1345 | ZINC000005736100 | 61.1994 | 4708 | ZINC000014447816 | 95.023 |
| 1346 | ZINC000005751251 | 90.2341 | 4709 | ZINC000014448311 | 75.4271 |
| 1347 | ZINC000005752238 | 62.2548 | 4710 | ZINC000014679957 | 61.8974 |
| 1348 | ZINC000005757941 | 110.936 | 4711 | ZINC000014680002 | 83.8919 |
| 1349 | ZINC000005761194 | 81.5106 | 4712 | ZINC000014680083 | 84.4778 |
| 1350 | ZINC000005764434 | 98.9487 | 4713 | ZINC000014448051 | 91.2102 |
| 1351 | ZINC000005765013 | 53.485 | 4714 | ZINC000014680038 | 92.7617 |
| 1352 | ZINC000005767267 | 116.267 | 4715 | ZINC000003871633 | 132.056 |
| 1353 | ZINC000005767672 | 84.641 | 4716 | ZINC000003871633 | 135.324 |
| 1354 | ZINC000005783606 | 100.353 | 4717 | ZINC000002585546 | 107.996 |
| 1355 | ZINC000004693574 | 145.756 | 4718 | ZINC000001846592 | 124.772 |
| 1356 | ZINC000004693574 | 144.58 | 4719 | ZINC000000002005 | 76.0412 |
| 1357 | ZINC000086040406 | 66.3179 | 4720 | ZINC000005133378 | 70.0457 |
| 1358 | ZINC000005133329 | 63.6564 | 4721 | ZINC000003635934 | 109.696 |
| 1359 | ZINC000118912393 | 123.603 | 4722 | ZINC000001586329 | 99.3788 |
| 1360 | ZINC000000000490 | 90.6118 | 4723 | ZINC000005037432 | 108.014 |
| 1361 | ZINC000000001706 | 80.1986 | 4724 | ZINC000005998696 | 131.443 |
| 1362 | ZINC000000002028 | 71.1389 | 4725 | ZINC000005999028 | 144.108 |
| 1363 | ZINC000000896409 | 76.5776 | 4726 | ZINC000001713168 | 140.569 |
| 1364 | ZINC000001576892 | 110.067 | 4727 | ZINC000005457778 | 78.0106 |
| 1365 | ZINC000002561203 | 104.641 | 4728 | ZINC000003861573 | 80.8614 |
| 1366 | ZINC000008214514 | 39.3568 | 4729 | ZINC000000897978 | 89.0037 |
| 1367 | ZINC000005733652 | 147.971 | 4730 | ZINC000000898819 | 85.0231 |
| 1368 | ZINC000004228258 | 118.218 | 4731 | ZINC000000898913 | 92.0545 |
| 1369 | ZINC000000405113 | 74.524 | 4732 | ZINC000000899137 | 96.5069 |
| 1370 | ZINC000005452635 | 110.094 | 4733 | ZINC000000899550 | 105.409 |
| 1371 | ZINC000005554720 | 145.067 | 4734 | ZINC000000900237 | 115.692 |
| 1372 | ZINC000005699918 | 105.385 | 4735 | ZINC000000900861 | 117.641 |
| 1373 | ZINC000005037433 | 90.4068 | 4736 | ZINC000002386261 | 99.2731 |
| 1374 | ZINC000005045477 | 115.494 | 4737 | ZINC000070454841 | 117.118 |
| 1375 | ZINC000005282655 | 114.029 | 4738 | ZINC000000157462 | 86.2306 |
| 1376 | ZINC000005357388 | 110.328 | 4739 | ZINC000095098822 | 97.3943 |
| 1377 | ZINC000000006556 | 103.643 | 4740 | ZINC000014684885 | 99.4098 |
| 1378 | ZINC000000043478 | 106.079 | 4741 | ZINC000014762510 | 132.344 |
| 1379 | ZINC000003612845 | 103.955 | 4742 | ZINC000000018056 | 123.995 |
| 1380 | ZINC000004102315 | 111.43 | 4743 | ZINC000014442973 | 113.064 |
| 1381 | ZINC000030731193 | 111.941 | 4744 | ZINC000014591289 | 102.386 |
| 1382 | ZINC000030731196 | 105.724 | 4745 | ZINC000008234282 | 91.7008 |
| 1383 | ZINC000000012342 | 153.013 | 4746 | ZINC000005759263 | 122.315 |
| 1384 | ZINC000000895271 | 70.9442 | 4747 | ZINC000005765166 | 115.832 |
| 1385 | ZINC000094437874 | 63.9916 | 4748 | ZINC000005973246 | 124.244 |
| 1386 | ZINC000004097791 | 76.9196 | 4749 | ZINC000000897928 | 134.436 |
| 1387 | ZINC000004097883 | 67.8329 | 4750 | ZINC000001531610 | 101.556 |
| 1388 | ZINC000004098032 | 106.963 | 4751 | ZINC000001599076 | 81.2744 |
| 1389 | ZINC000004098132 | 107.426 | 4752 | ZINC000002039760 | 106.635 |
| 1390 | ZINC000004102368 | 137.611 | 4753 | ZINC000003473153 | 132.918 |
| 1391 | ZINC000004102401 | 112.484 | 4754 | ZINC000003641076 | 85.2887 |
| 1392 | ZINC000003978347 | 60.6875 | 4755 | ZINC000004097838 | 108.584 |
| 1393 | ZINC000004097919 | 83.9008 | 4756 | ZINC000004098210 | 94.0127 |
| 1394 | ZINC000004098004 | 145.929 | 4757 | ZINC000004098841 | 60.5144 |
| 1395 | ZINC000004098045 | 99.1456 | 4758 | ZINC000000898291 | 120.368 |
| 1396 | ZINC000004098129 | 114.2 | 4759 | ZINC000001531097 | 114.034 |
| 1397 | ZINC000004098466 | 156.333 | 4760 | ZINC000001620487 | 113.042 |
| 1398 | ZINC000004098649 | 98.5176 | 4761 | ZINC000001729384 | 100.399 |
| 1399 | ZINC000000156974 | 96.0764 | 4762 | ZINC000002083320 | 88.5299 |
| 1400 | ZINC000033831984 | 95.3772 | 4763 | ZINC000003200778 | 98.156 |
| 1401 | ZINC000013520815 | 128.276 | 4764 | ZINC000015114975 | 137.658 |
| 1402 | ZINC000033842420 | 103.511 | 4765 | ZINC000003875576 | 94.2934 |
| 1403 | ZINC000034239698 | 112.094 | 4766 | ZINC000003982481 | 86.4146 |
| 1404 | ZINC000008855117 | 126.183 | 4767 | ZINC000004098262 | 104.965 |
| 1405 | ZINC000000898752 | 100.759 | 4768 | ZINC000012405001 | 88.6088 |
| 1406 | ZINC000000900158 | 137.933 | 4769 | ZINC000004026171 | 108.371 |
| 1407 | ZINC000003978779 | 128.221 | 4770 | ZINC000005277130 | 102.819 |
| 1408 | ZINC000003978568 | 113.906 | 4771 | ZINC000006069584 | 114.35 |
| 1409 | ZINC000004013299 | 126.18 | 4772 | ZINC000006069584 | 118.832 |
| 1410 | ZINC000002548243 | 121.963 | 4773 | ZINC000014646005 | 116.942 |
| 1411 | ZINC000003992526 | 98.5907 | 4774 | ZINC000014444870 | 125.938 |
| 1412 | ZINC000003909323 | 93.3013 | 4775 | ZINC000014681313 | 95.8324 |
| 1413 | ZINC000003898853 | 118.146 | 4776 | ZINC000014760938 | 115.567 |
| 1414 | ZINC000001846515 | 94.9703 | 4777 | ZINC000015274386 | 83.7465 |
| 1415 | ZINC000014681569 | 110.416 | 4778 | ZINC000003881905 | 91.5639 |
| 1416 | ZINC000014696109 | 121.285 | 4779 | ZINC000003882003 | 102.619 |
| 1417 | ZINC000014679257 | 95.1773 | 4780 | ZINC000002041733 | 93.2986 |
| 1418 | ZINC000014618143 | 101.398 | 4781 | ZINC000002047514 | 88.5097 |
| 1419 | ZINC000014585208 | 136.184 | 4782 | ZINC000000120286 | 124.103 |
| 1420 | ZINC000014558326 | 167.389 | 4783 | ZINC000000008492 | 91.3159 |
| 1421 | ZINC000014492390 | 97.8027 | 4784 | ZINC000000896546 | 78.142 |
| 1422 | ZINC000000006256 | 108.869 | 4785 | ZINC000004658290 | 80.9661 |
| 1423 | ZINC000013482860 | 94.2851 | 4786 | ZINC000004658290 | 75.9509 |
| 1424 | ZINC000013484812 | 128.008 | 4787 | ZINC000095616601 | 149.111 |
| 1425 | ZINC000013484812 | 138.213 | 4788 | ZINC000001633887 | 131.796 |
| 1426 | ZINC000013377898 | 124.527 | 4789 | ZINC000002548959 | 99.3859 |
| 1427 | ZINC000013384148 | 109.924 | 4790 | ZINC000000389747 | 127.906 |
| 1428 | ZINC000013384147 | 114.71 | 4791 | ZINC000003812897 | 141.339 |
| 1429 | ZINC000013436958 | 151.957 | 4792 | ZINC000000967520 | 63.773 |
| 1430 | ZINC000013436958 | 145.796 | 4793 | ZINC000001633889 | 131.982 |
| 1431 | ZINC000013335261 | 124.821 | 4794 | ZINC000004228257 | 116.373 |
| 1432 | ZINC000013341107 | 118.884 | 4795 | ZINC000004228257 | 110.211 |
| 1433 | ZINC000013340225 | 129.232 | 4796 | ZINC000001530303 | 103.158 |
| 1434 | ZINC000000900216 | 93.542 | 4797 | ZINC000003807917 | 119.504 |
| 1435 | ZINC000000898052 | 102.654 | 4798 | ZINC000013585233 | 118.082 |
| 1436 | ZINC000000899141 | 134.482 | 4799 | ZINC000013585233 | 110.274 |
| 1437 | ZINC000000715918 | 144.622 | 4800 | ZINC000000001084 | 81.1295 |
| 1438 | ZINC000000391161 | 97.9034 | 4801 | ZINC000003881958 | 137.112 |
| 1439 | ZINC000034189841 | 117.016 | 4802 | ZINC000018043251 | 85.4188 |
| 1440 | ZINC000001612828 | 97.3959 | 4803 | ZINC000000895048 | 57.2478 |
| 1441 | ZINC000001559986 | 96.5089 | 4804 | ZINC000000895316 | 47.3912 |
| 1442 | ZINC000001529249 | 100.693 | 4805 | ZINC000002539702 | 90.3629 |
| 1443 | ZINC000001090002 | 103.2 | 4806 | ZINC000000001411 | 93.442 |
| 1444 | ZINC000031474869 | 105.617 | 4807 | ZINC000000895318 | 50.3935 |
| 1445 | ZINC000031160548 | 124.864 | 4808 | ZINC000001530575 | 119.194 |
| 1446 | ZINC000028465419 | 145.349 | 4809 | ZINC000000039111 | 126.845 |
| 1447 | ZINC000027085555 | 91.9083 | 4810 | ZINC000005133273 | 43.2008 |
| 1448 | ZINC000026163116 | 92.1551 | 4811 | ZINC000018847034 | 128.903 |
| 1449 | ZINC000043197010 | 99.8644 | 4812 | ZINC000003847495 | 109.977 |
| 1450 | ZINC000000163657 | 129.779 | 4813 | ZINC000003871176 | 127.548 |
| 1451 | ZINC000040875123 | 103.315 | 4814 | ZINC000003915682 | 56.9696 |
| 1452 | ZINC000040936022 | 117.532 | 4815 | ZINC000003861633 | 107.907 |
| 1453 | ZINC000000858419 | 126.285 | 4816 | ZINC000000156701 | 135.13 |
| 1454 | ZINC000002077819 | 102.104 | 4817 | ZINC000000388512 | 77.0227 |
| 1455 | ZINC000002077817 | 108.341 | 4818 | ZINC000000047553 | 136.463 |
| 1456 | ZINC000018158910 | 109.874 | 4819 | ZINC000000607997 | 103.81 |
| 1457 | ZINC000002039759 | 108.702 | 4820 | ZINC000000828203 | 89.8792 |
| 1458 | ZINC000018247079 | 99.8347 | 4821 | ZINC000000028321 | 120.695 |
| 1459 | ZINC000038765987 | 94.8554 | 4822 | ZINC000000061520 | 119.828 |
| 1460 | ZINC000015148146 | 100.792 | 4823 | ZINC000000898006 | 122.038 |
| 1461 | ZINC000015265032 | 97.1621 | 4824 | ZINC000000015095 | 108.809 |
| 1462 | ZINC000015208889 | 111.704 | 4825 | ZINC000003203266 | 130.314 |
| 1463 | ZINC000015267657 | 125.107 | 4826 | ZINC000005167579 | 112.335 |
| 1464 | ZINC000015052537 | 125.319 | 4827 | ZINC000004098420 | 123.589 |
| 1465 | ZINC000014887151 | 123.76 | 4828 | ZINC000004098343 | 121.131 |
| 1466 | ZINC000000899122 | 119.471 | 4829 | ZINC000000900227 | 29.6057 |
| 1467 | ZINC000000518488 | 124.308 | 4830 | ZINC000004098050 | 104.89 |
| 1468 | ZINC000000608186 | 53.08 | 4831 | ZINC000004098063 | 108.256 |
| 1469 | ZINC000001556409 | 94.5615 | 4832 | ZINC000004098212 | 101.027 |
| 1470 | ZINC000000899046 | 88.4424 | 4833 | ZINC000004097634 | 89.1314 |
| 1471 | ZINC000000899139 | 134.392 | 4834 | ZINC000004097634 | 89.1314 |
| 1472 | ZINC000000899157 | 125.568 | 4835 | ZINC000004097998 | 98.9945 |
| 1473 | ZINC000000899668 | 126.59 | 4836 | ZINC000004082010 | 132.068 |
| 1474 | ZINC000000518797 | 104.772 | 4837 | ZINC000004095655 | 113.979 |
| 1475 | ZINC000001686158 | 110.173 | 4838 | ZINC000001531113 | 98.8944 |
| 1476 | ZINC000001689533 | 136.4 | 4839 | ZINC000000031164 | 99.2524 |
| 1477 | ZINC000000518486 | 129.21 | 4840 | ZINC000000901039 | 47.539 |
| 1478 | ZINC000001618541 | 76.5576 | 4841 | ZINC000000031165 | 98.1442 |
| 1479 | ZINC000001573788 | 139.831 | 4842 | ZINC000000058117 | 131.37 |
| 1480 | ZINC000000525679 | 128.247 | 4843 | ZINC000000391122 | 92.4559 |
| 1481 | ZINC000001562121 | 108.46 | 4844 | ZINC000001693280 | 86.4692 |
| 1482 | ZINC000001529518 | 67.9495 | 4845 | ZINC000001845780 | 105.671 |
| 1483 | ZINC000001565391 | 92.8046 | 4846 | ZINC000000105086 | 138.883 |
| 1484 | ZINC000000586491 | 126.027 | 4847 | ZINC000000899213 | 137.579 |
| 1485 | ZINC000000119386 | 100.202 | 4848 | ZINC000006483512 | 127.115 |
| 1486 | ZINC000000058049 | 97.0858 | 4849 | ZINC000000032351 | 119.371 |
| 1487 | ZINC000000156821 | 97.5894 | 4850 | ZINC000000032351 | 106.45 |
| 1488 | ZINC000000058170 | 137.692 | 4851 | ZINC000000039452 | 118.834 |
| 1489 | ZINC000000119381 | 88.2286 | 4852 | ZINC000028645580 | 87.0257 |
| 1490 | ZINC000000057667 | 132.376 | 4853 | ZINC000000899093 | 125.404 |
| 1491 | ZINC000000119389 | 95.9245 | 4854 | ZINC000000899123 | 129.35 |
| 1492 | ZINC000002039761 | 108.045 | 4855 | ZINC000000897736 | 132.399 |
| 1493 | ZINC000000119385 | 96.6816 | 4856 | ZINC000001651170 | 119.198 |
| 1494 | ZINC000000160491 | 92.8626 | 4857 | ZINC000001740871 | 60.0423 |
| 1495 | ZINC000002039758 | 107.344 | 4858 | ZINC000000058113 | 116.163 |
| 1496 | ZINC000002040431 | 104.724 | 4859 | ZINC000000157526 | 72.5203 |
| 1497 | ZINC000001318428 | 143.794 | 4860 | ZINC000001576883 | 84.9716 |
| 1498 | ZINC000000035531 | 124.754 | 4861 | ZINC000001845692 | 97.4701 |
| 1499 | ZINC000000967600 | 68.4118 | 4862 | ZINC000001666831 | 100.747 |
| 1500 | ZINC000000035528 | 124.36 | 4863 | ZINC000000265490 | 108.274 |
| 1501 | ZINC000000035530 | 129.682 | 4864 | ZINC000001669445 | 92.993 |
| 1502 | ZINC000003984022 | 81.7604 | 4865 | ZINC000005732370 | 121.92 |
| 1503 | ZINC000002561259 | 133.46 | 4866 | ZINC000005133096 | 66.3553 |
| 1504 | ZINC000002561259 | 136.005 | 4867 | ZINC000002019691 | 128.166 |
| 1505 | ZINC000003860434 | 116.33 | 4868 | ZINC000033843063 | 91.9526 |
| 1506 | ZINC000003875803 | 106.224 | 4869 | ZINC000100028895 | 95.1089 |
| 1507 | ZINC000002516970 | 81.6826 | 4870 | ZINC000005133103 | 65.9914 |
| 1508 | ZINC000002561271 | 104.474 | 4871 | ZINC000002019692 | 115.154 |
| 1509 | ZINC000003860823 | 105.411 | 4872 | ZINC000002019694 | 123.725 |
| 1510 | ZINC000003872206 | 104.774 | 4873 | ZINC000001666986 | 66.0293 |
| 1511 | ZINC000003881344 | 127.124 | 4874 | ZINC000001634211 | 81.1415 |
| 1512 | ZINC000003881617 | 83.7617 | 4875 | ZINC000001850485 | 65.2868 |
| 1513 | ZINC000003881717 | 125.698 | 4876 | ZINC000004369791 | 82.3737 |
| 1514 | ZINC000002561268 | 118.749 | 4877 | ZINC000004097939 | 97.3532 |
| 1515 | ZINC000002561268 | 120.233 | 4878 | ZINC000004098814 | 122.34 |
| 1516 | ZINC000003197734 | 107.467 | 4879 | ZINC000003917773 | 107.216 |
| 1517 | ZINC000002170196 | 107.948 | 4880 | ZINC000000338136 | 114.342 |
| 1518 | ZINC000003683318 | 88.766 | 4881 | ZINC000000338136 | 86.8407 |
| 1519 | ZINC000004027427 | 118.269 | 4882 | ZINC000001723143 | 117.899 |
| 1520 | ZINC000004025078 | 107.842 | 4883 | ZINC000059778348 | 89.8865 |
| 1521 | ZINC000000123900 | 125.729 | 4884 | ZINC000001698519 | 99.4595 |
| 1522 | ZINC000034781239 | 83.404 | 4885 | ZINC000000296185 | 105.063 |
| 1523 | ZINC000033832141 | 109.296 | 4886 | ZINC000002554900 | 115.407 |
| 1524 | ZINC000033834009 | 159.355 | 4887 | ZINC000002558154 | 107.809 |
| 1525 | ZINC000000225574 | 121.239 | 4888 | ZINC000005732527 | 110.292 |
| 1526 | ZINC000000488868 | 132.401 | 4889 | ZINC000005706823 | 106.201 |
| 1527 | ZINC000027085551 | 106.2 | 4890 | ZINC000006092939 | 139.983 |
| 1528 | ZINC000000057927 | 123.429 | 4891 | ZINC000005179146 | 131.684 |
| 1529 | ZINC000000060228 | 115.603 | 4892 | ZINC000005158373 | 106.088 |
| 1530 | ZINC000000080361 | 107.268 | 4893 | ZINC000005430816 | 116.238 |
| 1531 | ZINC000000493069 | 131.035 | 4894 | ZINC000059586169 | 88.2848 |
| 1532 | ZINC000000113310 | 117.969 | 4895 | ZINC000004832998 | 107.856 |
| 1533 | ZINC000000003046 | 109.227 | 4896 | ZINC000033805081 | 98.2162 |
| 1534 | ZINC000000057926 | 117.383 | 4897 | ZINC000000071446 | 113.032 |
| 1535 | ZINC000015193873 | 87.402 | 4898 | ZINC000000896426 | 73.1979 |
| 1536 | ZINC000015211904 | 102.955 | 4899 | ZINC000008585874 | 103.856 |
| 1537 | ZINC000014820552 | 138.216 | 4900 | ZINC000008578613 | 93.6618 |
| 1538 | ZINC000014690931 | 91.9996 | 4901 | ZINC000000113473 | 77.8258 |
| 1539 | ZINC000014774001 | 98.3534 | 4902 | ZINC000001532520 | 86.7017 |
| 1540 | ZINC000014776240 | 90.7437 | 4903 | ZINC000000000811 | 94.9901 |
| 1541 | ZINC000014806846 | 147.28 | 4904 | ZINC000000895026 | 91.659 |
| 1542 | ZINC000014445228 | 122.528 | 4905 | ZINC000000014168 | 129.226 |
| 1543 | ZINC000014505171 | 117.503 | 4906 | ZINC000001530833 | 114.967 |
| 1544 | ZINC000014645348 | 122.6 | 4907 | ZINC000018825330 | 135.502 |
| 1545 | ZINC000000001161 | 103.722 | 4908 | ZINC000018825330 | 131.995 |
| 1546 | ZINC000013660192 | 93.9105 | 4909 | ZINC000000119988 | 135.505 |
| 1547 | ZINC000013340319 | 119.759 | 4910 | ZINC000002512351 | 98.0333 |
| 1548 | ZINC000000149770 | 124.078 | 4911 | ZINC000037866089 | 121.859 |
| 1549 | ZINC000000061642 | 117.468 | 4912 | ZINC000001757340 | 90.0307 |
| 1550 | ZINC000000488402 | 101.094 | 4913 | ZINC000001628348 | 107.006 |
| 1551 | ZINC000015848231 | 81.7064 | 4914 | ZINC000003896782 | 106.935 |
| 1552 | ZINC000015167712 | 125.819 | 4915 | ZINC000017970819 | 80.6301 |
| 1553 | ZINC000015193871 | 129.246 | 4916 | ZINC000003881790 | 136.05 |
| 1554 | ZINC000015193875 | 132.938 | 4917 | ZINC000000897152 | 75.7798 |
| 1555 | ZINC000015212183 | 124.501 | 4918 | ZINC000000896527 | 61.8714 |
| 1556 | ZINC000014690904 | 132.117 | 4919 | ZINC000004096682 | 110.879 |
| 1557 | ZINC000014779351 | 121.192 | 4920 | ZINC000004098408 | 80.3502 |
| 1558 | ZINC000014585210 | 132.053 | 4921 | ZINC000002040188 | 67.34 |
| 1559 | ZINC000014617009 | 123.091 | 4922 | ZINC000002031376 | 81.7922 |
| 1560 | ZINC000014640460 | 67.375 | 4923 | ZINC000002039490 | 78.6828 |
| 1561 | ZINC000013373309 | 129.896 | 4924 | ZINC000000058171 | 132.389 |
| 1562 | ZINC000013378577 | 121.616 | 4925 | ZINC000000056434 | 116.509 |
| 1563 | ZINC000000175204 | 105.406 | 4926 | ZINC000000056550 | 83.5956 |
| 1564 | ZINC000013327557 | 130.076 | 4927 | ZINC000015120277 | 88.9868 |
| 1565 | ZINC000002100050 | 128.655 | 4928 | ZINC000004899521 | 112.173 |
| 1566 | ZINC000035271475 | 124.511 | 4929 | ZINC000005761027 | 76.9641 |
| 1567 | ZINC000035031806 | 93.6452 | 4930 | ZINC000005759011 | 78.4368 |
| 1568 | ZINC000000488403 | 104.09 | 4931 | ZINC000001576886 | 72.4066 |
| 1569 | ZINC000000281611 | 112.583 | 4932 | ZINC000001577208 | 73.6228 |
| 1570 | ZINC000000109841 | 107.85 | 4933 | ZINC000001622057 | 87.8586 |
| 1571 | ZINC000070454095 | 129.367 | 4934 | ZINC000001615315 | 54.4032 |
| 1572 | ZINC000000899783 | 116.553 | 4935 | ZINC000001631228 | 96.0851 |
| 1573 | ZINC000030725564 | 140.484 | 4936 | ZINC000001584039 | 104.709 |
| 1574 | ZINC000004098820 | 120.975 | 4937 | ZINC000001592406 | 81.4255 |
| 1575 | ZINC000004428529 | 94.8505 | 4938 | ZINC000001597137 | 77.6854 |
| 1576 | ZINC000002539827 | 115.698 | 4939 | ZINC000003078958 | 104.288 |
| 1577 | ZINC000002539827 | 111.966 | 4940 | ZINC000100779841 | 98.9833 |
| 1578 | ZINC000002169830 | 132.687 | 4941 | ZINC000059588329 | 97.9695 |
| 1579 | ZINC000006827693 | 37.0156 | 4942 | ZINC000059778317 | 100.499 |
| 1580 | ZINC000000895154 | 116.234 | 4943 | ZINC000064622563 | 123.339 |
| 1581 | ZINC000000895154 | 118.76 | 4944 | ZINC000001850313 | 88.428 |
| 1582 | ZINC000004025846 | 111.329 | 4945 | ZINC000100075757 | 83.5292 |
| 1583 | ZINC000002005854 | 56.7325 | 4946 | ZINC000100075761 | 78.8179 |
| 1584 | ZINC000012418399 | 91.5801 | 4947 | ZINC000100488727 | 65.4965 |
| 1585 | ZINC000002001176 | 84.6972 | 4948 | ZINC000100775666 | 122.196 |
| 1586 | ZINC000000895313 | 57.6628 | 4949 | ZINC000038198783 | 91.5022 |
| 1587 | ZINC000000895905 | 96.6137 | 4950 | ZINC000021999704 | 102.682 |
| 1588 | ZINC000000895928 | 83.4006 | 4951 | ZINC000165791815 | 72.0825 |
| 1589 | ZINC000000409386 | 68.283 | 4952 | ZINC000031290833 | 101.618 |
| 1590 | ZINC000000494255 | 92.0571 | 4953 | ZINC000031475157 | 95.0987 |
| 1591 | ZINC000000895436 | 50.2309 | 4954 | ZINC000031484861 | 87.4786 |
| 1592 | ZINC000012362794 | 84.7292 | 4955 | ZINC000032165362 | 60.9585 |
| 1593 | ZINC000018188714 | 94.8551 | 4956 | ZINC000032166540 | 71.0998 |
| 1594 | ZINC000018188714 | 87.3869 | 4957 | ZINC000032788664 | 127.261 |
| 1595 | ZINC000000409286 | 79.7005 | 4958 | ZINC000033611419 | 100.238 |
| 1596 | ZINC000000896810 | 76.6923 | 4959 | ZINC000033650331 | 100.766 |
| 1597 | ZINC000000897141 | 70.827 | 4960 | ZINC000033950845 | 94.1037 |
| 1598 | ZINC000000897468 | 86.8616 | 4961 | ZINC000034287734 | 79.554 |
| 1599 | ZINC000000895145 | 68.2333 | 4962 | ZINC000034746446 | 86.1384 |
| 1600 | ZINC000004096636 | 127.824 | 4963 | ZINC000013674168 | 113.116 |
| 1601 | ZINC000021986282 | 98.5684 | 4964 | ZINC000014421713 | 107.103 |
| 1602 | ZINC000022000195 | 66.6156 | 4965 | ZINC000014588387 | 82.9852 |
| 1603 | ZINC000012359992 | 119.55 | 4966 | ZINC000014588389 | 90.5846 |
| 1604 | ZINC000012428433 | 134.771 | 4967 | ZINC000014588423 | 92.4036 |
| 1605 | ZINC000013282307 | 105.667 | 4968 | ZINC000014588589 | 83.3448 |
| 1606 | ZINC000008628578 | 112.869 | 4969 | ZINC000014589053 | 92.4243 |
| 1607 | ZINC000004645692 | 116.97 | 4970 | ZINC000014592712 | 82.3066 |
| 1608 | ZINC000021303045 | 63.6153 | 4971 | ZINC000014614317 | 83.0702 |
| 1609 | ZINC000003831417 | 115.094 | 4972 | ZINC000014616892 | 96.1303 |
| 1610 | ZINC000019850177 | 105.165 | 4973 | ZINC000014649301 | 85.3609 |
| 1611 | ZINC000000409354 | 72.0619 | 4974 | ZINC000014715656 | 104.759 |
| 1612 | ZINC000000409330 | 86.884 | 4975 | ZINC000014822344 | 93.4055 |
| 1613 | ZINC000000487825 | 93.1731 | 4976 | ZINC000014822384 | 93.5362 |
| 1614 | ZINC000000402870 | 99.5282 | 4977 | ZINC000015272647 | 73.5581 |
| 1615 | ZINC000000402671 | 105.266 | 4978 | ZINC000011631246 | 99.8857 |
| 1616 | ZINC000001648359 | 68.933 | 4979 | ZINC000012153785 | 77.8848 |
| 1617 | ZINC000001574407 | 79.0344 | 4980 | ZINC000012493591 | 108.487 |
| 1618 | ZINC000013585362 | 100.057 | 4981 | ZINC000013130928 | 152.048 |
| 1619 | ZINC000000895129 | 87.8479 | 4982 | ZINC000013383221 | 119.419 |
| 1620 | ZINC000000895247 | 67.1794 | 4983 | ZINC000005722724 | 90.8043 |
| 1621 | ZINC000004096880 | 130.799 | 4984 | ZINC000005766879 | 90.1714 |
| 1622 | ZINC000034143744 | 64.4679 | 4985 | ZINC000005767121 | 93.9593 |
| 1623 | ZINC000095618218 | 106.394 | 4986 | ZINC000005767209 | 103.33 |
| 1624 | ZINC000036478810 | 110.337 | 4987 | ZINC000005767269 | 90.8362 |
| 1625 | ZINC000000895111 | 38.8045 | 4988 | ZINC000005819330 | 73.6929 |
| 1626 | ZINC000095617634 | 97.7608 | 4989 | ZINC000005820128 | 83.4903 |
| 1627 | ZINC000001395181 | 115.032 | 4990 | ZINC000005821936 | 81.1031 |
| 1628 | ZINC000000599985 | 60.9881 | 4991 | ZINC000005822251 | 79.8754 |
| 1629 | ZINC000019816066 | 92.2536 | 4992 | ZINC000005998230 | 87.2727 |
| 1630 | ZINC000014642046 | 145.346 | 4993 | ZINC000006031106 | 73.3555 |
| 1631 | ZINC000013378576 | 120.327 | 4994 | ZINC000006031308 | 97.6024 |
| 1632 | ZINC000013572724 | 86.5337 | 4995 | ZINC000006037901 | 96.1373 |
| 1633 | ZINC000014437230 | 79.7411 | 4996 | ZINC000006069856 | 74.5511 |
| 1634 | ZINC000003870887 | 96.805 | 4997 | ZINC000006070723 | 94.8459 |
| 1635 | ZINC000038212689 | 75.5215 | 4998 | ZINC000008700366 | 88.4858 |
| 1636 | ZINC000100191509 | 144.09 | 4999 | ZINC000001850051 | 80.8188 |
| 1637 | ZINC000100191509 | 145.957 | 5000 | ZINC000001850811 | 97.8022 |
| 1638 | ZINC000006484540 | 104.986 | 5001 | ZINC000001850889 | 79.1743 |
| 1639 | ZINC000070454448 | 104.217 | 5002 | ZINC000001850936 | 96.6944 |
| 1640 | ZINC000100051847 | 102.499 | 5003 | ZINC000001871730 | 73.8068 |
| 1641 | ZINC000004098822 | 123.003 | 5004 | ZINC000001871733 | 73.2809 |
| 1642 | ZINC000004098705 | 111.426 | 5005 | ZINC000002029395 | 115.995 |
| 1643 | ZINC000100031035 | 127.412 | 5006 | ZINC000002037536 | 108.557 |
| 1644 | ZINC000000105082 | 141.192 | 5007 | ZINC000002567754 | 114.524 |
| 1645 | ZINC000016889962 | 56.2524 | 5008 | ZINC000003630810 | 75.8254 |
| 1646 | ZINC000000402826 | 78.1585 | 5009 | ZINC000003869621 | 92.3181 |
| 1647 | ZINC000006117488 | 88.5982 | 5010 | ZINC000003869863 | 92.8353 |
| 1648 | ZINC000001639355 | 120.962 | 5011 | ZINC000004829970 | 78.7305 |
| 1649 | ZINC000000899161 | 133.087 | 5012 | ZINC000005159252 | 99.337 |
| 1650 | ZINC000000896042 | 139.903 | 5013 | ZINC000005513439 | 86.0078 |
| 1651 | ZINC000001657403 | 106.11 | 5014 | ZINC000014442550 | 110.343 |
| 1652 | ZINC000001721178 | 141.713 | 5015 | ZINC000014503322 | 97.0505 |
| 1653 | ZINC000001893420 | 106.53 | 5016 | ZINC000014588468 | 91.7701 |
| 1654 | ZINC000003957079 | 96.9566 | 5017 | ZINC000014588968 | 114.253 |
| 1655 | ZINC000004073899 | 124.537 | 5018 | ZINC000014642224 | 139.277 |
| 1656 | ZINC000004097623 | 65.1607 | 5019 | ZINC000014642991 | 130.028 |
| 1657 | ZINC000004217594 | 102.267 | 5020 | ZINC000014643000 | 150.481 |
| 1658 | ZINC000004716487 | 124.922 | 5021 | ZINC000014686696 | 143.957 |
| 1659 | ZINC000006484604 | 142.727 | 5022 | ZINC000014821963 | 131.487 |
| 1660 | ZINC000006530699 | 133.047 | 5023 | ZINC000014822214 | 72.9721 |
| 1661 | ZINC000012504453 | 89.6028 | 5024 | ZINC000014854293 | 81.2296 |
| 1662 | ZINC000013341109 | 126.197 | 5025 | ZINC000014883474 | 76.5802 |
| 1663 | ZINC000014779318 | 110.154 | 5026 | ZINC000014883477 | 85.1987 |
| 1664 | ZINC000016051972 | 96.0041 | 5027 | ZINC000015062584 | 102.065 |
| 1665 | ZINC000017175232 | 105.206 | 5028 | ZINC000015169318 | 98.8498 |
| 1666 | ZINC000019419113 | 102.242 | 5029 | ZINC000059283873 | 112.448 |
| 1667 | ZINC000000057949 | 88.2335 | 5030 | ZINC000095618233 | 118.157 |
| 1668 | ZINC000000058114 | 118.703 | 5031 | ZINC000000394813 | 105.207 |
| 1669 | ZINC000001588038 | 94.9138 | 5032 | ZINC000000895798 | 99.5881 |
| 1670 | ZINC000002950199 | 114.611 | 5033 | ZINC000001531619 | 83.9321 |
| 1671 | ZINC000005732241 | 138.293 | 5034 | ZINC000001664327 | 108.441 |
| 1672 | ZINC000013132551 | 112.886 | 5035 | ZINC000001684139 | 135.594 |
| 1673 | ZINC000013512198 | 81.1557 | 5036 | ZINC000001685984 | 104.141 |
| 1674 | ZINC000013838505 | 109.698 | 5037 | ZINC000004556492 | 121.395 |
| 1675 | ZINC000014727564 | 100.479 | 5038 | ZINC000005115873 | 118.808 |
| 1676 | ZINC000018804836 | 110.442 | 5039 | ZINC000005358329 | 118.842 |
| 1677 | ZINC000000039178 | 88.7546 | 5040 | ZINC000005513417 | 96.6144 |
| 1678 | ZINC000000057719 | 102.003 | 5041 | ZINC000011631247 | 98.4483 |
| 1679 | ZINC000000154645 | 84.3088 | 5042 | ZINC000012405105 | 122.154 |
| 1680 | ZINC000000406883 | 111.721 | 5043 | ZINC000013481852 | 86.1203 |
| 1681 | ZINC000000896120 | 139.972 | 5044 | ZINC000013542048 | 68.2867 |
| 1682 | ZINC000001532739 | 84.4077 | 5045 | ZINC000013542648 | 87.8768 |
| 1683 | ZINC000003795857 | 102.428 | 5046 | ZINC000013548506 | 124.198 |
| 1684 | ZINC000003874832 | 119.237 | 5047 | ZINC000013783705 | 134.788 |
| 1685 | ZINC000053174616 | 141.159 | 5048 | ZINC000014420733 | 119.084 |
| 1686 | ZINC000100031141 | 96.2076 | 5049 | ZINC000095618199 | 123.906 |
| 1687 | ZINC000001566147 | 117.558 | 5050 | ZINC000097973720 | 124.713 |
| 1688 | ZINC000013518422 | 120.501 | 5051 | ZINC000001530300 | 76.6796 |
| 1689 | ZINC000000058187 | 89.7893 | 5052 | ZINC000001614086 | 63.8424 |
| 1690 | ZINC000005842416 | 143.241 | 5053 | ZINC000001662871 | 107.439 |
| 1691 | ZINC000000899531 | 88.2545 | 5054 | ZINC000001711823 | 125.176 |
| 1692 | ZINC000000901191 | 89.5032 | 5055 | ZINC000001713247 | 70.7192 |
| 1693 | ZINC000000901405 | 79.46 | 5056 | ZINC000001849926 | 107.751 |
| 1694 | ZINC000000899240 | 90.1675 | 5057 | ZINC000001996094 | 102.585 |
| 1695 | ZINC000000899172 | 125.456 | 5058 | ZINC000002038441 | 61.0137 |
| 1696 | ZINC000001532765 | 61.25 | 5059 | ZINC000002525300 | 143.712 |
| 1697 | ZINC000000896695 | 65.3968 | 5060 | ZINC000004262047 | 120.678 |
| 1698 | ZINC000000901552 | 53.9255 | 5061 | ZINC000100028356 | 83.1196 |
| 1699 | ZINC000000901555 | 51.3998 | 5062 | ZINC000100771605 | 137.464 |
| 1700 | ZINC000000895302 | 72.4243 | 5063 | ZINC000100783456 | 107.615 |
| 1701 | ZINC000100037020 | 139.568 | 5064 | ZINC000038392781 | 105.365 |
| 1702 | ZINC000000518554 | 128.565 | 5065 | ZINC000043502271 | 85.9127 |
| 1703 | ZINC000000035529 | 120.77 | 5066 | ZINC000008568878 | 117.903 |
| 1704 | ZINC000038141473 | 111.704 | 5067 | ZINC000008568878 | 111.901 |
| 1705 | ZINC000000968281 | 66.3396 | 5068 | ZINC000012495470 | 118.376 |
| 1706 | ZINC000004474575 | 120.032 | 5069 | ZINC000012495470 | 122.084 |
| 1707 | ZINC000000058222 | 102.888 | 5070 | ZINC000012495479 | 119.989 |
| 1708 | ZINC000028107226 | 120.03 | 5071 | ZINC000012495479 | 115.983 |
| 1709 | ZINC000013424754 | 78.01 | 5072 | ZINC000013484895 | 93.2069 |
| 1710 | ZINC000008214691 | 46.7193 | 5073 | ZINC000013484895 | 93.679 |
| 1711 | ZINC000004097652 | 127.63 | 5074 | ZINC000018036401 | 117.024 |
| 1712 | ZINC000049600032 | 117.256 | 5075 | ZINC000022062286 | 107.399 |
| 1713 | ZINC000006483405 | 97.3992 | 5076 | ZINC000026387738 | 105.199 |
| 1714 | ZINC000006483405 | 93.8121 | 5077 | ZINC000032152465 | 67.0087 |
| 1715 | ZINC000006092955 | 138.47 | 5078 | ZINC000033951265 | 67.8753 |
| 1716 | ZINC000005996060 | 79.3245 | 5079 | ZINC000013380320 | 65.102 |
| 1717 | ZINC000006003042 | 115.342 | 5080 | ZINC000038611803 | 79.0538 |
| 1718 | ZINC000006019631 | 84.8838 | 5081 | ZINC000005820127 | 117.059 |
| 1719 | ZINC000005821904 | 98.2905 | 5082 | ZINC000034828682 | 83.6524 |
| 1720 | ZINC000005765855 | 91.5737 | 5083 | ZINC000005950348 | 104.7 |
| 1721 | ZINC000101103208 | 109.962 | 5084 | ZINC000006018558 | 41.1716 |
| 1722 | ZINC000100076738 | 110.878 | 5085 | ZINC000005460778 | 77.599 |
| 1723 | ZINC000004097976 | 128.511 | 5086 | ZINC000003861737 | 92.7213 |
| 1724 | ZINC000003197541 | 90.6533 | 5087 | ZINC000004026294 | 114.705 |
| 1725 | ZINC000000040395 | 107.632 | 5088 | ZINC000004027981 | 113.305 |
| 1726 | ZINC000000834478 | 89.6571 | 5089 | ZINC000002528314 | 95.0989 |
| 1727 | ZINC000000025159 | 128.002 | 5090 | ZINC000000898220 | 121.526 |
| 1728 | ZINC000005225089 | 92.065 | 5091 | ZINC000000902225 | 87.4231 |
| 1729 | ZINC000101179362 | 83.288 | 5092 | ZINC000001294932 | 50.187 |
| 1730 | ZINC000005157651 | 119.339 | 5093 | ZINC000001410176 | 88.9317 |
| 1731 | ZINC000005158354 | 102.469 | 5094 | ZINC000001755317 | 101.807 |
| 1732 | ZINC000005134316 | 85.2615 | 5095 | ZINC000002031533 | 73.4924 |
| 1733 | ZINC000004900009 | 98.0731 | 5096 | ZINC000096017931 | 86.3911 |
| 1734 | ZINC000004716494 | 141.231 | 5097 | ZINC000000306698 | 99.6761 |
| 1735 | ZINC000004654725 | 129.837 | 5098 | ZINC000000402672 | 110.068 |
| 1736 | ZINC000004716495 | 134.168 | 5099 | ZINC000100372231 | 64.0833 |
| 1737 | ZINC000004654730 | 128.033 | 5100 | ZINC000100777316 | 128.292 |
| 1738 | ZINC000095628034 | 83.8335 | 5101 | ZINC000049878297 | 119.942 |
| 1739 | ZINC000005732757 | 83.1579 | 5102 | ZINC000085949359 | 90.1073 |
| 1740 | ZINC000169371120 | 83.5467 | 5103 | ZINC000086051998 | 106.427 |
| 1741 | ZINC000005732375 | 140.364 | 5104 | ZINC000015121956 | 137.196 |
| 1742 | ZINC000005732375 | 136.954 | 5105 | ZINC000085592952 | 96.5624 |
| 1743 | ZINC000031156069 | 118.748 | 5106 | ZINC000004228312 | 70.5523 |
| 1744 | ZINC000031156069 | 114.589 | 5107 | ZINC000014687878 | 115.24 |
| 1745 | ZINC000005447705 | 122.988 | 5108 | ZINC000014687882 | 111.972 |
| 1746 | ZINC000002039891 | 89.3215 | 5109 | ZINC000004521894 | 69.6853 |
| 1747 | ZINC000000340372 | 154.464 | 5110 | ZINC000004098308 | 89.3989 |
| 1748 | ZINC000000155362 | 74.0795 | 5111 | ZINC000002038539 | 82.1476 |
| 1749 | ZINC000003979084 | 141.379 | 5112 | ZINC000013282986 | 111.096 |
| 1750 | ZINC000004073945 | 129.07 | 5113 | ZINC000033951308 | 73.793 |
| 1751 | ZINC000003683833 | 76.1932 | 5114 | ZINC000000402941 | 90.0574 |
| 1752 | ZINC000003777403 | 143.721 | 5115 | ZINC000001850311 | 91.1393 |
| 1753 | ZINC000003777403 | 137.288 | 5116 | ZINC000000391989 | 70.3024 |
| 1754 | ZINC000003876041 | 73.083 | 5117 | ZINC000077050241 | 82.2658 |
| 1755 | ZINC000005998737 | 103.092 | 5118 | ZINC000077050244 | 82.2309 |
| 1756 | ZINC000001665979 | 117.517 | 5119 | ZINC000078160854 | 72.1252 |
| 1757 | ZINC000001531449 | 138.262 | 5120 | ZINC000078160856 | 72.4546 |
| 1758 | ZINC000001530818 | 116.334 | 5121 | ZINC000078366699 | 61.9404 |
| 1759 | ZINC000018189423 | 92.962 | 5122 | ZINC000070595318 | 78.4814 |
| 1760 | ZINC000032911469 | 88.2624 | 5123 | ZINC000070451040 | 123.257 |
| 1761 | ZINC000001846159 | 110.507 | 5124 | ZINC000001850902 | 98.9303 |
| 1762 | ZINC000019861870 | 115.896 | 5125 | ZINC000100779842 | 113.109 |
| 1763 | ZINC000019230132 | 88.2624 | 5126 | ZINC000100781166 | 94.9312 |
| 1764 | ZINC000003977990 | 85.8558 | 5127 | ZINC000059585784 | 80.2056 |
| 1765 | ZINC000004027061 | 108.872 | 5128 | ZINC000100059038 | 100.369 |
| 1766 | ZINC000004081122 | 105.44 | 5129 | ZINC000100069556 | 80.1075 |
| 1767 | ZINC000004082455 | 139.652 | 5130 | ZINC000100075758 | 82.7081 |
| 1768 | ZINC000000004164 | 139.954 | 5131 | ZINC000057986694 | 95.3427 |
| 1769 | ZINC000003087481 | 150.317 | 5132 | ZINC000100775663 | 124.81 |
| 1770 | ZINC000003775158 | 140.918 | 5133 | ZINC000038613343 | 85.3649 |
| 1771 | ZINC000003775158 | 133.819 | 5134 | ZINC000044431718 | 101.887 |
| 1772 | ZINC000003861735 | 91.5666 | 5135 | ZINC000008220171 | 111.805 |
| 1773 | ZINC000003875781 | 129.368 | 5136 | ZINC000021995542 | 64.8603 |
| 1774 | ZINC000003894278 | 142.1 | 5137 | ZINC000025669570 | 113.926 |
| 1775 | ZINC000053098690 | 116.882 | 5138 | ZINC000028537734 | 125.819 |
| 1776 | ZINC000100011469 | 126.662 | 5139 | ZINC000031290836 | 103.159 |
| 1777 | ZINC000000057358 | 97.8982 | 5140 | ZINC000031475165 | 96.3011 |
| 1778 | ZINC000000019968 | 103.965 | 5141 | ZINC000031484864 | 87.3587 |
| 1779 | ZINC000013907151 | 138.158 | 5142 | ZINC000032165360 | 60.7361 |
| 1780 | ZINC000014759160 | 130.762 | 5143 | ZINC000032166539 | 70.37 |
| 1781 | ZINC000014927453 | 105.864 | 5144 | ZINC000032840885 | 126.939 |
| 1782 | ZINC000013327880 | 100.694 | 5145 | ZINC000033950846 | 96.6427 |
| 1783 | ZINC000013327880 | 102.273 | 5146 | ZINC000004521482 | 93.3563 |
| 1784 | ZINC000014692054 | 119.865 | 5147 | ZINC000013520486 | 93.5755 |
| 1785 | ZINC000013334818 | 63.9045 | 5148 | ZINC000013521393 | 80.0828 |
| 1786 | ZINC000014594569 | 85.3653 | 5149 | ZINC000014438654 | 78.7828 |
| 1787 | ZINC000014615966 | 114.06 | 5150 | ZINC000014498209 | 105.149 |
| 1788 | ZINC000044307421 | 105.772 | 5151 | ZINC000014588388 | 91.5461 |
| 1789 | ZINC000014489321 | 108.105 | 5152 | ZINC000014588390 | 92.3488 |
| 1790 | ZINC000031159830 | 116.067 | 5153 | ZINC000014588424 | 91.0568 |
| 1791 | ZINC000002558155 | 132.911 | 5154 | ZINC000014589052 | 93.6195 |
| 1792 | ZINC000072108660 | 131.52 | 5155 | ZINC000014592713 | 76.7275 |
| 1793 | ZINC000005732373 | 124.342 | 5156 | ZINC000014614330 | 86.9688 |
| 1794 | ZINC000000001785 | 136.064 | 5157 | ZINC000014616381 | 81.0722 |
| 1795 | ZINC000013334446 | 121.267 | 5158 | ZINC000014649299 | 85.9696 |
| 1796 | ZINC000014692052 | 123.081 | 5159 | ZINC000014649303 | 82.4117 |
| 1797 | ZINC000031156172 | 105.685 | 5160 | ZINC000014822381 | 93.6558 |
| 1798 | ZINC000013480243 | 63.2429 | 5161 | ZINC000015120477 | 96.5459 |
| 1799 | ZINC000028541771 | 86.4472 | 5162 | ZINC000015272645 | 70.1703 |
| 1800 | ZINC000029133928 | 111.437 | 5163 | ZINC000012153320 | 76.5472 |
| 1801 | ZINC000026280943 | 97.1586 | 5164 | ZINC000012358689 | 89.5264 |
| 1802 | ZINC000013660139 | 96.051 | 5165 | ZINC000012493539 | 129.353 |
| 1803 | ZINC000013118651 | 90.8601 | 5166 | ZINC000012496212 | 68.4638 |
| 1804 | ZINC000031156518 | 128.342 | 5167 | ZINC000012496212 | 71.8591 |
| 1805 | ZINC000013334350 | 105.942 | 5168 | ZINC000013433426 | 99.1077 |
| 1806 | ZINC000016926084 | 124.534 | 5169 | ZINC000002039889 | 55.0308 |
| 1807 | ZINC000038435846 | 92.4771 | 5170 | ZINC000005735741 | 73.1564 |
| 1808 | ZINC000039350454 | 109.808 | 5171 | ZINC000005761845 | 74.7712 |
| 1809 | ZINC000040380449 | 96.538 | 5172 | ZINC000005766881 | 93.1413 |
| 1810 | ZINC000005998918 | 119.584 | 5173 | ZINC000005767135 | 90.1121 |
| 1811 | ZINC000005998918 | 126.707 | 5174 | ZINC000005767255 | 92.715 |
| 1812 | ZINC000000021789 | 99.3981 | 5175 | ZINC000005820001 | 105.328 |
| 1813 | ZINC000000320698 | 59.8596 | 5176 | ZINC000005821935 | 81.5432 |
| 1814 | ZINC000002031813 | 146.323 | 5177 | ZINC000005822250 | 81.1548 |
| 1815 | ZINC000000967819 | 98.8662 | 5178 | ZINC000006020522 | 84.8857 |
| 1816 | ZINC000008234227 | 67.7271 | 5179 | ZINC000006031292 | 94.7586 |
| 1817 | ZINC000004252597 | 120.282 | 5180 | ZINC000006036299 | 86.1877 |
| 1818 | ZINC000001691180 | 139.414 | 5181 | ZINC000006037730 | 73.2838 |
| 1819 | ZINC000096923234 | 102.333 | 5182 | ZINC000006037917 | 97.82 |
| 1820 | ZINC000100898573 | 91.7844 | 5183 | ZINC000006069019 | 94.6853 |
| 1821 | ZINC000100230821 | 104.293 | 5184 | ZINC000006070288 | 122.807 |
| 1822 | ZINC000100152478 | 83.2175 | 5185 | ZINC000006091886 | 113.397 |
| 1823 | ZINC000100734137 | 76.3039 | 5186 | ZINC000008234284 | 92.2074 |
| 1824 | ZINC000030728630 | 65.6787 | 5187 | ZINC000006484761 | 135.502 |
| 1825 | ZINC000030728634 | 65.5276 | 5188 | ZINC000001846611 | 105.833 |
| 1826 | ZINC000014725518 | 116.031 | 5189 | ZINC000001849808 | 99.0763 |
| 1827 | ZINC000014644952 | 93.4531 | 5190 | ZINC000001850044 | 82.2125 |
| 1828 | ZINC000014417338 | 141.903 | 5191 | ZINC000001850048 | 83.6813 |
| 1829 | ZINC000013815791 | 116.636 | 5192 | ZINC000001850358 | 74.3781 |
| 1830 | ZINC000013481984 | 105.745 | 5193 | ZINC000001850521 | 83.6641 |
| 1831 | ZINC000013434200 | 106.402 | 5194 | ZINC000001850888 | 76.9493 |
| 1832 | ZINC000013377892 | 113.8 | 5195 | ZINC000001850935 | 97.7481 |
| 1833 | ZINC000013378578 | 121.126 | 5196 | ZINC000001871732 | 73.3162 |
| 1834 | ZINC000013377893 | 109.653 | 5197 | ZINC000001871735 | 71.4889 |
| 1835 | ZINC000013152592 | 102.903 | 5198 | ZINC000002037700 | 69.3022 |
| 1836 | ZINC000000897924 | 105.848 | 5199 | ZINC000002038706 | 84.6231 |
| 1837 | ZINC000000900208 | 91.8118 | 5200 | ZINC000002118296 | 78.1204 |
| 1838 | ZINC000000898345 | 84.5249 | 5201 | ZINC000002567753 | 91.1173 |
| 1839 | ZINC000000897926 | 107.56 | 5202 | ZINC000003630807 | 77.0672 |
| 1840 | ZINC000000898309 | 88.5379 | 5203 | ZINC000003869622 | 97.905 |
| 1841 | ZINC000001723552 | 113.205 | 5204 | ZINC000003869862 | 92.4369 |
| 1842 | ZINC000001690954 | 89.7458 | 5205 | ZINC000004095712 | 117.137 |
| 1843 | ZINC000001664037 | 99.8235 | 5206 | ZINC000004096637 | 118.343 |
| 1844 | ZINC000001645590 | 152.092 | 5207 | ZINC000004098404 | 85.854 |
| 1845 | ZINC000001645590 | 151.583 | 5208 | ZINC000004654624 | 115.561 |
| 1846 | ZINC000001674393 | 92.7705 | 5209 | ZINC000004654893 | 96.1507 |
| 1847 | ZINC000000391162 | 95.5924 | 5210 | ZINC000004829971 | 77.2507 |
| 1848 | ZINC000001557770 | 113.908 | 5211 | ZINC000005037499 | 130.38 |
| 1849 | ZINC000001531364 | 121.079 | 5212 | ZINC000005360100 | 113.476 |
| 1850 | ZINC000000384402 | 88.4395 | 5213 | ZINC000005509366 | 74.455 |
| 1851 | ZINC000031154896 | 116.862 | 5214 | ZINC000005513289 | 84.6797 |
| 1852 | ZINC000028875643 | 93.3272 | 5215 | ZINC000005513289 | 89.8942 |
| 1853 | ZINC000028968268 | 135.599 | 5216 | ZINC000014439153 | 94.4929 |
| 1854 | ZINC000028702246 | 103.261 | 5217 | ZINC000014488318 | 124.115 |
| 1855 | ZINC000028222051 | 143.046 | 5218 | ZINC000014588967 | 104.39 |
| 1856 | ZINC000022055629 | 104.304 | 5219 | ZINC000014642230 | 149.964 |
| 1857 | ZINC000043200202 | 119.323 | 5220 | ZINC000014642997 | 128.5 |
| 1858 | ZINC000040877965 | 91.8624 | 5221 | ZINC000014778687 | 129.559 |
| 1859 | ZINC000040576706 | 96.9051 | 5222 | ZINC000014817963 | 81.9822 |
| 1860 | ZINC000040894591 | 104.844 | 5223 | ZINC000014822225 | 78.0214 |
| 1861 | ZINC000000174849 | 113.335 | 5224 | ZINC000014828986 | 82.249 |
| 1862 | ZINC000018103090 | 69.5952 | 5225 | ZINC000014855479 | 123.884 |
| 1863 | ZINC000002020050 | 83.9618 | 5226 | ZINC000014883475 | 77.4022 |
| 1864 | ZINC000018021836 | 94.0509 | 5227 | ZINC000014883478 | 85.0267 |
| 1865 | ZINC000038816510 | 130.945 | 5228 | ZINC000015062581 | 103.467 |
| 1866 | ZINC000038304118 | 102.938 | 5229 | ZINC000015113336 | 108.106 |
| 1867 | ZINC000038228861 | 121.513 | 5230 | ZINC000100067862 | 135.423 |
| 1868 | ZINC000015211898 | 110.961 | 5231 | ZINC000000008866 | 130.719 |
| 1869 | ZINC000015250922 | 106.279 | 5232 | ZINC000000394812 | 102.437 |
| 1870 | ZINC000015207341 | 106.163 | 5233 | ZINC000000895795 | 99.6443 |
| 1871 | ZINC000014768470 | 109.602 | 5234 | ZINC000000967799 | 91.021 |
| 1872 | ZINC000014762993 | 128.147 | 5235 | ZINC000001530228 | 86.6609 |
| 1873 | ZINC000014762993 | 134.15 | 5236 | ZINC000001531140 | 92.68 |
| 1874 | ZINC000014766771 | 139.754 | 5237 | ZINC000001663924 | 67.5154 |
| 1875 | ZINC000014780122 | 138.417 | 5238 | ZINC000001680680 | 55.6945 |
| 1876 | ZINC000014780122 | 117.092 | 5239 | ZINC000001685819 | 107.835 |
| 1877 | ZINC000014780926 | 148.214 | 5240 | ZINC000001694875 | 109.808 |
| 1878 | ZINC000014766769 | 140.286 | 5241 | ZINC000004521481 | 95.7689 |
| 1879 | ZINC000014762603 | 122.122 | 5242 | ZINC000004658595 | 47.4182 |
| 1880 | ZINC000002109870 | 110.604 | 5243 | ZINC000004803675 | 123.826 |
| 1881 | ZINC000008579393 | 103.008 | 5244 | ZINC000005192324 | 90.6555 |
| 1882 | ZINC000006483423 | 138.343 | 5245 | ZINC000005358398 | 112.224 |
| 1883 | ZINC000006483425 | 110.969 | 5246 | ZINC000005513256 | 99.6309 |
| 1884 | ZINC000006491310 | 103.793 | 5247 | ZINC000005765982 | 72.7826 |
| 1885 | ZINC000008234270 | 122.325 | 5248 | ZINC000005840154 | 74.8853 |
| 1886 | ZINC000005854691 | 138.35 | 5249 | ZINC000006361574 | 79.1454 |
| 1887 | ZINC000006116425 | 133.304 | 5250 | ZINC000012153445 | 86.2219 |
| 1888 | ZINC000005761646 | 128.648 | 5251 | ZINC000012504162 | 130.059 |
| 1889 | ZINC000006070275 | 104.323 | 5252 | ZINC000013396777 | 50.2849 |
| 1890 | ZINC000006067600 | 104.262 | 5253 | ZINC000013484727 | 115.794 |
| 1891 | ZINC000006017923 | 104.222 | 5254 | ZINC000095618198 | 109.508 |
| 1892 | ZINC000005732364 | 103.348 | 5255 | ZINC000095618219 | 107.501 |
| 1893 | ZINC000005732364 | 111.572 | 5256 | ZINC000000338221 | 107.14 |
| 1894 | ZINC000006017925 | 106.074 | 5257 | ZINC000000895418 | 89.1395 |
| 1895 | ZINC000006017650 | 85.4559 | 5258 | ZINC000000897738 | 120.753 |
| 1896 | ZINC000005998961 | 105.296 | 5259 | ZINC000001320093 | 128.089 |
| 1897 | ZINC000005998961 | 105.377 | 5260 | ZINC000001711822 | 103.945 |
| 1898 | ZINC000005998754 | 114.296 | 5261 | ZINC000001711824 | 107.71 |
| 1899 | ZINC000005998754 | 138.548 | 5262 | ZINC000001845815 | 80.4055 |
| 1900 | ZINC000005999049 | 142.734 | 5263 | ZINC000001849924 | 96.5557 |
| 1901 | ZINC000005733553 | 135.621 | 5264 | ZINC000100769745 | 92.1844 |
| 1902 | ZINC000005998555 | 131.509 | 5265 | ZINC000043502273 | 82.2535 |
| 1903 | ZINC000005648525 | 128.171 | 5266 | ZINC000012153055 | 124.814 |
| 1904 | ZINC000006520505 | 109.285 | 5267 | ZINC000012495474 | 118.307 |
| 1905 | ZINC000006481654 | 120.31 | 5268 | ZINC000012495474 | 120.507 |
| 1906 | ZINC000006520504 | 119.076 | 5269 | ZINC000013540015 | 137.873 |
| 1907 | ZINC000006484603 | 105.66 | 5270 | ZINC000017994680 | 116.908 |
| 1908 | ZINC000006484603 | 116.734 | 5271 | ZINC000018068098 | 88.115 |
| 1909 | ZINC000005534507 | 100.813 | 5272 | ZINC000022062288 | 119.848 |
| 1910 | ZINC000005822220 | 95.0897 | 5273 | ZINC000026387734 | 133.528 |
| 1911 | ZINC000006116424 | 125.829 | 5274 | ZINC000033838509 | 101.073 |
| 1912 | ZINC000006092732 | 142.091 | 5275 | ZINC000033951264 | 66.5693 |
| 1913 | ZINC000005762063 | 138.244 | 5276 | ZINC000085831743 | 122.683 |
| 1914 | ZINC000005762063 | 147.126 | 5277 | ZINC000001531107 | 122.74 |
| 1915 | ZINC000006093689 | 117.884 | 5278 | ZINC000013437345 | 109.159 |
| 1916 | ZINC000005765136 | 93.8204 | 5279 | ZINC000008616481 | 93.0206 |
| 1917 | ZINC000005733551 | 142.983 | 5280 | ZINC000008952459 | 124.324 |
| 1918 | ZINC000005733551 | 137.83 | 5281 | ZINC000006067785 | 105.962 |
| 1919 | ZINC000005963645 | 123.866 | 5282 | ZINC000005820126 | 112.881 |
| 1920 | ZINC000005998671 | 141.001 | 5283 | ZINC000005997565 | 81.1034 |
| 1921 | ZINC000005998943 | 125.751 | 5284 | ZINC000006019180 | 104.617 |
| 1922 | ZINC000005999141 | 71.9736 | 5285 | ZINC000053275063 | 117.067 |
| 1923 | ZINC000005858402 | 79.7922 | 5286 | ZINC000004715091 | 85.5244 |
| 1924 | ZINC000008952422 | 121.556 | 5287 | ZINC000059725637 | 111.474 |
| 1925 | ZINC000012496319 | 72.4966 | 5288 | ZINC000004654657 | 113.705 |
| 1926 | ZINC000012661886 | 111.217 | 5289 | ZINC000003861373 | 111.434 |
| 1927 | ZINC000004097991 | 99.8725 | 5290 | ZINC000003897051 | 95.3922 |
| 1928 | ZINC000004098178 | 104.622 | 5291 | ZINC000004023795 | 122.386 |
| 1929 | ZINC000004098662 | 107.526 | 5292 | ZINC000004063367 | 112.535 |
| 1930 | ZINC000004802492 | 134.28 | 5293 | ZINC000004213126 | 134.363 |
| 1931 | ZINC000004095731 | 136.567 | 5294 | ZINC000027644505 | 122.837 |
| 1932 | ZINC000004716567 | 139.164 | 5295 | ZINC000002560469 | 94.8824 |
| 1933 | ZINC000004521655 | 89.7123 | 5296 | ZINC000030725574 | 116.282 |
| 1934 | ZINC000004098238 | 126.886 | 5297 | ZINC000100777317 | 119.713 |
| 1935 | ZINC000005158854 | 113.434 | 5298 | ZINC000000901908 | 92.3489 |
| 1936 | ZINC000004273393 | 74.5868 | 5299 | ZINC000001530487 | 115.203 |
| 1937 | ZINC000004095719 | 104.877 | 5300 | ZINC000001531857 | 115.546 |
| 1938 | ZINC000004143932 | 127.665 | 5301 | ZINC000001706140 | 71.1776 |
| 1939 | ZINC000004098839 | 89.4941 | 5302 | ZINC000100776197 | 62.187 |
| 1940 | ZINC000004097893 | 121.003 | 5303 | ZINC000100778048 | 85.9185 |
| 1941 | ZINC000004098230 | 105.159 | 5304 | ZINC000000402670 | 115.071 |
| 1942 | ZINC000004975331 | 115.767 | 5305 | ZINC000000402673 | 119.716 |
| 1943 | ZINC000004887265 | 94.1137 | 5306 | ZINC000000712672 | 136.644 |
| 1944 | ZINC000005015081 | 106.311 | 5307 | ZINC000017176122 | 125.81 |
| 1945 | ZINC000004175322 | 88.6611 | 5308 | ZINC000018169010 | 123.025 |
| 1946 | ZINC000005195795 | 153.942 | 5309 | ZINC000019884529 | 94.4234 |
| 1947 | ZINC000000388656 | 135.687 | 5310 | ZINC000085874227 | 121.627 |
| 1948 | ZINC000001650576 | 107.188 | 5311 | ZINC000015121957 | 134.694 |
| 1949 | ZINC000002034671 | 97.7771 | 5312 | ZINC000015206910 | 104.296 |
| 1950 | ZINC000049244633 | 133.331 | 5313 | ZINC000100079140 | 79.9499 |
| 1951 | ZINC000062592242 | 78.5231 | 5314 | ZINC000014819147 | 129.427 |
| 1952 | ZINC000070454906 | 116.766 | 5315 | ZINC000014859827 | 120.125 |
| 1953 | ZINC000058563822 | 138.06 | 5316 | ZINC000004097594 | 95.7611 |
| 1954 | ZINC000053201251 | 103.295 | 5317 | ZINC000004097829 | 78.5076 |
| 1955 | ZINC000049793116 | 87.9768 | 5318 | ZINC000004758249 | 126.344 |
| 1956 | ZINC000066099337 | 111.235 | 5319 | ZINC000004097544 | 83.2395 |
| 1957 | ZINC000066100070 | 116.444 | 5320 | ZINC000004097827 | 78.1741 |
| 1958 | ZINC000049051501 | 119.649 | 5321 | ZINC000013533347 | 98.6829 |
| 1959 | ZINC000049112458 | 115.91 | 5322 | ZINC000003869593 | 68.8017 |
| 1960 | ZINC000057268646 | 92.2029 | 5323 | ZINC000004096580 | 119.526 |
| 1961 | ZINC000103555391 | 85.568 | 5324 | ZINC000004096580 | 125.412 |
| 1962 | ZINC000085638768 | 116.207 | 5325 | ZINC000070450896 | 127.284 |
| 1963 | ZINC000000058118 | 114.966 | 5326 | ZINC000040454340 | 51.4283 |
| 1964 | ZINC000001758808 | 91.87 | 5327 | ZINC000012496246 | 103.073 |
| 1965 | ZINC000001648289 | 78.3759 | 5328 | ZINC000002041113 | 74.1838 |
| 1966 | ZINC000015205244 | 65.7666 | 5329 | ZINC000002166787 | 89.7178 |
| 1967 | ZINC000005282658 | 91.5388 | 5330 | ZINC000003814418 | 99.5053 |
| 1968 | ZINC000000391894 | 126.911 | 5331 | ZINC000012495605 | 125.196 |
| 1969 | ZINC000001718715 | 91.6205 | 5332 | ZINC000001850904 | 94.1874 |
| 1970 | ZINC000000389516 | 79.6624 | 5333 | ZINC000001850996 | 99.8157 |
| 1971 | ZINC000000538242 | 62.483 | 5334 | ZINC000002558610 | 75.4889 |
| 1972 | ZINC000002019693 | 121.759 | 5335 | ZINC000002041112 | 77.6167 |
| 1973 | ZINC000006484599 | 135.215 | 5336 | ZINC000001850997 | 101.956 |
| 1974 | ZINC000006484598 | 134.984 | 5337 | ZINC000003897050 | 106.919 |
| 1975 | ZINC000006484601 | 137.194 | 5338 | ZINC000002037431 | 80.4036 |
| 1976 | ZINC000006484602 | 146.055 | 5339 | ZINC000002567598 | 91.587 |
| 1977 | ZINC000000105227 | 90.455 | 5340 | ZINC000085600895 | 91.697 |
| 1978 | ZINC000001692442 | 51.9418 | 5341 | ZINC000003881689 | 107.989 |
| 1979 | ZINC000000898539 | 110.111 | 5342 | ZINC000011535640 | 94.2286 |
| 1980 | ZINC000012359923 | 72.8096 | 5343 | ZINC000002018419 | 100.053 |
| 1981 | ZINC000000080829 | 69.449 | 5344 | ZINC000002040970 | 111.721 |
| 1982 | ZINC000003861058 | 96.699 | 5345 | ZINC000002045163 | 123.026 |
| 1983 | ZINC000044005644 | 115.138 | 5346 | ZINC000002041119 | 106.6 |
| 1984 | ZINC000044387596 | 123.284 | 5347 | ZINC000002015523 | 103.935 |
| 1985 | ZINC000012358879 | 115.394 | 5348 | ZINC000001850666 | 84.1275 |
| 1986 | ZINC000000901022 | 70.2924 | 5349 | ZINC000001583750 | 69.5459 |
| 1987 | ZINC000000901648 | 46.2957 | 5350 | ZINC000001532199 | 120.553 |
| 1988 | ZINC000003847505 | 122.54 | 5351 | ZINC000001697415 | 97.8825 |
| 1989 | ZINC000000226366 | 99.7048 | 5352 | ZINC000001697406 | 122.297 |
| 1990 | ZINC000000157527 | 72.5499 | 5353 | ZINC000001600957 | 102.188 |
| 1991 | ZINC000000391885 | 64.2599 | 5354 | ZINC000000519080 | 117.711 |
| 1992 | ZINC000002140511 | 83.2588 | 5355 | ZINC000001532200 | 118.261 |
| 1993 | ZINC000085340887 | 112.8 | 5356 | ZINC000001481919 | 109.401 |
| 1994 | ZINC000000057753 | 96.8085 | 5357 | ZINC000000968034 | 75.1616 |
| 1995 | ZINC000001530625 | 138.357 | 5358 | ZINC000000967800 | 92.3683 |
| 1996 | ZINC000000330136 | 83.2944 | 5359 | ZINC000000154688 | 55.4454 |
| 1997 | ZINC000000058126 | 92.9647 | 5360 | ZINC000001600115 | 91.7439 |
| 1998 | ZINC000000967566 | 69.1859 | 5361 | ZINC000002007585 | 75.5527 |
| 1999 | ZINC000000967597 | 91.0039 | 5362 | ZINC000002018420 | 101.586 |
| 2000 | ZINC000000967521 | 64.5485 | 5363 | ZINC000001850809 | 105.693 |
| 2001 | ZINC000000006226 | 80.0426 | 5364 | ZINC000001850414 | 78.4763 |
| 2002 | ZINC000000137884 | 114.018 | 5365 | ZINC000001712065 | 57.8468 |
| 2003 | ZINC000001035331 | 114.422 | 5366 | ZINC000001734352 | 97.5369 |
| 2004 | ZINC000003795819 | 123.242 | 5367 | ZINC000001850413 | 79.9377 |
| 2005 | ZINC000003814422 | 129.702 | 5368 | ZINC000001677819 | 106.281 |
| 2006 | ZINC000003881640 | 64.4045 | 5369 | ZINC000001529820 | 89.4845 |
| 2007 | ZINC000019875504 | 103.11 | 5370 | ZINC000001531550 | 113.124 |
| 2008 | ZINC000031165899 | 99.1854 | 5371 | ZINC000001677813 | 102.425 |
| 2009 | ZINC000031502517 | 122.453 | 5372 | ZINC000001677786 | 96.787 |
| 2010 | ZINC000031997771 | 129.273 | 5373 | ZINC000001697412 | 101.986 |
| 2011 | ZINC000031164702 | 136.07 | 5374 | ZINC000001584796 | 80.8705 |
| 2012 | ZINC000031167746 | 116.213 | 5375 | ZINC000000967809 | 89.4477 |
| 2013 | ZINC000002560611 | 66.7946 | 5376 | ZINC000000391156 | 80.3254 |
| 2014 | ZINC000001690036 | 73.2399 | 5377 | ZINC000005195071 | 82.3394 |
| 2015 | ZINC000001691037 | 97.1778 | 5378 | ZINC000004098372 | 90.4813 |
| 2016 | ZINC000001692500 | 87.5972 | 5379 | ZINC000002041118 | 109.633 |
| 2017 | ZINC000001693415 | 117.48 | 5380 | ZINC000001849681 | 89.3191 |
| 2018 | ZINC000001693630 | 91.0301 | 5381 | ZINC000001846610 | 87.8116 |
| 2019 | ZINC000001765484 | 74.7809 | 5382 | ZINC000001724775 | 100.889 |
| 2020 | ZINC000001529210 | 92.585 | 5383 | ZINC000014096289 | 96.859 |
| 2021 | ZINC000002566154 | 97.9256 | 5384 | ZINC000001692449 | 58.7972 |
| 2022 | ZINC000002560463 | 72.1008 | 5385 | ZINC000001665018 | 81.5419 |
| 2023 | ZINC000001531600 | 87.7008 | 5386 | ZINC000001677809 | 103.051 |
| 2024 | ZINC000001531601 | 88.5293 | 5387 | ZINC000000391154 | 89.4242 |
| 2025 | ZINC000001531618 | 83.8424 | 5388 | ZINC000001081096 | 91.0882 |
| 2026 | ZINC000001850007 | 80.6214 | 5389 | ZINC000001599726 | 77.9091 |
| 2027 | ZINC000000895323 | 77.073 | 5390 | ZINC000001555584 | 105.611 |
| 2028 | ZINC000000895404 | 108.004 | 5391 | ZINC000013319968 | 119.276 |
| 2029 | ZINC000001529847 | 103.652 | 5392 | ZINC000008552023 | 72.151 |
| 2030 | ZINC000001697400 | 70.2154 | 5393 | ZINC000005158342 | 82.4607 |
| 2031 | ZINC000001698517 | 73.5665 | 5394 | ZINC000004099108 | 81.2093 |
| 2032 | ZINC000001765815 | 121.462 | 5395 | ZINC000004097365 | 86.4827 |
| 2033 | ZINC000001849752 | 91.5358 | 5396 | ZINC000004521640 | 87.1476 |
| 2034 | ZINC000001719252 | 108.986 | 5397 | ZINC000002041270 | 107.033 |
| 2035 | ZINC000002566373 | 83.7914 | 5398 | ZINC000002007587 | 82.8065 |
| 2036 | ZINC000001850080 | 81.088 | 5399 | ZINC000002038282 | 86.4332 |
| 2037 | ZINC000001850303 | 79.8828 | 5400 | ZINC000002040990 | 87.8465 |
| 2038 | ZINC000001765488 | 77.1153 | 5401 | ZINC000001850094 | 100.726 |
| 2039 | ZINC000002522581 | 131.198 | 5402 | ZINC000001874393 | 112.109 |
| 2040 | ZINC000032151702 | 46.4402 | 5403 | ZINC000001850726 | 126.669 |
| 2041 | ZINC000032161939 | 51.9815 | 5404 | ZINC000001693210 | 97.5916 |
| 2042 | ZINC000032161943 | 54.6236 | 5405 | ZINC000001530385 | 77.4286 |
| 2043 | ZINC000032161937 | 55.7579 | 5406 | ZINC000001562157 | 79.7101 |
| 2044 | ZINC000032161941 | 54.1572 | 5407 | ZINC000001532681 | 47.2689 |
| 2045 | ZINC000004658605 | 65.4253 | 5408 | ZINC000001680392 | 58.242 |
| 2046 | ZINC000004658628 | 44.9362 | 5409 | ZINC000001677832 | 122.809 |
| 2047 | ZINC000004661833 | 93.9518 | 5410 | ZINC000000967814 | 90.1345 |
| 2048 | ZINC000004342580 | 69.1751 | 5411 | ZINC000000967817 | 87.3475 |
| 2049 | ZINC000004404468 | 82.2371 | 5412 | ZINC000000388674 | 88.5979 |
| 2050 | ZINC000004501351 | 46.6182 | 5413 | ZINC000015207393 | 103.991 |
| 2051 | ZINC000004521395 | 53.8852 | 5414 | ZINC000004099110 | 84.8015 |
| 2052 | ZINC000004521742 | 100.645 | 5415 | ZINC000004403544 | 109.456 |
| 2053 | ZINC000004528636 | 75.9993 | 5416 | ZINC000002559023 | 131.043 |
| 2054 | ZINC000016051666 | 125.161 | 5417 | ZINC000005158345 | 88.4963 |
| 2055 | ZINC000017176123 | 117.792 | 5418 | ZINC000002568345 | 91.0938 |
| 2056 | ZINC000004658570 | 47.4024 | 5419 | ZINC000003861397 | 96.5815 |
| 2057 | ZINC000002167283 | 78.7872 | 5420 | ZINC000002029577 | 109.176 |
| 2058 | ZINC000004352602 | 119.929 | 5421 | ZINC000002026960 | 78.9984 |
| 2059 | ZINC000004428526 | 120.771 | 5422 | ZINC000001850669 | 81.5629 |
| 2060 | ZINC000004528589 | 87.2411 | 5423 | ZINC000001850667 | 84.788 |
| 2061 | ZINC000002384562 | 95.8451 | 5424 | ZINC000001683670 | 69.1353 |
| 2062 | ZINC000000135199 | 93.333 | 5425 | ZINC000001674756 | 73.227 |
| 2063 | ZINC000002382899 | 69.341 | 5426 | ZINC000001680040 | 84.9299 |
| 2064 | ZINC000001848574 | 108.649 | 5427 | ZINC000001698307 | 85.5194 |
| 2065 | ZINC000002557905 | 125.083 | 5428 | ZINC000000391155 | 90.6142 |
| 2066 | ZINC000002560465 | 85.359 | 5429 | ZINC000001577275 | 86.4587 |
| 2067 | ZINC000001689463 | 92.3368 | 5430 | ZINC000002569302 | 110.127 |
| 2068 | ZINC000001690956 | 80.533 | 5431 | ZINC000002038283 | 88.3885 |
| 2069 | ZINC000001692473 | 73.4094 | 5432 | ZINC000002034403 | 56.6081 |
| 2070 | ZINC000001693358 | 66.2283 | 5433 | ZINC000002015871 | 47.2144 |
| 2071 | ZINC000001693597 | 85.1285 | 5434 | ZINC000002007583 | 85.124 |
| 2072 | ZINC000002566086 | 94.5429 | 5435 | ZINC000002007589 | 78.9324 |
| 2073 | ZINC000002516012 | 103.252 | 5436 | ZINC000001724776 | 103.229 |
| 2074 | ZINC000002510279 | 83.4547 | 5437 | ZINC000001849683 | 89.1189 |
| 2075 | ZINC000005761930 | 86.2117 | 5438 | ZINC000001531613 | 97.127 |
| 2076 | ZINC000001531693 | 127.16 | 5439 | ZINC000001591830 | 103.02 |
| 2077 | ZINC000001700007 | 84.1368 | 5440 | ZINC000001577282 | 92.6912 |
| 2078 | ZINC000001704769 | 102.383 | 5441 | ZINC000001561518 | 74.1907 |
| 2079 | ZINC000001687490 | 104.063 | 5442 | ZINC000001691281 | 109.829 |
| 2080 | ZINC000001690437 | 71.1833 | 5443 | ZINC000001575584 | 67.5891 |
| 2081 | ZINC000001691515 | 102.061 | 5444 | ZINC000000967552 | 106.282 |
| 2082 | ZINC000001693320 | 83.9626 | 5445 | ZINC000000901213 | 40.9247 |
| 2083 | ZINC000001693595 | 94.9813 | 5446 | ZINC000000967525 | 87.9857 |
| 2084 | ZINC000002584406 | 80.1069 | 5447 | ZINC000002567418 | 79.7746 |
| 2085 | ZINC000001532046 | 68.7517 | 5448 | ZINC000000056583 | 137.325 |
| 2086 | ZINC000004098840 | 139.044 | 5449 | ZINC000002522343 | 69.0855 |
| 2087 | ZINC000001849834 | 97.6598 | 5450 | ZINC000015120456 | 122.22 |
| 2088 | ZINC000001850029 | 96.0594 | 5451 | ZINC000000388762 | 83.5689 |
| 2089 | ZINC000015248714 | 105.626 | 5452 | ZINC000000396313 | 92.7392 |
| 2090 | ZINC000015256869 | 121.615 | 5453 | ZINC000000402227 | 107.644 |
| 2091 | ZINC000015263540 | 122.644 | 5454 | ZINC000000163730 | 71.6929 |
| 2092 | ZINC000015272295 | 68.4984 | 5455 | ZINC000000157077 | 86.5067 |
| 2093 | ZINC000016921576 | 111.083 | 5456 | ZINC000000157773 | 85.4463 |
| 2094 | ZINC000017175239 | 113.217 | 5457 | ZINC000000164379 | 65.0791 |
| 2095 | ZINC000018061098 | 120.675 | 5458 | ZINC000000157430 | 79.1586 |
| 2096 | ZINC000019850522 | 104.236 | 5459 | ZINC000077293282 | 107.56 |
| 2097 | ZINC000014588411 | 87.6081 | 5460 | ZINC000000394532 | 61.0956 |
| 2098 | ZINC000014591755 | 87.551 | 5461 | ZINC000000394916 | 109.461 |
| 2099 | ZINC000014591970 | 72.5529 | 5462 | ZINC000004025994 | 87.8918 |
| 2100 | ZINC000014612909 | 101.009 | 5463 | ZINC000000388077 | 72.4211 |
| 2101 | ZINC000014642684 | 120.071 | 5464 | ZINC000000388663 | 86.9983 |
| 2102 | ZINC000014652207 | 85.5 | 5465 | ZINC000000330141 | 78.0563 |
| 2103 | ZINC000014652286 | 110.037 | 5466 | ZINC000000388085 | 90.1235 |
| 2104 | ZINC000014658239 | 129.622 | 5467 | ZINC000001081321 | 99.3494 |
| 2105 | ZINC000014689153 | 127.885 | 5468 | ZINC000001081099 | 74.8757 |
| 2106 | ZINC000014728050 | 137.797 | 5469 | ZINC000000388659 | 142.207 |
| 2107 | ZINC000014757320 | 86.3824 | 5470 | ZINC000004216848 | 95.2833 |
| 2108 | ZINC000014758734 | 136.168 | 5471 | ZINC000001726301 | 72.3334 |
| 2109 | ZINC000014819291 | 144.309 | 5472 | ZINC000001850500 | 83.7376 |
| 2110 | ZINC000014820417 | 143.152 | 5473 | ZINC000002036789 | 111.261 |
| 2111 | ZINC000014859826 | 119.013 | 5474 | ZINC000001693319 | 92.022 |
| 2112 | ZINC000015120276 | 98.6338 | 5475 | ZINC000014779854 | 119.463 |
| 2113 | ZINC000013380510 | 95.1488 | 5476 | ZINC000024718305 | 70.5223 |
| 2114 | ZINC000013412576 | 93.9453 | 5477 | ZINC000000402911 | 117.85 |
| 2115 | ZINC000013437566 | 74.6377 | 5478 | ZINC000000402911 | 106.62 |
| 2116 | ZINC000013540498 | 101.326 | 5479 | ZINC000014658236 | 121.823 |
| 2117 | ZINC000013542964 | 128.954 | 5480 | ZINC000014652213 | 94.9324 |
| 2118 | ZINC000013545758 | 108.468 | 5481 | ZINC000014588994 | 84.9672 |
| 2119 | ZINC000013677763 | 114.925 | 5482 | ZINC000001672876 | 87.0353 |
| 2120 | ZINC000014438651 | 86.4218 | 5483 | ZINC000006661796 | 71.0939 |
| 2121 | ZINC000014444365 | 115.477 | 5484 | ZINC000001851029 | 82.5237 |
| 2122 | ZINC000014487641 | 93.2597 | 5485 | ZINC000000896625 | 80.7069 |
| 2123 | ZINC000014504541 | 106.698 | 5486 | ZINC000000968100 | 69.8797 |
| 2124 | ZINC000008214541 | 43.6547 | 5487 | ZINC000000968102 | 86.9089 |
| 2125 | ZINC000008616483 | 108.1 | 5488 | ZINC000000967796 | 82.6795 |
| 2126 | ZINC000012496105 | 91.2055 | 5489 | ZINC000003873647 | 69.4475 |
| 2127 | ZINC000012496767 | 91.9198 | 5490 | ZINC000001648324 | 100.283 |
| 2128 | ZINC000012502479 | 104.672 | 5491 | ZINC000003898703 | 130.352 |
| 2129 | ZINC000013130930 | 128.425 | 5492 | ZINC000003861206 | 46.32 |
| 2130 | ZINC000013136661 | 146.16 | 5493 | ZINC000001648505 | 89.4111 |
| 2131 | ZINC000013282308 | 105.24 | 5494 | ZINC000001631379 | 74.1918 |
| 2132 | ZINC000013302909 | 127.683 | 5495 | ZINC000001648317 | 95.8131 |
| 2133 | ZINC000006206699 | 150.133 | 5496 | ZINC000001640853 | 87.1388 |
| 2134 | ZINC000006030959 | 94.8863 | 5497 | ZINC000001641287 | 68.2077 |
| 2135 | ZINC000006037626 | 93.1771 | 5498 | ZINC000001648266 | 65.4665 |
| 2136 | ZINC000006067283 | 69.3856 | 5499 | ZINC000001653137 | 65.7938 |
| 2137 | ZINC000006206700 | 139.707 | 5500 | ZINC000000087959 | 95.4465 |
| 2138 | ZINC000006483435 | 135.433 | 5501 | ZINC000002120337 | 118.422 |
| 2139 | ZINC000004832996 | 106.298 | 5502 | ZINC000002123339 | 100.346 |
| 2140 | ZINC000005158468 | 116.156 | 5503 | ZINC000000088245 | 83.8481 |
| 2141 | ZINC000004721188 | 130.675 | 5504 | ZINC000038611839 | 46.327 |
| 2142 | ZINC000005138189 | 53.9638 | 5505 | ZINC000038338719 | 101.01 |
| 2143 | ZINC000004467880 | 71.0424 | 5506 | ZINC000038613534 | 86.7414 |
| 2144 | ZINC000026672172 | 67.1406 | 5507 | ZINC000038654420 | 125.909 |
| 2145 | ZINC000026673320 | 69.8019 | 5508 | ZINC000000073693 | 124.28 |
| 2146 | ZINC000015160861 | 84.8138 | 5509 | ZINC000038609494 | 76.8668 |
| 2147 | ZINC000015248711 | 110.712 | 5510 | ZINC000038642932 | 100.327 |
| 2148 | ZINC000015272291 | 67.7577 | 5511 | ZINC000038654406 | 132.123 |
| 2149 | ZINC000017860887 | 117.105 | 5512 | ZINC000038609497 | 77.8104 |
| 2150 | ZINC000018123143 | 131.435 | 5513 | ZINC000038654414 | 132.281 |
| 2151 | ZINC000021999709 | 111.164 | 5514 | ZINC000038139837 | 115.479 |
| 2152 | ZINC000014588413 | 89.6327 | 5515 | ZINC000059586951 | 78.853 |
| 2153 | ZINC000014591969 | 72.7468 | 5516 | ZINC000059587184 | 91.3986 |
| 2154 | ZINC000014610081 | 131.269 | 5517 | ZINC000002381588 | 115.929 |
| 2155 | ZINC000014651125 | 119.442 | 5518 | ZINC000002035935 | 100.771 |
| 2156 | ZINC000014652216 | 92.1557 | 5519 | ZINC000001995085 | 73.7257 |
| 2157 | ZINC000014658220 | 126.803 | 5520 | ZINC000001677812 | 96.8721 |
| 2158 | ZINC000014658375 | 106.799 | 5521 | ZINC000001697404 | 103.931 |
| 2159 | ZINC000014684254 | 85.8196 | 5522 | ZINC000001081211 | 90.4694 |
| 2160 | ZINC000014722314 | 116.238 | 5523 | ZINC000001320177 | 94.9607 |
| 2161 | ZINC000014728051 | 140.312 | 5524 | ZINC000000967582 | 66.7005 |
| 2162 | ZINC000014756841 | 91.5271 | 5525 | ZINC000002558675 | 90.0483 |
| 2163 | ZINC000014758732 | 133.174 | 5526 | ZINC000012153979 | 88.7984 |
| 2164 | ZINC000014759216 | 112.129 | 5527 | ZINC000033953242 | 78.5254 |
| 2165 | ZINC000014764385 | 104.626 | 5528 | ZINC000001063075 | 80.1236 |
| 2166 | ZINC000014807241 | 99.072 | 5529 | ZINC000000391977 | 130.822 |
| 2167 | ZINC000014819293 | 146.585 | 5530 | ZINC000000392595 | 83.741 |
| 2168 | ZINC000014820413 | 139.424 | 5531 | ZINC000000404390 | 67.4064 |
| 2169 | ZINC000014820468 | 129.191 | 5532 | ZINC000000899863 | 107.091 |
| 2170 | ZINC000014859832 | 112.305 | 5533 | ZINC000003881445 | 70.7652 |
| 2171 | ZINC000015121415 | 101.385 | 5534 | ZINC000003881977 | 111.457 |
| 2172 | ZINC000013374377 | 92.0862 | 5535 | ZINC000000968269 | 68.9386 |
| 2173 | ZINC000013380829 | 95.5862 | 5536 | ZINC000003875629 | 78.3157 |
| 2174 | ZINC000013382496 | 98.437 | 5537 | ZINC000003875730 | 85.7522 |
| 2175 | ZINC000013412520 | 108.772 | 5538 | ZINC000003875920 | 77.6977 |
| 2176 | ZINC000013480221 | 73.8602 | 5539 | ZINC000003881456 | 86.2036 |
| 2177 | ZINC000014438723 | 103.698 | 5540 | ZINC000000967595 | 88.828 |
| 2178 | ZINC000014487643 | 92.1509 | 5541 | ZINC000002037591 | 65.1855 |
| 2179 | ZINC000008613400 | 89.3635 | 5542 | ZINC000002031414 | 107.767 |
| 2180 | ZINC000013109226 | 133.504 | 5543 | ZINC000002041035 | 98.5606 |
| 2181 | ZINC000013208862 | 109.533 | 5544 | ZINC000002045104 | 101.971 |
| 2182 | ZINC000013303598 | 119.553 | 5545 | ZINC000001851023 | 92.7756 |
| 2183 | ZINC000013305956 | 66.2391 | 5546 | ZINC000001850806 | 76.5664 |
| 2184 | ZINC000013307107 | 71.2921 | 5547 | ZINC000001850986 | 101.818 |
| 2185 | ZINC000013340290 | 99.1452 | 5548 | ZINC000001697340 | 104.705 |
| 2186 | ZINC000001850401 | 68.3425 | 5549 | ZINC000001599725 | 78.2089 |
| 2187 | ZINC000004429301 | 79.1324 | 5550 | ZINC000001529819 | 89.1016 |
| 2188 | ZINC000006031075 | 94.857 | 5551 | ZINC000001566492 | 90.4319 |
| 2189 | ZINC000006032061 | 69.9479 | 5552 | ZINC000001699876 | 46.7525 |
| 2190 | ZINC000006037706 | 91.0665 | 5553 | ZINC000001665012 | 80.7195 |
| 2191 | ZINC000006067790 | 87.7769 | 5554 | ZINC000001530356 | 50.6495 |
| 2192 | ZINC000006030496 | 79.0186 | 5555 | ZINC000000967810 | 86.62 |
| 2193 | ZINC000006090917 | 119.453 | 5556 | ZINC000000967813 | 80.9959 |
| 2194 | ZINC000006094144 | 121.089 | 5557 | ZINC000000967816 | 84.0564 |
| 2195 | ZINC000005132893 | 62.1692 | 5558 | ZINC000000968289 | 86.8279 |
| 2196 | ZINC000005964150 | 119.677 | 5559 | ZINC000002571348 | 84.2812 |
| 2197 | ZINC000005998641 | 127.388 | 5560 | ZINC000012153978 | 81.1204 |
| 2198 | ZINC000006017736 | 101.878 | 5561 | ZINC000012153977 | 85.1601 |
| 2199 | ZINC000005157963 | 67.1226 | 5562 | ZINC000000404256 | 127.994 |
| 2200 | ZINC000005964153 | 103.962 | 5563 | ZINC000000404268 | 66.3358 |
| 2201 | ZINC000005999143 | 79.3398 | 5564 | ZINC000000404382 | 78.4278 |
| 2202 | ZINC000031157838 | 100.403 | 5565 | ZINC000000404765 | 62.5063 |
| 2203 | ZINC000030725719 | 140.785 | 5566 | ZINC000001607785 | 75.7245 |
| 2204 | ZINC000003598530 | 90.4468 | 5567 | ZINC000001608669 | 130.827 |
| 2205 | ZINC000000899938 | 138.503 | 5568 | ZINC000001613603 | 92.8527 |
| 2206 | ZINC000001850481 | 69.9077 | 5569 | ZINC000003827362 | 120.06 |
| 2207 | ZINC000000899558 | 115.888 | 5570 | ZINC000028971866 | 110.779 |
| 2208 | ZINC000000402709 | 112.808 | 5571 | ZINC000002566769 | 116.702 |
| 2209 | ZINC000100779031 | 86.5199 | 5572 | ZINC000002566769 | 119.141 |
| 2210 | ZINC000001727527 | 139.301 | 5573 | ZINC000004027543 | 88.1523 |
| 2211 | ZINC000001563568 | 92.6206 | 5574 | ZINC000001677096 | 93.0091 |
| 2212 | ZINC000001684890 | 109.389 | 5575 | ZINC000001602836 | 74.0923 |
| 2213 | ZINC000000851689 | 82.7736 | 5576 | ZINC000014918951 | 125.394 |
| 2214 | ZINC000001626974 | 78.8398 | 5577 | ZINC000003861695 | 87.4627 |
| 2215 | ZINC000002564119 | 99.4623 | 5578 | ZINC000000404381 | 77.4523 |
| 2216 | ZINC000000156635 | 85.7271 | 5579 | ZINC000000404383 | 80.0497 |
| 2217 | ZINC000001618130 | 108.877 | 5580 | ZINC000036431114 | 106.981 |
| 2218 | ZINC000002562337 | 112.683 | 5581 | ZINC000070455110 | 94.4047 |
| 2219 | ZINC000000899920 | 133.018 | 5582 | ZINC000004202722 | 67.0844 |
| 2220 | ZINC000001711825 | 98.575 | 5583 | ZINC000000153137 | 62.769 |
| 2221 | ZINC000001660013 | 78.7021 | 5584 | ZINC000011535715 | 93.2269 |
| 2222 | ZINC000002561256 | 138.292 | 5585 | ZINC000001648316 | 91.2478 |
| 2223 | ZINC000001634344 | 152.394 | 5586 | ZINC000001641187 | 58.5121 |
| 2224 | ZINC000001620282 | 135.816 | 5587 | ZINC000018847046 | 120.538 |
| 2225 | ZINC000000900143 | 117.254 | 5588 | ZINC000003861733 | 112.677 |
| 2226 | ZINC000001532816 | 86.8799 | 5589 | ZINC000001677799 | 99.9931 |
| 2227 | ZINC000002334300 | 99.8602 | 5590 | ZINC000001574315 | 64.2583 |
| 2228 | ZINC000100824325 | 97.0289 | 5591 | ZINC000090416377 | 64.6811 |
| 2229 | ZINC000001580553 | 93.0137 | 5592 | ZINC000090756079 | 103.13 |
| 2230 | ZINC000004097193 | 123.419 | 5593 | ZINC000095618208 | 96.9525 |
| 2231 | ZINC000100823363 | 82.6234 | 5594 | ZINC000095620577 | 103.308 |
| 2232 | ZINC000001620280 | 142.727 | 5595 | ZINC000096085903 | 123.334 |
| 2233 | ZINC000001673578 | 94.0812 | 5596 | ZINC000086047262 | 100.473 |
| 2234 | ZINC000100823357 | 84.0098 | 5597 | ZINC000090416378 | 64.3564 |
| 2235 | ZINC000000900047 | 138.027 | 5598 | ZINC000095617487 | 118.159 |
| 2236 | ZINC000001715027 | 88.7302 | 5599 | ZINC000095617487 | 121.188 |
| 2237 | ZINC000001668238 | 86.4824 | 5600 | ZINC000095618247 | 121.036 |
| 2238 | ZINC000000899567 | 127.63 | 5601 | ZINC000095620578 | 99.9794 |
| 2239 | ZINC000100823364 | 84.8878 | 5602 | ZINC000003472623 | 93.4911 |
| 2240 | ZINC000001575028 | 137.052 | 5603 | ZINC000003472623 | 89.8624 |
| 2241 | ZINC000001626973 | 82.704 | 5604 | ZINC000000152265 | 69.0406 |
| 2242 | ZINC000100055842 | 98.7202 | 5605 | ZINC000001850860 | 102.802 |
| 2243 | ZINC000100823358 | 82.2991 | 5606 | ZINC000003860608 | 62.6477 |
| 2244 | ZINC000001593013 | 99.719 | 5607 | ZINC000003860693 | 47.4472 |
| 2245 | ZINC000003897049 | 99.7503 | 5608 | ZINC000003860797 | 47.8121 |
| 2246 | ZINC000001647160 | 65.3237 | 5609 | ZINC000003860956 | 114.267 |
| 2247 | ZINC000000898283 | 122.75 | 5610 | ZINC000003861280 | 85.9429 |
| 2248 | ZINC000001850005 | 81.4777 | 5611 | ZINC000003861550 | 119.441 |
| 2249 | ZINC000000899797 | 152.871 | 5612 | ZINC000003861736 | 96.4168 |
| 2250 | ZINC000000517336 | 96.8836 | 5613 | ZINC000001615324 | 67.9645 |
| 2251 | ZINC000002077712 | 119.062 | 5614 | ZINC000001677815 | 110.731 |
| 2252 | ZINC000000895803 | 95.8741 | 5615 | ZINC000003881431 | 73.9468 |
| 2253 | ZINC000001726300 | 88.1291 | 5616 | ZINC000000967570 | 66.8194 |
| 2254 | ZINC000002149675 | 128.629 | 5617 | ZINC000001658753 | 58.6371 |
| 2255 | ZINC000002149675 | 124.924 | 5618 | ZINC000001638432 | 93.5509 |
| 2256 | ZINC000004096681 | 126.978 | 5619 | ZINC000001644076 | 109.507 |
| 2257 | ZINC000003979023 | 116.573 | 5620 | ZINC000001648262 | 56.1573 |
| 2258 | ZINC000004096445 | 129.118 | 5621 | ZINC000001648312 | 85.8731 |
| 2259 | ZINC000004096811 | 125.965 | 5622 | ZINC000085880350 | 91.2665 |
| 2260 | ZINC000003979024 | 105.186 | 5623 | ZINC000005359641 | 105.143 |
| 2261 | ZINC000001692499 | 86.708 | 5624 | ZINC000005319055 | 136.202 |
| 2262 | ZINC000001693359 | 75.1848 | 5625 | ZINC000000056963 | 88.318 |
| 2263 | ZINC000001693629 | 92.916 | 5626 | ZINC000005976902 | 130.348 |
| 2264 | ZINC000000057361 | 101.63 | 5627 | ZINC000000057505 | 119.896 |
| 2265 | ZINC000000388657 | 149.127 | 5628 | ZINC000000057675 | 116.02 |
| 2266 | ZINC000000899200 | 130.408 | 5629 | ZINC000005082886 | 98.8891 |
| 2267 | ZINC000001026053 | 117.729 | 5630 | ZINC000001699884 | 82.356 |
| 2268 | ZINC000001531872 | 136.919 | 5631 | ZINC000001703461 | 121.663 |
| 2269 | ZINC000001848538 | 91.2479 | 5632 | ZINC000001706746 | 79.2642 |
| 2270 | ZINC000000896129 | 92.3323 | 5633 | ZINC000001708386 | 104.057 |
| 2271 | ZINC000003995410 | 130.882 | 5634 | ZINC000001680382 | 87.9295 |
| 2272 | ZINC000000967532 | 52.9898 | 5635 | ZINC000001680438 | 101.685 |
| 2273 | ZINC000005373275 | 105.166 | 5636 | ZINC000001680640 | 97.1934 |
| 2274 | ZINC000013377888 | 101.526 | 5637 | ZINC000001680784 | 81.181 |
| 2275 | ZINC000001850834 | 75.5317 | 5638 | ZINC000001723266 | 76.5593 |
| 2276 | ZINC000003861281 | 83.8723 | 5639 | ZINC000001729185 | 95.4895 |
| 2277 | ZINC000036351948 | 117.691 | 5640 | ZINC000001866952 | 46.1721 |
| 2278 | ZINC000001672808 | 74.5246 | 5641 | ZINC000005820872 | 87.9419 |
| 2279 | ZINC000001602584 | 73.6764 | 5642 | ZINC000001712308 | 64.0403 |
| 2280 | ZINC000001667585 | 86.2116 | 5643 | ZINC000005837711 | 70.0601 |
| 2281 | ZINC000000895443 | 89.6404 | 5644 | ZINC000005837961 | 72.7819 |
| 2282 | ZINC000001850818 | 101.994 | 5645 | ZINC000001532098 | 130.082 |
| 2283 | ZINC000000895269 | 74.3605 | 5646 | ZINC000001849800 | 86.9859 |
| 2284 | ZINC000001850790 | 111.9 | 5647 | ZINC000000895226 | 49.9356 |
| 2285 | ZINC000001591828 | 103.915 | 5648 | ZINC000000895304 | 55.9068 |
| 2286 | ZINC000001596590 | 92.6699 | 5649 | ZINC000001693667 | 97.4859 |
| 2287 | ZINC000001599553 | 78.2491 | 5650 | ZINC000005821005 | 85.5801 |
| 2288 | ZINC000001595727 | 93.2162 | 5651 | ZINC000005822039 | 89.289 |
| 2289 | ZINC000001663926 | 73.1548 | 5652 | ZINC000005161652 | 88.6205 |
| 2290 | ZINC000001672954 | 90.1509 | 5653 | ZINC000000001551 | 126.159 |
| 2291 | ZINC000000968247 | 88.0868 | 5654 | ZINC000001850393 | 106.965 |
| 2292 | ZINC000001665919 | 105.313 | 5655 | ZINC000001849803 | 103.003 |
| 2293 | ZINC000001670361 | 87.737 | 5656 | ZINC000001850068 | 90.8985 |
| 2294 | ZINC000001666987 | 56.8943 | 5657 | ZINC000001850304 | 80.11 |
| 2295 | ZINC000001667603 | 55.8016 | 5658 | ZINC000001850492 | 96.0703 |
| 2296 | ZINC000001672809 | 96.889 | 5659 | ZINC000001532641 | 85.4604 |
| 2297 | ZINC000001633876 | 93.9477 | 5660 | ZINC000003869856 | 120.055 |
| 2298 | ZINC000000409361 | 76.0988 | 5661 | ZINC000003869856 | 124.245 |
| 2299 | ZINC000000895412 | 54.8141 | 5662 | ZINC000002574224 | 95.0218 |
| 2300 | ZINC000001997926 | 82.3973 | 5663 | ZINC000002575203 | 80.4634 |
| 2301 | ZINC000001565389 | 89.9495 | 5664 | ZINC000004342579 | 68.0807 |
| 2302 | ZINC000038321878 | 122.531 | 5665 | ZINC000004352598 | 118.644 |
| 2303 | ZINC000040880628 | 116.825 | 5666 | ZINC000004501350 | 48.04 |
| 2304 | ZINC000005821721 | 87.5242 | 5667 | ZINC000004528575 | 58.5985 |
| 2305 | ZINC000001532070 | 91.0069 | 5668 | ZINC000004655105 | 128.415 |
| 2306 | ZINC000001532162 | 76.4687 | 5669 | ZINC000002164190 | 90.0369 |
| 2307 | ZINC000001529769 | 90.2471 | 5670 | ZINC000002164694 | 105.179 |
| 2308 | ZINC000005854698 | 122.415 | 5671 | ZINC000004529159 | 74.386 |
| 2309 | ZINC000005225061 | 77.9107 | 5672 | ZINC000004528592 | 87.233 |
| 2310 | ZINC000005224866 | 75.5986 | 5673 | ZINC000004642624 | 105.309 |
| 2311 | ZINC000000152996 | 75.6467 | 5674 | ZINC000002165950 | 101.31 |
| 2312 | ZINC000000154698 | 75.2625 | 5675 | ZINC000004521557 | 63.5837 |
| 2313 | ZINC000034955329 | 51.1838 | 5676 | ZINC000004528554 | 62.647 |
| 2314 | ZINC000035455581 | 100.939 | 5677 | ZINC000002527917 | 91.3664 |
| 2315 | ZINC000002509755 | 120.315 | 5678 | ZINC000001529195 | 47.2512 |
| 2316 | ZINC000002526389 | 116.969 | 5679 | ZINC000001529206 | 87.4146 |
| 2317 | ZINC000003870515 | 84.3536 | 5680 | ZINC000001504382 | 101.318 |
| 2318 | ZINC000017971067 | 73.3423 | 5681 | ZINC000002557414 | 142.179 |
| 2319 | ZINC000169743072 | 55.3934 | 5682 | ZINC000002548258 | 81.0975 |
| 2320 | ZINC000000001601 | 119.992 | 5683 | ZINC000000388593 | 87.454 |
| 2321 | ZINC000000001601 | 115.641 | 5684 | ZINC000002015539 | 61.9435 |
| 2322 | ZINC000000839083 | 148.915 | 5685 | ZINC000001866965 | 60.6106 |
| 2323 | ZINC000000839083 | 146.413 | 5686 | ZINC000000896726 | 66.6945 |
| 2324 | ZINC000022061255 | 122.088 | 5687 | ZINC000000897129 | 56.9948 |
| 2325 | ZINC000022061261 | 119.167 | 5688 | ZINC000001850763 | 82.6701 |
| 2326 | ZINC000002526388 | 118.399 | 5689 | ZINC000017327670 | 43.7717 |
| 2327 | ZINC000002528510 | 83.1538 | 5690 | ZINC000032840893 | 105.805 |
| 2328 | ZINC000003870514 | 82.3358 | 5691 | ZINC000002015645 | 71.1626 |
| 2329 | ZINC000000001684 | 78.0367 | 5692 | ZINC000000391550 | 97.8722 |
| 2330 | ZINC000013449412 | 93.6944 | 5693 | ZINC000002002632 | 103.272 |
| 2331 | ZINC000021981288 | 155.756 | 5694 | ZINC000002011489 | 81.7219 |
| 2332 | ZINC000021981288 | 144.026 | 5695 | ZINC000002014873 | 55.726 |
| 2333 | ZINC000021981290 | 147.087 | 5696 | ZINC000006031700 | 92.8233 |
| 2334 | ZINC000021981290 | 140.114 | 5697 | ZINC000006067031 | 92.0931 |
| 2335 | ZINC000006072259 | 95.7576 | 5698 | ZINC000005764361 | 93.5692 |
| 2336 | ZINC000008214519 | 103.607 | 5699 | ZINC000006017718 | 124.37 |
| 2337 | ZINC000004743888 | 104.888 | 5700 | ZINC000006031812 | 95.2573 |
| 2338 | ZINC000008214699 | 45.6278 | 5701 | ZINC000006032238 | 130.606 |
| 2339 | ZINC000001849759 | 111.542 | 5702 | ZINC000006066878 | 95.5902 |
| 2340 | ZINC000000038545 | 107.428 | 5703 | ZINC000000388192 | 74.2398 |
| 2341 | ZINC000013449409 | 131.202 | 5704 | ZINC000028568062 | 136.316 |
| 2342 | ZINC000009133172 | 145.989 | 5705 | ZINC000001849753 | 98.1865 |
| 2343 | ZINC000009133172 | 154.488 | 5706 | ZINC000013456555 | 100.641 |
| 2344 | ZINC000000388234 | 69.963 | 5707 | ZINC000003869972 | 84.0685 |
| 2345 | ZINC000000347124 | 78.8476 | 5708 | ZINC000003869972 | 85.7756 |
| 2346 | ZINC000001081581 | 124.843 | 5709 | ZINC000012358735 | 87.704 |
| 2347 | ZINC000001621671 | 135.502 | 5710 | ZINC000003869684 | 114.493 |
| 2348 | ZINC000000398630 | 60.4063 | 5711 | ZINC000001850854 | 75.5468 |
| 2349 | ZINC000003874585 | 155.264 | 5712 | ZINC000000403038 | 107.784 |
| 2350 | ZINC000034275400 | 103.296 | 5713 | ZINC000003881404 | 80.9955 |
| 2351 | ZINC000034257026 | 57.5444 | 5714 | ZINC000000968037 | 79.2346 |
| 2352 | ZINC000034257028 | 59.1842 | 5715 | ZINC000003860327 | 86.6297 |
| 2353 | ZINC000000388357 | 79.1679 | 5716 | ZINC000003860474 | 61.2135 |
| 2354 | ZINC000001683674 | 80.5592 | 5717 | ZINC000003860604 | 62.0321 |
| 2355 | ZINC000001699425 | 74.6535 | 5718 | ZINC000003860903 | 95.7836 |
| 2356 | ZINC000001699905 | 68.4295 | 5719 | ZINC000003861086 | 68.6398 |
| 2357 | ZINC000001706892 | 82.3093 | 5720 | ZINC000003861263 | 80.9864 |
| 2358 | ZINC000001699947 | 52.957 | 5721 | ZINC000000967562 | 80.5977 |
| 2359 | ZINC000001707856 | 67.114 | 5722 | ZINC000001658458 | 146.55 |
| 2360 | ZINC000001681500 | 94.7143 | 5723 | ZINC000000394644 | 83.8875 |
| 2361 | ZINC000002003566 | 73.7751 | 5724 | ZINC000000394998 | 103.174 |
| 2362 | ZINC000018275062 | 118.755 | 5725 | ZINC000000158751 | 62.1381 |
| 2363 | ZINC000033833583 | 103.905 | 5726 | ZINC000000163290 | 73.3622 |
| 2364 | ZINC000002575486 | 77.3316 | 5727 | ZINC000001673034 | 66.1905 |
| 2365 | ZINC000000967630 | 87.8068 | 5728 | ZINC000008234283 | 86.9081 |
| 2366 | ZINC000000895559 | 85.7647 | 5729 | ZINC000000001287 | 73.5599 |
| 2367 | ZINC000017969422 | 102.502 | 5730 | ZINC000000968230 | 67.5534 |
| 2368 | ZINC000017969422 | 110.271 | 5731 | ZINC000006483503 | 106.559 |
| 2369 | ZINC000118912814 | 109.465 | 5732 | ZINC000006483503 | 103.103 |
| 2370 | ZINC000000525655 | 107.364 | 5733 | ZINC000006437922 | 70.8939 |
| 2371 | ZINC000000517337 | 105.625 | 5734 | ZINC000000153666 | 73.3306 |
| 2372 | ZINC000001081121 | 81.413 | 5735 | ZINC000006481415 | 71.6624 |
| 2373 | ZINC000003599462 | 154.249 | 5736 | ZINC000000389873 | 107.267 |
| 2374 | ZINC000000519621 | 122.219 | 5737 | ZINC000005736008 | 66.7521 |
| 2375 | ZINC000000519621 | 119.748 | 5738 | ZINC000078566363 | 36.5902 |
| 2376 | ZINC000004098623 | 131.065 | 5739 | ZINC000000899675 | 97.6038 |
| 2377 | ZINC000004098527 | 123.177 | 5740 | ZINC000006484014 | 129.681 |
| 2378 | ZINC000004097950 | 97.3468 | 5741 | ZINC000000157062 | 75.3517 |
| 2379 | ZINC000004098580 | 83.9664 | 5742 | ZINC000005752223 | 73.2056 |
| 2380 | ZINC000003979026 | 78.7939 | 5743 | ZINC000004654623 | 142.681 |
| 2381 | ZINC000004096264 | 74.3 | 5744 | ZINC000003881598 | 102.514 |
| 2382 | ZINC000004096200 | 83.0164 | 5745 | ZINC000000899907 | 133.433 |
| 2383 | ZINC000001558484 | 101.464 | 5746 | ZINC000004098740 | 139.097 |
| 2384 | ZINC000002030715 | 132.802 | 5747 | ZINC000001999286 | 124.223 |
| 2385 | ZINC000000265434 | 111.225 | 5748 | ZINC000001715917 | 98.3219 |
| 2386 | ZINC000001845768 | 104.42 | 5749 | ZINC000004098416 | 84.3535 |
| 2387 | ZINC000002017391 | 78.529 | 5750 | ZINC000002002060 | 85.6624 |
| 2388 | ZINC000030724298 | 145.486 | 5751 | ZINC000001680788 | 87.9568 |
| 2389 | ZINC000003814413 | 123.663 | 5752 | ZINC000033606582 | 105.647 |
| 2390 | ZINC000002569298 | 68.597 | 5753 | ZINC000001684870 | 91.0355 |
| 2391 | ZINC000003604261 | 122.65 | 5754 | ZINC000004802714 | 65.9651 |
| 2392 | ZINC000003604263 | 120.031 | 5755 | ZINC000002015857 | 86.3144 |
| 2393 | ZINC000003648307 | 146.079 | 5756 | ZINC000000056481 | 83.0534 |
| 2394 | ZINC000002555622 | 99.4352 | 5757 | ZINC000001532803 | 49.1124 |
| 2395 | ZINC000002513716 | 123.132 | 5758 | ZINC000001577077 | 48.8505 |
| 2396 | ZINC000002507487 | 114.264 | 5759 | ZINC000000409329 | 84.7306 |
| 2397 | ZINC000003881403 | 119.548 | 5760 | ZINC000000406908 | 74.3618 |
| 2398 | ZINC000003979027 | 108.69 | 5761 | ZINC000001996067 | 95.5142 |
| 2399 | ZINC000001850679 | 61.8315 | 5762 | ZINC000000035527 | 135.489 |
| 2400 | ZINC000100297478 | 117.712 | 5763 | ZINC000000391815 | 85.6212 |
| 2401 | ZINC000002024479 | 78.2471 | 5764 | ZINC000000491073 | 107.201 |
| 2402 | ZINC000100779034 | 85.508 | 5765 | ZINC000002583437 | 92.7011 |
| 2403 | ZINC000001850785 | 90.3303 | 5766 | ZINC000001850881 | 98.0643 |
| 2404 | ZINC000000851690 | 81.89 | 5767 | ZINC000002558114 | 88.8014 |
| 2405 | ZINC000000898340 | 109.896 | 5768 | ZINC000002560466 | 93.3574 |
| 2406 | ZINC000001850165 | 107.096 | 5769 | ZINC000002044619 | 102.013 |
| 2407 | ZINC000000000566 | 94.6937 | 5770 | ZINC000000058257 | 97.1044 |
| 2408 | ZINC000016051887 | 106.221 | 5771 | ZINC000000056460 | 92.6643 |
| 2409 | ZINC000031161457 | 106.985 | 5772 | ZINC000005179611 | 90.569 |
| 2410 | ZINC000031157853 | 81.4181 | 5773 | ZINC000005103851 | 94.8869 |
| 2411 | ZINC000031158247 | 106.876 | 5774 | ZINC000005966178 | 69.3229 |
| 2412 | ZINC000029134496 | 111.298 | 5775 | ZINC000004262348 | 68.0013 |
| 2413 | ZINC000029134692 | 130.308 | 5776 | ZINC000004262577 | 93.2187 |
| 2414 | ZINC000031155701 | 128.086 | 5777 | ZINC000005134501 | 70.8696 |
| 2415 | ZINC000029042204 | 102.269 | 5778 | ZINC000005116778 | 97.2595 |
| 2416 | ZINC000000898179 | 122.57 | 5779 | ZINC000005103853 | 104.305 |
| 2417 | ZINC000031157848 | 85.6318 | 5780 | ZINC000012405245 | 122.251 |
| 2418 | ZINC000022066528 | 137.558 | 5781 | ZINC000000338331 | 110.174 |
| 2419 | ZINC000001531846 | 125.773 | 5782 | ZINC000002564122 | 108.269 |
| 2420 | ZINC000001608542 | 95.12 | 5783 | ZINC000001693633 | 92.9511 |
| 2421 | ZINC000000155364 | 70.6641 | 5784 | ZINC000002573891 | 92.3592 |
| 2422 | ZINC000000388238 | 94.7524 | 5785 | ZINC000001689826 | 88.4214 |
| 2423 | ZINC000001683666 | 59.2713 | 5786 | ZINC000001698309 | 90.8862 |
| 2424 | ZINC000001684719 | 86.4549 | 5787 | ZINC000001699445 | 86.7244 |
| 2425 | ZINC000002510213 | 73.1728 | 5788 | ZINC000001687155 | 47.4509 |
| 2426 | ZINC000001747833 | 81.5797 | 5789 | ZINC000001690291 | 54.0794 |
| 2427 | ZINC000001758084 | 91.4472 | 5790 | ZINC000001565363 | 100.128 |
| 2428 | ZINC000002569925 | 83.8569 | 5791 | ZINC000003814414 | 119.613 |
| 2429 | ZINC000002510288 | 73.4904 | 5792 | ZINC000001849646 | 78.8788 |
| 2430 | ZINC000118913143 | 110.967 | 5793 | ZINC000001850504 | 80.9408 |
| 2431 | ZINC000002522807 | 73.635 | 5794 | ZINC000001850547 | 101.869 |
| 2432 | ZINC000002504530 | 79.9827 | 5795 | ZINC000001850619 | 87.2289 |
| 2433 | ZINC000001702514 | 100.969 | 5796 | ZINC000001850403 | 70.4524 |
| 2434 | ZINC000001619742 | 96.5368 | 5797 | ZINC000001849801 | 103.078 |
| 2435 | ZINC000001872258 | 152.529 | 5798 | ZINC000002557900 | 94.3548 |
| 2436 | ZINC000001562070 | 113.161 | 5799 | ZINC000003814412 | 124.762 |
| 2437 | ZINC000001604019 | 121.906 | 5800 | ZINC000002577993 | 90.3875 |
| 2438 | ZINC000001531806 | 104.604 | 5801 | ZINC000001530259 | 50.7712 |
| 2439 | ZINC000001651264 | 95.9595 | 5802 | ZINC000001530354 | 50.1514 |
| 2440 | ZINC000001689527 | 132.371 | 5803 | ZINC000005441112 | 96.3163 |
| 2441 | ZINC000001735195 | 98.45 | 5804 | ZINC000001532245 | 88.7584 |
| 2442 | ZINC000001761213 | 126.553 | 5805 | ZINC000001574322 | 83.3797 |
| 2443 | ZINC000001846642 | 89.589 | 5806 | ZINC000003830679 | 92.0654 |
| 2444 | ZINC000001863457 | 102.711 | 5807 | ZINC000001697402 | 99.7769 |
| 2445 | ZINC000001871615 | 120.403 | 5808 | ZINC000001697409 | 100.984 |
| 2446 | ZINC000002114142 | 95.8773 | 5809 | ZINC000001850792 | 113.877 |
| 2447 | ZINC000002145015 | 135.541 | 5810 | ZINC000001688155 | 78.7632 |
| 2448 | ZINC000000057657 | 131.294 | 5811 | ZINC000001693323 | 108.388 |
| 2449 | ZINC000000025672 | 114.297 | 5812 | ZINC000001693596 | 91.4753 |
| 2450 | ZINC000000895045 | 67.3543 | 5813 | ZINC000000895809 | 79.8391 |
| 2451 | ZINC000001537184 | 113.287 | 5814 | ZINC000000895906 | 95.6601 |
| 2452 | ZINC000001550030 | 125.053 | 5815 | ZINC000001586656 | 54.1179 |
| 2453 | ZINC000001622055 | 48.9474 | 5816 | ZINC000001586746 | 73.5383 |
| 2454 | ZINC000001850101 | 123.568 | 5817 | ZINC000001586769 | 105.338 |
| 2455 | ZINC000002006632 | 85.024 | 5818 | ZINC000001319891 | 139.536 |
| 2456 | ZINC000002562219 | 106.874 | 5819 | ZINC000001603220 | 71.2231 |
| 2457 | ZINC000014685555 | 95.0067 | 5820 | ZINC000000896404 | 75.92 |
| 2458 | ZINC000018286013 | 123.617 | 5821 | ZINC000000896814 | 78.3346 |
| 2459 | ZINC000001677793 | 99.0155 | 5822 | ZINC000000897147 | 91.9065 |
| 2460 | ZINC000001529768 | 88.1262 | 5823 | ZINC000001529222 | 74.7933 |
| 2461 | ZINC000000407006 | 65.4386 | 5824 | ZINC000001529270 | 89.1523 |
| 2462 | ZINC000001584052 | 109.224 | 5825 | ZINC000001529452 | 76.9777 |
| 2463 | ZINC000001850501 | 81.2512 | 5826 | ZINC000001529247 | 90.5924 |
| 2464 | ZINC000000895149 | 67.3386 | 5827 | ZINC000001529404 | 96.8116 |
| 2465 | ZINC000001850863 | 93.3043 | 5828 | ZINC000001586315 | 79.2121 |
| 2466 | ZINC000001718566 | 74.2785 | 5829 | ZINC000002556430 | 80.8237 |
| 2467 | ZINC000001718855 | 95.7088 | 5830 | ZINC000001708728 | 101.088 |
| 2468 | ZINC000001719255 | 102.054 | 5831 | ZINC000001849641 | 87.9712 |
| 2469 | ZINC000001694423 | 106.707 | 5832 | ZINC000000967563 | 92.4878 |
| 2470 | ZINC000001694745 | 100.314 | 5833 | ZINC000000967593 | 83.0214 |
| 2471 | ZINC000001695428 | 91.4414 | 5834 | ZINC000000137119 | 76.255 |
| 2472 | ZINC000001850730 | 104.179 | 5835 | ZINC000000145734 | 85.1635 |
| 2473 | ZINC000001561960 | 91.3622 | 5836 | ZINC000000144268 | 79.5497 |
| 2474 | ZINC000003815419 | 133.702 | 5837 | ZINC000001850827 | 93.6397 |
| 2475 | ZINC000002568158 | 86.5785 | 5838 | ZINC000003978454 | 99.0079 |
| 2476 | ZINC000001850757 | 95.2483 | 5839 | ZINC000003978454 | 98.5882 |
| 2477 | ZINC000000407092 | 81.7265 | 5840 | ZINC000003860973 | 110.66 |
| 2478 | ZINC000001621620 | 45.5298 | 5841 | ZINC000003860201 | 110.031 |
| 2479 | ZINC000001583673 | 73.7088 | 5842 | ZINC000002510059 | 89.7171 |
| 2480 | ZINC000001591842 | 98.6901 | 5843 | ZINC000118913144 | 106.163 |
| 2481 | ZINC000001597133 | 109.317 | 5844 | ZINC000118924831 | 110.24 |
| 2482 | ZINC000238730544 | 101.667 | 5845 | ZINC000001592388 | 63.533 |
| 2483 | ZINC000097922822 | 73.8213 | 5846 | ZINC000001680841 | 89.0108 |
| 2484 | ZINC000097056130 | 77.4134 | 5847 | ZINC000014765844 | 87.7294 |
| 2485 | ZINC000095630290 | 127.556 | 5848 | ZINC000014765842 | 87.3983 |
| 2486 | ZINC000095630292 | 121.501 | 5849 | ZINC000014765841 | 82.4194 |
| 2487 | ZINC000095911093 | 92.3592 | 5850 | ZINC000001673031 | 98.7182 |
| 2488 | ZINC000095630291 | 131.271 | 5851 | ZINC000001666982 | 90.1444 |
| 2489 | ZINC000095630293 | 129.14 | 5852 | ZINC000001677816 | 106.265 |
| 2490 | ZINC000095620526 | 83.2812 | 5853 | ZINC000001577197 | 69.5926 |
| 2491 | ZINC000230086290 | 99.1832 | 5854 | ZINC000001532732 | 53.0329 |
| 2492 | ZINC000095619682 | 110.152 | 5855 | ZINC000000967533 | 68.1675 |
| 2493 | ZINC000095619694 | 113.386 | 5856 | ZINC000000967579 | 72.5012 |
| 2494 | ZINC000095619732 | 101.233 | 5857 | ZINC000000967716 | 97.9719 |
| 2495 | ZINC000061389522 | 131.079 | 5858 | ZINC000001608665 | 123.389 |
| 2496 | ZINC000095619687 | 81.6836 | 5859 | ZINC000001609699 | 84.1083 |
| 2497 | ZINC000095619731 | 102.787 | 5860 | ZINC000001613340 | 85.3043 |
| 2498 | ZINC000059778294 | 62.9826 | 5861 | ZINC000000004935 | 126.115 |
| 2499 | ZINC000095618131 | 82.1167 | 5862 | ZINC000005103850 | 99.3502 |
| 2500 | ZINC000095618235 | 110.609 | 5863 | ZINC000005132507 | 80.2164 |
| 2501 | ZINC000095618468 | 117.105 | 5864 | ZINC000005196459 | 60.4306 |
| 2502 | ZINC000095619429 | 95.4741 | 5865 | ZINC000005225143 | 72.1199 |
| 2503 | ZINC000095619681 | 113.46 | 5866 | ZINC000005159232 | 67.3221 |
| 2504 | ZINC000095618467 | 106.602 | 5867 | ZINC000005178380 | 49.2197 |
| 2505 | ZINC000095617974 | 101.773 | 5868 | ZINC000005179610 | 88.3384 |
| 2506 | ZINC000095617973 | 103.899 | 5869 | ZINC000002048856 | 140.633 |
| 2507 | ZINC000001663619 | 90.0184 | 5870 | ZINC000005225057 | 78.3276 |
| 2508 | ZINC000001665023 | 96.4491 | 5871 | ZINC000000058119 | 132.116 |
| 2509 | ZINC000001667422 | 81.2913 | 5872 | ZINC000004097541 | 82.121 |
| 2510 | ZINC000001672941 | 73.3235 | 5873 | ZINC000004098346 | 73.0501 |
| 2511 | ZINC000086050393 | 86.4542 | 5874 | ZINC000005641300 | 107.215 |
| 2512 | ZINC000086050511 | 96.2003 | 5875 | ZINC000005641298 | 92.153 |
| 2513 | ZINC000050179659 | 81.3596 | 5876 | ZINC000002040440 | 82.3996 |
| 2514 | ZINC000086050396 | 86.8049 | 5877 | ZINC000002035981 | 91.4546 |
| 2515 | ZINC000053683696 | 90.788 | 5878 | ZINC000001530011 | 107.365 |
| 2516 | ZINC000059588101 | 97.1788 | 5879 | ZINC000002038974 | 102.63 |
| 2517 | ZINC000059724843 | 90.9082 | 5880 | ZINC000002013201 | 66.1596 |
| 2518 | ZINC000059257760 | 108.954 | 5881 | ZINC000002039832 | 132.359 |
| 2519 | ZINC000043618606 | 80.2119 | 5882 | ZINC000015113824 | 144.817 |
| 2520 | ZINC000044710259 | 96.4337 | 5883 | ZINC000002031573 | 68.0022 |
| 2521 | ZINC000043482217 | 102.131 | 5884 | ZINC000002034716 | 60.7442 |
| 2522 | ZINC000043482214 | 101.644 | 5885 | ZINC000004899706 | 75.0269 |
| 2523 | ZINC000044417879 | 97.9157 | 5886 | ZINC000002038848 | 92.8298 |
| 2524 | ZINC000043618605 | 77.1324 | 5887 | ZINC000004899513 | 105.575 |
| 2525 | ZINC000044710258 | 95.7443 | 5888 | ZINC000004098644 | 121.538 |
| 2526 | ZINC000001484626 | 46.6603 | 5889 | ZINC000001529191 | 67.3127 |
| 2527 | ZINC000035021147 | 73.6249 | 5890 | ZINC000002040174 | 91.6618 |
| 2528 | ZINC000039067134 | 66.1478 | 5891 | ZINC000002035951 | 57.584 |
| 2529 | ZINC000039167973 | 73.495 | 5892 | ZINC000002040186 | 69.6296 |
| 2530 | ZINC000040542580 | 99.9526 | 5893 | ZINC000002038642 | 73.6096 |
| 2531 | ZINC000042685596 | 101.624 | 5894 | ZINC000002038847 | 94.801 |
| 2532 | ZINC000085913296 | 107.496 | 5895 | ZINC000002039359 | 93.6175 |
| 2533 | ZINC000034676246 | 94.002 | 5896 | ZINC000000056472 | 138.01 |
| 2534 | ZINC000039090697 | 94.3905 | 5897 | ZINC000000056584 | 138.919 |
| 2535 | ZINC000039116380 | 89.7994 | 5898 | ZINC000002039834 | 115.939 |
| 2536 | ZINC000039224989 | 105.52 | 5899 | ZINC000000388187 | 68.149 |
| 2537 | ZINC000035653780 | 79.3091 | 5900 | ZINC000000967928 | 85.8103 |
| 2538 | ZINC000039069556 | 75.8104 | 5901 | ZINC000000967189 | 73.454 |
| 2539 | ZINC000039090696 | 98.0376 | 5902 | ZINC000000388291 | 75.8573 |
| 2540 | ZINC000043284710 | 123.833 | 5903 | ZINC000000338284 | 134.526 |
| 2541 | ZINC000035021146 | 73.8449 | 5904 | ZINC000000388067 | 80.6312 |
| 2542 | ZINC000039061874 | 77.421 | 5905 | ZINC000004097654 | 114.053 |
| 2543 | ZINC000000968157 | 105.351 | 5906 | ZINC000002003763 | 95.2908 |
| 2544 | ZINC000000968466 | 87.1023 | 5907 | ZINC000000001239 | 98.596 |
| 2545 | ZINC000033839262 | 85.5227 | 5908 | ZINC000003983907 | 107.742 |
| 2546 | ZINC000033839275 | 96.9964 | 5909 | ZINC000001577167 | 75.0551 |
| 2547 | ZINC000033951137 | 85.5248 | 5910 | ZINC000001577287 | 101.777 |
| 2548 | ZINC000085835668 | 105.243 | 5911 | ZINC000001591826 | 98.2916 |
| 2549 | ZINC000033839264 | 83.8526 | 5912 | ZINC000001594272 | 67.5282 |
| 2550 | ZINC000033839273 | 114.313 | 5913 | ZINC000001599098 | 53.7709 |
| 2551 | ZINC000033844408 | 75.6429 | 5914 | ZINC000001606159 | 70.5911 |
| 2552 | ZINC000033951149 | 82.1066 | 5915 | ZINC000001609512 | 80.5835 |
| 2553 | ZINC000033951475 | 82.1485 | 5916 | ZINC000001606158 | 70.3727 |
| 2554 | ZINC000033839276 | 111.156 | 5917 | ZINC000001609510 | 80.7775 |
| 2555 | ZINC000033861415 | 106.255 | 5918 | ZINC000001584050 | 109.391 |
| 2556 | ZINC000033951470 | 83.8744 | 5919 | ZINC000002384560 | 100.106 |
| 2557 | ZINC000034037251 | 96.2588 | 5920 | ZINC000005020016 | 90.0311 |
| 2558 | ZINC000033839274 | 89.5972 | 5921 | ZINC000002569742 | 98.8801 |
| 2559 | ZINC000085773084 | 101.339 | 5922 | ZINC000001531087 | 69.3651 |
| 2560 | ZINC000032163924 | 69.4377 | 5923 | ZINC000001627276 | 105.286 |
| 2561 | ZINC000032167026 | 94.4521 | 5924 | ZINC000000899053 | 98.0263 |
| 2562 | ZINC000033639584 | 95.1114 | 5925 | ZINC000000897516 | 70.8809 |
| 2563 | ZINC000032163928 | 59.9297 | 5926 | ZINC000001561970 | 124.955 |
| 2564 | ZINC000032169152 | 75.7908 | 5927 | ZINC000001602945 | 73.0876 |
| 2565 | ZINC000033506402 | 66.2085 | 5928 | ZINC000001680439 | 109.051 |
| 2566 | ZINC000032162511 | 83.6801 | 5929 | ZINC000001680661 | 103.296 |
| 2567 | ZINC000032163922 | 69.4354 | 5930 | ZINC000001680692 | 84.7414 |
| 2568 | ZINC000032175869 | 87.5181 | 5931 | ZINC000001680785 | 85.8321 |
| 2569 | ZINC000032190038 | 97.8373 | 5932 | ZINC000001680803 | 105.848 |
| 2570 | ZINC000033506403 | 67.3364 | 5933 | ZINC000001681318 | 62.0286 |
| 2571 | ZINC000085644707 | 96.8397 | 5934 | ZINC000013513777 | 111.574 |
| 2572 | ZINC000032163926 | 62.2033 | 5935 | ZINC000002566194 | 142.449 |
| 2573 | ZINC000032164300 | 82.429 | 5936 | ZINC000002567933 | 86.1505 |
| 2574 | ZINC000032176538 | 87.6472 | 5937 | ZINC000001715690 | 137.961 |
| 2575 | ZINC000032302828 | 92.6664 | 5938 | ZINC000001732431 | 98.6686 |
| 2576 | ZINC000033639585 | 96.6223 | 5939 | ZINC000000003252 | 104.368 |
| 2577 | ZINC000029786589 | 81.468 | 5940 | ZINC000005227006 | 81.5684 |
| 2578 | ZINC000029786605 | 72.3279 | 5941 | ZINC000005227717 | 56.2054 |
| 2579 | ZINC000031159895 | 135.177 | 5942 | ZINC000004228277 | 82.5672 |
| 2580 | ZINC000031356999 | 104.781 | 5943 | ZINC000005226618 | 102.651 |
| 2581 | ZINC000031362026 | 72.5182 | 5944 | ZINC000001680836 | 91.6165 |
| 2582 | ZINC000032153276 | 81.8737 | 5945 | ZINC000001699441 | 76.9282 |
| 2583 | ZINC000029786595 | 83.4039 | 5946 | ZINC000002034648 | 80.0232 |
| 2584 | ZINC000029786610 | 71.1526 | 5947 | ZINC000006071095 | 93.6996 |
| 2585 | ZINC000031555018 | 89.342 | 5948 | ZINC000004228245 | 133.695 |
| 2586 | ZINC000032006111 | 87.4821 | 5949 | ZINC000059200504 | 85.9926 |
| 2587 | ZINC000032142150 | 80.8427 | 5950 | ZINC000005224689 | 57.9658 |
| 2588 | ZINC000032142150 | 92.5773 | 5951 | ZINC000002077816 | 113.754 |
| 2589 | ZINC000032154769 | 68.8764 | 5952 | ZINC000005239363 | 82.61 |
| 2590 | ZINC000084843253 | 69.6185 | 5953 | ZINC000004228290 | 98.0937 |
| 2591 | ZINC000085491784 | 94.2588 | 5954 | ZINC000005227023 | 84.5251 |
| 2592 | ZINC000029786592 | 77.2322 | 5955 | ZINC000002566738 | 121.465 |
| 2593 | ZINC000029786608 | 74.7667 | 5956 | ZINC000001693632 | 93.2329 |
| 2594 | ZINC000031159899 | 139.279 | 5957 | ZINC000002561222 | 135.017 |
| 2595 | ZINC000031357002 | 118.746 | 5958 | ZINC000013513316 | 104.325 |
| 2596 | ZINC000032006109 | 89.7785 | 5959 | ZINC000031970695 | 91.0787 |
| 2597 | ZINC000032150481 | 77.4464 | 5960 | ZINC000001491943 | 131.707 |
| 2598 | ZINC000032152670 | 65.0039 | 5961 | ZINC000000001799 | 73.5948 |
| 2599 | ZINC000032153930 | 63.1975 | 5962 | ZINC000026515305 | 59.3658 |
| 2600 | ZINC000072221556 | 85.4186 | 5963 | ZINC000026515311 | 63.6685 |
| 2601 | ZINC000082946719 | 81.9897 | 5964 | ZINC000026515307 | 63.7213 |
| 2602 | ZINC000084397766 | 75.6907 | 5965 | ZINC000019701796 | 130.414 |
| 2603 | ZINC000029786597 | 77.5341 | 5966 | ZINC000001081322 | 87.6129 |
| 2604 | ZINC000029786613 | 71.478 | 5967 | ZINC000019014778 | 106.65 |
| 2605 | ZINC000032149496 | 78.656 | 5968 | ZINC000100003928 | 60.9572 |
| 2606 | ZINC000032153274 | 77.7015 | 5969 | ZINC000100004273 | 92.8361 |
| 2607 | ZINC000071775220 | 66.3906 | 5970 | ZINC000100006104 | 40.2104 |
| 2608 | ZINC000080052522 | 82.0694 | 5971 | ZINC000100019800 | 58.3084 |
| 2609 | ZINC000082946718 | 79.1601 | 5972 | ZINC000100042658 | 74.7057 |
| 2610 | ZINC000084397754 | 61.8117 | 5973 | ZINC000100021178 | 84.6191 |
| 2611 | ZINC000084403365 | 72.1275 | 5974 | ZINC000100021178 | 84.2221 |
| 2612 | ZINC000084403365 | 70.8853 | 5975 | ZINC000100030112 | 85.9842 |
| 2613 | ZINC000001596062 | 97.6146 | 5976 | ZINC000100014157 | 118.534 |
| 2614 | ZINC000001597767 | 46.8929 | 5977 | ZINC000100046328 | 69.3306 |
| 2615 | ZINC000070450851 | 88.8844 | 5978 | ZINC000100003619 | 84.769 |
| 2616 | ZINC000070451103 | 128.415 | 5979 | ZINC000100004275 | 87.5673 |
| 2617 | ZINC000070457351 | 58.7052 | 5980 | ZINC000100005530 | 89.3384 |
| 2618 | ZINC000070454420 | 119.697 | 5981 | ZINC000100014205 | 88.115 |
| 2619 | ZINC000055169533 | 89.7101 | 5982 | ZINC000100054128 | 71.0037 |
| 2620 | ZINC000059587222 | 89.1993 | 5983 | ZINC000008689949 | 107.093 |
| 2621 | ZINC000000057676 | 122.085 | 5984 | ZINC000008689957 | 119.022 |
| 2622 | ZINC000000057676 | 121.636 | 5985 | ZINC000100469657 | 96.4937 |
| 2623 | ZINC000001706910 | 71.7166 | 5986 | ZINC000100053615 | 139.458 |
| 2624 | ZINC000000407059 | 92.8221 | 5987 | ZINC000100070359 | 75.4198 |
| 2625 | ZINC000000002226 | 110.813 | 5988 | ZINC000100065712 | 97.0273 |
| 2626 | ZINC000000001288 | 92.2676 | 5989 | ZINC000100009149 | 64.8588 |
| 2627 | ZINC000000000491 | 99.7246 | 5990 | ZINC000000057751 | 122.206 |
| 2628 | ZINC000006093906 | 91.1859 | 5991 | ZINC000100014196 | 128.403 |
| 2629 | ZINC000000057677 | 121.26 | 5992 | ZINC000014438707 | 90.6598 |
| 2630 | ZINC000062234570 | 85.1232 | 5993 | ZINC000014438711 | 96.0632 |
| 2631 | ZINC000019132424 | 110.526 | 5994 | ZINC000014590585 | 92.4585 |
| 2632 | ZINC000003875336 | 120.51 | 5995 | ZINC000096068418 | 91.6664 |
| 2633 | ZINC000001665895 | 74.3622 | 5996 | ZINC000095618205 | 107.446 |
| 2634 | ZINC000001674080 | 76.7336 | 5997 | ZINC000014444683 | 84.6318 |
| 2635 | ZINC000001677800 | 97.821 | 5998 | ZINC000095953048 | 94.6273 |
| 2636 | ZINC000001680021 | 46.2848 | 5999 | ZINC000096014346 | 76.2297 |
| 2637 | ZINC000037866093 | 90.0592 | 6000 | ZINC000096014346 | 78.1928 |
| 2638 | ZINC000038141440 | 98.1871 | 6001 | ZINC000013540278 | 124.51 |
| 2639 | ZINC000038141452 | 101.549 | 6002 | ZINC000095617488 | 113.435 |
| 2640 | ZINC000038559595 | 112.379 | 6003 | ZINC000095617488 | 115.818 |
| 2641 | ZINC000038600322 | 112.796 | 6004 | ZINC000095620510 | 90.491 |
| 2642 | ZINC000038611873 | 94.2479 | 6005 | ZINC000014438709 | 93.6538 |
| 2643 | ZINC000038612043 | 98.0816 | 6006 | ZINC000096300864 | 60.4278 |
| 2644 | ZINC000038858000 | 99.0408 | 6007 | ZINC000096901137 | 85.0779 |
| 2645 | ZINC000038886594 | 69.3728 | 6008 | ZINC000002048317 | 107.847 |
| 2646 | ZINC000039260233 | 65.1183 | 6009 | ZINC000001635640 | 128.406 |
| 2647 | ZINC000038143543 | 120.579 | 6010 | ZINC000014588869 | 83.8246 |
| 2648 | ZINC000038143713 | 114.949 | 6011 | ZINC000013512210 | 76.1693 |
| 2649 | ZINC000038143761 | 125.039 | 6012 | ZINC000014587709 | 92.3055 |
| 2650 | ZINC000038143805 | 133.836 | 6013 | ZINC000014448169 | 94.6702 |
| 2651 | ZINC000038609808 | 95.5616 | 6014 | ZINC000000154980 | 105.077 |
| 2652 | ZINC000038611099 | 105.935 | 6015 | ZINC000000156517 | 81.5114 |
| 2653 | ZINC000038611874 | 95.5016 | 6016 | ZINC000002047226 | 72.7235 |
| 2654 | ZINC000038611958 | 68.7885 | 6017 | ZINC000004098749 | 118.153 |
| 2655 | ZINC000039260232 | 64.1623 | 6018 | ZINC000014655907 | 138.431 |
| 2656 | ZINC000001669521 | 112.828 | 6019 | ZINC000014589063 | 108.346 |
| 2657 | ZINC000001677794 | 101.056 | 6020 | ZINC000014589124 | 66.8882 |
| 2658 | ZINC000001653216 | 81.1645 | 6021 | ZINC000014590680 | 75.4719 |
| 2659 | ZINC000003861284 | 67.0194 | 6022 | ZINC000002040376 | 100.477 |
| 2660 | ZINC000003861661 | 119.813 | 6023 | ZINC000014438713 | 93.1604 |
| 2661 | ZINC000033975163 | 72.1786 | 6024 | ZINC000000056474 | 151.804 |
| 2662 | ZINC000033975162 | 71.5709 | 6025 | ZINC000006031150 | 67.1434 |
| 2663 | ZINC000000895208 | 88.3259 | 6026 | ZINC000006030659 | 63.6416 |
| 2664 | ZINC000033754089 | 95.7195 | 6027 | ZINC000006018691 | 111.48 |
| 2665 | ZINC000058574669 | 143.412 | 6028 | ZINC000004100761 | 106.996 |
| 2666 | ZINC000000057670 | 132.969 | 6029 | ZINC000005650763 | 44.3848 |
| 2667 | ZINC000013108265 | 115.722 | 6030 | ZINC000005650642 | 144.967 |
| 2668 | ZINC000000057674 | 115.83 | 6031 | ZINC000006037891 | 60.9031 |
| 2669 | ZINC000001636704 | 89.1276 | 6032 | ZINC000000057125 | 130.706 |
| 2670 | ZINC000018168715 | 84.4683 | 6033 | ZINC000000187911 | 128.316 |
| 2671 | ZINC000000305336 | 98.633 | 6034 | ZINC000001081243 | 100.91 |
| 2672 | ZINC000001693738 | 94.572 | 6035 | ZINC000001545034 | 131.375 |
| 2673 | ZINC000001699890 | 64.6118 | 6036 | ZINC000004081428 | 124.832 |
| 2674 | ZINC000001686990 | 81.2925 | 6037 | ZINC000004216870 | 130.369 |
| 2675 | ZINC000001598717 | 69.4578 | 6038 | ZINC000000004749 | 144.357 |
| 2676 | ZINC000004098600 | 140.331 | 6039 | ZINC000012358883 | 120.231 |
| 2677 | ZINC000004262103 | 86.2573 | 6040 | ZINC000012358720 | 98.1448 |
| 2678 | ZINC000003871358 | 134.998 | 6041 | ZINC000003834164 | 117.722 |
| 2679 | ZINC000003871358 | 135.6 | 6042 | ZINC000000158743 | 95.5427 |
| 2680 | ZINC000003197732 | 98.4129 | 6043 | ZINC000000162515 | 87.1263 |
| 2681 | ZINC000060194474 | 78.3932 | 6044 | ZINC000002379217 | 94.9471 |
| 2682 | ZINC000001531089 | 99.8518 | 6045 | ZINC000038192546 | 62.6942 |
| 2683 | ZINC000059587245 | 75.3338 | 6046 | ZINC000005133396 | 61.6614 |
| 2684 | ZINC000059588874 | 80.1205 | 6047 | ZINC000005841334 | 74.7247 |
| 2685 | ZINC000006092865 | 143.102 | 6048 | ZINC000005842678 | 90.3596 |
| 2686 | ZINC000006092269 | 112.876 | 6049 | ZINC000005842977 | 110.563 |
| 2687 | ZINC000006403375 | 94.4372 | 6050 | ZINC000005841087 | 70.6614 |
| 2688 | ZINC000006403375 | 97.4542 | 6051 | ZINC000005841345 | 78.1976 |
| 2689 | ZINC000000388551 | 97.771 | 6052 | ZINC000000157118 | 104.3 |
| 2690 | ZINC000000389532 | 93.3069 | 6053 | ZINC000000157134 | 87.2952 |
| 2691 | ZINC000000392003 | 72.2372 | 6054 | ZINC000000402623 | 96.4831 |
| 2692 | ZINC000000153731 | 102.001 | 6055 | ZINC000001559620 | 143.357 |
| 2693 | ZINC000000105076 | 146.723 | 6056 | ZINC000033903696 | 100.797 |
| 2694 | ZINC000000388507 | 68.2578 | 6057 | ZINC000000391826 | 74.124 |
| 2695 | ZINC000000001021 | 114.856 | 6058 | ZINC000000155807 | 143.938 |
| 2696 | ZINC000012660678 | 93.7564 | 6059 | ZINC000005819999 | 104.96 |
| 2697 | ZINC000000900182 | 95.3982 | 6060 | ZINC000000388757 | 92.5464 |
| 2698 | ZINC000013378574 | 118.017 | 6061 | ZINC000000057920 | 122.868 |
| 2699 | ZINC000001582158 | 138.09 | 6062 | ZINC000000002209 | 105.613 |
| 2700 | ZINC000000899102 | 132.538 | 6063 | ZINC000002031663 | 83.5144 |
| 2701 | ZINC000013377934 | 145.405 | 6064 | ZINC000002034630 | 70.5449 |
| 2702 | ZINC000001724917 | 122.154 | 6065 | ZINC000002034880 | 69.6984 |
| 2703 | ZINC000019340795 | 132.888 | 6066 | ZINC000000057908 | 94.0348 |
| 2704 | ZINC000000013245 | 83.7343 | 6067 | ZINC000004887087 | 118.821 |
| 2705 | ZINC000000056654 | 84.9223 | 6068 | ZINC000002038252 | 112.71 |
| 2706 | ZINC000100070981 | 116.774 | 6069 | ZINC000002036968 | 103.145 |
| 2707 | ZINC000004252553 | 125.9 | 6070 | ZINC000002037372 | 102.053 |
| 2708 | ZINC000000001083 | 132.815 | 6071 | ZINC000002038253 | 100.818 |
| 2709 | ZINC000001703459 | 121.731 | 6072 | ZINC000014860465 | 99.0986 |
| 2710 | ZINC000001699444 | 89.5266 | 6073 | ZINC000005735437 | 130.071 |
| 2711 | ZINC000000155259 | 69.7622 | 6074 | ZINC000005735752 | 80.4853 |
| 2712 | ZINC000005137905 | 114.773 | 6075 | ZINC000005735822 | 79.527 |
| 2713 | ZINC000100392377 | 97.329 | 6076 | ZINC000014947781 | 147.28 |
| 2714 | ZINC000005157983 | 113.934 | 6077 | ZINC000005732869 | 95.718 |
| 2715 | ZINC000004098354 | 120.228 | 6078 | ZINC000001577074 | 86.4698 |
| 2716 | ZINC000019701798 | 104.743 | 6079 | ZINC000005457154 | 78.1007 |
| 2717 | ZINC000000265747 | 105.497 | 6080 | ZINC000003999316 | 94.9918 |
| 2718 | ZINC000000057165 | 82.8147 | 6081 | ZINC000000967571 | 71.5523 |
| 2719 | ZINC000000391840 | 135.257 | 6082 | ZINC000000409206 | 90.8724 |
| 2720 | ZINC000000391840 | 127.51 | 6083 | ZINC000005600727 | 75.7095 |
| 2721 | ZINC000000901251 | 76.4556 | 6084 | ZINC000003875620 | 111.8 |
| 2722 | ZINC000000338330 | 108.644 | 6085 | ZINC000003875620 | 110.698 |
| 2723 | ZINC000000406991 | 93.0132 | 6086 | ZINC000003875620 | 111.8 |
| 2724 | ZINC000000225578 | 112.606 | 6087 | ZINC000056871335 | 104.241 |
| 2725 | ZINC000000225579 | 114.716 | 6088 | ZINC000056871336 | 109.351 |
| 2726 | ZINC000000056510 | 100.776 | 6089 | ZINC000056874634 | 69.2716 |
| 2727 | ZINC000000518798 | 102.055 | 6090 | ZINC000005179608 | 87.3956 |
| 2728 | ZINC000000895645 | 128.554 | 6091 | ZINC000004082318 | 88.665 |
| 2729 | ZINC000000897612 | 136 | 6092 | ZINC000004087886 | 76.1981 |
| 2730 | ZINC000000898299 | 111.848 | 6093 | ZINC000056874637 | 72.1595 |
| 2731 | ZINC000000898605 | 106.561 | 6094 | ZINC000000968471 | 109.856 |
| 2732 | ZINC000000899362 | 134.829 | 6095 | ZINC000001845930 | 106.763 |
| 2733 | ZINC000000899620 | 106.2 | 6096 | ZINC000100028524 | 110.591 |
| 2734 | ZINC000013328231 | 118.442 | 6097 | ZINC000000895807 | 97.4744 |
| 2735 | ZINC000013377554 | 124.272 | 6098 | ZINC000000901783 | 89.4729 |
| 2736 | ZINC000013378188 | 145.408 | 6099 | ZINC000000967598 | 79.7881 |
| 2737 | ZINC000014558314 | 131.994 | 6100 | ZINC000000968236 | 82.6941 |
| 2738 | ZINC000013509247 | 107.468 | 6101 | ZINC000000333860 | 68.6603 |
| 2739 | ZINC000013532023 | 107.894 | 6102 | ZINC000000393725 | 110.365 |
| 2740 | ZINC000013889040 | 132.938 | 6103 | ZINC000000401997 | 79.2638 |
| 2741 | ZINC000000039119 | 89.8833 | 6104 | ZINC000000895805 | 93.8674 |
| 2742 | ZINC000014761282 | 118.811 | 6105 | ZINC000000896001 | 93.2547 |
| 2743 | ZINC000014776066 | 88.9099 | 6106 | ZINC000000897525 | 87.2023 |
| 2744 | ZINC000014814114 | 98.4653 | 6107 | ZINC000000899546 | 84.5468 |
| 2745 | ZINC000014618924 | 102.699 | 6108 | ZINC000000967583 | 81.9069 |
| 2746 | ZINC000014780940 | 104.918 | 6109 | ZINC000000968078 | 140.694 |
| 2747 | ZINC000012153222 | 88.0347 | 6110 | ZINC000000400150 | 84.8353 |
| 2748 | ZINC000012153561 | 117.628 | 6111 | ZINC000000410088 | 87.3575 |
| 2749 | ZINC000013481874 | 155.842 | 6112 | ZINC000002164100 | 94.1916 |
| 2750 | ZINC000013532008 | 105.639 | 6113 | ZINC000000410085 | 90.797 |
| 2751 | ZINC000014439508 | 124.962 | 6114 | ZINC000002035304 | 102.535 |
| 2752 | ZINC000014516509 | 90.237 | 6115 | ZINC000004582587 | 101.632 |
| 2753 | ZINC000014455391 | 114.299 | 6116 | ZINC000005734944 | 88.1706 |
| 2754 | ZINC000014504538 | 102.026 | 6117 | ZINC000013838497 | 127.85 |
| 2755 | ZINC000013307925 | 153.427 | 6118 | ZINC000095618214 | 78.1484 |
| 2756 | ZINC000013340660 | 129.943 | 6119 | ZINC000000410087 | 83.4647 |
| 2757 | ZINC000013375725 | 130.338 | 6120 | ZINC000001633170 | 109.317 |
| 2758 | ZINC000013380841 | 102.22 | 6121 | ZINC000002164098 | 95.38 |
| 2759 | ZINC000013412695 | 71.2107 | 6122 | ZINC000004582605 | 80.2614 |
| 2760 | ZINC000000080786 | 95.1379 | 6123 | ZINC000015121960 | 130.1 |
| 2761 | ZINC000013302375 | 126.146 | 6124 | ZINC000095618215 | 79.2196 |
| 2762 | ZINC000020470300 | 136.968 | 6125 | ZINC000000899553 | 126.219 |
| 2763 | ZINC000006482465 | 148.981 | 6126 | ZINC000000899126 | 124.118 |
| 2764 | ZINC000006484558 | 136.358 | 6127 | ZINC000000568062 | 116.865 |
| 2765 | ZINC000006484558 | 135.689 | 6128 | ZINC000000898296 | 97.751 |
| 2766 | ZINC000016954710 | 106.037 | 6129 | ZINC000004027246 | 94.0718 |
| 2767 | ZINC000016943064 | 90.4164 | 6130 | ZINC000000967601 | 72.8793 |
| 2768 | ZINC000016051536 | 112.369 | 6131 | ZINC000000248378 | 103.353 |
| 2769 | ZINC000015115129 | 146.771 | 6132 | ZINC000013452216 | 113.417 |
| 2770 | ZINC000015119500 | 125.996 | 6133 | ZINC000013452213 | 120.993 |
| 2771 | ZINC000015207329 | 102.171 | 6134 | ZINC000015274358 | 104.787 |
| 2772 | ZINC000005934539 | 135.812 | 6135 | ZINC000085808820 | 125.735 |
| 2773 | ZINC000005037516 | 104.256 | 6136 | ZINC000085659636 | 108.753 |
| 2774 | ZINC000005998557 | 135.484 | 6137 | ZINC000001529208 | 90.6445 |
| 2775 | ZINC000006070307 | 96.748 | 6138 | ZINC000084403374 | 91.154 |
| 2776 | ZINC000003977785 | 91.6924 | 6139 | ZINC000085508720 | 115.227 |
| 2777 | ZINC000004261920 | 88.5439 | 6140 | ZINC000086052053 | 117.414 |
| 2778 | ZINC000013374351 | 136.991 | 6141 | ZINC000085936592 | 111.199 |
| 2779 | ZINC000013374352 | 149.929 | 6142 | ZINC000086022659 | 108.447 |
| 2780 | ZINC000015115178 | 144.383 | 6143 | ZINC000085808811 | 129.373 |
| 2781 | ZINC000001850847 | 79.8843 | 6144 | ZINC000085659450 | 105.869 |
| 2782 | ZINC000003964188 | 122.469 | 6145 | ZINC000086022558 | 111.083 |
| 2783 | ZINC000003964188 | 117.91 | 6146 | ZINC000085659453 | 111.517 |
| 2784 | ZINC000002516185 | 104.685 | 6147 | ZINC000084397744 | 61.2262 |
| 2785 | ZINC000028537635 | 100.237 | 6148 | ZINC000085808802 | 128.756 |
| 2786 | ZINC000028702248 | 104.741 | 6149 | ZINC000086022662 | 106.944 |
| 2787 | ZINC000004098120 | 83.4766 | 6150 | ZINC000086022556 | 110.767 |
| 2788 | ZINC000004098322 | 114.666 | 6151 | ZINC000085808798 | 113.653 |
| 2789 | ZINC000004098362 | 101.815 | 6152 | ZINC000043523728 | 107.56 |
| 2790 | ZINC000003871987 | 61.7448 | 6153 | ZINC000042804200 | 55.4509 |
| 2791 | ZINC000003947422 | 130.612 | 6154 | ZINC000000058120 | 134.285 |
| 2792 | ZINC000004073913 | 95.8431 | 6155 | ZINC000004098425 | 105.165 |
| 2793 | ZINC000003978626 | 97.0802 | 6156 | ZINC000000409331 | 84.7528 |
| 2794 | ZINC000004102397 | 124.911 | 6157 | ZINC000001586738 | 55.9229 |
| 2795 | ZINC000004098152 | 101.21 | 6158 | ZINC000015042997 | 73.5849 |
| 2796 | ZINC000004098195 | 114.397 | 6159 | ZINC000000897493 | 37.3869 |
| 2797 | ZINC000004098657 | 130.15 | 6160 | ZINC000001586317 | 85.5668 |
| 2798 | ZINC000003874657 | 77.7406 | 6161 | ZINC000000155696 | 121.855 |
| 2799 | ZINC000003874658 | 80.364 | 6162 | ZINC000001087344 | 85.9811 |
| 2800 | ZINC000004098663 | 120.191 | 6163 | ZINC000000164581 | 88.3382 |
| 2801 | ZINC000004252572 | 137.95 | 6164 | ZINC000000166877 | 81.7005 |
| 2802 | ZINC000004533847 | 116.48 | 6165 | ZINC000000334875 | 74.8568 |
| 2803 | ZINC000002554931 | 119.229 | 6166 | ZINC000002013560 | 76.8613 |
| 2804 | ZINC000005125148 | 90.3897 | 6167 | ZINC000002011663 | 79.5036 |
| 2805 | ZINC000004804529 | 124.71 | 6168 | ZINC000001531085 | 90.1815 |
| 2806 | ZINC000004831363 | 78.6728 | 6169 | ZINC000001558184 | 119.46 |
| 2807 | ZINC000006654972 | 87.0434 | 6170 | ZINC000001567243 | 106.275 |
| 2808 | ZINC000006037446 | 101.581 | 6171 | ZINC000008036015 | 115.925 |
| 2809 | ZINC000006030951 | 80.2791 | 6172 | ZINC000000409269 | 107.033 |
| 2810 | ZINC000006067341 | 105.664 | 6173 | ZINC000001602516 | 65.8722 |
| 2811 | ZINC000006030836 | 82.0386 | 6174 | ZINC000001586745 | 71.1144 |
| 2812 | ZINC000006018466 | 99.5564 | 6175 | ZINC000003881372 | 135.473 |
| 2813 | ZINC000005247757 | 102.153 | 6176 | ZINC000003874807 | 137.272 |
| 2814 | ZINC000005854640 | 107.433 | 6177 | ZINC000002004049 | 47.3513 |
| 2815 | ZINC000006006751 | 137.814 | 6178 | ZINC000000001912 | 123.377 |
| 2816 | ZINC000015058362 | 105.07 | 6179 | ZINC000015272539 | 95.6295 |
| 2817 | ZINC000014922237 | 91.0804 | 6180 | ZINC000000897751 | 67.691 |
| 2818 | ZINC000014658321 | 122.926 | 6181 | ZINC000059587341 | 99.2966 |
| 2819 | ZINC000014692588 | 132.49 | 6182 | ZINC000059588901 | 92.4224 |
| 2820 | ZINC000003638105 | 79.4597 | 6183 | ZINC000059727289 | 106.328 |
| 2821 | ZINC000005934541 | 122.959 | 6184 | ZINC000059778565 | 79.4258 |
| 2822 | ZINC000033400147 | 125.583 | 6185 | ZINC000059778576 | 87.0234 |
| 2823 | ZINC000000152926 | 82.8309 | 6186 | ZINC000136185123 | 110.796 |
| 2824 | ZINC000000156899 | 85.0668 | 6187 | ZINC000070454250 | 83.9884 |
| 2825 | ZINC000019796061 | 101.36 | 6188 | ZINC000078275009 | 95.3187 |
| 2826 | ZINC000003860577 | 80.3269 | 6189 | ZINC000079566582 | 99.6179 |
| 2827 | ZINC000003881649 | 98.7358 | 6190 | ZINC000079566589 | 98.2099 |
| 2828 | ZINC000005212248 | 111.282 | 6191 | ZINC000083812292 | 67.7373 |
| 2829 | ZINC000000001906 | 96.4137 | 6192 | ZINC000230081381 | 85.8834 |
| 2830 | ZINC000003875850 | 103.3 | 6193 | ZINC000085598972 | 92.5647 |
| 2831 | ZINC000000033299 | 118.782 | 6194 | ZINC000085599280 | 99.1526 |
| 2832 | ZINC000029056300 | 140.339 | 6195 | ZINC000085644719 | 85.3197 |
| 2833 | ZINC000005202250 | 92.8341 | 6196 | ZINC000230085876 | 87.0409 |
| 2834 | ZINC000005732268 | 101.587 | 6197 | ZINC000085809561 | 108.447 |
| 2835 | ZINC000005461939 | 117.8 | 6198 | ZINC000085947793 | 76.9888 |
| 2836 | ZINC000006018481 | 143.183 | 6199 | ZINC000085972319 | 97.7187 |
| 2837 | ZINC000006018481 | 138.13 | 6200 | ZINC000085972327 | 94.3717 |
| 2838 | ZINC000006018563 | 130.814 | 6201 | ZINC000085972335 | 97.4104 |
| 2839 | ZINC000003845299 | 104.336 | 6202 | ZINC000085972342 | 95.0384 |
| 2840 | ZINC000000898477 | 95.887 | 6203 | ZINC000085972365 | 92.3397 |
| 2841 | ZINC000000586482 | 92.3421 | 6204 | ZINC000085972373 | 94.9195 |
| 2842 | ZINC000000517281 | 96.5802 | 6205 | ZINC000086050410 | 96.9966 |
| 2843 | ZINC000004098306 | 136.285 | 6206 | ZINC000086052266 | 105.018 |
| 2844 | ZINC000000404370 | 90.9954 | 6207 | ZINC000087496269 | 103.704 |
| 2845 | ZINC000003860432 | 109.694 | 6208 | ZINC000001532203 | 48.6316 |
| 2846 | ZINC000084619434 | 114.899 | 6209 | ZINC000001532246 | 92.0971 |
| 2847 | ZINC000084634324 | 94.0859 | 6210 | ZINC000001532575 | 79.1367 |
| 2848 | ZINC000084619432 | 97.4508 | 6211 | ZINC000095618075 | 82.3179 |
| 2849 | ZINC000000086470 | 118.559 | 6212 | ZINC000095618207 | 73.5865 |
| 2850 | ZINC000018190318 | 128.201 | 6213 | ZINC000095618232 | 85.8057 |
| 2851 | ZINC000000035526 | 114.346 | 6214 | ZINC000095618366 | 57.3172 |
| 2852 | ZINC000000058121 | 87.8383 | 6215 | ZINC000095914189 | 85.5668 |
| 2853 | ZINC000001587606 | 89.0452 | 6216 | ZINC000098045375 | 93.4272 |
| 2854 | ZINC000006018480 | 140.154 | 6217 | ZINC000095618206 | 74.6831 |
| 2855 | ZINC000053312686 | 95.3017 | 6218 | ZINC000095618228 | 91.997 |
| 2856 | ZINC000000001536 | 134.748 | 6219 | ZINC000095618238 | 38.3833 |
| 2857 | ZINC000049033512 | 71.047 | 6220 | ZINC000002583634 | 122.536 |
| 2858 | ZINC000005962605 | 124.913 | 6221 | ZINC000002600023 | 104.873 |
| 2859 | ZINC000014593164 | 77.3149 | 6222 | ZINC000059066461 | 87.8382 |
| 2860 | ZINC000000094256 | 123.367 | 6223 | ZINC000059193058 | 88.2601 |
| 2861 | ZINC000000898785 | 135.405 | 6224 | ZINC000059217448 | 69.5287 |
| 2862 | ZINC000006116596 | 122.63 | 6225 | ZINC000059727292 | 105.71 |
| 2863 | ZINC000000164488 | 73.4217 | 6226 | ZINC000059778561 | 86.9432 |
| 2864 | ZINC000000199415 | 98.7394 | 6227 | ZINC000059778570 | 91.4045 |
| 2865 | ZINC000000164545 | 74.7138 | 6228 | ZINC000059778948 | 90.9886 |
| 2866 | ZINC000000238671 | 122.582 | 6229 | ZINC000059994377 | 69.7063 |
| 2867 | ZINC000002020114 | 122.796 | 6230 | ZINC000070454368 | 90.1459 |
| 2868 | ZINC000012153709 | 97.0058 | 6231 | ZINC000070455184 | 90.2279 |
| 2869 | ZINC000006661321 | 73.2465 | 6232 | ZINC000078138492 | 79.6336 |
| 2870 | ZINC000012153803 | 64.7638 | 6233 | ZINC000078275012 | 94.6638 |
| 2871 | ZINC000004758250 | 124.931 | 6234 | ZINC000079566585 | 98.3435 |
| 2872 | ZINC000000236891 | 112.904 | 6235 | ZINC000079566593 | 93.3976 |
| 2873 | ZINC000000156245 | 80.5959 | 6236 | ZINC000083409141 | 103.255 |
| 2874 | ZINC000001085207 | 71.1952 | 6237 | ZINC000083409146 | 104.59 |
| 2875 | ZINC000000394712 | 77.8944 | 6238 | ZINC000083812291 | 68.8138 |
| 2876 | ZINC000000396075 | 69.2396 | 6239 | ZINC000085566876 | 97.2272 |
| 2877 | ZINC000000394775 | 67.0445 | 6240 | ZINC000085599008 | 95.8694 |
| 2878 | ZINC000000400176 | 80.3719 | 6241 | ZINC000085600726 | 87.1339 |
| 2879 | ZINC000001095292 | 98.8444 | 6242 | ZINC000230085886 | 86.1301 |
| 2880 | ZINC000000389599 | 98.6688 | 6243 | ZINC000085809558 | 106.919 |
| 2881 | ZINC000000395594 | 82.6814 | 6244 | ZINC000085897787 | 104.405 |
| 2882 | ZINC000001529214 | 78.5941 | 6245 | ZINC000085947795 | 78.3684 |
| 2883 | ZINC000001530705 | 101.187 | 6246 | ZINC000085972323 | 95.0073 |
| 2884 | ZINC000001997121 | 97.2105 | 6247 | ZINC000085972331 | 96.4404 |
| 2885 | ZINC000000388744 | 119.141 | 6248 | ZINC000085972338 | 98.0443 |
| 2886 | ZINC000002097863 | 140.976 | 6249 | ZINC000085972363 | 95.0812 |
| 2887 | ZINC000029340877 | 117.086 | 6250 | ZINC000085972368 | 92.364 |
| 2888 | ZINC000013377889 | 114.201 | 6251 | ZINC000086002189 | 102.089 |
| 2889 | ZINC000000038933 | 124.271 | 6252 | ZINC000086052267 | 106.685 |
| 2890 | ZINC000004098142 | 104.404 | 6253 | ZINC000090372801 | 101.409 |
| 2891 | ZINC000004098173 | 101.256 | 6254 | ZINC000001850444 | 86.5615 |
| 2892 | ZINC000004098229 | 104.579 | 6255 | ZINC000001850505 | 79.3299 |
| 2893 | ZINC000004097989 | 104.664 | 6256 | ZINC000001850550 | 69.5776 |
| 2894 | ZINC000004097949 | 100.447 | 6257 | ZINC000000345844 | 99.6584 |
| 2895 | ZINC000002031811 | 117.803 | 6258 | ZINC000000388057 | 75.8605 |
| 2896 | ZINC000013408812 | 85.4655 | 6259 | ZINC000000388078 | 60.8245 |
| 2897 | ZINC000005733537 | 120.977 | 6260 | ZINC000001699440 | 77.9291 |
| 2898 | ZINC000005733537 | 120.977 | 6261 | ZINC000001699944 | 51.9872 |
| 2899 | ZINC000004097684 | 101.734 | 6262 | ZINC000001707854 | 99.8675 |
| 2900 | ZINC000015054024 | 101.139 | 6263 | ZINC000001687154 | 58.8543 |
| 2901 | ZINC000014758407 | 134.162 | 6264 | ZINC000002561202 | 82.3525 |
| 2902 | ZINC000016343443 | 120.762 | 6265 | ZINC000003814411 | 123.606 |
| 2903 | ZINC000014684940 | 113.013 | 6266 | ZINC000004975178 | 68.1292 |
| 2904 | ZINC000014971343 | 109.497 | 6267 | ZINC000004983274 | 94.565 |
| 2905 | ZINC000006719013 | 112.943 | 6268 | ZINC000000358476 | 75.3992 |
| 2906 | ZINC000006575643 | 108.287 | 6269 | ZINC000001850460 | 68.6329 |
| 2907 | ZINC000006403380 | 117.721 | 6270 | ZINC000001850552 | 79.0499 |
| 2908 | ZINC000000001412 | 135.32 | 6271 | ZINC000001850690 | 67.336 |
| 2909 | ZINC000012358996 | 88.1236 | 6272 | ZINC000000402871 | 101.351 |
| 2910 | ZINC000100868074 | 115.645 | 6273 | ZINC000000388082 | 75.2875 |
| 2911 | ZINC000100868074 | 109.249 | 6274 | ZINC000001850675 | 100.661 |
| 2912 | ZINC000100868074 | 114.391 | 6275 | ZINC000001712309 | 65.8128 |
| 2913 | ZINC000005822255 | 99.5403 | 6276 | ZINC000001867144 | 60.3807 |
| 2914 | ZINC000018136415 | 129.795 | 6277 | ZINC000000968254 | 69.4849 |
| 2915 | ZINC000018136415 | 128.062 | 6278 | ZINC000000985403 | 129.273 |
| 2916 | ZINC000004175523 | 122.514 | 6279 | ZINC000000967332 | 74.4143 |
| 2917 | ZINC000000154661 | 88.7508 | 6280 | ZINC000001851030 | 110.521 |
| 2918 | ZINC000005998596 | 95.7617 | 6281 | ZINC000000388167 | 78.6985 |
| 2919 | ZINC000012446789 | 113.992 | 6282 | ZINC000002384556 | 80.4224 |
| 2920 | ZINC000008579298 | 89.0656 | 6283 | ZINC000002566224 | 70.5498 |
| 2921 | ZINC000003581377 | 77.6398 | 6284 | ZINC000001734242 | 92.0581 |
| 2922 | ZINC000004529319 | 81.137 | 6285 | ZINC000000007267 | 81.1628 |
| 2923 | ZINC000000157156 | 71.8374 | 6286 | ZINC000000009073 | 120.463 |
| 2924 | ZINC000001678791 | 84.0927 | 6287 | ZINC000000388086 | 73.9057 |
| 2925 | ZINC000015987659 | 101.833 | 6288 | ZINC000000391812 | 92.7538 |
| 2926 | ZINC000000026301 | 124.264 | 6289 | ZINC000000075008 | 97.6501 |
| 2927 | ZINC000004321512 | 114.049 | 6290 | ZINC000000000353 | 102.605 |
| 2928 | ZINC000012360009 | 154.338 | 6291 | ZINC000000120179 | 99.2045 |
| 2929 | ZINC000000388241 | 71.8973 | 6292 | ZINC000028642532 | 123.647 |
| 2930 | ZINC000000388289 | 72.9106 | 6293 | ZINC000002558125 | 105.181 |
| 2931 | ZINC000000526257 | 84.218 | 6294 | ZINC000000239015 | 90.9056 |
| 2932 | ZINC000000157427 | 75.8864 | 6295 | ZINC000003873123 | 100.287 |
| 2933 | ZINC000004097876 | 127.52 | 6296 | ZINC000014806381 | 128.373 |
| 2934 | ZINC000004097107 | 125.673 | 6297 | ZINC000008214634 | 123.342 |
| 2935 | ZINC000002516866 | 102.118 | 6298 | ZINC000008585883 | 40.12 |
| 2936 | ZINC000002583633 | 120.946 | 6299 | ZINC000002077814 | 97.1754 |
| 2937 | ZINC000003818826 | 105.509 | 6300 | ZINC000059585871 | 112.147 |
| 2938 | ZINC000000156709 | 78.9078 | 6301 | ZINC000001235986 | 78.7109 |
| 2939 | ZINC000000157146 | 81.7742 | 6302 | ZINC000004082214 | 115.986 |
| 2940 | ZINC000001586749 | 67.7178 | 6303 | ZINC000018286095 | 106.341 |
| 2941 | ZINC000001586770 | 93.9198 | 6304 | ZINC000000155806 | 129.74 |
| 2942 | ZINC000001677783 | 97.3745 | 6305 | ZINC000000164617 | 89.4657 |
| 2943 | ZINC000001676373 | 81.3723 | 6306 | ZINC000001573356 | 100.964 |
| 2944 | ZINC000001589669 | 74.3707 | 6307 | ZINC000012405046 | 81.8447 |
| 2945 | ZINC000001674077 | 58.655 | 6308 | ZINC000012428336 | 126.832 |
| 2946 | ZINC000003861630 | 106.496 | 6309 | ZINC000012416741 | 108.528 |
| 2947 | ZINC000003860202 | 77.3132 | 6310 | ZINC000000391207 | 65.8122 |
| 2948 | ZINC000003847413 | 89.5283 | 6311 | ZINC000012496101 | 97.1991 |
| 2949 | ZINC000001850952 | 87.4918 | 6312 | ZINC000012496640 | 111.58 |
| 2950 | ZINC000001851033 | 84.2095 | 6313 | ZINC000012494057 | 124.106 |
| 2951 | ZINC000001591604 | 57.2012 | 6314 | ZINC000000338411 | 106.723 |
| 2952 | ZINC000001597766 | 46.9614 | 6315 | ZINC000002146973 | 141.731 |
| 2953 | ZINC000000899402 | 73.6599 | 6316 | ZINC000000538386 | 43.5129 |
| 2954 | ZINC000003860326 | 85.6139 | 6317 | ZINC000012341538 | 70.7399 |
| 2955 | ZINC000003860476 | 66.4372 | 6318 | ZINC000001644308 | 113.674 |
| 2956 | ZINC000003860605 | 62.0931 | 6319 | ZINC000001874346 | 104.806 |
| 2957 | ZINC000001850920 | 80.1944 | 6320 | ZINC000001874348 | 108.078 |
| 2958 | ZINC000001850883 | 96.6569 | 6321 | ZINC000001874350 | 107.547 |
| 2959 | ZINC000001850963 | 85.8448 | 6322 | ZINC000001874351 | 107.059 |
| 2960 | ZINC000001851037 | 89.0426 | 6323 | ZINC000002583632 | 114.283 |
| 2961 | ZINC000000157490 | 97.0351 | 6324 | ZINC000003831614 | 85.5749 |
| 2962 | ZINC000000157525 | 89.0935 | 6325 | ZINC000000000857 | 140.947 |
| 2963 | ZINC000000157768 | 78.7264 | 6326 | ZINC000000043475 | 99.0616 |
| 2964 | ZINC000003875521 | 92.9824 | 6327 | ZINC000000391883 | 70.6886 |
| 2965 | ZINC000003875729 | 85.1076 | 6328 | ZINC000000896041 | 140.129 |
| 2966 | ZINC000003875772 | 113.393 | 6329 | ZINC000001529772 | 125.171 |
| 2967 | ZINC000001672944 | 76.8672 | 6330 | ZINC000001532735 | 78.8407 |
| 2968 | ZINC000001867146 | 60.6623 | 6331 | ZINC000001547195 | 102.05 |
| 2969 | ZINC000003860657 | 84.6175 | 6332 | ZINC000001850826 | 98.3189 |
| 2970 | ZINC000000402666 | 112.598 | 6333 | ZINC000003833954 | 104.69 |
| 2971 | ZINC000003881360 | 124.581 | 6334 | ZINC000003860906 | 127.464 |
| 2972 | ZINC000000968029 | 77.9871 | 6335 | ZINC000003861537 | 81.8613 |
| 2973 | ZINC000000967335 | 96.6206 | 6336 | ZINC000004262096 | 90.2307 |
| 2974 | ZINC000001655743 | 105.6 | 6337 | ZINC000004658608 | 38.8009 |
| 2975 | ZINC000000901286 | 57.7702 | 6338 | ZINC000004658609 | 37.6645 |
| 2976 | ZINC000000901619 | 52.7461 | 6339 | ZINC000008584338 | 119.413 |
| 2977 | ZINC000003881614 | 113.02 | 6340 | ZINC000100016113 | 97.8471 |
| 2978 | ZINC000000967523 | 82.4455 | 6341 | ZINC000001841306 | 104.121 |
| 2979 | ZINC000000901301 | 54.1157 | 6342 | ZINC000001611085 | 106.266 |
| 2980 | ZINC000000899915 | 129.273 | 6343 | ZINC000100776283 | 106.987 |
| 2981 | ZINC000000900119 | 93.2958 | 6344 | ZINC000100776288 | 110.056 |
| 2982 | ZINC000000900185 | 92.5216 | 6345 | ZINC000100776280 | 107.917 |
| 2983 | ZINC000000901075 | 49.6468 | 6346 | ZINC000100776285 | 106.163 |
| 2984 | ZINC000001063073 | 77.0461 | 6347 | ZINC000006020493 | 131.006 |
| 2985 | ZINC000000393724 | 91.2557 | 6348 | ZINC000001532071 | 100.672 |
| 2986 | ZINC000001597139 | 85.2765 | 6349 | ZINC000002036787 | 79.5567 |
| 2987 | ZINC000003860664 | 55.5034 | 6350 | ZINC000002036788 | 81.0641 |
| 2988 | ZINC000003860714 | 83.7927 | 6351 | ZINC000002563374 | 132.609 |
| 2989 | ZINC000000404262 | 92.2524 | 6352 | ZINC000000287668 | 102.674 |
| 2990 | ZINC000000404262 | 91.4777 | 6353 | ZINC000001081537 | 129.402 |
| 2991 | ZINC000000901514 | 47.7667 | 6354 | ZINC000004521084 | 89.0299 |
| 2992 | ZINC000001648986 | 71.9994 | 6355 | ZINC000001087345 | 85.2792 |
| 2993 | ZINC000001641012 | 48.0004 | 6356 | ZINC000004661832 | 92.0609 |
| 2994 | ZINC000001641694 | 103.881 | 6357 | ZINC000001841324 | 111.252 |
| 2995 | ZINC000001648285 | 73.0218 | 6358 | ZINC000001843472 | 64.0936 |
| 2996 | ZINC000001651948 | 70.6584 | 6359 | ZINC000001848542 | 95.7465 |
| 2997 | ZINC000001632727 | 95.8166 | 6360 | ZINC000001849750 | 89.6081 |
| 2998 | ZINC000001633880 | 104.909 | 6361 | ZINC000001532209 | 55.4472 |
| 2999 | ZINC000001635717 | 96.0835 | 6362 | ZINC000001682476 | 54.7555 |
| 3000 | ZINC000019735105 | 60.6426 | 6363 | ZINC000001848561 | 104.826 |
| 3001 | ZINC000001648325 | 104.687 | 6364 | ZINC000001850693 | 57.5245 |
| 3002 | ZINC000019735100 | 65.1739 | 6365 | ZINC000002565574 | 88.6832 |
| 3003 | ZINC000019735126 | 100.568 | 6366 | ZINC000001850692 | 61.2017 |
| 3004 | ZINC000001606157 | 71.5739 | 6367 | ZINC000001590885 | 84.3799 |
| 3005 | ZINC000001640892 | 97.6932 | 6368 | ZINC000000001624 | 144.166 |
| 3006 | ZINC000001641352 | 91.281 | 6369 | ZINC000000001651 | 93.6544 |
| 3007 | ZINC000001648281 | 84.1677 | 6370 | ZINC000001891034 | 139.941 |
| 3008 | ZINC000000968099 | 69.6706 | 6371 | ZINC000001699882 | 66.7912 |
| 3009 | ZINC000000968101 | 71.8645 | 6372 | ZINC000001699948 | 54.795 |
| 3010 | ZINC000000968134 | 95.8512 | 6373 | ZINC000001704714 | 135.154 |
| 3011 | ZINC000000968225 | 77.1126 | 6374 | ZINC000001715689 | 104.696 |
| 3012 | ZINC000001677791 | 102.951 | 6375 | ZINC000001729593 | 62.1805 |
| 3013 | ZINC000001679978 | 114.442 | 6376 | ZINC000001712677 | 73.2572 |
| 3014 | ZINC000000896830 | 56.2398 | 6377 | ZINC000000895238 | 54.5047 |
| 3015 | ZINC000001677789 | 94.9463 | 6378 | ZINC000000895240 | 59.1244 |
| 3016 | ZINC000000056459 | 95.8562 | 6379 | ZINC000000002151 | 93.9302 |
| 3017 | ZINC000001667454 | 115.972 | 6380 | ZINC000001693431 | 97.5439 |
| 3018 | ZINC000001677792 | 104.655 | 6381 | ZINC000001529437 | 51.3959 |
| 3019 | ZINC000001586750 | 79.0984 | 6382 | ZINC000004096030 | 94.6864 |
| 3020 | ZINC000001638431 | 87.9288 | 6383 | ZINC000000164363 | 97.8368 |
| 3021 | ZINC000001641697 | 110.075 | 6384 | ZINC000003861073 | 92.4727 |
| 3022 | ZINC000001648253 | 45.5279 | 6385 | ZINC000000517261 | 110.683 |
| 3023 | ZINC000001648304 | 78.7308 | 6386 | ZINC000003872686 | 123.048 |
| 3024 | ZINC000003860296 | 71.7955 | 6387 | ZINC000000105309 | 123.073 |
| 3025 | ZINC000003860817 | 62.9298 | 6388 | ZINC000000057919 | 124.508 |
| 3026 | ZINC000001850864 | 90.1201 | 6389 | ZINC000012495255 | 120.45 |
| 3027 | ZINC000002570135 | 135.151 | 6390 | ZINC000000895205 | 93.1899 |
| 3028 | ZINC000049586678 | 80.2277 | 6391 | ZINC000000895228 | 64.7617 |
| 3029 | ZINC000005781270 | 73.2974 | 6392 | ZINC000000410076 | 97.3834 |
| 3030 | ZINC000008214560 | 83.9203 | 6393 | ZINC000000895216 | 50.7668 |
| 3031 | ZINC000013408703 | 131.162 | 6394 | ZINC000012358858 | 93.068 |
| 3032 | ZINC000005999089 | 115.85 | 6395 | ZINC000012359045 | 98.8645 |
| 3033 | ZINC000004098105 | 107.622 | 6396 | ZINC000012417104 | 95.2653 |
| 3034 | ZINC000000899592 | 130.94 | 6397 | ZINC000018157343 | 80.9631 |
| 3035 | ZINC000000320695 | 92.8446 | 6398 | ZINC000018181336 | 113.217 |
| 3036 | ZINC000000477938 | 82.1649 | 6399 | ZINC000018181336 | 124.324 |
| 3037 | ZINC000000488161 | 109.672 | 6400 | ZINC000005977893 | 82.118 |
| 3038 | ZINC000000897959 | 114.459 | 6401 | ZINC000005977893 | 86.7386 |
| 3039 | ZINC000000897959 | 114.776 | 6402 | ZINC000018271950 | 102.455 |
| 3040 | ZINC000000898509 | 101.54 | 6403 | ZINC000000968246 | 83.2334 |
| 3041 | ZINC000001848558 | 105.059 | 6404 | ZINC000000968248 | 90.5796 |
| 3042 | ZINC000026832397 | 97.1009 | 6405 | ZINC000000968147 | 95.6747 |
| 3043 | ZINC000000899760 | 97.5065 | 6406 | ZINC000005911680 | 125.938 |
| 3044 | ZINC000000898952 | 117.222 | 6407 | ZINC000005911680 | 128.203 |
| 3045 | ZINC000002126785 | 131.94 | 6408 | ZINC000000394884 | 91.3367 |
| 3046 | ZINC000000901630 | 79.1366 | 6409 | ZINC000008218968 | 108.847 |
| 3047 | ZINC000000155803 | 122.73 | 6410 | ZINC000008437621 | 125.975 |
| 3048 | ZINC000013378196 | 144.879 | 6411 | ZINC000008217469 | 105.472 |
| 3049 | ZINC000000058125 | 95.3882 | 6412 | ZINC000002040989 | 126.284 |
| 3050 | ZINC000006094027 | 158.445 | 6413 | ZINC000005420992 | 92.5681 |
| 3051 | ZINC000000689683 | 124.739 | 6414 | ZINC000000968125 | 86.8269 |
| 3052 | ZINC000001664038 | 121.202 | 6415 | ZINC000000968282 | 63.7802 |
| 3053 | ZINC000001676026 | 127.555 | 6416 | ZINC000000968461 | 111.3 |
| 3054 | ZINC000001608667 | 129.156 | 6417 | ZINC000002038279 | 90.2643 |
| 3055 | ZINC000001686938 | 107.395 | 6418 | ZINC000002039906 | 102.905 |
| 3056 | ZINC000000058176 | 108.711 | 6419 | ZINC000054009854 | 63.6614 |
| 3057 | ZINC000006498737 | 120.514 | 6420 | ZINC000054009855 | 60.1081 |
| 3058 | ZINC000000402930 | 104.581 | 6421 | ZINC000002038603 | 81.7027 |
| 3059 | ZINC000000066039 | 103.167 | 6422 | ZINC000002038648 | 68.6867 |
| 3060 | ZINC000062233804 | 108.962 | 6423 | ZINC000002039358 | 93.0154 |
| 3061 | ZINC000002015646 | 101.934 | 6424 | ZINC000002039792 | 75.0947 |
| 3062 | ZINC000005344125 | 130.488 | 6425 | ZINC000002031610 | 82.4 |
| 3063 | ZINC000001872131 | 106.728 | 6426 | ZINC000002035915 | 62.5315 |
| 3064 | ZINC000004098812 | 112.143 | 6427 | ZINC000002040993 | 57.0716 |
| 3065 | ZINC000003875795 | 74.8034 | 6428 | ZINC000002041241 | 111.495 |
| 3066 | ZINC000000189892 | 127.159 | 6429 | ZINC000002031407 | 87.9633 |
| 3067 | ZINC000000164508 | 68.9985 | 6430 | ZINC000002034113 | 96.7636 |
| 3068 | ZINC000003900766 | 85.7489 | 6431 | ZINC000002034885 | 48.3566 |
| 3069 | ZINC000001679741 | 67.1547 | 6432 | ZINC000002040973 | 103.809 |
| 3070 | ZINC000014438715 | 97.2125 | 6433 | ZINC000002034321 | 66.8045 |
| 3071 | ZINC000002038687 | 52.5939 | 6434 | ZINC000053199482 | 106.57 |
| 3072 | ZINC000002039808 | 60.8094 | 6435 | ZINC000002041122 | 91.4673 |
| 3073 | ZINC000014589006 | 80.4784 | 6436 | ZINC000002041162 | 90.3802 |
| 3074 | ZINC000014589123 | 65.8166 | 6437 | ZINC000005809705 | 124.771 |
| 3075 | ZINC000014807483 | 142.839 | 6438 | ZINC000002023332 | 101.387 |
| 3076 | ZINC000014811846 | 147.825 | 6439 | ZINC000002027286 | 84.2367 |
| 3077 | ZINC000014822283 | 92.3968 | 6440 | ZINC000002029390 | 129.379 |
| 3078 | ZINC000014616888 | 92.2915 | 6441 | ZINC000053022903 | 120.741 |
| 3079 | ZINC000013526620 | 76.8322 | 6442 | ZINC000005651512 | 72.5384 |
| 3080 | ZINC000014590584 | 78.4486 | 6443 | ZINC000008216082 | 108.293 |
| 3081 | ZINC000014588965 | 107.29 | 6444 | ZINC000002041110 | 78.9128 |
| 3082 | ZINC000014811844 | 137.737 | 6445 | ZINC000002041115 | 89.329 |
| 3083 | ZINC000014590681 | 83.6249 | 6446 | ZINC000053195720 | 107.198 |
| 3084 | ZINC000014590586 | 89.1182 | 6447 | ZINC000005652324 | 66.9866 |
| 3085 | ZINC000014589774 | 118.276 | 6448 | ZINC000053193391 | 108.199 |
| 3086 | ZINC000014822379 | 67.9883 | 6449 | ZINC000053194795 | 81.2886 |
| 3087 | ZINC000014614421 | 89.035 | 6450 | ZINC000005420993 | 96.1446 |
| 3088 | ZINC000000538127 | 126.902 | 6451 | ZINC000002030900 | 94.2497 |
| 3089 | ZINC000004026870 | 95.9109 | 6452 | ZINC000002034780 | 80.2106 |
| 3090 | ZINC000001680659 | 155.003 | 6453 | ZINC000002040977 | 109.786 |
| 3091 | ZINC000001680656 | 153.171 | 6454 | ZINC000008234281 | 84.8102 |
| 3092 | ZINC000000901155 | 91.1499 | 6455 | ZINC000008234325 | 75.4917 |
| 3093 | ZINC000001850742 | 97.7778 | 6456 | ZINC000008217220 | 102.266 |
| 3094 | ZINC000049538668 | 95.8313 | 6457 | ZINC000002041042 | 61.3037 |
| 3095 | ZINC000051421382 | 88.3555 | 6458 | ZINC000002041097 | 59.2235 |
| 3096 | ZINC000002004972 | 62.2041 | 6459 | ZINC000002041243 | 114.77 |
| 3097 | ZINC000003860713 | 87.4122 | 6460 | ZINC000002034329 | 69.3469 |
| 3098 | ZINC000049538671 | 88.4976 | 6461 | ZINC000002041101 | 97.3626 |
| 3099 | ZINC000000518643 | 98.5546 | 6462 | ZINC000002041121 | 97.3347 |
| 3100 | ZINC000001609008 | 44.6274 | 6463 | ZINC000008418983 | 74.74 |
| 3101 | ZINC000049586679 | 80.3188 | 6464 | ZINC000008294954 | 67.8648 |
| 3102 | ZINC000100018178 | 63.2013 | 6465 | ZINC000002040513 | 76.157 |
| 3103 | ZINC000005440503 | 82.7985 | 6466 | ZINC000002040978 | 108.557 |
| 3104 | ZINC000052064449 | 104.445 | 6467 | ZINC000002030906 | 119.925 |
| 3105 | ZINC000001714282 | 128.07 | 6468 | ZINC000002041189 | 83.2924 |
| 3106 | ZINC000005923283 | 67.0217 | 6469 | ZINC000002041123 | 72.0596 |
| 3107 | ZINC000005956067 | 70.482 | 6470 | ZINC000051943844 | 85.068 |
| 3108 | ZINC000100033029 | 110.486 | 6471 | ZINC000052541469 | 83.4351 |
| 3109 | ZINC000000409322 | 67.2509 | 6472 | ZINC000002020097 | 72.3854 |
| 3110 | ZINC000000518644 | 102.117 | 6473 | ZINC000002566243 | 87.0038 |
| 3111 | ZINC000000899803 | 112.609 | 6474 | ZINC000002384555 | 91.225 |
| 3112 | ZINC000001319998 | 119.49 | 6475 | ZINC000002384585 | 82.615 |
| 3113 | ZINC000003641069 | 114.858 | 6476 | ZINC000002384557 | 77.7323 |
| 3114 | ZINC000001676257 | 103.865 | 6477 | ZINC000002384561 | 90.7694 |
| 3115 | ZINC000000074770 | 96.9342 | 6478 | ZINC000002384564 | 99.8934 |
| 3116 | ZINC000001850745 | 97.9505 | 6479 | ZINC000002384588 | 98.3085 |
| 3117 | ZINC000008614606 | 80.3682 | 6480 | ZINC000002384605 | 114.112 |
| 3118 | ZINC000004802475 | 85.5915 | 6481 | ZINC000002384607 | 83.1803 |
| 3119 | ZINC000004802725 | 65.5687 | 6482 | ZINC000000895354 | 52.4199 |
| 3120 | ZINC000005829557 | 82.582 | 6483 | ZINC000001529427 | 83.956 |
| 3121 | ZINC000005766979 | 83.1141 | 6484 | ZINC000001683479 | 80.2681 |
| 3122 | ZINC000005764492 | 123.748 | 6485 | ZINC000001684740 | 98.6694 |
| 3123 | ZINC000005808686 | 96.1572 | 6486 | ZINC000001685159 | 110.001 |
| 3124 | ZINC000005313579 | 84.5689 | 6487 | ZINC000001532611 | 80.5178 |
| 3125 | ZINC000005158603 | 111.815 | 6488 | ZINC000000968478 | 91.4627 |
| 3126 | ZINC000005225126 | 80.4015 | 6489 | ZINC000000056578 | 68.4695 |
| 3127 | ZINC000005179609 | 91.7966 | 6490 | ZINC000000967331 | 76.2016 |
| 3128 | ZINC000005141501 | 109.242 | 6491 | ZINC000000967511 | 89.7804 |
| 3129 | ZINC000001886965 | 149.174 | 6492 | ZINC000001584141 | 48.5505 |
| 3130 | ZINC000000388287 | 59.0969 | 6493 | ZINC000001680812 | 44.9656 |
| 3131 | ZINC000001574309 | 54.6399 | 6494 | ZINC000001689531 | 133.497 |
| 3132 | ZINC000001850928 | 63.7794 | 6495 | ZINC000001627642 | 118.009 |
| 3133 | ZINC000001851025 | 97.736 | 6496 | ZINC000005356586 | 94.3878 |
| 3134 | ZINC000017721961 | 110.649 | 6497 | ZINC000000174016 | 115.987 |
| 3135 | ZINC000017819771 | 89.8785 | 6498 | ZINC000002572265 | 107.606 |
| 3136 | ZINC000017886255 | 63.6523 | 6499 | ZINC000005187769 | 78.2401 |
| 3137 | ZINC000004096638 | 139.801 | 6500 | ZINC000005434436 | 109.228 |
| 3138 | ZINC000005528095 | 94.0582 | 6501 | ZINC000001529403 | 101.203 |
| 3139 | ZINC000000160496 | 111.178 | 6502 | ZINC000000967580 | 77.3561 |
| 3140 | ZINC000000409285 | 92.6538 | 6503 | ZINC000000967794 | 78.9863 |
| 3141 | ZINC000014854290 | 83.274 | 6504 | ZINC000000402700 | 106.701 |
| 3142 | ZINC000030726969 | 87.3243 | 6505 | ZINC000001303441 | 111.267 |
| 3143 | ZINC000008034818 | 38.8175 | 6506 | ZINC000004557101 | 143.583 |
| 3144 | ZINC000000895664 | 50.4803 | 6507 | ZINC000004577626 | 111.014 |
| 3145 | ZINC000001529413 | 61.2718 | 6508 | ZINC000004649679 | 107.019 |
| 3146 | ZINC000001608904 | 53.8689 | 6509 | ZINC000004649679 | 125.478 |
| 3147 | ZINC000000901231 | 70.2829 | 6510 | ZINC000000142824 | 74.8926 |
| 3148 | ZINC000000901320 | 85.8306 | 6511 | ZINC000001686993 | 84.5204 |
| 3149 | ZINC000000004016 | 124.38 | 6512 | ZINC000001850503 | 79.2568 |
| 3150 | ZINC000000900788 | 78.1699 | 6513 | ZINC000001850546 | 62.6602 |
| 3151 | ZINC000002109876 | 111.034 | 6514 | ZINC000001850618 | 85.5017 |
| 3152 | ZINC000000056973 | 95.9248 | 6515 | ZINC000016892206 | 77.876 |
| 3153 | ZINC000001562565 | 112.841 | 6516 | ZINC000004082316 | 85.8135 |
| 3154 | ZINC000001319187 | 98.0655 | 6517 | ZINC000004090532 | 118.386 |
| 3155 | ZINC000001410681 | 85.171 | 6518 | ZINC000002164189 | 89.8252 |
| 3156 | ZINC000001482107 | 61.4985 | 6519 | ZINC000002164365 | 88.0635 |
| 3157 | ZINC000005923633 | 78.6447 | 6520 | ZINC000002164695 | 104.668 |
| 3158 | ZINC000000391099 | 106.243 | 6521 | ZINC000002168242 | 70.1235 |
| 3159 | ZINC000000900177 | 88.1634 | 6522 | ZINC000039070276 | 82.3153 |
| 3160 | ZINC000000901172 | 46.9767 | 6523 | ZINC000004556034 | 76.9096 |
| 3161 | ZINC000015120479 | 110.118 | 6524 | ZINC000001482164 | 86.9582 |
| 3162 | ZINC000000896812 | 77.1552 | 6525 | ZINC000000901159 | 40.6826 |
| 3163 | ZINC000000897142 | 72.2491 | 6526 | ZINC000000968233 | 98.0126 |
| 3164 | ZINC000100778903 | 134.342 | 6527 | ZINC000003795098 | 113.202 |
| 3165 | ZINC000000403494 | 82.9831 | 6528 | ZINC000000000882 | 89.927 |
| 3166 | ZINC000008616085 | 84.1977 | 6529 | ZINC000000000882 | 84.1531 |
| 3167 | ZINC000001606156 | 72.3373 | 6530 | ZINC000000049154 | 94.1717 |
| 3168 | ZINC000001609418 | 114.046 | 6531 | ZINC000003925823 | 135.892 |
| 3169 | ZINC000008214515 | 35.9869 | 6532 | ZINC000001693679 | 98.6873 |
| 3170 | ZINC000001532814 | 83.5872 | 6533 | ZINC000001531621 | 79.2809 |
| 3171 | ZINC000008234271 | 122.047 | 6534 | ZINC000000895327 | 104.084 |
| 3172 | ZINC000100018343 | 134.004 | 6535 | ZINC000038642928 | 101.225 |
| 3173 | ZINC000001531699 | 83.0806 | 6536 | ZINC000005759008 | 92.6556 |
| 3174 | ZINC000001532127 | 63.2493 | 6537 | ZINC000038141455 | 108.721 |
| 3175 | ZINC000100781734 | 78.9349 | 6538 | ZINC000038647448 | 99.5115 |
| 3176 | ZINC000005752097 | 80.1575 | 6539 | ZINC000014725429 | 137.577 |
| 3177 | ZINC000005761204 | 92.7948 | 6540 | ZINC000000086978 | 110.825 |
| 3178 | ZINC000005732866 | 102.842 | 6541 | ZINC000000087933 | 91.495 |
| 3179 | ZINC000005758885 | 87.8512 | 6542 | ZINC000000114121 | 84.9544 |
| 3180 | ZINC000004046820 | 102.293 | 6543 | ZINC000001529215 | 79.9567 |
| 3181 | ZINC000000901115 | 118.104 | 6544 | ZINC000004097376 | 124 |
| 3182 | ZINC000000001852 | 88.4569 | 6545 | ZINC000001697652 | 113.736 |
| 3183 | ZINC000000035525 | 89.2422 | 6546 | ZINC000000394662 | 89.274 |
| 3184 | ZINC000005998558 | 130.169 | 6547 | ZINC000013130932 | 105.385 |
| 3185 | ZINC000001095311 | 72.2318 | 6548 | ZINC000004706612 | 44.0925 |
| 3186 | ZINC000000057733 | 100.256 | 6549 | ZINC000004743114 | 110.805 |
| 3187 | ZINC000000057754 | 89.1804 | 6550 | ZINC000004692530 | 64.7107 |
| 3188 | ZINC000002011664 | 79.3507 | 6551 | ZINC000004706381 | 124.506 |
| 3189 | ZINC000000043479 | 123.462 | 6552 | ZINC000004706588 | 47.6225 |
| 3190 | ZINC000000043479 | 122.694 | 6553 | ZINC000004720638 | 58.9457 |
| 3191 | ZINC000000056507 | 108.957 | 6554 | ZINC000004742853 | 125.777 |
| 3192 | ZINC000000056531 | 99.4622 | 6555 | ZINC000095910864 | 94.9005 |
| 3193 | ZINC000000057020 | 83.4018 | 6556 | ZINC000012153091 | 84.2918 |
| 3194 | ZINC000000057722 | 104.505 | 6557 | ZINC000013143502 | 70.8443 |
| 3195 | ZINC000000120502 | 98.6174 | 6558 | ZINC000100018612 | 87.4307 |
| 3196 | ZINC000000152578 | 78.4458 | 6559 | ZINC000012153090 | 79.6181 |
| 3197 | ZINC000000155721 | 77.268 | 6560 | ZINC000100779837 | 105.512 |
| 3198 | ZINC000000157016 | 87.3715 | 6561 | ZINC000049538595 | 71.2422 |
| 3199 | ZINC000000158145 | 74.9408 | 6562 | ZINC000049708061 | 88.0473 |
| 3200 | ZINC000000163859 | 112.652 | 6563 | ZINC000004521740 | 97.2836 |
| 3201 | ZINC000000265476 | 124.704 | 6564 | ZINC000033839258 | 80.9507 |
| 3202 | ZINC000000338143 | 90.7594 | 6565 | ZINC000033839259 | 84.5771 |
| 3203 | ZINC000000388232 | 83.7325 | 6566 | ZINC000003831031 | 81.9063 |
| 3204 | ZINC000000388712 | 74.3066 | 6567 | ZINC000004655109 | 86.7732 |
| 3205 | ZINC000000391792 | 71.1854 | 6568 | ZINC000005029089 | 84.8312 |
| 3206 | ZINC000000393781 | 97.7332 | 6569 | ZINC000004521741 | 95.1981 |
| 3207 | ZINC000000394966 | 102.829 | 6570 | ZINC000014918954 | 126.442 |
| 3208 | ZINC000000402696 | 122.167 | 6571 | ZINC000018145794 | 96.4253 |
| 3209 | ZINC000000002501 | 84.7281 | 6572 | ZINC000001684877 | 94.9695 |
| 3210 | ZINC000000003183 | 64.1184 | 6573 | ZINC000012495988 | 54.2421 |
| 3211 | ZINC000000004042 | 71.312 | 6574 | ZINC000000157424 | 77.4515 |
| 3212 | ZINC000000019726 | 72.2382 | 6575 | ZINC000000155456 | 78.8672 |
| 3213 | ZINC000000035111 | 97.9624 | 6576 | ZINC000012494671 | 95.8153 |
| 3214 | ZINC000000120329 | 106.692 | 6577 | ZINC000012405152 | 54.6523 |
| 3215 | ZINC000000154632 | 99.6682 | 6578 | ZINC000000157405 | 86.8461 |
| 3216 | ZINC000000156963 | 102.918 | 6579 | ZINC000000157429 | 78.7443 |
| 3217 | ZINC000000157467 | 66.4791 | 6580 | ZINC000000391177 | 95.9141 |
| 3218 | ZINC000000159604 | 73.5371 | 6581 | ZINC000012494524 | 103.796 |
| 3219 | ZINC000000192311 | 100.559 | 6582 | ZINC000003999315 | 96.3569 |
| 3220 | ZINC000000334460 | 65.9379 | 6583 | ZINC000006484559 | 138.717 |
| 3221 | ZINC000000346082 | 125.081 | 6584 | ZINC000000057672 | 114.091 |
| 3222 | ZINC000000388118 | 58.6857 | 6585 | ZINC000000008046 | 103.105 |
| 3223 | ZINC000000389504 | 90.4549 | 6586 | ZINC000020111233 | 126.439 |
| 3224 | ZINC000000393723 | 108.314 | 6587 | ZINC000020058065 | 91.7395 |
| 3225 | ZINC000000394686 | 94.0189 | 6588 | ZINC000000409284 | 92.7828 |
| 3226 | ZINC000000397608 | 121.917 | 6589 | ZINC000004775613 | 71.5518 |
| 3227 | ZINC000006761039 | 75.881 | 6590 | ZINC000064219762 | 117.16 |
| 3228 | ZINC000006761088 | 77.1283 | 6591 | ZINC000019230125 | 86.4982 |
| 3229 | ZINC000008100931 | 69.0413 | 6592 | ZINC000034689286 | 63.3044 |
| 3230 | ZINC000008221136 | 97.3308 | 6593 | ZINC000034781669 | 63.4592 |
| 3231 | ZINC000008221195 | 99.0211 | 6594 | ZINC000003875798 | 93.6089 |
| 3232 | ZINC000008234343 | 121.76 | 6595 | ZINC000003876073 | 129.424 |
| 3233 | ZINC000008437745 | 42.031 | 6596 | ZINC000003881595 | 77.0274 |
| 3234 | ZINC000008437752 | 107.408 | 6597 | ZINC000000001080 | 97.7816 |
| 3235 | ZINC000008579417 | 100.11 | 6598 | ZINC000001628334 | 103.585 |
| 3236 | ZINC000008580042 | 86.167 | 6599 | ZINC000001666990 | 86.3034 |
| 3237 | ZINC000008580496 | 82.1174 | 6600 | ZINC000001663923 | 68.2213 |
| 3238 | ZINC000008582046 | 95.3825 | 6601 | ZINC000001672943 | 73.0292 |
| 3239 | ZINC000008616482 | 101.745 | 6602 | ZINC000000901303 | 54.1171 |
| 3240 | ZINC000008655241 | 115.893 | 6603 | ZINC000000901461 | 143.856 |
| 3241 | ZINC000008662732 | 144.215 | 6604 | ZINC000000901834 | 87.217 |
| 3242 | ZINC000008662733 | 96.3757 | 6605 | ZINC000001666984 | 73.2251 |
| 3243 | ZINC000008681596 | 133.391 | 6606 | ZINC000001677780 | 88.4107 |
| 3244 | ZINC000008728204 | 69.1305 | 6607 | ZINC000000409176 | 82.0237 |
| 3245 | ZINC000008801890 | 83.7939 | 6608 | ZINC000000409328 | 81.0048 |
| 3246 | ZINC000008844372 | 139.791 | 6609 | ZINC000000968028 | 83.5165 |
| 3247 | ZINC000008844373 | 144.435 | 6610 | ZINC000003875797 | 98.6271 |
| 3248 | ZINC000009280616 | 93.7489 | 6611 | ZINC000000895210 | 69.8023 |
| 3249 | ZINC000000051923 | 119.753 | 6612 | ZINC000001671473 | 76.2156 |
| 3250 | ZINC000000056528 | 104.617 | 6613 | ZINC000001529401 | 46.2871 |
| 3251 | ZINC000000057736 | 82.1897 | 6614 | ZINC000001529424 | 61.9995 |
| 3252 | ZINC000000119978 | 142.226 | 6615 | ZINC000003882101 | 116.951 |
| 3253 | ZINC000000388039 | 78.3583 | 6616 | ZINC000001529484 | 95.2168 |
| 3254 | ZINC000000388713 | 73.8717 | 6617 | ZINC000003861150 | 120.919 |
| 3255 | ZINC000000393808 | 108.647 | 6618 | ZINC000003975659 | 126.766 |
| 3256 | ZINC000006071289 | 68.3547 | 6619 | ZINC000004097406 | 116.102 |
| 3257 | ZINC000006071880 | 89.3259 | 6620 | ZINC000008585900 | 107.202 |
| 3258 | ZINC000006072081 | 63.495 | 6621 | ZINC000001672966 | 102.117 |
| 3259 | ZINC000006093290 | 116.457 | 6622 | ZINC000001555566 | 73.6392 |
| 3260 | ZINC000006094187 | 146.796 | 6623 | ZINC000013456778 | 134.044 |
| 3261 | ZINC000006205881 | 103.249 | 6624 | ZINC000013452167 | 66.0956 |
| 3262 | ZINC000006411540 | 127.62 | 6625 | ZINC000001680739 | 63.9381 |
| 3263 | ZINC000006411540 | 130.855 | 6626 | ZINC000001680824 | 73.2915 |
| 3264 | ZINC000006437474 | 87.0362 | 6627 | ZINC000000394334 | 88.2244 |
| 3265 | ZINC000006458015 | 146.713 | 6628 | ZINC000001670837 | 66.3459 |
| 3266 | ZINC000006472605 | 136.063 | 6629 | ZINC000096321491 | 86.3746 |
| 3267 | ZINC000006472605 | 143.495 | 6630 | ZINC000001644079 | 94.3212 |
| 3268 | ZINC000006523722 | 103.891 | 6631 | ZINC000005408229 | 112.814 |
| 3269 | ZINC000006536390 | 136.644 | 6632 | ZINC000002040169 | 86.2332 |
| 3270 | ZINC000006536390 | 139.416 | 6633 | ZINC000014587710 | 93.8684 |
| 3271 | ZINC000000391851 | 89.227 | 6634 | ZINC000013508950 | 110.646 |
| 3272 | ZINC000001850064 | 112.044 | 6635 | ZINC000013508950 | 98.8357 |
| 3273 | ZINC000001850211 | 78.6413 | 6636 | ZINC000013508949 | 108.55 |
| 3274 | ZINC000001850438 | 69.8552 | 6637 | ZINC000013508949 | 96.4043 |
| 3275 | ZINC000001850677 | 66.222 | 6638 | ZINC000013512214 | 71.1456 |
| 3276 | ZINC000001850769 | 106.249 | 6639 | ZINC000014723523 | 90.3352 |
| 3277 | ZINC000001850815 | 104.593 | 6640 | ZINC000002038799 | 95.655 |
| 3278 | ZINC000001850892 | 83.1724 | 6641 | ZINC000002038899 | 55.9554 |
| 3279 | ZINC000001853865 | 77.08 | 6642 | ZINC000002039285 | 90.7263 |
| 3280 | ZINC000002004617 | 85.0498 | 6643 | ZINC000014588455 | 83.8666 |
| 3281 | ZINC000002010676 | 73.0969 | 6644 | ZINC000013545799 | 122.546 |
| 3282 | ZINC000002015842 | 79.1618 | 6645 | ZINC000014589005 | 80.6612 |
| 3283 | ZINC000006018751 | 110.773 | 6646 | ZINC000012343956 | 126.317 |
| 3284 | ZINC000006019397 | 102.736 | 6647 | ZINC000002043543 | 97.0452 |
| 3285 | ZINC000006031263 | 53.5354 | 6648 | ZINC000013543038 | 84.0694 |
| 3286 | ZINC000006037875 | 55.2692 | 6649 | ZINC000002040187 | 71.1847 |
| 3287 | ZINC000006067281 | 77.5816 | 6650 | ZINC000014438663 | 103.117 |
| 3288 | ZINC000006068630 | 87.7547 | 6651 | ZINC000014588868 | 83.0494 |
| 3289 | ZINC000006068808 | 108.014 | 6652 | ZINC000013549494 | 102.725 |
| 3290 | ZINC000001850419 | 79.9589 | 6653 | ZINC000006071066 | 111.12 |
| 3291 | ZINC000001850423 | 92.528 | 6654 | ZINC000025695693 | 67.272 |
| 3292 | ZINC000001850454 | 102.043 | 6655 | ZINC000025723415 | 116.794 |
| 3293 | ZINC000001850685 | 68.3226 | 6656 | ZINC000025695868 | 90.9492 |
| 3294 | ZINC000001850820 | 113.337 | 6657 | ZINC000014591922 | 72.6025 |
| 3295 | ZINC000001857526 | 103.77 | 6658 | ZINC000013516321 | 88.4593 |
| 3296 | ZINC000001995154 | 62.139 | 6659 | ZINC000013516321 | 79.981 |
| 3297 | ZINC000002002624 | 63.7608 | 6660 | ZINC000014590682 | 92.5321 |
| 3298 | ZINC000002010941 | 97.8329 | 6661 | ZINC000002242702 | 98.1455 |
| 3299 | ZINC000002015545 | 105.087 | 6662 | ZINC000004410593 | 78.5204 |
| 3300 | ZINC000002015849 | 56.17 | 6663 | ZINC000004492895 | 126.285 |
| 3301 | ZINC000005821675 | 68.6824 | 6664 | ZINC000004492895 | 112.882 |
| 3302 | ZINC000005821996 | 69.7715 | 6665 | ZINC000004521558 | 64.0068 |
| 3303 | ZINC000005839894 | 73.5833 | 6666 | ZINC000004528568 | 101.448 |
| 3304 | ZINC000005843219 | 74.5221 | 6667 | ZINC000004658567 | 54.8245 |
| 3305 | ZINC000005851109 | 104.609 | 6668 | ZINC000002242686 | 87.6943 |
| 3306 | ZINC000005859351 | 91.2057 | 6669 | ZINC000004343508 | 116.591 |
| 3307 | ZINC000005863164 | 117.167 | 6670 | ZINC000004353766 | 86.0608 |
| 3308 | ZINC000005863711 | 81.0328 | 6671 | ZINC000004353766 | 84.6876 |
| 3309 | ZINC000005922165 | 85.7605 | 6672 | ZINC000004430890 | 97.3522 |
| 3310 | ZINC000005933618 | 104.544 | 6673 | ZINC000004655107 | 121.564 |
| 3311 | ZINC000005933671 | 109.093 | 6674 | ZINC000039365143 | 88.5403 |
| 3312 | ZINC000005934859 | 101.694 | 6675 | ZINC000004501352 | 46.6551 |
| 3313 | ZINC000005956065 | 80.7999 | 6676 | ZINC000004521480 | 70.6248 |
| 3314 | ZINC000005956077 | 72.4373 | 6677 | ZINC000004528590 | 86.4685 |
| 3315 | ZINC000005998163 | 70.4403 | 6678 | ZINC000004623761 | 81.5226 |
| 3316 | ZINC000005999105 | 98.1243 | 6679 | ZINC000039365142 | 86.8481 |
| 3317 | ZINC000001850213 | 120.103 | 6680 | ZINC000004538008 | 124.906 |
| 3318 | ZINC000001850294 | 82.7643 | 6681 | ZINC000000057905 | 112.944 |
| 3319 | ZINC000001850772 | 113.066 | 6682 | ZINC000001687319 | 70.3217 |
| 3320 | ZINC000001850817 | 104.325 | 6683 | ZINC000001734221 | 85.9833 |
| 3321 | ZINC000002002623 | 90.5704 | 6684 | ZINC000034276222 | 84.6945 |
| 3322 | ZINC000002005192 | 61.9506 | 6685 | ZINC000001747064 | 74.2297 |
| 3323 | ZINC000002010910 | 88.3532 | 6686 | ZINC000000526834 | 99.4766 |
| 3324 | ZINC000002015151 | 138.215 | 6687 | ZINC000006037768 | 90.9822 |
| 3325 | ZINC000002015152 | 133.507 | 6688 | ZINC000006037906 | 62.3183 |
| 3326 | ZINC000005688568 | 84.5143 | 6689 | ZINC000006037750 | 72.7176 |
| 3327 | ZINC000005759086 | 114.758 | 6690 | ZINC000006030257 | 124.061 |
| 3328 | ZINC000005760081 | 80.144 | 6691 | ZINC000006037688 | 78.4488 |
| 3329 | ZINC000005761690 | 81.3932 | 6692 | ZINC000000039092 | 129.666 |
| 3330 | ZINC000005761901 | 88.0719 | 6693 | ZINC000000120249 | 81.3153 |
| 3331 | ZINC000005761926 | 89.4482 | 6694 | ZINC000026515309 | 63.6172 |
| 3332 | ZINC000005765023 | 56.1174 | 6695 | ZINC000100095425 | 128.549 |
| 3333 | ZINC000005767132 | 111.941 | 6696 | ZINC000100776178 | 84.4092 |
| 3334 | ZINC000005767143 | 112.935 | 6697 | ZINC000006031279 | 61.2518 |
| 3335 | ZINC000005819412 | 95.4764 | 6698 | ZINC000008613011 | 88.0765 |
| 3336 | ZINC000005819503 | 73.2437 | 6699 | ZINC000006018386 | 135.254 |
| 3337 | ZINC000005820491 | 97.2711 | 6700 | ZINC000006018556 | 68.4755 |
| 3338 | ZINC000005820551 | 94.7272 | 6701 | ZINC000005651192 | 73.2425 |
| 3339 | ZINC000005820603 | 95.7063 | 6702 | ZINC000004096672 | 117.782 |
| 3340 | ZINC000001850318 | 77.8259 | 6703 | ZINC000100267306 | 84.5373 |
| 3341 | ZINC000001850395 | 73.9522 | 6704 | ZINC000004096578 | 124.245 |
| 3342 | ZINC000001850422 | 91.7278 | 6705 | ZINC000004623758 | 70.7207 |
| 3343 | ZINC000001850427 | 86.0187 | 6706 | ZINC000100067274 | 115.1 |
| 3344 | ZINC000001850435 | 104.632 | 6707 | ZINC000100067274 | 119.249 |
| 3345 | ZINC000001850458 | 104.849 | 6708 | ZINC000001589916 | 94.7634 |
| 3346 | ZINC000001850686 | 61.1362 | 6709 | ZINC000001532231 | 96.6896 |
| 3347 | ZINC000001850839 | 73.6599 | 6710 | ZINC000001532860 | 116.194 |
| 3348 | ZINC000001850933 | 97.7658 | 6711 | ZINC000001586752 | 73.3092 |
| 3349 | ZINC000001867127 | 61.5784 | 6712 | ZINC000001586788 | 99.2914 |
| 3350 | ZINC000001995170 | 116.633 | 6713 | ZINC000001529451 | 75.7581 |
| 3351 | ZINC000002003567 | 71.5777 | 6714 | ZINC000001481898 | 88.0161 |
| 3352 | ZINC000002011247 | 84.3279 | 6715 | ZINC000017004078 | 70.6316 |
| 3353 | ZINC000005225119 | 79.8209 | 6716 | ZINC000000164490 | 81.6167 |
| 3354 | ZINC000005227203 | 57.97 | 6717 | ZINC000000164685 | 69.2653 |
| 3355 | ZINC000005356284 | 98.2196 | 6718 | ZINC000000163492 | 103.043 |
| 3356 | ZINC000005360197 | 67.553 | 6719 | ZINC000002168243 | 93.8471 |
| 3357 | ZINC000005385354 | 94.3788 | 6720 | ZINC000002169156 | 54.5798 |
| 3358 | ZINC000005447650 | 94.5734 | 6721 | ZINC000002243021 | 96.9161 |
| 3359 | ZINC000005456169 | 94.8211 | 6722 | ZINC000004652497 | 72.7384 |
| 3360 | ZINC000005457663 | 76.5386 | 6723 | ZINC000038139289 | 116.14 |
| 3361 | ZINC000005486355 | 122.45 | 6724 | ZINC000000405329 | 96.7447 |
| 3362 | ZINC000005512462 | 108.342 | 6725 | ZINC000000001798 | 91.4345 |
| 3363 | ZINC000005512602 | 108.828 | 6726 | ZINC000000898100 | 118.592 |
